# Unraveling the Influence of Elevation on Moss Species–Area Relationships and the Effect of Spatial Scale on Elevational Richness Patterns in Mt Wutai With a Nested‐Plot Sampling Design

**DOI:** 10.1002/ece3.73473

**Published:** 2026-04-10

**Authors:** Haozhe Wang, Fenghua Wang, Yu Zhao, Chenglong Li, Xiaowei Ma, Xiaopan Wang, Lina Zhang, De Gao

**Affiliations:** ^1^ College of Geographical Sciences Hebei Normal University Shijiazhuang China; ^2^ Foreign Language Teaching Department Changzhi Medical College Changzhi China; ^3^ School of Ecology Hainan University Haikou China

**Keywords:** annual mean temperature, boosted regression trees, mosses, net primary productivity, normalized difference vegetation index, precipitation of driest month, the mid‐domain effect

## Abstract

Understanding how species–area relationships (SARs) vary with elevation as well as how elevational richness patterns vary across spatial scales is critical for biodiversity conservation in montane systems. On Mt Wutai of China, we sampled sets of nested plots and collected moss species along the elevational gradient. We examined how the *c*‐value (richness per unit area) and *z*‐value (rate of richness increase with area) of SARs vary with elevation and how elevational richness patterns vary under different scales. We analyzed the driving factors behind the variation of *c*‐ and *z*‐values along the elevational gradient as well as the variation of elevational richness patterns across spatial scales through a machine learning method. Last, we explored how the driving factors of elevational richness patterns vary with spatial scale. We found a positively skewed hump‐shaped pattern in *c*‐values along the elevational gradient and a monotonic increasing trend in *z*‐values with rising elevation. NPP and precipitation of the driest month (Bio14) were the most influential predictors for the variation of *c*‐ and *z*‐values, respectively. A positively skewed hump‐shaped pattern in species richness along the elevational gradient was found at small spatial scales, whereas a decelerating increasing trend with a less distinct mid‐elevation peak was found at larger spatial scales. A stronger relationship between elevational richness pattern and environmental variables was detected as sampling scale increased. With increasing spatial scale, the relative importance of the mid‐domain effect and Bio14 declined, whereas that of NDVI, NPP, and annual temperature range rose significantly when explaining variations in species richness. The scale‐dependent elevational richness patterns of mosses, marked by a fine‐scale mid‐elevation peak and shifting predictor importance, are driven by their sensitivity to microhabitat, climate, energy, and scale‐dependent ecological processes. Given scale‐dependent elevational richness patterns, we emphasize the need to establish an integrated “large‐scale guiding, small‐scale refining” conservation framework.

## Graphical abstract

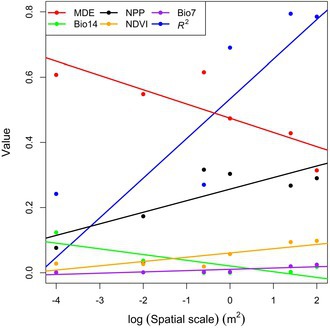

## 1 Introduction

Mountains are crucial in biodiversity research due to their unique ecological, environmental, and geographical characteristics. First, mountains act as isolated habitat islands, restricting species movement due to steep slopes, climate gradients, and geographic barriers. This isolation effect may drive adaptive radiation and speciation, leading to a high proportion of endemic species (Flantua et al. 2020). Second, mountains exhibit rapid changes in altitude, temperature, precipitation, and oxygen levels over short distances, which create diverse microhabitats, supporting a wide range of species adapted to specific conditions (Hodkinson 2005). Third, during past climate shifts (e.g., ice ages), mountains often served as refugia where species survived when lowland areas became uninhabitable. Many mountain‐dwelling species thus have ancient lineages, providing insights into historical biodiversity patterns, extinction risks, and long‐term ecological resilience (Michalak et al. 2018). In summary, harboring a large number of endemic, rare and threatened species, mountains are often recognized as global biodiversity hotspots (Körner and Spehn 2002; Socolar et al. 2016) and offer unparalleled opportunities to study biodiversity patterns, evolutionary processes, and ecological responses to environmental change, making them indispensable in global biodiversity research (Fu et al. 2023, 2024; Gao et al. 2021). In mountain biodiversity research, how species–area relationships (SARs) vary along elevational gradients and how elevational richness patterns are affected by study scales are two important research topics, and they play a pivotal role in the formulation of biodiversity conservation strategies. However, current research on these two topics remains very limited.

The SAR pattern is a fundamental law in ecology, indicating that species richness tends to increase with the size of the study area (Lomolino 2000; Triantis et al. 2012). This relationship plays a vital role in conservation biology and applied ecology, frequently used to identify biodiversity hotspots (Fattorini 2007), optimize the spatial configuration of protected areas (Neigel 2003), and assess extinction risks due to habitat loss (Harcourt and Doherty 2005). The species–area relationship is generally expressed as a power function: *S* = *cA* ^*z*^, where *S* represents species richness, *A* denotes area, and *c* and *z* are model parameters (Arrhenius 1921). This model structure is simple, requiring only two parameters with clear ecological meaning, and has thus been widely adopted and well‐interpreted in practical research (Fattorini et al. 2017; Matthews et al. 2016). Parameter *c* reflects the average number of species per unit area, while *z* measures the rate at which species numbers increase with area. Although numerous studies have compared *c*‐ and *z*‐values across different taxonomic groups and ecosystems (Fattorini et al. 2017; Matthews et al. 2016), the influence of ecological drivers on these parameters and whether they exhibit systematic variation patterns remain understudied. In particular, studies investigating how SARs vary along elevational gradients are scarce. The only two existing studies have also produced inconsistent results. Baumann et al. (2016) observed that *z*‐values for nested plots (ranging from 0.0001 to 100 m^2^) were positively influenced by elevation in alpine grassland vegetation. Whereas, in their study of the SARs of vascular plants along the elevational gradient of the Alborz Mountains, Iran, Moradi et al. (2020) demonstrated that *z*‐values were positively influenced by temperature and soil nitrogen, while decreasing with elevation. Meanwhile, *c*‐values were positively influenced by temperature and soil nitrogen, negatively by rock cover, and also decreased with elevation.

Understanding the elevational richness patterns has been a fundamental yet controversial topic in biogeography, ecology and biodiversity conservation (Lomolino 2001; Sanders and Rahbek 2012). Studies across diverse biological groups and topographic gradients reveal several characteristic patterns in species richness distribution along altitudinal gradients, with the most common being positively skewed (hump‐shaped) and monotonically decreasing patterns (Fu et al. 2023; Zhou et al. 2019). To explain the causes of these patterns, researchers have proposed multiple hypotheses. The habitat complexity hypothesis suggests that more structurally complex habitats provide greater ecological niche availability, thereby supporting higher species richness (Brown 2001; Wu et al. 2013). The energy hypothesis emphasizes the positive promotion of diversity by environmental energy levels and productivity (Hawkins et al. 2003; O'Brien 2006). The mid‐domain effect (MDE) posits that species distributed randomly within a finite geographic range exhibit maximum overlap in their mid‐range distribution, leading to a peak in species numbers at mid‐range (Colwell and Hurtt 1994; Colwell and Lees 2000). Furthermore, the environmental hypothesis proposes that current climatic conditions—such as precipitation, temperature, and water availability—constitute key limiting factors influencing terrestrial community species richness and shaping their altitudinal distribution patterns (Heaney 2001; McCain 2007; Sánchez‐Cordero 2001). Most research on elevational richness patterns has focused primarily on the demonstration of such patterns and the testing of hypotheses; however, studies exploring how elevational richness patterns are influenced by study scales remain sparse. Recent studies highlight the importance of spatial scale in sampling montane biodiversity, stressing its role in interpreting elevational richness patterns and their driving mechanisms (Dáttilo et al. 2023; Graham et al. 2014; Montes et al. 2021). For example, Rahbek (2005) noted that when analyzing at different spatial scales, the peak in species richness observed at mid‐to‐high elevations undergoes significant changes, potentially even exhibiting completely opposite trends. Similarly, McCain and Grytnes (2010) confirmed that the hump‐shaped distribution of species richness along elevation gradients is only prevalent at large spatial scales. At more local scales, the distribution pattern exhibits greater variability, manifested as shifts in peak locations or complete disappearance of peaks. Furthermore, Montes et al. (2021) investigated the variability of biodiversity across different spatial scales and proposed potential pathways for optimizing scale‐aware biodiversity sampling strategies.

Although the elevational richness patterns and SARs have been widely examined separately (Moradi et al. 2020; Onditi et al. 2024), few studies have explicitly integrated elevational gradients, scale‐dependent shifts in richness pattern shape, and scale‐dependent changes in the relative importance of environmental drivers within a single system. This conceptual gap remains particularly pronounced for montane mosses, for which the combined elevational and scale dependencies of SARs and richness patterns remain poorly understood. Mosses are exceptionally well‐suited to fill this gap, for two key reasons. First, montane mosses support high species richness and are highly sensitive to climatic and microenvironmental changes (Turetsky et al. 2012; Kou et al. 2020). Second, their low resource requirements enable robust scale‐dependent research using relatively small sampling units (He et al. 2016): a 100 m^2^ plot can host tens of co‐occurring moss species (Fu et al. 2023, 2024; Gao et al. 2021), whereas vertebrate and many vascular plant assemblages would require orders‐of‐magnitude larger areas to yield comparable species numbers. Our nested plot design further allows us to disentangle two distinct forms of scale dependence: (1) scale dependence in the form of elevational richness patterns (e.g., mid‐elevation peak shift, flattening, or disappearance), and (2) scale dependence in the relative importance of environmental predictors. Based on bryophyte ecological theory and our nested sampling design, we propose the following testable predictions: (1) SAR *c*‐ and *z*‐values will show consistent directional shifts across the elevational gradient, driven by climatic and microenvironmental variables associated with water availability and temperature seasonality. (2) The elevational richness pattern will shift from a hump‐shaped pattern at fine scales toward a weaker or flattened pattern at broad scales, due to the integration of microrefugia and reduced dispersal limitation in larger plots. (3) The relative importance of climatic predictors will shift with scale: fine‐scale richness will be more strongly driven by moisture‐related variables (e.g., drought stress), whereas broad‐scale richness will be better explained by temperature seasonality and vegetation productivity. (4) The strength of elevational richness–environment relationships will increase with spatial scale, as larger plots reduce microenvironmental stochasticity and better represent broad climatic gradients. Using nested plots along elevational gradients on Mt Wutai, we constructed nested moss SARs and scale‐dependent elevational richness patterns to evaluate these predictions and clarify the multi‐scale drivers of moss biodiversity in mountain ecosystems.

## 2 Materials and Methods

### 2.1 Study Area

**FIGURE 1:**
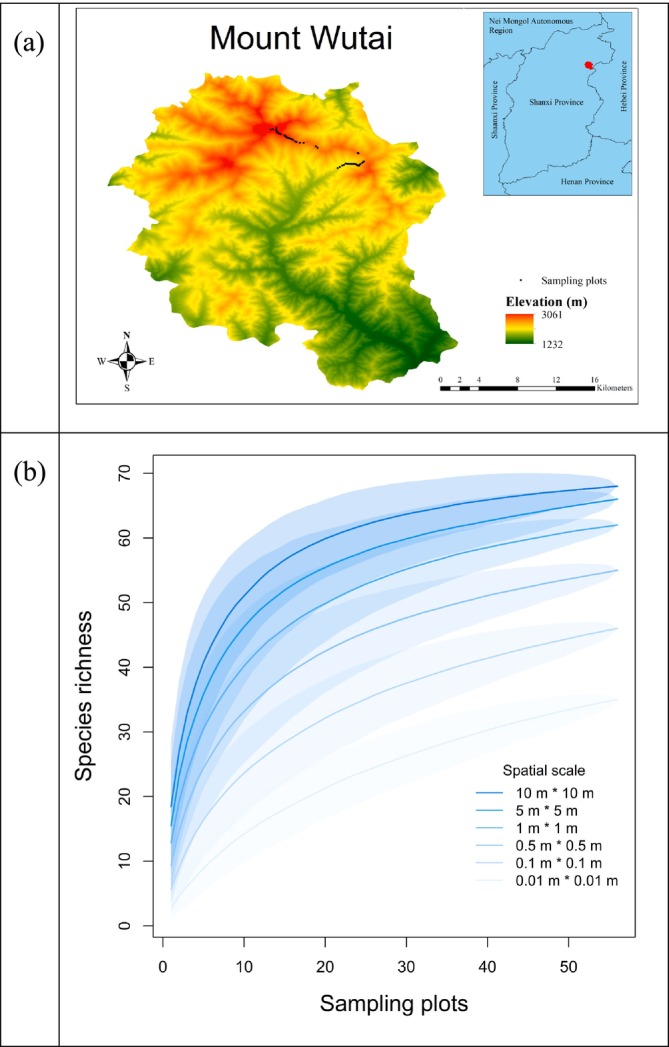
Map of the Mt Wutai, showing the distribution of 56 sampling plots (a) and species richness rarefaction at six spatial scales (b). Shaded areas represent 95% confidence intervals.

This study was conducted on Mt Wutai (38°55′–39°66′ N, 113°29′–113°39′ E; maximum elevation 3061 m a.s.l.), a United Nations Educational, Scientific and Cultural Organization (UNESCO) World Heritage Site and one of the Four Sacred Mountains of Chinese Buddhism. Located in Xinzhou City, Shanxi Province, China, it forms the northern terminus of the Taihang Mountain Range. The region has a continental alpine climate with a mean annual temperature of −4°C, peaking in warmth during July–August (average maximum: 9.5°C) and reaching extreme cold in January (mean temperature: −18.8°C). The summit, North Terrace Peak, stands at 3061 m a.s.l., making it the highest point in northern China's Huabei Region and earning it the epithet “Roof of Northern China” (Figure 1a).

### 2.2 Sampling and Species Identification

56 plots were sampled at c. 20 m elevational intervals and over 100 m apart between each other from 1960 to 3060 m a.s.l. during July–August 2024 on Mt Wutai, covering four main environments: grassland, forest, alpine meadow, and alpine rocky meadow. In each interval, we took at random one square plot of 100 m^2^ (i.e., 10 m × 10 m). From one corner of each 10 m × 10 m square plot, a series of nested subplots (Type I curves; Scheiner 2003) of 0.0001, 0.01, 0.25, 1, and 25 m^2^ were sampled for the presence of moss species. Within each plot and subplot, we collected all moss specimens growing from the ground surface up to a height of 2 m (Tables S14–S19, Appendix S3). Subsequently, in the laboratory of the College of Geographical Sciences, Hebei Normal University, these specimens underwent species identification using stereomicroscopes and optical microscopes. All species names were assigned according to relevant literature (Gao 1994a, 1994b, 2003; Gao and Lai 2003; Gao and Wu 2008; Hu and Wang 2005; Li 2000, 2006; Wu 2002; Wu and Jia 2004).

### 2.3 Ecological Variables

Mean annual temperature (Bio1, in °C), minimum temperature of the coldest month (Bio6, in °C), annual temperature range (Bio7, in °C), annual precipitation (Bio12, in mm), precipitation of the driest month (Bio14, in mm), and wind speed (WS, in m/s) were obtained from the WorldClim v2 database (Fick and Hijmans 2017) as 30‐arc‐second (≈1 km^2^) digital maps (the raster resolution assigned to the plots) and extracted for each plot using ArcGIS 10.2 (ESRI). These bioclimatic variables were selected because they are especially biologically meaningful for mosses, which lack true roots and are highly sensitive to thermal and water regimes. Productivity metrics were obtained from the Moderate Resolution Imaging Spectroradiometer (MODIS) satellite imagery, including the normalized difference vegetation index (NDVI; Didan 2021) and net primary productivity (NPP, in kg C/m^2^/year; Running and Zhao 2021). We classified four habitat types, including aquatic, terricolous, saxicolous, and epiphytic and counted the total number of habitat types in each plot and subplot to infer habitat diversity (HD; Table S13, Appendix S3).

In analyzing species richness distribution patterns along altitudinal gradients, this study also employed the discrete domain analysis method provided by RangeModel 5 (Colwell 2008) to estimate theoretical values for the mid‐domain effect (MDE). Using the analytical random models established by Colwell and Hurtt (1994) and Colwell and Lees (2000), 100,000 Monte Carlo simulations were performed on the observed species ranges within the defined geographic area. This process was randomized through sampling without replacement to calculate the average predicted species richness for each plot and subplot, along with their 95% confidence intervals (Figure S3).

Moss species richness and SAR parameters were measured at fine spatial grains: nested subplots from 0.0001 m^2^ up to 100 m^2^, in contrast, all climatic variables were extracted from satellite‐derived data at 30‐arc‐second resolution, representing average conditions over a 1 km × 1 km grid. Admittedly, this mismatch introduces measurement uncertainty, as key microclimatic and microhabitat factors governing mosses (including substrate moisture, light availability, and within‐plot topographic heterogeneity) were not directly measured at the plot or subplot scale. The 1 km^2^ climatic grids cannot capture fine‐scale microenvironmental variation, which may lead to discrepancies between the assigned climatic values and the actual conditions experienced by moss colonies. To account for spatial scale mismatch between biological observations and environmental variables, we conducted both drop‐one‐variable and perturbation sensitivity analyses within driver analyses for *c*‐value, *z*‐value, and species richness at each spatial scale. We first constructed the full model with all variables as the baseline. For the drop‐one‐variable sensitivity analysis, we sequentially omitted one variable at a time from the full model and refitted the model. For the perturbation sensitivity analysis, we added 5% independent random noise to each satellite‐derived variable to simulate realistic measurement uncertainty, refitted the model using the perturbed variables, and repeated this procedure for 1000 iterations. We then compared the explanatory power of all refitted models with that of the baseline model. These refitted models exhibited only modest variation in explanatory power, confirming that our model results are stable and insensitive to minor uncertainty in the satellite‐derived variables (Figure S9, Appendixes S1 and S2).

### 2.4 Data Analyses

#### 2.4.1 SAR Fitting

We used the “sars” package (Matthews et al. 2019) to fit each set of nested plots with a power function model as the species accumulation curve, thereby obtaining the *c*‐ and *z*‐values for the SARs. Given the uncertainty in *c*‐ and *z*‐values derived from SAR fitting, their standard errors (SEs) were calculated and included in subsequent generalized additive models and boosted regression trees. We used 1/SE as a weighting term, such that estimates with larger SEs received smaller weights, minimizing the impact of low‐reliability, high‐error *c*‐ and *z*‐values on model fitting. Pairwise *t*‐tests were conducted to compare *c*‐ and *z*‐values across the four environments.

#### 2.4.2 Generalized Additive Models Trends

Generalized additive models (GAMs) with a Gaussian variance function were applied to determine the trend in the response curves of *c*‐ and *z*‐values, and species richness along the elevational gradient (Bhattarai and Vetaas 2006). To further evaluate the role of spatial structure in shaping elevational patterns of species richness and SAR parameters (i.e., *c*‐ and *z*‐values), we performed a sequential analytical procedure.

First, we constructed baseline GAMs with elevation as the sole predictor. In these baseline models, a cubic smooth spline was employed as the smooth basis function for elevation, with a basis dimension (*k*) set to 5 to balance the flexibility of capturing non‐linear elevational trends and the risk of overfitting. The optimal smoothness parameters of the GAMs were selected via generalized cross‐validation (GCV), which minimizes prediction error and prevents overfitting (Craven and Wahba 1979; Hastie and Tibshirani 1990).

Next, we assessed the presence of spatial autocorrelation in the residuals of these baseline GAMs using Moran's *I* test with 999 Monte Carlo permutations (Cliff and Ord 1981; Bivand et al. 2013). Spatial weight matrices were constructed from plot latitude and longitude using a k‐nearest neighbor approach (*k* = 5). Based on the results of the Moran's *I* test, we adopted two distinct analytical strategies for different groups:

For analytical groups where no significant spatial autocorrelation was detected in the residuals, the baseline GAM (with elevation as the only predictor) was retained to characterize their elevational trends. This decision was justified by the absence of spatial structure confounding the relationship between elevation and the response variables (*c*‐values, *z*‐values, and species richness).

For groups where significant spatial autocorrelation was detected, we further compared the explained variance (*R* ^2^) among three GAM specifications to disentangle the respective effects of elevation and spatial structure on the response variables (*c*‐values, *z*‐values, and species richness): (1) Model 1 (baseline model): only elevation as the predictor; (2) Model 2: only spatial coordinates as the predictor; (3) Model 3: both elevation and spatial coordinates as predictors. If the *R* ^2^ of Model 2 was much smaller than that of Model 1, spatial autocorrelation was interpreted as a byproduct of elevational trends. In this case, the spatial structure did not exert an independent influence, and no additional adjustments to the baseline model were needed. In contrast, if Model 2 exhibited a comparable or higher *R* ^2^ relative to Model 1, spatial autocorrelation was considered an independent factor shaping the elevational patterns. To address this and avoid confounding the elevational trend with independent spatial structure, we incorporated latitude and longitude as a 2D smooth term into the GAM, thereby controlling for the effects of spatial autocorrelation in subsequent analyses (Table S12, Appendix S1).

Model diagnostics were performed to evaluate fit reliability: residual normality and homoscedasticity were verified using Q–Q plots and residual vs. fitted value plots, respectively; additionally, the percentage of deviance explained and GCV scores were monitored to further confirm the absence of overfitting (Hastie and Tibshirani 1990; Zhou et al. 2019).

#### 2.4.3 Boosted Regression Trees Predictors

Boosted regression trees (BRT) model was used to investigate the drivers of *c*‐ and *z*‐values and species richness variations along the elevational gradient. BRT is an ensemble machine learning technique that combines the characteristics of regression trees and boosting methods (Elith et al. 2008). Regression trees construct models based on recursive binary splitting, while boosting methods optimize model performance by progressively adding weak learners (i.e., tree structures) that maximize the reduction of the loss function (Hastie et al. 2009). Compared to traditional modeling approaches such as generalized linear models or generalized additive models, BRT offers a series of significant advantages: for instance, it does not require pre‐processing data transformations, exhibits strong robustness to outliers, can handle missing values using proxy variables, and automatically identifies interaction effects among predictor variables (Leathwick et al. 2006). For these reasons, we ultimately selected the BRT method, primarily for the following two reasons: First, our sample‐to‐predictor ratio is relatively small, which may challenge model stability; however, BRT is more robust to small samples and less sensitive to high predictor counts. Second, the relationship between the independent and dependent variables may not be monotonic (e.g., peaks in species richness typically occur under optimal environmental conditions, such as temperature; and temperatures that are extremely low or high are not conducive to maximizing species richness), fortunately BRT can fit complex non‐linear relationships (Elith et al. 2008; Leathwick et al. 2006; Zhang et al. 2021).

Nine environmental variables (HD, Bio1, Bio6, Bio7, Bio12, Bio14, WS, NPP, and NDVI) were used as predictive variables to explain variations in *c*‐ and *z*‐values. In contrast, MDE, along with these nine environmental variables, served as predictive variables for explaining variations in species richness. We performed BRT analyses using the “gbm.step” function in the R package dismo (Hijmans et al. 2020), and Gaussian response type was chosen, aiming at minimizing squared error. Ten‐fold cross‐validation (CV) was employed to identify the optimal number of trees to be used for each model and to subsequently assess the predictive performance of BRT. Deviance (squared error for Gaussian response type) was used as the loss function in the sequential model‐fitting process for the BRT algorithm (Friedman 2001). We fitted 360 BRT models with all possible combinations of the three meta‐parameters, including tree complexity, learning rate, and bag fraction (Tables S2–S10; Appendix S1), and the optimal settings with the lowest CV deviance were then selected to generate the final BRT (Zhang et al. 2021). To evaluate the performance of each model, we also calculated the percentage of explained deviance (pseudo‐*R* ^2^ value), and the CV correlation between the observed and predicted values. Moreover, we calculated values of root mean square error (RMSE), mean absolute error (MAE), and *R* ^2^ based on the observed and predicted values using the “Metrics” package (Hamner and Frasco 2018). To better interpret the fitted functions, we created partial dependence plots using the “gbm.plot” function in the R package gbm (Ridgeway and Developers 2024) to visualize the effects of individual predictor variables after accounting for the average effects of all other variables in the model (Friedman 2001). If the determined tree complexity exceeded one, we assessed the relative importance of all pairwise interactions with the “gbm.interactions” function and generated perspective plots (Figures S4–S7, Appendix S1) to visualize the four most important interactions via the “gbm.perspec” function (Ridgeway and Developers 2024).

#### 2.4.4 Relative Importance of BRT Predictors

Although correlations among variables (Figure S1, Appendix S1) do not affect BRT predictions, they can influence the evaluation of variables' relative importance (RI; Hastie et al. 2009). To avoid inaccurate calculation of the RI of each variable caused by multicollinearity among variables, we did not directly extract the RI values of individual variables from the final BRT model. Instead, we calculated the conditional variable importance (CVI), a metric that controls for the confounding effects of other variables and effectively mitigates the impact of multicollinearity, using the “vip” package (Greenwell and Boehmke 2020). Through this calculation procedure, we employed the permutation approach for CVI calculation, which assesses variable importance by quantifying the reduction in prediction accuracy (using RMSE) when the values of a given variable are randomly permuted while all other variables remain unchanged. We set 20 permutations for each variable to minimize random errors and enhance the stability of CVI results. To ensure consistency between the permutation process and BRT model training, we used the “pred_wrapper” function to guarantee that the prediction process adopts the optimal number of decision trees from the final BRT model, thus avoiding deviations associated with inappropriate tree number selection. Subsequent to obtaining CVI scores for all predictors, we manually normalized these scores and converted them into percentages (with the total sum of normalized scores equal to 100), facilitating intuitive comparison of relative importance across different variables. Finally, the normalized CVI percentage values were defined as the RI values for each variable in the BRT model. Although variables whose RI score exceeded the median importance value were classified as “high importance” (Lloyd et al. 2013; Zhang et al. 2021), here we designated the top four variables by RI score as the most important determinants, as the cumulative sum of the RI scores of these top four variables in each group of analyses already exceeds 70%.

#### 2.4.5 Spatial Autocorrelation of BRT Model Residuals

Given the spatial distribution of sampling plots/subplots, we evaluated spatial autocorrelation in BRT model residuals by generating spatial distribution plots of standardized residuals and calculating the Moran's *I* statistic with its corresponding *p*‐value. To this end, we converted our dataset into a spatial feature object with the coordinate reference system set to WGS_1984 (EPSG: 4326) for geographic consistency, while retaining original coordinate variables to maintain data integrity (Bivand et al. 2013). To support Moran's *I* calculation, we constructed a row‐standardized spatial weights matrix by identifying the 5 nearest neighbors for each valid sampling plot/subplot and converting these into the weight matrix—this approach eliminates bias from uneven sample distribution—and verified the matrix's successful construction afterward (Dormann et al. 2007; Moran 1950). We subsequently calculated BRT model residuals using the optimal number of decision trees from the final model: raw residuals were computed as the difference between observed and predicted values, then standardized to mitigate the impact of data scale variations. Lastly, a two‐tailed Moran's *I* test was conducted on the standardized residuals to detect both positive and negative spatial autocorrelation, with provisions to address potential zero‐neighbor issues. And no significant spatial autocorrelation was detected in any of the analyses (Figure S10).

#### 2.4.6 RI and *R* ^2^ Versus Scale Regression

To investigate how the predictor variables of elevational richness patterns change with spatial scale, we applied linear regression models to regress both the RI score for each predictor variable and the *R* ^2^ of each specific BRT model against the logarithm of subplot size. We performed all analyses using R 4.3.3 (R Development Core Team 2024).

## 3 Results

### 3.1 Variations of *c* ‐ and *z* ‐Values of SARs With Elevation

**FIGURE 2:**
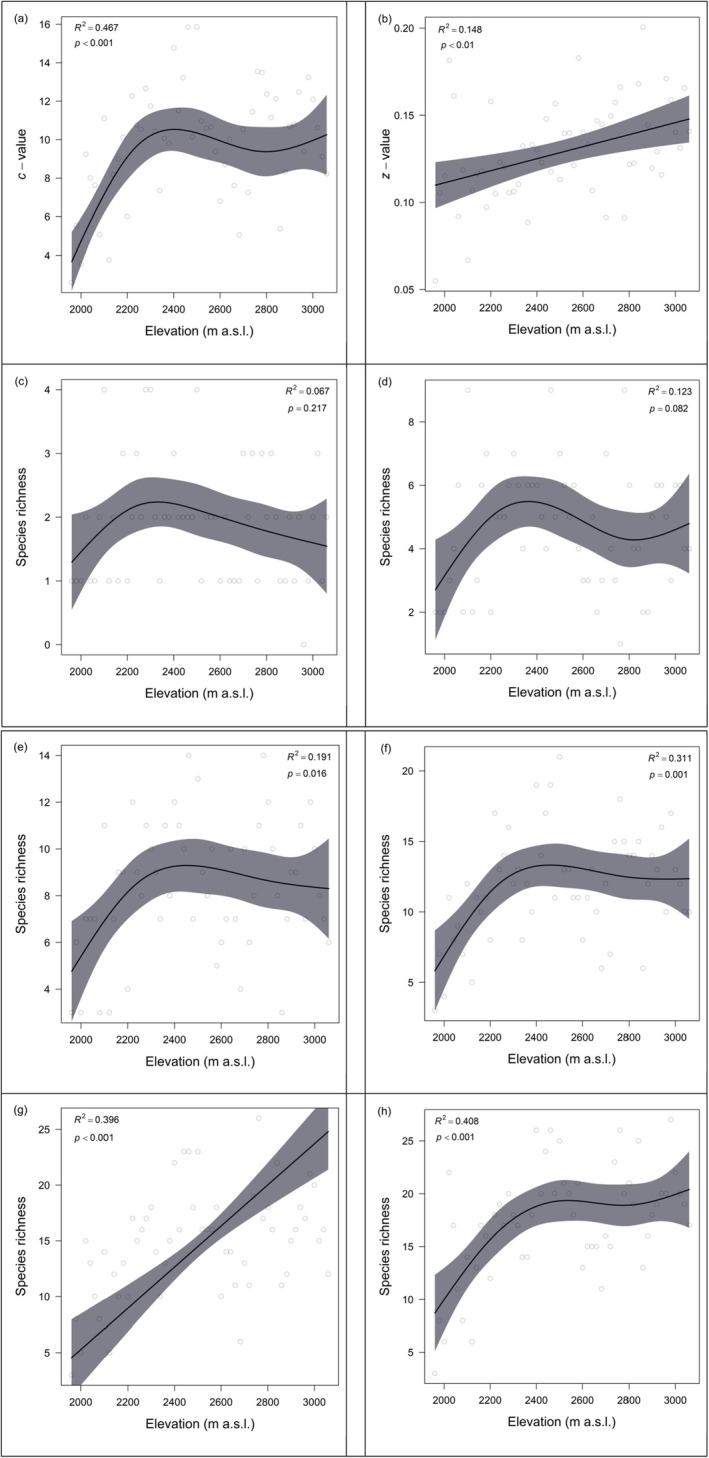
Variations of (a) *c*‐value, (b) *z*‐value, and species richness at different spatial scales: (c) 0.0001 m^2^; (d) 0.01 m^2^; (e) 0.25 m^2^; (f) 1 m^2^; (g) 25 m^2^; and (h) 100 m^2^ along elevation in Mt Wutai. Hollow circles represent *c*‐value, *z*‐value, and species richness respectively, and the black line is the predicted mean derived from GAMs. Shaded areas show the 95% confidence interval of the prediction. The *R* ^2^ and *p*‐values of the smooth effect of elevation were also provided.

A total of 67 moss species, belonging to 45 genera under 16 families, were identified from 56 sampling plots along the elevational gradient on Mt Wutai (Table S1). From fine to broad spatial scales, species richness–plot number rarefaction curves rise gradually and approach an asymptote, indicating that sampling is sufficient to capture the majority of regional species diversity (Figure 1b). Our GAMs revealed a positively skewed hump‐shaped pattern in *c*‐values of SARs along the elevational gradient, with a peak occurring around 2400 m a.s.l. (Figure 2a). Specifically, below 2400 m a.s.l., *c*‐values increased rapidly with rising elevation; above 2400 m a.s.l., they decreased gradually as the increase of elevation. In contrast, *z*‐values of SARs demonstrated a positive correlation with elevation, showing a monotonic increase across the gradient with no discernible peak formation (Figure 2b). There was no significant difference in *c*‐values across the four environments; however, *z*‐values in alpine rocky meadow and forest were significantly higher than those in grassland (Figure S2, Appendix S1).

### 3.2 Determinants of *c* ‐ and *z* ‐Values

**FIGURE 3:**
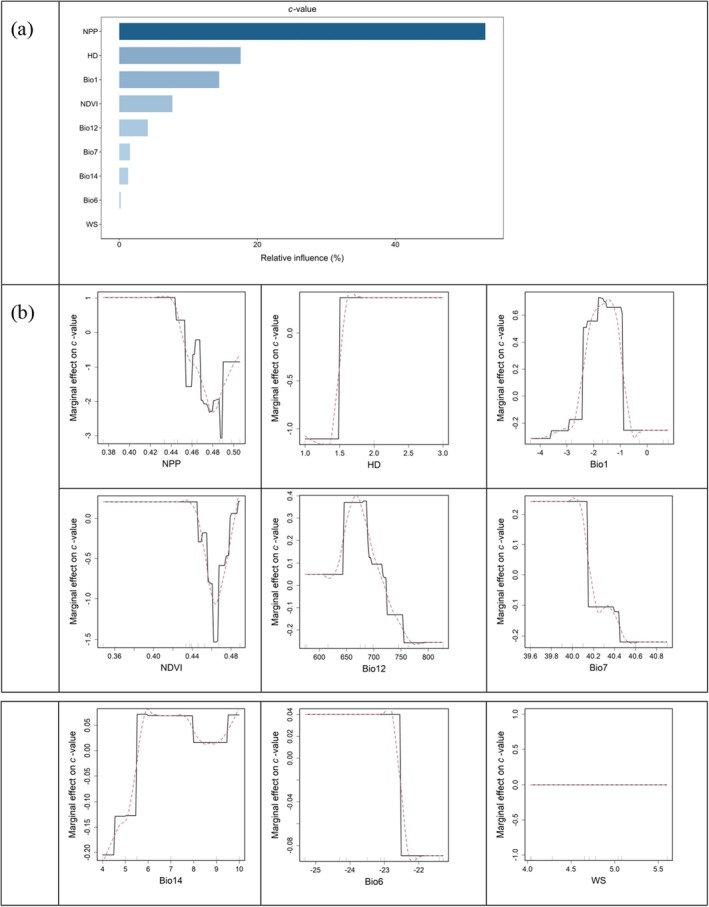
Relative influence (a) and partial dependence plots (b) of individual variables on the variation of *c*‐values. In panel (b), fitted functions are centered by subtracting their mean and plotted on a common scale (black lines), with smoothed lines in red. The deciles of the distribution of the predictor variables are indicated by tick marks. Bio1, mean annual temperature; Bio6, minimum temperature of the coldest month; Bio7, annual temperature range; Bio12, annual precipitation; Bio14, precipitation of the driest month; HD, habitat diversity; NDVI, the normalized difference vegetation index; NPP, net primary productivity; WS, wind speed.

NPP was the most influential predictor for the variation of *c*‐values with a RI of 53.0%, followed by HD (17.6%), Bio1 (14.5%), and NDVI (7.7%). From the smoothed lines in red in the partial dependence plots, as NPP increased, *c*‐values first fell, then rose, and finally flattened once NPP exceeded 0.48 kg C/m^2^/year. As Bio1 increased, *c*‐values first rose, then fell, and finally flattened once Bio1 exceeded −0.5°C. As NDVI increased, *c*‐values remained constant initially; once NDVI exceeded 0.44, *c*‐values decreased, and after NDVI surpassed 0.46, they started to increase again. And HD exerted positive effects on *c*‐values (Figure 3).

**FIGURE 4:**
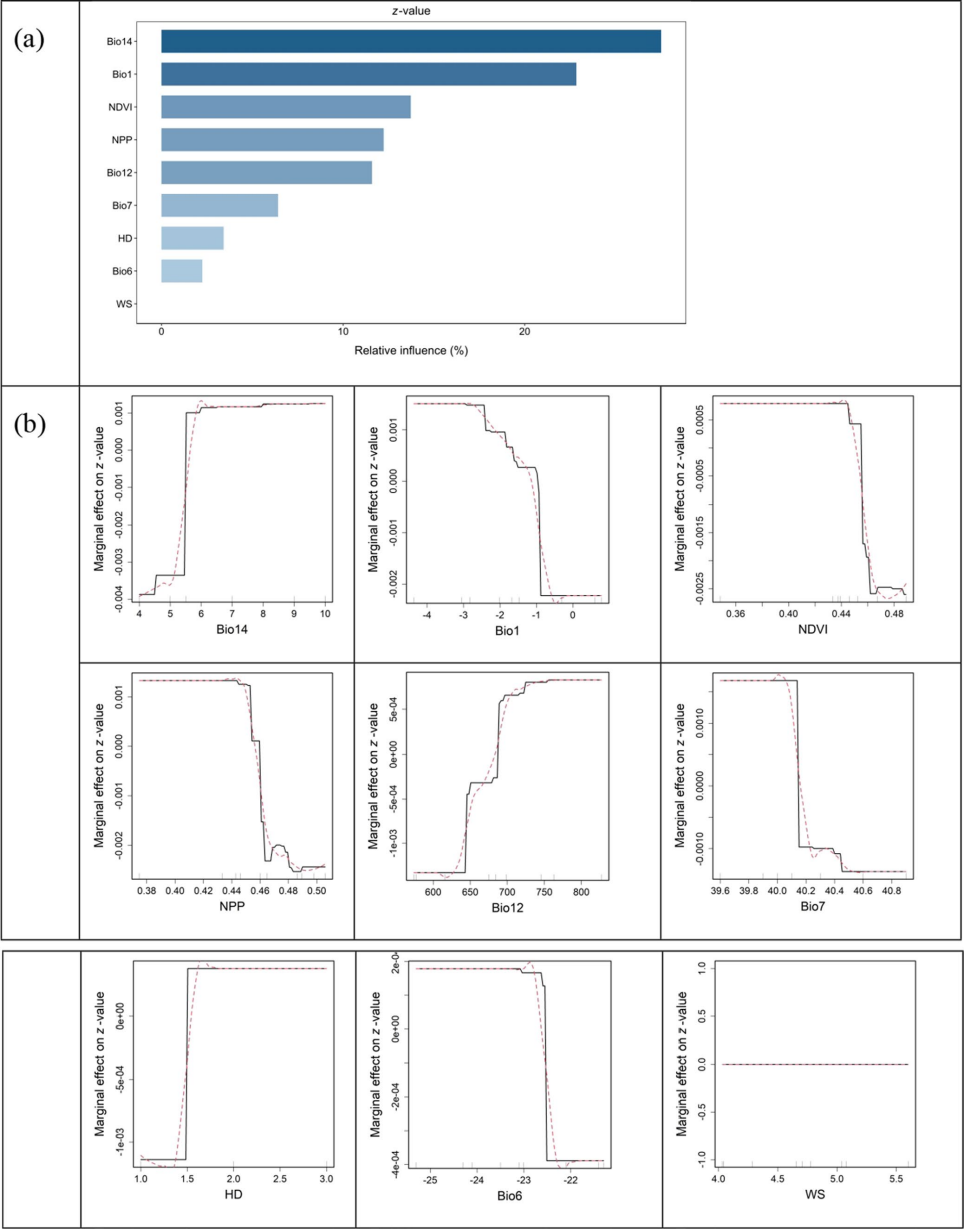
Relative influence (a) and partial dependence plots (b) of individual variables on the variation of *z*‐values. In panel (b), fitted functions are centered by subtracting their mean and plotted on a common scale (black lines), with smoothed lines in red. The deciles of the distribution of the predictor variables are indicated by tick marks. Bio1, mean annual temperature; Bio6, minimum temperature of the coldest month; Bio7, annual temperature range; Bio12, annual precipitation; Bio14, precipitation of the driest month; HD, habitat diversity; NDVI, the normalized difference vegetation index; NPP, net primary productivity; WS, wind speed.

Bio14 was the most influential predictor for the variation of *z*‐values with a RI of 27.5%, followed by Bio1 (22.8%), NDVI (13.7%), and NPP (12.2%). From the smoothed lines in red in the partial dependence plots, Bio1, NPP, and NDVI exerted negative effects on *z*‐values whereas Bio14 exerted positive effects on *z*‐values (Figure 4).

### 3.3 Variations of Elevational Richness Patterns With Spatial Scale

Elevational richness patterns were examined under six different spatial scales (i.e., 0.0001, 0.01, 0.25, 1, 25, and 100 m^2^). As spatial scale increased, *R* ^2^ of GAMs gradually rose and *p*‐values became increasingly significant (Table 1), indicating that the predictability of the elevational richness pattern grew more robust. At the spatial scales of 0.0001, 0.01, 0.25, and 1 m^2^, similarly with the variation of *c*‐values, our GAMs revealed a positively skewed hump‐shaped pattern in species richness along the elevational gradient, with a peak occurring around 2400 m a.s.l., below 2400 m a.s.l., species richness increased rapidly with rising elevation; above 2400 m a.s.l., they decreased gradually with increasing elevation. However, at the spatial scales of 25 m^2^, species richness was positively correlated with elevation after controlling for the effects of spatial autocorrelation (Table S12), whereas at the spatial scales of 100 m^2^, species richness increased with elevation, with the rate of increase gradually slowing down, and the mid‐elevation peak became less distinct (Figure 2; Table 1).

**TABLE 1:** Summary of variations in elevational richness patterns, including peak location and strength; GAM fit, including *R* ^2^ and the *p*‐value of the smooth effect of elevation (*p*‐s(elevation)); and BRT performance, including root mean square error (RMSE), mean absolute error (MAE), and *R* ^2^ across scales.

Scale (m^2^)	Elevational richness pattern		GAM fit		BRT performance		
	Peak location (m a.s.l.)	Strength	*R* ^2^	*p*‐s(elevation)	RMSE (%)	MAE (%)	*R* ^2^
0.0001	2351.8	Weak	0.067	0.217	0.746	0.602	0.242
0.01	2380.6	Weak	0.123	0.082	1.894	1.579	0.030
0.25	2411.7	Strong	0.191	0.016	2.390	1.934	0.270
1	2429.1	Strong	0.311	0.001	1.992	1.644	0.690
25	Non‐hump‐shaped	—	0.396	< 0.001	2.180	1.677	0.794
100	Non‐hump‐shaped	—	0.408	< 0.001	2.424	1.709	0.785

### 3.4 Spatial Scale Effects on the Determinants of Elevational Richness Patterns

At the spatial scale of 0.0001 m^2^, MDE was the most influential predictor for the variation of species richness with a RI of 60.7%, followed by HD (13.2%), Bio14 (12.4%), and NPP (7.6%). From the smoothed lines in red in the partial dependence plots, as MDE increased, species richness first rose, then fell. Bio14 and NPP exerted negative effects whereas HD exerted positive effects on species richness (Figure S8). At the spatial scale of 0.01 m^2^, MDE was the most influential predictor for the variation of species richness with a RI of 54.8%, followed by NPP (17.3%), Bio12 (8.9%), and Bio1 (8.5%). Bio12 and NPP exerted negative effects whereas MDE and Bio1 exerted positive effects on species richness (Figure S8). At the spatial scale of 0.25 m^2^, MDE was the most influential predictor for the variation of species richness with a RI of 61.5%, followed by NPP (31.6%), NDVI (3.9%), and HD (2.1%). As MDE increased, species richness first rose, then fell. NDVI and NPP exerted negative effects whereas HD exerted positive effects on species richness (Figure S8). At the spatial scale of 1 m^2^, MDE was the most influential predictor for the variation of species richness with a RI of 47.4%, followed by NPP (30.4%), HD (13.4%), and NDVI (5.7%). As MDE increased, species richness first rose, then fell. NDVI and NPP exerted negative effects whereas HD exerted positive effects on species richness (Figure S8). At the spatial scale of 25 m^2^, MDE was the most influential predictor for the variation of species richness with a RI of 42.8%, followed by NPP (26.7%), HD (11.6%), and NDVI (9.4%). As MDE increased, species richness first rose, then fell. Bio7 and NPP exerted negative effects whereas HD exerted positive effects on species richness (Figure S8). At the spatial scale of 100 m^2^, MDE was the most influential predictor for the variation of species richness with a RI of 31.4%, followed by NPP (29.0%), HD (15.1%), and Bio1 (9.2%). As MDE and Bio1 increased, species richness first rose, then fell; and as NPP increased, species richness first fell, then rose whereas HD exerted positive effects on species richness (Figure S8).

**FIGURE 5:**
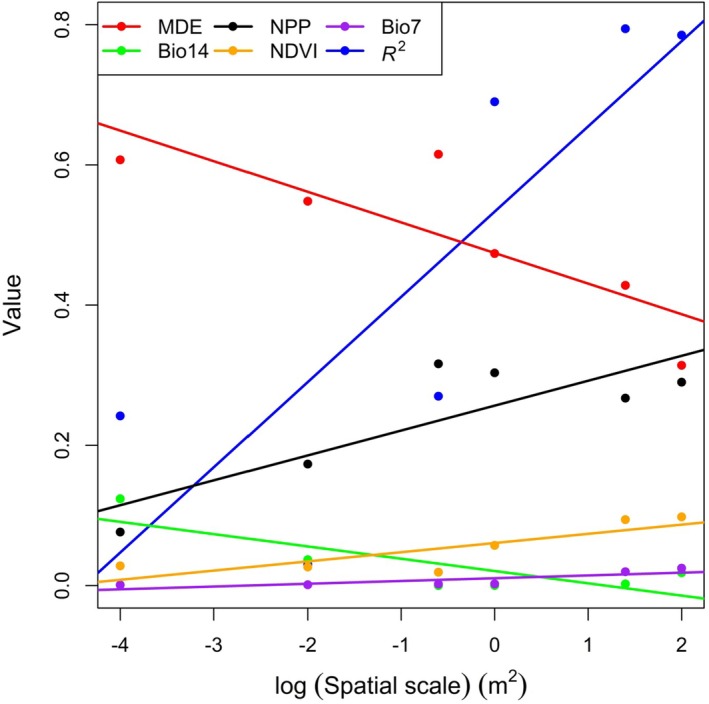
Significant variations of the predictive performance (*R* ^2^) and the driving factors of elevational diversity patterns acquired from BRT models with spatial scale. Bio7, annual temperature range; Bio14, precipitation of the driest month; MDE, the mid‐domain effect; NDVI, the normalized difference vegetation index; NPP, net primary productivity.

According to the results of linear regression models regressing both the RI score for each predictor variable and the *R* ^2^ of each specific BRT model against the logarithm of subplot size, *R* ^2^ of BRTs, Bio7, NPP, and NDVI had a significantly positive relationship with spatial scales, whereas MDE and Bio14 had a significantly negative relationship with spatial scales (*p* < 0.05; Figure 5; Table S11). Other predictor variables, such as HD, Bio1, Bio6, Bio12, and WS, had a nonsignificant relationship with spatial scales (*p* > 0.05; Table S11).

## 4 Discussion

In this study, we elucidated the effects of elevation on moss SARs and the influence of spatial scale on elevational patterns of moss species richness in Mt Wutai. To date, studies on these two research themes in plants have been largely restricted to vascular plants, whereas equivalent investigations remain scarce for non‐vascular taxa such as mosses. Notably, our study represents the first attempt to examine how the relative contributions of individual predictors in shaping elevational richness patterns vary across spatial scales.

### 4.1 Variations and Determinants of *c* ‐Values

Since *c*‐values denote the expected mean number of species per unit area (Moradi et al. 2020), the elevational pattern of *c*‐values (Figure 2a) is notably analogous to that of species richness at fine spatial scales, rather than coarser scales; this consistency arises because both *c*‐values and fine‐grain species richness exhibit a distinct mid‐elevation richness peak, whereas this peak diminishes at larger plot sizes. We found that non‐linear responses of variations of *c*‐values to environmental variables were prevalent, with some variables even exhibiting non‐monotonic relationships (Figure 3), which is consistent with our expectations. This suggests that linear or monotonic frameworks may be inadequate to capture the complex relationships in our data. Fortunately, more sophisticated nonparametric and machine learning methods developed in recent years have offered novel approaches to exploring complex nonlinear processes in ecological studies (Wüest et al. 2020). The positively skewed hump‐shaped pattern of *c*‐values along the elevational gradient is mainly affected by energy availability, environmental factors, and habitat complexity.

The reason why *c*‐values first decrease then increase as NPP and NDVI rise along elevational gradients may arise from the unique ecological traits of mosses and how NPP and NDVI mediate resource availability, habitat filtering, and interspecific interactions across environmental gradients. Mosses are non‐vascular, poikilohydric organisms (He et al. 2016). Their poikilohydric nature does not enable them to withstand prolonged dehydration under conditions of unpredictable water availability (Proctor and Tuba 2002). Their survival is more sensitive to microhabitat conditions (e.g., humidity, substrate availability) than vascular plants, so NPP and NDVI gradients may indirectly shape moss *c*‐values by altering these microconditions. When NPP and NDVI are low, vascular plant cover is sparse (low NDVI), and overall productivity (NPP) is limited by harsh conditions; however, mosses thrive here because: mosses' poikilohydry enables certain moss taxa to tolerate periodic desiccation (low‐NPP and low‐moisture environments) better than small vascular plants; moreover, sparse vascular cover results in little overlap in resource use and mosses avoid competition with vascular plants. When NPP and NDVI are moderate, grasses and herbs grow more vigorously, and dense vascular canopies will shade the understory. Although mosses are shade‐tolerant, extreme shading reduces their photosynthetic capacity (Marschall and Proctor 2004). Furthermore, vascular plants absorb water via roots, reducing both underground and surface moisture; the resulting dry microhabitats can exclude moisture‐dependent moss species and lower moss richness per unit area. Meanwhile, competition with vascular plants, which is likely associated with higher NPP values, also plays a fundamental role in reducing moss richness. When NPP and NDVI reach high levels, multi‐layered forests with trees, shrubs, and epiphytes become dominant. Tree trunks, branches, and leaves provide new surfaces for epiphytic mosses. Dense canopies reduce evaporation and moderate extreme temperatures (Breshears et al. 1998), creating moist and cooling conditions suitable for moss species (Waite and Sack 2010).

The reason *c*‐values first increase and then decrease as Bio1 rises along the elevational gradient lies in the fact that species richness reaches its peak under the most suitable climatic conditions. Additionally, the monotonic positive correlation between *c*‐values and HD supports the habitat complexity hypothesis (Brown 2001; Wu et al. 2013). Higher habitat complexity creates a greater variety of microhabitats and niche spaces, which in turn allows more moss species to coexist within the same unit area, leading to elevated *c*‐values.

The monotonic decline in *c*‐values with elevation reported by Moradi et al. (2020) stands in stark contrast to our positively skewed hump‐shaped *c*‐value pattern, and this discrepancy may arise primarily from study organism traits and regional environmental constraints. Moradi et al. (2020) focused on vascular plants in the dry, continental Alborz Mountains (Iran), where *c*‐values decreased steadily with rising elevation, driven by falling temperature and soil nitrogen availability, alongside increasing rock cover. Vascular plants are highly dependent on soil nutrients and thermal energy, making them extremely sensitive to these resource limitations at high elevations, which directly suppresses unit‐area species richness. In contrast, our study targets mosses on Mt Wutai, a humid temperate mountain system with distinct ecological constraints. Mosses lack true roots, relying instead on atmospheric moisture and microtopography rather than soil nitrogen, and exhibit greater tolerance to moderate abiotic stress than vascular plants. The humid mid‐elevations of Mt Wutai provide optimal moisture conditions and high microhabitat heterogeneity, supporting a peak in *c*‐values; low elevations face intensified biotic competition, while high elevations experience extreme abiotic pressure, both leading to reduced *c*‐values and forming the skewed hump‐shaped trend. Additionally, rock cover on Mt Wutai acts as a moisture‐retaining microhabitat for mosses, rather than a limiting factor, further decoupling our *c*‐value pattern from the monotonic decline observed by Moradi et al. (2020).

### 4.2 Variations and Determinants of *z* ‐Values

In studies of SARs, *z*‐values typically range between 0.20 and 0.35 (Panitsa et al. 2006; Santos et al. 2010). Our findings, however, revealed notably lower *z*‐values (0.05–0.20; Figure 2b) for moss communities on Mt Wutai, indicating comparatively slower rates of species accumulation. *z*‐values displayed a positive correlation with elevation, showing a monotonic increase along the elevational gradient with no discernible peak. This pattern indicates that bryophyte species accumulate more rapidly with increasing sampling area at higher elevations, which also reflects faster species turnover rates across space. This result may be due to two reasons: first, owing to their poikilohydric strategy, mosses exhibit strong sensitivity to environmental fluctuations. Air temperature and humidity are usually the main factors determining the richness of moss species along the elevation gradient (Fu et al. 2023; Gao et al. 2021). Generally, as elevation increases, temperature decreases and humidity changes, creating a harsher microclimate. In this case, the species composition varies more significantly with the increase in area, resulting in a higher *z*‐value. Second, as elevation increases, both terrain complexity and biotic interactions between vascular plants and mosses intensify. Furthermore, we found that lithophytic habitats characterized by large rock and scree substrates were widespread at higher elevations, but nearly absent at lower elevations. The presence of these rocky microhabitats created additional niche space and contributed to a more heterogeneous environment at higher elevations. When the area increases, more different microhabitats can be covered, and the number of species will increase significantly, thus increasing the *z*‐value.

The increase in *z*‐values of moss species with elevation and their negative correlation with Bio1 along the elevational gradient can be attributed to the fact that, as we discussed above, the diverse topographies at higher elevations (lower Bio1), such as steep slopes, cliffs, and rocky outcrops, create a variety of microhabitats, leading to an increase in the *z*‐values. While warmer temperatures at lower elevations (higher Bio1) tend to create more homogeneous habitats, thus reducing the *z*‐values. Besides, owing to mosses' poikilohydric adaptations and cold tolerance, traits that confer competitive advantages under thermal constraints (Takezawa 2018), reduced temperatures at higher elevations are likely to suppress vascular plant growth via diminished photosynthetic efficiency or frost damage (Körner 1998). This, in turn, alleviates competition, enabling mosses to rapidly colonize vacated niches. The positive correlation observed between *z*‐values and Bio14 along the elevational gradient is a result of the fact that, as elevation increases, Bio14 also rises, driven by orographic precipitation or fog. Together with the abundant rocky microhabitats at higher elevations, this forms moist and buffered, relatively stable microenvironmental conditions that facilitate habitat diversification and species specialization, ultimately leading to higher *z*‐values. Moreover, the negative correlation observed between *z*‐values and NDVI or NPP along the elevational gradient can be ascribed to the fact that dense vascular vegetation (high NDVI and NPP) outcompetes mosses for critical resources, including light, space, and moisture, thereby lowering *z*‐values. In contrast, sparse vascular vegetation (low NDVI and NPP) may benefit mosses by providing microhabitats, which in turn enhances *z*‐values.

Our finding that *z*‐values increase monotonically with elevation is consistent with Baumann et al. (2016), probably driven by shared sampling design and high‐elevation ecological mechanisms. Both studies employed nested plot sampling across fine spatial scales, and focused on temperate montane/alpine vegetation where environmental filtering intensifies with rising elevation. At higher elevations, harsher climates reduce species colonization ability, fragment microhabitats, and increase spatial turnover of species—small plots capture only a subset of stress‐tolerant species, while larger plots integrate more microhabitats and rare species, steepening the species–area slope. Lower elevations have more connected habitats and wider species dispersal, resulting in lower *z*‐values. This elevational enhancement of *z*‐values via strengthened environmental filtering and reduced spatial connectivity is a shared mechanism between our moss study and Baumann et al.'s (2016) alpine grassland study.

The opposing *z*‐value trends between our work and Moradi et al. (2020) further reflect distinct dominant drivers and organismal responses across study systems. Moradi et al. (2020) reported *z*‐values decreasing with elevation in arid montane vascular plants, as rising elevation reduced temperature and soil nutrients—key limiting factors that homogenize species composition across spatial scales, weakening the species‐area relationship and lowering *z*‐values. For their vascular plant communities, high elevations act as a severe environmental filter that restricts species to a narrow, stress‐tolerant pool, reducing spatial turnover and flattening the species–area slope. In our Mt Wutai moss system, high elevations increase microhabitat heterogeneity (e.g., shaded crevices, moist substrates) rather than homogenizing communities; mosses exhibit high habitat specificity, and small plots at high elevations fail to capture most microhabitat specialists. Larger plots are required to encompass these diverse microhabitat‐associated species, leading to a steeper *z*‐value trend with elevation. Moreover, the humid climate of Mt Wutai prevents the extreme abiotic homogenization observed in the arid Alborz Mountains, preserving high spatial turnover and driving a monotonic increase in *z*‐values, directly contradicting the declining trend documented by Moradi et al. (2020).

### 4.3 Variations of Elevational Richness Patterns With Spatial Scale

The shift in moss species richness patterns along the elevational gradient, from a positively skewed hump shape at smaller spatial scales (0.0001, 0.01, 0.25, 1 m^2^) to a decelerating increasing trend with a less distinct mid‐elevation peak at larger scales (100 m^2^; Figure 2; Table 1), may be attributed to the following causes. First, small subplots capture only tiny subsets of the local environment. Along elevations, mid‐elevations typically have the highest microhabitat diversity (Gao et al. 2021) that supports both low‐elevation generalists (tolerant of warmer conditions) and high‐elevation specialists (adapted to milder cold stress). This mix creates a richness peak at mid‐elevations. Second, at small scales, high elevations appear species‐poor because cold stress restricts mosses to scattered microrefugia, however, at large scales, subplots cover more of these isolated microrefugia, each contributing new stress‐tolerant species. As large sampling plots at high elevations capture more microrefugia and their specialized moss species. As a result, species richness at high elevations becomes similar to or even higher than that at mid elevations, and the distinct mid‐elevation richness peak therefore disappears. Third, while high‐elevation richness increases with scale, the rate slows because cold stress and short growing seasons still limit the total number of cold‐tolerant moss species (i.e., the regional high‐elevation species pool is smaller than the mid/low‐elevation pool). Moreover, high‐elevation sites are geographically isolated because they occur as discrete, island‐like habitat patches separated by extensive areas of lower‐elevation environments that are unsuitable for most specialist mosses. This strong dispersal barrier prevents new species from colonizing rapidly, causing the rate of richness increase to slow gradually (MacArthur and Wilson 1967). Interestingly, our findings are contrary to those of Bhatta et al. (2018), who found that at all spatial scales, the species richness of herbaceous plants consistently exhibits a hump‐shaped elevational pattern. At the finest scale (1 m^2^), the species richness of woody plants does not show a clear elevational pattern. At larger scales, by contrast, the species richness of woody plants displays a monotonically decreasing elevational pattern. Such conflicting findings underscore that, owing to their unique features, mosses likely exhibit distinct biogeographical patterns of their own (Fu et al. 2023). Even so, our observations remain broadly consistent with the general framework of scale dependence documented in previous studies (Rahbek 2005; McCain and Grytnes 2010; Dáttilo et al. 2023), confirming that elevational richness patterns shift markedly in curve shape, peak magnitude, and peak position with increasing spatial grain. In accordance with Graham et al. (2014) and Montes et al. (2021), larger sampling scales smoothed local ecological variation, reduced stochasticity, weakened the mid‐elevation peak, and produced a relatively gradual, decelerating increase in richness. Specifically, we detected a distinct skewed humped pattern at fine spatial scales, reflecting high local variability and pronounced mid‐elevation peaks at local extents (McCain and Grytnes 2010), whereas at coarse scales the hump diminished into an indistinctly peaking trend, consistent with diluted site‐specific effects and stronger control by regional species pools (Rahbek 2005). Although our results follow the well‐established scale‐dependent paradigm, the positively skewed hump observed for mosses at small scales differs from the symmetric or left‐skewed patterns typical of vascular plants, highlighting a trait‐driven distinction inherent to bryophyte assemblages.

### 4.4 Variations of the Driving Factors of Elevational Richness Patterns With Spatial Scale

The predictive performance (*R* ^2^) of BRT models for species richness exhibited a positive correlation with spatial scale (Figure 5), demonstrating that the strength of the relationship between elevational richness patterns and environmental predictors increases with sampling scale. Small plots primarily reflect fine‐scale microhabitat conditions that are highly variable and weakly coupled to broad elevational gradients. By contrast, larger sampling units incorporate a wider range of microhabitats and environmental conditions, reducing noise from local stochasticity and better representing the systematic environmental shifts driven by elevation. Consequently, the association between elevation and species richness becomes clearer and statistically stronger at larger scales. This observation is consistent with the environmental heterogeneity and energy‐availability hypotheses, which posit that greater environmental diversity in larger areas enhances species coexistence and strengthens species–environment relationships (Onditi et al. 2024; MacArthur and MacArthur 1961; Stein et al. 2014).

As the spatial scale increases, the RI score of the MDE declines significantly when explaining variations in species richness (Figure 5). This aligns with how elevational richness patterns change with spatial scale: the distinct mid‐elevation peak becomes erased at larger spatial scales. It is probably because MDE is a null model and it relies on geometric constraints (random packing of species' elevational ranges within mountain boundaries) to explain richness patterns (Colwell and Hurtt 1994; Colwell and Lees 2000), but larger sampling plots shift the focus toward ecological processes; therefore, undermining MDE's influence. For example, larger sampling plots will include diverse microhabitats and each microhabitat supports distinct species that may not be limited by elevational range alone (Tubay et al. 2015). Their presence depends on local conditions, not just whether the elevation falls within their range. Moreover, larger sampling plots support more species and more complex interactions (Arrhenius 1921). These interactions redefine richness patterns: species presence is no longer just a function of elevational range overlap (MDE) but of biotic compatibility. MDE, which cannot account for this, becomes less important.

It should be emphasized that all WorldClim bioclimatic variables used in this study represent macroclimatic conditions and thus do not capture fine‐scale microclimatic variation or local biotic interactions. The reason why the relative importance of these variables responds differently to increasing spatial scale lies in their ecological nature: Bio14 (precipitation of the driest month) represents extreme drought stress, which can be strongly buffered by microhabitats (e.g., seeps, shaded rocky crevices, and moist substrates) within large plots (Maclean et al. 2015). As plot size increases, the decoupling between local moisture availability and macroclimatic dryness becomes more evident, leading to a marked decline in the relative importance of Bio14 (Figure 5). Bio7 (annual temperature range) reflects large‐scale climatic seasonality and temperature fluctuation. Although microhabitats can moderate short‐term, local temperature extremes, they do not alter the regional annual temperature range. Larger plots therefore better capture this broad, consistent climatic gradient, leading to an increase in the relative importance of Bio7 with spatial scale (Figure 5). In contrast, Bio1 (mean annual temperature), Bio6 (minimum temperature of the coldest month), and Bio12 (annual precipitation) represent average climatic conditions that change consistently and predictably along the elevational gradient. Because microhabitats exert relatively weak effects on these broad, average climatic trends, their relative importance remains stable and does not differ significantly across sampling scales.

The RI score of NDVI and NPP rise as sampling plot size increases when explaining elevational richness patterns (Figure 5). This is likely because for small plots, the number of species present is often limited by random colonization or microhabitat constraints, rather than the larger‐scale energy availability measured by NDVI or NPP (Chase and Knight 2013). In this case, NDVI or NPP has little explanatory power. However, for larger plots, the species assemblage becomes more representative of the elevational zone's typical community, which is directly tied to NDVI and NPP (Rafique et al. 2016; Whittaker et al. 2001; Zhang et al. 2009). Therefore, as plot size increases, the scale of measurement matches the scale of NDVI or NPP's ecological effect, amplifying their ability to explain elevational richness patterns. Additionally, at small scales, other factors (e.g., microclimate, soil pH, or competition from a single dominant species) often override the influence of NDVI or NPP, masking their relationship with species richness. Whereas larger plots are more likely to include habitat heterogeneity that supports species coexistence, and this heterogeneity is itself correlated with NDVI and NPP, strengthening their explanatory power for species richness.

### 4.5 Limitations and Prospects

Notably, this study relied on coarse (≈1 km^2^) climatic and remote‐sensing datasets for environmental covariates. Despite the clear elevational gradient, this low resolution generated limited unique values across the 56 sampling plots, indicating that existing predictors may fail to fully capture the fine‐scale environmental heterogeneity governing local species richness. Although BRT and other statistical models were applied to analyze SAR parameters and richness–environment relationships, statistical corrections alone cannot fully eliminate the spatial grain mismatch between predictor variables and field sampling design. Therefore, the explanatory power of these environmental covariates warrants cautious interpretation. Rather than pursuing strong causal inference, this study emphasizes the observational and ecological value of field data, documenting SAR parameters and species richness patterns along a continuous elevational gradient in an understudied region. Caution is needed when extrapolating these findings, and future research is recommended to adopt finer‐resolution environmental data to better clarify the local‐scale drivers of SAR parameters and species richness.

Future studies targeting fine‐scale drivers of SAR parameters and species richness should prioritize high‐resolution environmental data consistent with field sampling grain. In particular, in situ measurements of microclimate, soil properties, topography, and vegetation structure can detect fine‐scale heterogeneity that coarse remote‐sensing or interpolated climate layers cannot resolve. These detailed, variable covariates will alleviate the limited unique‐value problem across plots and enhance the reliability and interpretability of regression‐tree and other statistical models. Furthermore, future work should adopt multi‐scale sampling designs that align predictor resolution with the ecological processes under investigation. Integrating field‐measured microenvironmental variables with progressively finer remote‐sensing data will strengthen causal inference and reduce spatial grain mismatches. These improvements will enhance the scientific rigor of richness–environment association studies and support more robust tests of ecological hypotheses.

## 5 Conclusions

By exploring how SARs vary with elevation and how elevational diversity patterns change across spatial scales, our study provides targeted insights for the long‐term conservation of moss diversity. The scale‐dependent strengthening of elevational richness–environment associations, coupled with dynamic shifts in the relative importance of environmental drivers across spatial scales, supports an integrated “large‐scale guiding, small‐scale refining” conservation framework. Specifically, broad‐scale environmental drivers—most notably the annual temperature range (Bio7), which exhibits increasing predictive power with larger spatial scales—should serve as the foundation for delineating core conservation zones along the elevational gradient. In contrast, fine‐scale management strategies should prioritize the protection of microrefugia that buffer drought stress (linked to Bio14, whose predictive importance declines with increasing plot size) and maintain habitat connectivity to effectively mitigate dispersal limitation in high‐elevation, scale‐sensitive moss communities. At larger spatial scales, conservation efforts must also focus on safeguarding vegetation structure and productivity (reflected by NDVI and NPP) to sustain the environmental conditions that underpin moss diversity. By aligning conservation actions with the scale‐specific environmental drivers identified in this study, this integrated approach enhances the effectiveness, robustness, and targeted nature of moss diversity conservation, ensuring that management strategies accurately reflect the ecological mechanisms shaping moss elevational richness patterns across spatial scales.

## Author Contributions

**Haozhe Wang:** formal analysis (equal), investigation (equal), writing – original draft (equal). **Fenghua Wang:** investigation (equal), writing – original draft (equal). **Yu Zhao:** formal analysis (equal), investigation (equal). **Chenglong Li:** investigation (equal). **Xiaowei Ma:** investigation (equal). **Xiaopan Wang:** investigation (equal). **Lina Zhang:** formal analysis (equal). **De Gao:** conceptualization (lead), formal analysis (equal), investigation (equal), methodology (lead), supervision (lead), writing – review and editing (equal).

## Funding

This work was supported by National Natural Science Foundation of China (32160315, 42271045) and Hebei Normal University (L2024J05).

## Conflicts of Interest

The authors declare no conflicts of interest.

## Supporting information


**Appendix S1:** Results of moss species identification, influence of elevation on the species–area relationship, GAMs, BRT models, and spatial scale effects on elevational richness pattern, including Tables S1–S12 and Figures S1–S10.

**Appendix S2:** The detailed pseudo R code utilized for the statistical analyses in this study.

**Appendix S3:** The data used in this article, including Tables S13–S19.
**Table S13:** Site_Environment_Data.
**Table S14:** Site_Species_Occurrence (0.01 m).
**Table S15:** Site_Species_Occurrence (0.1 m).
**Table S16:** Site_Species_Occurrence (0.5 m).
**Table S17:** Site_Species_Occurrence (1 m).
**Table S18:** Site_Species_Occurrence (5 m).
**Table S19:** Site_Species_Occurrence (10 m).

## Data Availability

The data are provided in the Supporting Information for this article.
