# Supplementary material for: Unraveling the Influence of Elevation on Moss Species–Area Relationships and the Effect of Spatial Scale on Elevational Richness Patterns in Mt Wutai With a Nested‐Plot Sampling Design
Source: Ecol Evol. 2026 Apr 10;16(4):e73473. doi: 10.1002/ece3.73473 (PMC13068500; doi:10.1002/ece3.73473)
Supplement: Supplementary file 1 — Appendix S1: Results of moss species identification, influence of elevation on the species–area relationship, GAMs, BRT models, and spatial scale effects on elevational richness pattern, including Tables S1–S12 and Figures S1–S10. [file ECE3-16-e73473-s001.docx]

**Unravelling the influence of elevation on moss species–area relationships and the effect of spatial scale on elevational richness patterns in Mt Wutai with a nested-plot sampling design**

Haozhe Wang, Fenghua Wang, Yu Zhao, Chenglong Li, Xiaowei Ma, Xiaopan Wang, Lina Zhang, De Gao*

**SUPPORTING INFORMATION**

**Appendix S1** Results of moss species identification, influence of elevation on the species–area relationship, GAMs, BRT models, and spatial scale effects on elevational richness pattern, including Table S1–S12 and Figure S1–S10.

**Table S1.** Checklist of moss species identified in the 56 sampling plots.

**Table S2.** Meta-parameter values used in tuning BRT models.

**Table S3.** Possible combinations of the three meta-parameters for fitted 360 BRT models for *c*-value.

**Table S4.** Possible combinations of the three meta-parameters for fitted 360 BRT models for *z*-value.

**Table S5.** Possible combinations of the three meta-parameters for fitted 360 BRT models for species richness at 0.0001 m^2^ spatial scale.

**Table S6.** Possible combinations of the three meta-parameters for fitted 360 BRT models for species richness at 0.01 m^2^ spatial scale.

**Table S7.** Possible combinations of the three meta-parameters for fitted 360 BRT models for species richness at 0.25 m^2^ spatial scale.

**Table S8.** Possible combinations of the three meta-parameters for fitted 360 BRT models for species richness at 1 m^2^ spatial scale.

**Table S9.** Possible combinations of the three meta-parameters for fitted 360 BRT models for species richness at 25 m^2^ spatial scale.

**Table S10.** Possible combinations of the three meta-parameters for fitted 360 BRT models for species richness at 100 m^2^ spatial scale.

**Table S11.** Results of linear regression models showing the correlations between each of the items and spatial scales.

**Table S12.** Spatial autocorrelation test in residuals of elevation-only GAMs using Moran’s *I* test with Monte Carlo simulation.

**Figure S1.** Scatterplots of each variable for species richness at 100 m^2^ spatial scale.

**Figure S2.** Comparison of *c*- and *z*-values across four main environments.

**Figure S3.** Relationship between the MDE and species richness under each spatial scale.

**Figure S4.** The important interactive effects between variables in driving *c*-value.

**Figure S5.** The important interactive effects between variables in driving species richness at 0.0001 m^2^ spatial scale.

**Figure S6.** The important interactive effects between variables in driving species richness at 0.01 m^2^ spatial scale.

**Figure S7.** The important interactive effects between variables in driving species richness at 25 m^2^ spatial scale.

**Figure S8.** Relative influence and partial dependence plots of each individual variable on moss species richness variation.

**Figure S9.** Sensitivity analysis due to the spatial scale mismatch between biological observations and environmental predictors.

**Figure S10.** Spatial distribution of standardized BRT residuals with Moran’s *I* statistic and *p*-value.

**Table S1.** Checklist of moss species identified in the 56 sampling plots.

| Family | Genus | Species |
| --- | --- | --- |
| Anomodontaceae | *Anomodon* | *Anomodon giraldii* Müll. Hal. |
| Brachytheciaceae | *Cirriphyllum* | *Cirriphyllum cirrosum*(Schwägr.) Grout |
|  | *Myuroclada* | *Myuroclada maximowiczii*(G. G. Borshch.) Steere & W. B. Schofield |
|  | *Rhynchostegium* | *Rhynchostegium fauriei*Cardot |
| Bryaceae | *Anomobryum* | *Anomobryum filiforme*(Griff.) A. Jaeger |
|  |  | *Anomobryum gemmigerum*Broth. |
|  | *Bryum* | *Bryum arcticum*(R. Br.) Bruch & Schimp. |
|  |  | *Bryum argenteum*Hedw. |
|  |  | *Bryum caespiticium*Hedw. |
|  |  | *Bryum cellulare*Hook. |
|  |  | *Bryum coronatum*Schwägr. |
|  |  | *Bryum funkii*Schwägr. |
|  |  | *Bryum uliginosum*(Brid.) Bruch & Schimp. |
|  | *Plagiobryum* | *Plagiobryum demissum*(Hook.) Lindb. |
|  | *Pohlia* | *Pohlia elongata*Hedw. |
| Dicranaceae | *Atractylocarpus* | *Atractylocarpus alpinus*(Schimp. ex Milde) Lindb. |
|  | *Dicranella* | *Dicranella micro-divariata*(C. Muell.) Par. |
|  | *Dicranoweisia* | *Dicranoweisia crispula*(Hedw.) Lindb. ex Milde |
|  | *Leucoloma* | *Leucoloma okamurae*Broth. |
| Ditrichaceae | *Pleuridium* | *Pleuridium subulatum*(Hedw.) Rabenh. |
| Encalyptaceae | *Encalypta* | *Encalypta vulgaris*Hedw. |
| Entodontaceae | *Entodon* | *Entodon schensianus*Müll. Hal. |
| Fabroniaceae | *Anacamptodon* | *Anacamptodon latidens*(Besch.) Broth. |
| Fissidentaceae | *Fissidens* | *Fissidens bryoides*var.*bryoides* |
| Grimmiaceae | *Grimmia* | *Grimmia atrata* Miel. |
|  |  | *Grimmia pilifera* P. Beauv. |
| Hypnaceae | *Ctenidium* | *Ctenidium capillifolium*(Mitt.) Broth. |
|  | *Homomallium* | *Homomallium plagiangium* (Müll. Hal.) Broth. |
|  | *Hypnum* | *Hypnum revolutum*(Mitt.) Lindb. |
| Leskeaceae | *Lescuraea* | *Lescuraea incurvata*(Hedw.) E. Lawton |
|  | *Lindbergia* | *Lindbergia serrulata*C. Gao, T. Cao & W. H. Wang |
| Mniaceae | *Mnium* | *Mnium laevinerve*Cardot |
|  |  | *Mnium spinosum*Schwägr. |
|  | *Plagiomnium* | *Plagiomnium acutum* T. J. Kop. |
|  | *Rhizomnium* | *Rhizomnium punctatum*T. J. Kop. |
| Pottiaceae | *Barbula* | *Barbula fallax*Hedw. |
|  |  | *Barbula longicostata*Li |
|  |  | *Barbula rufidula* C. Muell. |
|  |  | *Barbula tectorum*C. Muell. |
|  |  | *Barbula unguiculata*Hedw. |
|  |  | *Barbula vinealis*Brid. |
|  | *Bryoerythrophyllum* | *Bryoerythrophyllum brachystegium*(Besch.) Saito |
|  |  | *Bryoerythrophyllum gymnostomum*(Broth.) P. C. Chen |
|  |  | *Bryoerythrophyllum recurvirostrum*(Hedw.) P. C. Chen |
|  |  | *Bryoerythrophyllum yunnanense*(Herzog) P. C. Chen |
|  | *Desmatodon* | *Desmatodon latifolius*(Hedw.) Brid. |
|  |  | *Desmatodon systylius*Schimp. |
|  | *Gymnostomum* | *Gymnostomum recurvirostre*Hedw. |
|  | *Hyophila* | *Hyophila spathulata*(Harv.) A. Jaeger |
|  | *Oxystegus* | *Oxystegus cuspidatus*(Doz. et Molk.) Chen |
|  | *Pseudosymblepharis* | *Pseudosymblepharis duriuscula*(Mitt.) P. C. Chen |
|  | *Stegonia* | *Stegonia latifolia*(Schwägr.) Vent. ex Broth. |
|  | *Streblotrichum* | *Streblotrichum convolutum*(Hedw.) P. Beauv. |
|  | *Timmiella* | *Timmiella anomala*(Bruch & Schimp.) Limpr. |
|  | *Tortella* | *Tortella tortuosa*(Hedw.) Limpr. |
|  | *Tortula* | *Tortula nankomontana*Nog. |
|  |  | *Tortula subulata*Hedw. |
|  | *Weissia* | *Weissia controversa*Hedw. |
|  |  | *Weissia planifolia*Dix. |
|  |  | *Weissia platyphylloides*Card. |
| Theliaceae | *Myurella* | *Myurella julacea*(Schwägr.) Schimp. |
| Thuidiaceae | *Actinothuidium* | *Actinothuidium hookeri*(Mitt.) Broth. |
|  | *Haplocladium* | *Haplocladium angustifolium*(Hampe & Müll. Hal.) Broth. |
|  | *Leptopterigynandrum* | *Leptopterigynandrum incurvatum*Broth. |
|  |  | *Leptopterigynandrum subintegrum*(Mitt.) Broth. |
|  | *Rauiella* | *Rauiella fujisana*(Paris) Reimers |
|  | *Thuidium* | *Thuidium delicatulum* (Hedw.) Mitt. |

**Table S2.** Meta-parameter values used in tuning BRT models.

| Meta-parameter | Tree complexity | Learning rate | Bag fraction |
| --- | --- | --- | --- |
| Values considered | 1, 2, 3, 4, 5, 6, 7, 8, 9, 10 | 0.0001, 0.0005, 0.001, 0.005, 0.01, 0.05 | 0.5, 0.55, 0.60, 0.65, 0.70, 0.75 |

**Table S3.** Possible combinations of the three meta-parameters (learning rate, tree complexity, bag fraction) for fitted 360 BRT models for *c*-value based on 10-fold cross validation (CV) and the associated model performance parameters. Numbers are sorted according to increasing CV deviance (in bold). RMSE represents root mean square error, and MAE represents mean absolute error.

| No. | Learning rate | Tree complexity | Bag fraction | **CV deviance** | Percentage of explained deviance (%) | CV correlation | Training data correlation | RMSE  (%) | MAE  (%) | *R*^2^ |
| --- | --- | --- | --- | --- | --- | --- | --- | --- | --- | --- |
| 1 | 0.01 | 3 | 0.75 | **6.379** | 25.268 | 0.514 | 0.789 | 1.803 | 1.446 | 0.619 |
| 2 | 0.01 | 4 | 0.75 | **6.379** | 25.268 | 0.514 | 0.789 | 1.803 | 1.446 | 0.619 |
| 3 | 0.01 | 5 | 0.75 | **6.379** | 25.268 | 0.514 | 0.789 | 1.803 | 1.446 | 0.619 |
| 4 | 0.01 | 6 | 0.75 | **6.379** | 25.268 | 0.514 | 0.789 | 1.803 | 1.446 | 0.619 |
| 5 | 0.01 | 7 | 0.75 | **6.379** | 25.268 | 0.514 | 0.789 | 1.803 | 1.446 | 0.619 |
| 6 | 0.01 | 8 | 0.75 | **6.379** | 25.268 | 0.514 | 0.789 | 1.803 | 1.446 | 0.619 |
| 7 | 0.01 | 9 | 0.75 | **6.379** | 25.268 | 0.514 | 0.789 | 1.803 | 1.446 | 0.619 |
| 8 | 0.01 | 10 | 0.75 | **6.379** | 25.268 | 0.514 | 0.789 | 1.803 | 1.446 | 0.619 |
| 9 | 0.01 | 2 | 0.75 | **6.379** | 25.268 | 0.514 | 0.786 | 1.815 | 1.457 | 0.614 |
| 10 | 0.01 | 2 | 0.70 | **6.405** | 24.968 | 0.502 | 0.781 | 1.836 | 1.479 | 0.605 |
| 11 | 0.01 | 3 | 0.70 | **6.405** | 24.968 | 0.502 | 0.781 | 1.836 | 1.479 | 0.605 |
| 12 | 0.01 | 4 | 0.70 | **6.405** | 24.968 | 0.502 | 0.781 | 1.836 | 1.479 | 0.605 |
| 13 | 0.01 | 5 | 0.70 | **6.405** | 24.968 | 0.502 | 0.781 | 1.836 | 1.479 | 0.605 |
| 14 | 0.01 | 6 | 0.70 | **6.405** | 24.968 | 0.502 | 0.781 | 1.836 | 1.479 | 0.605 |
| 15 | 0.01 | 7 | 0.70 | **6.405** | 24.968 | 0.502 | 0.781 | 1.836 | 1.479 | 0.605 |
| 16 | 0.01 | 8 | 0.70 | **6.405** | 24.968 | 0.502 | 0.781 | 1.836 | 1.479 | 0.605 |
| 17 | 0.01 | 9 | 0.70 | **6.405** | 24.968 | 0.502 | 0.781 | 1.836 | 1.479 | 0.605 |
| 18 | 0.01 | 10 | 0.70 | **6.405** | 24.968 | 0.502 | 0.781 | 1.836 | 1.479 | 0.605 |
| 19 | 0.005 | 3 | 0.75 | **6.417** | 24.830 | 0.500 | 0.781 | 1.839 | 1.488 | 0.604 |
| 20 | 0.005 | 4 | 0.75 | **6.417** | 24.830 | 0.500 | 0.781 | 1.839 | 1.488 | 0.604 |
| 21 | 0.005 | 5 | 0.75 | **6.417** | 24.830 | 0.500 | 0.781 | 1.839 | 1.488 | 0.604 |
| 22 | 0.005 | 6 | 0.75 | **6.417** | 24.830 | 0.500 | 0.781 | 1.839 | 1.488 | 0.604 |
| 23 | 0.005 | 7 | 0.75 | **6.417** | 24.830 | 0.500 | 0.781 | 1.839 | 1.488 | 0.604 |
| 24 | 0.005 | 8 | 0.75 | **6.417** | 24.830 | 0.500 | 0.781 | 1.839 | 1.488 | 0.604 |
| 25 | 0.005 | 9 | 0.75 | **6.417** | 24.830 | 0.500 | 0.781 | 1.839 | 1.488 | 0.604 |
| 26 | 0.005 | 10 | 0.75 | **6.417** | 24.830 | 0.500 | 0.781 | 1.839 | 1.488 | 0.604 |
| 27 | 0.005 | 2 | 0.75 | **6.417** | 24.830 | 0.500 | 0.777 | 1.852 | 1.500 | 0.598 |
| 28 | 0.005 | 2 | 0.70 | **6.429** | 24.689 | 0.504 | 0.786 | 1.817 | 1.461 | 0.613 |
| 29 | 0.005 | 3 | 0.70 | **6.429** | 24.689 | 0.504 | 0.786 | 1.817 | 1.461 | 0.613 |
| 30 | 0.005 | 4 | 0.70 | **6.429** | 24.689 | 0.504 | 0.786 | 1.817 | 1.461 | 0.613 |
| 31 | 0.005 | 5 | 0.70 | **6.429** | 24.689 | 0.504 | 0.786 | 1.817 | 1.461 | 0.613 |
| 32 | 0.005 | 6 | 0.70 | **6.429** | 24.689 | 0.504 | 0.786 | 1.817 | 1.461 | 0.613 |
| 33 | 0.005 | 7 | 0.70 | **6.429** | 24.689 | 0.504 | 0.786 | 1.817 | 1.461 | 0.613 |
| 34 | 0.005 | 8 | 0.70 | **6.429** | 24.689 | 0.504 | 0.786 | 1.817 | 1.461 | 0.613 |
| 35 | 0.005 | 9 | 0.70 | **6.429** | 24.689 | 0.504 | 0.786 | 1.817 | 1.461 | 0.613 |
| 36 | 0.005 | 10 | 0.70 | **6.429** | 24.689 | 0.504 | 0.786 | 1.817 | 1.461 | 0.613 |
| 37 | 0.001 | 3 | 0.75 | **6.462** | 24.302 | 0.487 | 0.769 | 1.891 | 1.531 | 0.581 |
| 38 | 0.001 | 4 | 0.75 | **6.462** | 24.302 | 0.487 | 0.769 | 1.891 | 1.531 | 0.581 |
| 39 | 0.001 | 5 | 0.75 | **6.462** | 24.302 | 0.487 | 0.769 | 1.891 | 1.531 | 0.581 |
| 40 | 0.001 | 6 | 0.75 | **6.462** | 24.302 | 0.487 | 0.769 | 1.891 | 1.531 | 0.581 |
| 41 | 0.001 | 7 | 0.75 | **6.462** | 24.302 | 0.487 | 0.769 | 1.891 | 1.531 | 0.581 |
| 42 | 0.001 | 8 | 0.75 | **6.462** | 24.302 | 0.487 | 0.769 | 1.891 | 1.531 | 0.581 |
| 43 | 0.001 | 9 | 0.75 | **6.462** | 24.302 | 0.487 | 0.769 | 1.891 | 1.531 | 0.581 |
| 44 | 0.001 | 10 | 0.75 | **6.462** | 24.302 | 0.487 | 0.769 | 1.891 | 1.531 | 0.581 |
| 45 | 0.001 | 2 | 0.75 | **6.462** | 24.302 | 0.487 | 0.766 | 1.902 | 1.540 | 0.576 |
| 46 | 0.001 | 2 | 0.70 | **6.478** | 24.112 | 0.483 | 0.762 | 1.917 | 1.554 | 0.570 |
| 47 | 0.001 | 3 | 0.70 | **6.478** | 24.112 | 0.483 | 0.762 | 1.917 | 1.554 | 0.570 |
| 48 | 0.001 | 4 | 0.70 | **6.478** | 24.112 | 0.483 | 0.762 | 1.917 | 1.554 | 0.570 |
| 49 | 0.001 | 5 | 0.70 | **6.478** | 24.112 | 0.483 | 0.762 | 1.917 | 1.554 | 0.570 |
| 50 | 0.001 | 6 | 0.70 | **6.478** | 24.112 | 0.483 | 0.762 | 1.917 | 1.554 | 0.570 |
| 51 | 0.001 | 7 | 0.70 | **6.478** | 24.112 | 0.483 | 0.762 | 1.917 | 1.554 | 0.570 |
| 52 | 0.001 | 8 | 0.70 | **6.478** | 24.112 | 0.483 | 0.762 | 1.917 | 1.554 | 0.570 |
| 53 | 0.001 | 9 | 0.70 | **6.478** | 24.112 | 0.483 | 0.762 | 1.917 | 1.554 | 0.570 |
| 54 | 0.001 | 10 | 0.70 | **6.478** | 24.112 | 0.483 | 0.762 | 1.917 | 1.554 | 0.570 |
| 55 | 0.0005 | 3 | 0.75 | **6.483** | 24.048 | 0.487 | 0.764 | 1.912 | 1.550 | 0.572 |
| 56 | 0.0005 | 4 | 0.75 | **6.483** | 24.048 | 0.487 | 0.764 | 1.912 | 1.550 | 0.572 |
| 57 | 0.0005 | 5 | 0.75 | **6.483** | 24.048 | 0.487 | 0.764 | 1.912 | 1.550 | 0.572 |
| 58 | 0.0005 | 6 | 0.75 | **6.483** | 24.048 | 0.487 | 0.764 | 1.912 | 1.550 | 0.572 |
| 59 | 0.0005 | 7 | 0.75 | **6.483** | 24.048 | 0.487 | 0.764 | 1.912 | 1.550 | 0.572 |
| 60 | 0.0005 | 8 | 0.75 | **6.483** | 24.048 | 0.487 | 0.764 | 1.912 | 1.550 | 0.572 |
| 61 | 0.0005 | 9 | 0.75 | **6.483** | 24.048 | 0.487 | 0.764 | 1.912 | 1.550 | 0.572 |
| 62 | 0.0005 | 10 | 0.75 | **6.483** | 24.048 | 0.487 | 0.764 | 1.912 | 1.550 | 0.572 |
| 63 | 0.0005 | 2 | 0.75 | **6.483** | 24.048 | 0.487 | 0.761 | 1.921 | 1.558 | 0.568 |
| 64 | 0.0005 | 2 | 0.70 | **6.493** | 23.939 | 0.483 | 0.756 | 1.941 | 1.578 | 0.559 |
| 65 | 0.0005 | 3 | 0.70 | **6.493** | 23.939 | 0.483 | 0.756 | 1.941 | 1.578 | 0.559 |
| 66 | 0.0005 | 4 | 0.70 | **6.493** | 23.939 | 0.483 | 0.756 | 1.941 | 1.578 | 0.559 |
| 67 | 0.0005 | 5 | 0.70 | **6.493** | 23.939 | 0.483 | 0.756 | 1.941 | 1.578 | 0.559 |
| 68 | 0.0005 | 6 | 0.70 | **6.493** | 23.939 | 0.483 | 0.756 | 1.941 | 1.578 | 0.559 |
| 69 | 0.0005 | 7 | 0.70 | **6.493** | 23.939 | 0.483 | 0.756 | 1.941 | 1.578 | 0.559 |
| 70 | 0.0005 | 8 | 0.70 | **6.493** | 23.939 | 0.483 | 0.756 | 1.941 | 1.578 | 0.559 |
| 71 | 0.0005 | 9 | 0.70 | **6.493** | 23.939 | 0.483 | 0.756 | 1.941 | 1.578 | 0.559 |
| 72 | 0.0005 | 10 | 0.70 | **6.493** | 23.939 | 0.483 | 0.756 | 1.941 | 1.578 | 0.559 |
| 73 | 0.005 | 2 | 0.65 | **6.542** | 23.359 | 0.482 | 0.775 | 1.860 | 1.504 | 0.595 |
| 74 | 0.005 | 3 | 0.65 | **6.542** | 23.359 | 0.482 | 0.775 | 1.860 | 1.504 | 0.595 |
| 75 | 0.005 | 4 | 0.65 | **6.542** | 23.359 | 0.482 | 0.775 | 1.860 | 1.504 | 0.595 |
| 76 | 0.005 | 5 | 0.65 | **6.542** | 23.359 | 0.482 | 0.775 | 1.860 | 1.504 | 0.595 |
| 77 | 0.005 | 6 | 0.65 | **6.542** | 23.359 | 0.482 | 0.775 | 1.860 | 1.504 | 0.595 |
| 78 | 0.005 | 7 | 0.65 | **6.542** | 23.359 | 0.482 | 0.775 | 1.860 | 1.504 | 0.595 |
| 79 | 0.005 | 8 | 0.65 | **6.542** | 23.359 | 0.482 | 0.775 | 1.860 | 1.504 | 0.595 |
| 80 | 0.005 | 9 | 0.65 | **6.542** | 23.359 | 0.482 | 0.775 | 1.860 | 1.504 | 0.595 |
| 81 | 0.005 | 10 | 0.65 | **6.542** | 23.359 | 0.482 | 0.775 | 1.860 | 1.504 | 0.595 |
| 82 | 0.01 | 2 | 0.65 | **6.585** | 22.857 | 0.479 | 0.775 | 1.857 | 1.503 | 0.596 |
| 83 | 0.01 | 3 | 0.65 | **6.585** | 22.857 | 0.479 | 0.775 | 1.857 | 1.503 | 0.596 |
| 84 | 0.01 | 4 | 0.65 | **6.585** | 22.857 | 0.479 | 0.775 | 1.857 | 1.503 | 0.596 |
| 85 | 0.01 | 5 | 0.65 | **6.585** | 22.857 | 0.479 | 0.775 | 1.857 | 1.503 | 0.596 |
| 86 | 0.01 | 6 | 0.65 | **6.585** | 22.857 | 0.479 | 0.775 | 1.857 | 1.503 | 0.596 |
| 87 | 0.01 | 7 | 0.65 | **6.585** | 22.857 | 0.479 | 0.775 | 1.857 | 1.503 | 0.596 |
| 88 | 0.01 | 8 | 0.65 | **6.585** | 22.857 | 0.479 | 0.775 | 1.857 | 1.503 | 0.596 |
| 89 | 0.01 | 9 | 0.65 | **6.585** | 22.857 | 0.479 | 0.775 | 1.857 | 1.503 | 0.596 |
| 90 | 0.01 | 10 | 0.65 | **6.585** | 22.857 | 0.479 | 0.775 | 1.857 | 1.503 | 0.596 |
| 91 | 0.001 | 2 | 0.65 | **6.630** | 22.330 | 0.466 | 0.756 | 1.935 | 1.570 | 0.561 |
| 92 | 0.001 | 3 | 0.65 | **6.630** | 22.330 | 0.466 | 0.756 | 1.935 | 1.570 | 0.561 |
| 93 | 0.001 | 4 | 0.65 | **6.630** | 22.330 | 0.466 | 0.756 | 1.935 | 1.570 | 0.561 |
| 94 | 0.001 | 5 | 0.65 | **6.630** | 22.330 | 0.466 | 0.756 | 1.935 | 1.570 | 0.561 |
| 95 | 0.001 | 6 | 0.65 | **6.630** | 22.330 | 0.466 | 0.756 | 1.935 | 1.570 | 0.561 |
| 96 | 0.001 | 7 | 0.65 | **6.630** | 22.330 | 0.466 | 0.756 | 1.935 | 1.570 | 0.561 |
| 97 | 0.001 | 8 | 0.65 | **6.630** | 22.330 | 0.466 | 0.756 | 1.935 | 1.570 | 0.561 |
| 98 | 0.001 | 9 | 0.65 | **6.630** | 22.330 | 0.466 | 0.756 | 1.935 | 1.570 | 0.561 |
| 99 | 0.001 | 10 | 0.65 | **6.630** | 22.330 | 0.466 | 0.756 | 1.935 | 1.570 | 0.561 |
| 100 | 0.0005 | 2 | 0.65 | **6.654** | 22.049 | 0.466 | 0.747 | 1.971 | 1.603 | 0.545 |
| 101 | 0.0005 | 3 | 0.65 | **6.654** | 22.049 | 0.466 | 0.747 | 1.971 | 1.603 | 0.545 |
| 102 | 0.0005 | 4 | 0.65 | **6.654** | 22.049 | 0.466 | 0.747 | 1.971 | 1.603 | 0.545 |
| 103 | 0.0005 | 5 | 0.65 | **6.654** | 22.049 | 0.466 | 0.747 | 1.971 | 1.603 | 0.545 |
| 104 | 0.0005 | 6 | 0.65 | **6.654** | 22.049 | 0.466 | 0.747 | 1.971 | 1.603 | 0.545 |
| 105 | 0.0005 | 7 | 0.65 | **6.654** | 22.049 | 0.466 | 0.747 | 1.971 | 1.603 | 0.545 |
| 106 | 0.0005 | 8 | 0.65 | **6.654** | 22.049 | 0.466 | 0.747 | 1.971 | 1.603 | 0.545 |
| 107 | 0.0005 | 9 | 0.65 | **6.654** | 22.049 | 0.466 | 0.747 | 1.971 | 1.603 | 0.545 |
| 108 | 0.0005 | 10 | 0.65 | **6.654** | 22.049 | 0.466 | 0.747 | 1.971 | 1.603 | 0.545 |
| 109 | 0.0001 | 2 | 0.70 | **6.832** | 19.967 | 0.472 | 0.692 | 2.169 | 1.772 | 0.449 |
| 110 | 0.0001 | 3 | 0.70 | **6.832** | 19.967 | 0.472 | 0.692 | 2.169 | 1.772 | 0.449 |
| 111 | 0.0001 | 4 | 0.70 | **6.832** | 19.967 | 0.472 | 0.692 | 2.169 | 1.772 | 0.449 |
| 112 | 0.0001 | 5 | 0.70 | **6.832** | 19.967 | 0.472 | 0.692 | 2.169 | 1.772 | 0.449 |
| 113 | 0.0001 | 6 | 0.70 | **6.832** | 19.967 | 0.472 | 0.692 | 2.169 | 1.772 | 0.449 |
| 114 | 0.0001 | 7 | 0.70 | **6.832** | 19.967 | 0.472 | 0.692 | 2.169 | 1.772 | 0.449 |
| 115 | 0.0001 | 8 | 0.70 | **6.832** | 19.967 | 0.472 | 0.692 | 2.169 | 1.772 | 0.449 |
| 116 | 0.0001 | 9 | 0.70 | **6.832** | 19.967 | 0.472 | 0.692 | 2.169 | 1.772 | 0.449 |
| 117 | 0.0001 | 10 | 0.70 | **6.832** | 19.967 | 0.472 | 0.692 | 2.169 | 1.772 | 0.449 |
| 118 | 0.0001 | 3 | 0.75 | **6.861** | 19.617 | 0.467 | 0.693 | 2.169 | 1.775 | 0.449 |
| 119 | 0.0001 | 4 | 0.75 | **6.861** | 19.617 | 0.467 | 0.693 | 2.169 | 1.775 | 0.449 |
| 120 | 0.0001 | 5 | 0.75 | **6.861** | 19.617 | 0.467 | 0.693 | 2.169 | 1.775 | 0.449 |
| 121 | 0.0001 | 6 | 0.75 | **6.861** | 19.617 | 0.467 | 0.693 | 2.169 | 1.775 | 0.449 |
| 122 | 0.0001 | 7 | 0.75 | **6.861** | 19.617 | 0.467 | 0.693 | 2.169 | 1.775 | 0.449 |
| 123 | 0.0001 | 8 | 0.75 | **6.861** | 19.617 | 0.467 | 0.693 | 2.169 | 1.775 | 0.449 |
| 124 | 0.0001 | 9 | 0.75 | **6.861** | 19.617 | 0.467 | 0.693 | 2.169 | 1.775 | 0.449 |
| 125 | 0.0001 | 10 | 0.75 | **6.861** | 19.617 | 0.467 | 0.693 | 2.169 | 1.775 | 0.449 |
| 126 | 0.0001 | 2 | 0.75 | **6.861** | 19.617 | 0.467 | 0.690 | 2.176 | 1.780 | 0.445 |
| 127 | 0.005 | 2 | 0.60 | **6.878** | 19.423 | 0.439 | 0.748 | 1.961 | 1.594 | 0.549 |
| 128 | 0.005 | 3 | 0.60 | **6.878** | 19.423 | 0.439 | 0.748 | 1.961 | 1.594 | 0.549 |
| 129 | 0.005 | 4 | 0.60 | **6.878** | 19.423 | 0.439 | 0.748 | 1.961 | 1.594 | 0.549 |
| 130 | 0.005 | 5 | 0.60 | **6.878** | 19.423 | 0.439 | 0.748 | 1.961 | 1.594 | 0.549 |
| 131 | 0.005 | 6 | 0.60 | **6.878** | 19.423 | 0.439 | 0.748 | 1.961 | 1.594 | 0.549 |
| 132 | 0.005 | 7 | 0.60 | **6.878** | 19.423 | 0.439 | 0.748 | 1.961 | 1.594 | 0.549 |
| 133 | 0.005 | 8 | 0.60 | **6.878** | 19.423 | 0.439 | 0.748 | 1.961 | 1.594 | 0.549 |
| 134 | 0.005 | 9 | 0.60 | **6.878** | 19.423 | 0.439 | 0.748 | 1.961 | 1.594 | 0.549 |
| 135 | 0.005 | 10 | 0.60 | **6.878** | 19.423 | 0.439 | 0.748 | 1.961 | 1.594 | 0.549 |
| 136 | 0.01 | 2 | 0.60 | **6.907** | 19.080 | 0.440 | 0.757 | 1.928 | 1.563 | 0.565 |
| 137 | 0.01 | 3 | 0.60 | **6.907** | 19.080 | 0.440 | 0.757 | 1.928 | 1.563 | 0.565 |
| 138 | 0.01 | 4 | 0.60 | **6.907** | 19.080 | 0.440 | 0.757 | 1.928 | 1.563 | 0.565 |
| 139 | 0.01 | 5 | 0.60 | **6.907** | 19.080 | 0.440 | 0.757 | 1.928 | 1.563 | 0.565 |
| 140 | 0.01 | 6 | 0.60 | **6.907** | 19.080 | 0.440 | 0.757 | 1.928 | 1.563 | 0.565 |
| 141 | 0.01 | 7 | 0.60 | **6.907** | 19.080 | 0.440 | 0.757 | 1.928 | 1.563 | 0.565 |
| 142 | 0.01 | 8 | 0.60 | **6.907** | 19.080 | 0.440 | 0.757 | 1.928 | 1.563 | 0.565 |
| 143 | 0.01 | 9 | 0.60 | **6.907** | 19.080 | 0.440 | 0.757 | 1.928 | 1.563 | 0.565 |
| 144 | 0.01 | 10 | 0.60 | **6.907** | 19.080 | 0.440 | 0.757 | 1.928 | 1.563 | 0.565 |
| 145 | 0.001 | 2 | 0.60 | **6.918** | 18.955 | 0.436 | 0.731 | 2.024 | 1.651 | 0.520 |
| 146 | 0.001 | 3 | 0.60 | **6.918** | 18.955 | 0.436 | 0.731 | 2.024 | 1.651 | 0.520 |
| 147 | 0.001 | 4 | 0.60 | **6.918** | 18.955 | 0.436 | 0.731 | 2.024 | 1.651 | 0.520 |
| 148 | 0.001 | 5 | 0.60 | **6.918** | 18.955 | 0.436 | 0.731 | 2.024 | 1.651 | 0.520 |
| 149 | 0.001 | 6 | 0.60 | **6.918** | 18.955 | 0.436 | 0.731 | 2.024 | 1.651 | 0.520 |
| 150 | 0.001 | 7 | 0.60 | **6.918** | 18.955 | 0.436 | 0.731 | 2.024 | 1.651 | 0.520 |
| 151 | 0.001 | 8 | 0.60 | **6.918** | 18.955 | 0.436 | 0.731 | 2.024 | 1.651 | 0.520 |
| 152 | 0.001 | 9 | 0.60 | **6.918** | 18.955 | 0.436 | 0.731 | 2.024 | 1.651 | 0.520 |
| 153 | 0.001 | 10 | 0.60 | **6.918** | 18.955 | 0.436 | 0.731 | 2.024 | 1.651 | 0.520 |
| 154 | 0.0001 | 2 | 0.65 | **6.965** | 18.410 | 0.464 | 0.674 | 2.229 | 1.811 | 0.418 |
| 155 | 0.0001 | 3 | 0.65 | **6.965** | 18.410 | 0.464 | 0.674 | 2.229 | 1.811 | 0.418 |
| 156 | 0.0001 | 4 | 0.65 | **6.965** | 18.410 | 0.464 | 0.674 | 2.229 | 1.811 | 0.418 |
| 157 | 0.0001 | 5 | 0.65 | **6.965** | 18.410 | 0.464 | 0.674 | 2.229 | 1.811 | 0.418 |
| 158 | 0.0001 | 6 | 0.65 | **6.965** | 18.410 | 0.464 | 0.674 | 2.229 | 1.811 | 0.418 |
| 159 | 0.0001 | 7 | 0.65 | **6.965** | 18.410 | 0.464 | 0.674 | 2.229 | 1.811 | 0.418 |
| 160 | 0.0001 | 8 | 0.65 | **6.965** | 18.410 | 0.464 | 0.674 | 2.229 | 1.811 | 0.418 |
| 161 | 0.0001 | 9 | 0.65 | **6.965** | 18.410 | 0.464 | 0.674 | 2.229 | 1.811 | 0.418 |
| 162 | 0.0001 | 10 | 0.65 | **6.965** | 18.410 | 0.464 | 0.674 | 2.229 | 1.811 | 0.418 |
| 163 | 0.0005 | 2 | 0.60 | **6.966** | 18.397 | 0.435 | 0.714 | 2.078 | 1.693 | 0.494 |
| 164 | 0.0005 | 3 | 0.60 | **6.966** | 18.397 | 0.435 | 0.714 | 2.078 | 1.693 | 0.494 |
| 165 | 0.0005 | 4 | 0.60 | **6.966** | 18.397 | 0.435 | 0.714 | 2.078 | 1.693 | 0.494 |
| 166 | 0.0005 | 5 | 0.60 | **6.966** | 18.397 | 0.435 | 0.714 | 2.078 | 1.693 | 0.494 |
| 167 | 0.0005 | 6 | 0.60 | **6.966** | 18.397 | 0.435 | 0.714 | 2.078 | 1.693 | 0.494 |
| 168 | 0.0005 | 7 | 0.60 | **6.966** | 18.397 | 0.435 | 0.714 | 2.078 | 1.693 | 0.494 |
| 169 | 0.0005 | 8 | 0.60 | **6.966** | 18.397 | 0.435 | 0.714 | 2.078 | 1.693 | 0.494 |
| 170 | 0.0005 | 9 | 0.60 | **6.966** | 18.397 | 0.435 | 0.714 | 2.078 | 1.693 | 0.494 |
| 171 | 0.0005 | 10 | 0.60 | **6.966** | 18.397 | 0.435 | 0.714 | 2.078 | 1.693 | 0.494 |
| 172 | 0.005 | 2 | 0.55 | **7.066** | 17.217 | 0.414 | 0.684 | 2.158 | 1.761 | 0.454 |
| 173 | 0.005 | 3 | 0.55 | **7.066** | 17.217 | 0.414 | 0.684 | 2.158 | 1.761 | 0.454 |
| 174 | 0.005 | 4 | 0.55 | **7.066** | 17.217 | 0.414 | 0.684 | 2.158 | 1.761 | 0.454 |
| 175 | 0.005 | 5 | 0.55 | **7.066** | 17.217 | 0.414 | 0.684 | 2.158 | 1.761 | 0.454 |
| 176 | 0.005 | 6 | 0.55 | **7.066** | 17.217 | 0.414 | 0.684 | 2.158 | 1.761 | 0.454 |
| 177 | 0.005 | 7 | 0.55 | **7.066** | 17.217 | 0.414 | 0.684 | 2.158 | 1.761 | 0.454 |
| 178 | 0.005 | 8 | 0.55 | **7.066** | 17.217 | 0.414 | 0.684 | 2.158 | 1.761 | 0.454 |
| 179 | 0.005 | 9 | 0.55 | **7.066** | 17.217 | 0.414 | 0.684 | 2.158 | 1.761 | 0.454 |
| 180 | 0.005 | 10 | 0.55 | **7.066** | 17.217 | 0.414 | 0.684 | 2.158 | 1.761 | 0.454 |
| 181 | 0.005 | 1 | 0.55 | **7.066** | 17.217 | 0.414 | 0.664 | 2.211 | 1.795 | 0.427 |
| 182 | 0.001 | 1 | 0.50 | **7.067** | 17.205 | 0.405 | 0.647 | 2.259 | 1.832 | 0.402 |
| 183 | 0.001 | 2 | 0.50 | **7.067** | 17.205 | 0.405 | 0.647 | 2.259 | 1.832 | 0.402 |
| 184 | 0.001 | 3 | 0.50 | **7.067** | 17.205 | 0.405 | 0.647 | 2.259 | 1.832 | 0.402 |
| 185 | 0.001 | 4 | 0.50 | **7.067** | 17.205 | 0.405 | 0.647 | 2.259 | 1.832 | 0.402 |
| 186 | 0.001 | 5 | 0.50 | **7.067** | 17.205 | 0.405 | 0.647 | 2.259 | 1.832 | 0.402 |
| 187 | 0.001 | 6 | 0.50 | **7.067** | 17.205 | 0.405 | 0.647 | 2.259 | 1.832 | 0.402 |
| 188 | 0.001 | 7 | 0.50 | **7.067** | 17.205 | 0.405 | 0.647 | 2.259 | 1.832 | 0.402 |
| 189 | 0.001 | 8 | 0.50 | **7.067** | 17.205 | 0.405 | 0.647 | 2.259 | 1.832 | 0.402 |
| 190 | 0.001 | 9 | 0.50 | **7.067** | 17.205 | 0.405 | 0.647 | 2.259 | 1.832 | 0.402 |
| 191 | 0.001 | 10 | 0.50 | **7.067** | 17.205 | 0.405 | 0.647 | 2.259 | 1.832 | 0.402 |
| 192 | 0.01 | 2 | 0.55 | **7.072** | 17.146 | 0.410 | 0.679 | 2.172 | 1.776 | 0.448 |
| 193 | 0.01 | 3 | 0.55 | **7.072** | 17.146 | 0.410 | 0.679 | 2.172 | 1.776 | 0.448 |
| 194 | 0.01 | 4 | 0.55 | **7.072** | 17.146 | 0.410 | 0.679 | 2.172 | 1.776 | 0.448 |
| 195 | 0.01 | 5 | 0.55 | **7.072** | 17.146 | 0.410 | 0.679 | 2.172 | 1.776 | 0.448 |
| 196 | 0.01 | 6 | 0.55 | **7.072** | 17.146 | 0.410 | 0.679 | 2.172 | 1.776 | 0.448 |
| 197 | 0.01 | 7 | 0.55 | **7.072** | 17.146 | 0.410 | 0.679 | 2.172 | 1.776 | 0.448 |
| 198 | 0.01 | 8 | 0.55 | **7.072** | 17.146 | 0.410 | 0.679 | 2.172 | 1.776 | 0.448 |
| 199 | 0.01 | 9 | 0.55 | **7.072** | 17.146 | 0.410 | 0.679 | 2.172 | 1.776 | 0.448 |
| 200 | 0.01 | 10 | 0.55 | **7.072** | 17.146 | 0.410 | 0.679 | 2.172 | 1.776 | 0.448 |
| 201 | 0.01 | 1 | 0.55 | **7.072** | 17.146 | 0.410 | 0.658 | 2.224 | 1.812 | 0.421 |
| 202 | 0.0005 | 1 | 0.50 | **7.077** | 17.096 | 0.406 | 0.634 | 2.298 | 1.855 | 0.382 |
| 203 | 0.0005 | 2 | 0.50 | **7.077** | 17.096 | 0.406 | 0.634 | 2.298 | 1.855 | 0.382 |
| 204 | 0.0005 | 3 | 0.50 | **7.077** | 17.096 | 0.406 | 0.634 | 2.298 | 1.855 | 0.382 |
| 205 | 0.0005 | 4 | 0.50 | **7.077** | 17.096 | 0.406 | 0.634 | 2.298 | 1.855 | 0.382 |
| 206 | 0.0005 | 5 | 0.50 | **7.077** | 17.096 | 0.406 | 0.634 | 2.298 | 1.855 | 0.382 |
| 207 | 0.0005 | 6 | 0.50 | **7.077** | 17.096 | 0.406 | 0.634 | 2.298 | 1.855 | 0.382 |
| 208 | 0.0005 | 7 | 0.50 | **7.077** | 17.096 | 0.406 | 0.634 | 2.298 | 1.855 | 0.382 |
| 209 | 0.0005 | 8 | 0.50 | **7.077** | 17.096 | 0.406 | 0.634 | 2.298 | 1.855 | 0.382 |
| 210 | 0.0005 | 9 | 0.50 | **7.077** | 17.096 | 0.406 | 0.634 | 2.298 | 1.855 | 0.382 |
| 211 | 0.0005 | 10 | 0.50 | **7.077** | 17.096 | 0.406 | 0.634 | 2.298 | 1.855 | 0.382 |
| 212 | 0.005 | 1 | 0.50 | **7.087** | 16.970 | 0.404 | 0.637 | 2.286 | 1.844 | 0.388 |
| 213 | 0.005 | 2 | 0.50 | **7.087** | 16.970 | 0.404 | 0.637 | 2.286 | 1.844 | 0.388 |
| 214 | 0.005 | 3 | 0.50 | **7.087** | 16.970 | 0.404 | 0.637 | 2.286 | 1.844 | 0.388 |
| 215 | 0.005 | 4 | 0.50 | **7.087** | 16.970 | 0.404 | 0.637 | 2.286 | 1.844 | 0.388 |
| 216 | 0.005 | 5 | 0.50 | **7.087** | 16.970 | 0.404 | 0.637 | 2.286 | 1.844 | 0.388 |
| 217 | 0.005 | 6 | 0.50 | **7.087** | 16.970 | 0.404 | 0.637 | 2.286 | 1.844 | 0.388 |
| 218 | 0.005 | 7 | 0.50 | **7.087** | 16.970 | 0.404 | 0.637 | 2.286 | 1.844 | 0.388 |
| 219 | 0.005 | 8 | 0.50 | **7.087** | 16.970 | 0.404 | 0.637 | 2.286 | 1.844 | 0.388 |
| 220 | 0.005 | 9 | 0.50 | **7.087** | 16.970 | 0.404 | 0.637 | 2.286 | 1.844 | 0.388 |
| 221 | 0.005 | 10 | 0.50 | **7.087** | 16.970 | 0.404 | 0.637 | 2.286 | 1.844 | 0.388 |
| 222 | 0.001 | 2 | 0.55 | **7.088** | 16.958 | 0.411 | 0.673 | 2.194 | 1.782 | 0.436 |
| 223 | 0.001 | 3 | 0.55 | **7.088** | 16.958 | 0.411 | 0.673 | 2.194 | 1.782 | 0.436 |
| 224 | 0.001 | 4 | 0.55 | **7.088** | 16.958 | 0.411 | 0.673 | 2.194 | 1.782 | 0.436 |
| 225 | 0.001 | 5 | 0.55 | **7.088** | 16.958 | 0.411 | 0.673 | 2.194 | 1.782 | 0.436 |
| 226 | 0.001 | 6 | 0.55 | **7.088** | 16.958 | 0.411 | 0.673 | 2.194 | 1.782 | 0.436 |
| 227 | 0.001 | 7 | 0.55 | **7.088** | 16.958 | 0.411 | 0.673 | 2.194 | 1.782 | 0.436 |
| 228 | 0.001 | 8 | 0.55 | **7.088** | 16.958 | 0.411 | 0.673 | 2.194 | 1.782 | 0.436 |
| 229 | 0.001 | 9 | 0.55 | **7.088** | 16.958 | 0.411 | 0.673 | 2.194 | 1.782 | 0.436 |
| 230 | 0.001 | 10 | 0.55 | **7.088** | 16.958 | 0.411 | 0.673 | 2.194 | 1.782 | 0.436 |
| 231 | 0.001 | 1 | 0.55 | **7.088** | 16.958 | 0.411 | 0.653 | 2.245 | 1.816 | 0.409 |
| 232 | 0.005 | 1 | 0.60 | **7.089** | 16.950 | 0.412 | 0.690 | 2.137 | 1.734 | 0.465 |
| 233 | 0.01 | 1 | 0.50 | **7.092** | 16.918 | 0.408 | 0.642 | 2.274 | 1.841 | 0.394 |
| 234 | 0.01 | 2 | 0.50 | **7.092** | 16.918 | 0.408 | 0.642 | 2.274 | 1.841 | 0.394 |
| 235 | 0.01 | 3 | 0.50 | **7.092** | 16.918 | 0.408 | 0.642 | 2.274 | 1.841 | 0.394 |
| 236 | 0.01 | 4 | 0.50 | **7.092** | 16.918 | 0.408 | 0.642 | 2.274 | 1.841 | 0.394 |
| 237 | 0.01 | 5 | 0.50 | **7.092** | 16.918 | 0.408 | 0.642 | 2.274 | 1.841 | 0.394 |
| 238 | 0.01 | 6 | 0.50 | **7.092** | 16.918 | 0.408 | 0.642 | 2.274 | 1.841 | 0.394 |
| 239 | 0.01 | 7 | 0.50 | **7.092** | 16.918 | 0.408 | 0.642 | 2.274 | 1.841 | 0.394 |
| 240 | 0.01 | 8 | 0.50 | **7.092** | 16.918 | 0.408 | 0.642 | 2.274 | 1.841 | 0.394 |
| 241 | 0.01 | 9 | 0.50 | **7.092** | 16.918 | 0.408 | 0.642 | 2.274 | 1.841 | 0.394 |
| 242 | 0.01 | 10 | 0.50 | **7.092** | 16.918 | 0.408 | 0.642 | 2.274 | 1.841 | 0.394 |
| 243 | 0.0005 | 2 | 0.55 | **7.110** | 16.703 | 0.413 | 0.659 | 2.241 | 1.817 | 0.412 |
| 244 | 0.0005 | 3 | 0.55 | **7.110** | 16.703 | 0.413 | 0.659 | 2.241 | 1.817 | 0.412 |
| 245 | 0.0005 | 4 | 0.55 | **7.110** | 16.703 | 0.413 | 0.659 | 2.241 | 1.817 | 0.412 |
| 246 | 0.0005 | 5 | 0.55 | **7.110** | 16.703 | 0.413 | 0.659 | 2.241 | 1.817 | 0.412 |
| 247 | 0.0005 | 6 | 0.55 | **7.110** | 16.703 | 0.413 | 0.659 | 2.241 | 1.817 | 0.412 |
| 248 | 0.0005 | 7 | 0.55 | **7.110** | 16.703 | 0.413 | 0.659 | 2.241 | 1.817 | 0.412 |
| 249 | 0.0005 | 8 | 0.55 | **7.110** | 16.703 | 0.413 | 0.659 | 2.241 | 1.817 | 0.412 |
| 250 | 0.0005 | 9 | 0.55 | **7.110** | 16.703 | 0.413 | 0.659 | 2.241 | 1.817 | 0.412 |
| 251 | 0.0005 | 10 | 0.55 | **7.110** | 16.703 | 0.413 | 0.659 | 2.241 | 1.817 | 0.412 |
| 252 | 0.0005 | 1 | 0.55 | **7.110** | 16.703 | 0.413 | 0.637 | 2.293 | 1.849 | 0.384 |
| 253 | 0.005 | 1 | 0.65 | **7.128** | 16.491 | 0.405 | 0.697 | 2.117 | 1.729 | 0.475 |
| 254 | 0.01 | 1 | 0.60 | **7.131** | 16.456 | 0.409 | 0.665 | 2.210 | 1.795 | 0.428 |
| 255 | 0.005 | 1 | 0.70 | **7.177** | 15.919 | 0.395 | 0.704 | 2.092 | 1.710 | 0.487 |
| 256 | 0.01 | 1 | 0.70 | **7.191** | 15.758 | 0.394 | 0.710 | 2.074 | 1.695 | 0.496 |
| 257 | 0.001 | 1 | 0.60 | **7.192** | 15.745 | 0.411 | 0.647 | 2.263 | 1.822 | 0.400 |
| 258 | 0.01 | 1 | 0.65 | **7.194** | 15.720 | 0.402 | 0.710 | 2.073 | 1.699 | 0.496 |
| 259 | 0.05 | 1 | 0.75 | **7.203** | 15.616 | 0.390 | 0.713 | 2.056 | 1.671 | 0.505 |
| 260 | 0.0005 | 1 | 0.60 | **7.204** | 15.603 | 0.412 | 0.640 | 2.285 | 1.837 | 0.388 |
| 261 | 0.0001 | 2 | 0.60 | **7.206** | 15.577 | 0.435 | 0.654 | 2.281 | 1.842 | 0.390 |
| 262 | 0.0001 | 3 | 0.60 | **7.206** | 15.577 | 0.435 | 0.654 | 2.281 | 1.842 | 0.390 |
| 263 | 0.0001 | 4 | 0.60 | **7.206** | 15.577 | 0.435 | 0.654 | 2.281 | 1.842 | 0.390 |
| 264 | 0.0001 | 5 | 0.60 | **7.206** | 15.577 | 0.435 | 0.654 | 2.281 | 1.842 | 0.390 |
| 265 | 0.0001 | 6 | 0.60 | **7.206** | 15.577 | 0.435 | 0.654 | 2.281 | 1.842 | 0.390 |
| 266 | 0.0001 | 7 | 0.60 | **7.206** | 15.577 | 0.435 | 0.654 | 2.281 | 1.842 | 0.390 |
| 267 | 0.0001 | 8 | 0.60 | **7.206** | 15.577 | 0.435 | 0.654 | 2.281 | 1.842 | 0.390 |
| 268 | 0.0001 | 9 | 0.60 | **7.206** | 15.577 | 0.435 | 0.654 | 2.281 | 1.842 | 0.390 |
| 269 | 0.0001 | 10 | 0.60 | **7.206** | 15.577 | 0.435 | 0.654 | 2.281 | 1.842 | 0.390 |
| 270 | 0.005 | 1 | 0.75 | **7.210** | 15.537 | 0.388 | 0.710 | 2.074 | 1.691 | 0.496 |
| 271 | 0.05 | 1 | 0.70 | **7.214** | 15.487 | 0.394 | 0.715 | 2.051 | 1.672 | 0.507 |
| 272 | 0.01 | 1 | 0.75 | **7.219** | 15.430 | 0.387 | 0.707 | 2.082 | 1.698 | 0.492 |
| 273 | 0.001 | 1 | 0.65 | **7.236** | 15.225 | 0.401 | 0.672 | 2.194 | 1.777 | 0.436 |
| 274 | 0.0001 | 1 | 0.50 | **7.279** | 14.721 | 0.413 | 0.583 | 2.439 | 1.950 | 0.303 |
| 275 | 0.0001 | 2 | 0.50 | **7.279** | 14.721 | 0.413 | 0.583 | 2.439 | 1.950 | 0.303 |
| 276 | 0.0001 | 3 | 0.50 | **7.279** | 14.721 | 0.413 | 0.583 | 2.439 | 1.950 | 0.303 |
| 277 | 0.0001 | 4 | 0.50 | **7.279** | 14.721 | 0.413 | 0.583 | 2.439 | 1.950 | 0.303 |
| 278 | 0.0001 | 5 | 0.50 | **7.279** | 14.721 | 0.413 | 0.583 | 2.439 | 1.950 | 0.303 |
| 279 | 0.0001 | 6 | 0.50 | **7.279** | 14.721 | 0.413 | 0.583 | 2.439 | 1.950 | 0.303 |
| 280 | 0.0001 | 7 | 0.50 | **7.279** | 14.721 | 0.413 | 0.583 | 2.439 | 1.950 | 0.303 |
| 281 | 0.0001 | 8 | 0.50 | **7.279** | 14.721 | 0.413 | 0.583 | 2.439 | 1.950 | 0.303 |
| 282 | 0.0001 | 9 | 0.50 | **7.279** | 14.721 | 0.413 | 0.583 | 2.439 | 1.950 | 0.303 |
| 283 | 0.0001 | 10 | 0.50 | **7.279** | 14.721 | 0.413 | 0.583 | 2.439 | 1.950 | 0.303 |
| 284 | 0.0001 | 2 | 0.55 | **7.288** | 14.618 | 0.418 | 0.609 | 2.386 | 1.908 | 0.333 |
| 285 | 0.0001 | 3 | 0.55 | **7.288** | 14.618 | 0.418 | 0.609 | 2.386 | 1.908 | 0.333 |
| 286 | 0.0001 | 4 | 0.55 | **7.288** | 14.618 | 0.418 | 0.609 | 2.386 | 1.908 | 0.333 |
| 287 | 0.0001 | 5 | 0.55 | **7.288** | 14.618 | 0.418 | 0.609 | 2.386 | 1.908 | 0.333 |
| 288 | 0.0001 | 6 | 0.55 | **7.288** | 14.618 | 0.418 | 0.609 | 2.386 | 1.908 | 0.333 |
| 289 | 0.0001 | 7 | 0.55 | **7.288** | 14.618 | 0.418 | 0.609 | 2.386 | 1.908 | 0.333 |
| 290 | 0.0001 | 8 | 0.55 | **7.288** | 14.618 | 0.418 | 0.609 | 2.386 | 1.908 | 0.333 |
| 291 | 0.0001 | 9 | 0.55 | **7.288** | 14.618 | 0.418 | 0.609 | 2.386 | 1.908 | 0.333 |
| 292 | 0.0001 | 10 | 0.55 | **7.288** | 14.618 | 0.418 | 0.609 | 2.386 | 1.908 | 0.333 |
| 293 | 0.0001 | 1 | 0.55 | **7.288** | 14.618 | 0.418 | 0.587 | 2.427 | 1.939 | 0.310 |
| 294 | 0.001 | 1 | 0.70 | **7.297** | 14.513 | 0.392 | 0.676 | 2.180 | 1.767 | 0.443 |
| 295 | 0.0005 | 1 | 0.65 | **7.303** | 14.442 | 0.407 | 0.640 | 2.285 | 1.838 | 0.388 |
| 296 | 0.001 | 1 | 0.75 | **7.336** | 14.060 | 0.384 | 0.681 | 2.163 | 1.754 | 0.452 |
| 297 | 0.0001 | 1 | 0.60 | **7.396** | 13.351 | 0.414 | 0.579 | 2.442 | 1.950 | 0.302 |
| 298 | 0.0005 | 1 | 0.70 | **7.409** | 13.198 | 0.400 | 0.638 | 2.290 | 1.841 | 0.386 |
| 299 | 0.0005 | 1 | 0.75 | **7.497** | 12.170 | 0.393 | 0.636 | 2.295 | 1.846 | 0.383 |
| 300 | 0.0001 | 1 | 0.65 | **7.501** | 12.129 | 0.408 | 0.570 | 2.460 | 1.971 | 0.291 |
| 301 | 0.0001 | 1 | 0.70 | **7.628** | 10.640 | 0.397 | 0.554 | 2.493 | 2.005 | 0.272 |
| 302 | 0.0001 | 1 | 0.75 | **7.710** | 9.672 | 0.389 | 0.542 | 2.514 | 2.027 | 0.259 |
| 303 | 0.05 | 1 | 0.50 | **NA** | NA | NA | NA | NA | NA | NA |
| 304 | 0.05 | 2 | 0.50 | **NA** | NA | NA | NA | NA | NA | NA |
| 305 | 0.05 | 3 | 0.50 | **NA** | NA | NA | NA | NA | NA | NA |
| 306 | 0.05 | 4 | 0.50 | **NA** | NA | NA | NA | NA | NA | NA |
| 307 | 0.05 | 5 | 0.50 | **NA** | NA | NA | NA | NA | NA | NA |
| 308 | 0.05 | 6 | 0.50 | **NA** | NA | NA | NA | NA | NA | NA |
| 309 | 0.05 | 7 | 0.50 | **NA** | NA | NA | NA | NA | NA | NA |
| 310 | 0.05 | 8 | 0.50 | **NA** | NA | NA | NA | NA | NA | NA |
| 311 | 0.05 | 9 | 0.50 | **NA** | NA | NA | NA | NA | NA | NA |
| 312 | 0.05 | 10 | 0.50 | **NA** | NA | NA | NA | NA | NA | NA |
| 313 | 0.05 | 1 | 0.55 | **NA** | NA | NA | NA | NA | NA | NA |
| 314 | 0.05 | 2 | 0.55 | **NA** | NA | NA | NA | NA | NA | NA |
| 315 | 0.05 | 3 | 0.55 | **NA** | NA | NA | NA | NA | NA | NA |
| 316 | 0.05 | 4 | 0.55 | **NA** | NA | NA | NA | NA | NA | NA |
| 317 | 0.05 | 5 | 0.55 | **NA** | NA | NA | NA | NA | NA | NA |
| 318 | 0.05 | 6 | 0.55 | **NA** | NA | NA | NA | NA | NA | NA |
| 319 | 0.05 | 7 | 0.55 | **NA** | NA | NA | NA | NA | NA | NA |
| 320 | 0.05 | 8 | 0.55 | **NA** | NA | NA | NA | NA | NA | NA |
| 321 | 0.05 | 9 | 0.55 | **NA** | NA | NA | NA | NA | NA | NA |
| 322 | 0.05 | 10 | 0.55 | **NA** | NA | NA | NA | NA | NA | NA |
| 323 | 0.05 | 1 | 0.60 | **NA** | NA | NA | NA | NA | NA | NA |
| 324 | 0.05 | 2 | 0.60 | **NA** | NA | NA | NA | NA | NA | NA |
| 325 | 0.05 | 3 | 0.60 | **NA** | NA | NA | NA | NA | NA | NA |
| 326 | 0.05 | 4 | 0.60 | **NA** | NA | NA | NA | NA | NA | NA |
| 327 | 0.05 | 5 | 0.60 | **NA** | NA | NA | NA | NA | NA | NA |
| 328 | 0.05 | 6 | 0.60 | **NA** | NA | NA | NA | NA | NA | NA |
| 329 | 0.05 | 7 | 0.60 | **NA** | NA | NA | NA | NA | NA | NA |
| 330 | 0.05 | 8 | 0.60 | **NA** | NA | NA | NA | NA | NA | NA |
| 331 | 0.05 | 9 | 0.60 | **NA** | NA | NA | NA | NA | NA | NA |
| 332 | 0.05 | 10 | 0.60 | **NA** | NA | NA | NA | NA | NA | NA |
| 333 | 0.05 | 1 | 0.65 | **NA** | NA | NA | NA | NA | NA | NA |
| 334 | 0.05 | 2 | 0.65 | **NA** | NA | NA | NA | NA | NA | NA |
| 335 | 0.05 | 3 | 0.65 | **NA** | NA | NA | NA | NA | NA | NA |
| 336 | 0.05 | 4 | 0.65 | **NA** | NA | NA | NA | NA | NA | NA |
| 337 | 0.05 | 5 | 0.65 | **NA** | NA | NA | NA | NA | NA | NA |
| 338 | 0.05 | 6 | 0.65 | **NA** | NA | NA | NA | NA | NA | NA |
| 339 | 0.05 | 7 | 0.65 | **NA** | NA | NA | NA | NA | NA | NA |
| 340 | 0.05 | 8 | 0.65 | **NA** | NA | NA | NA | NA | NA | NA |
| 341 | 0.05 | 9 | 0.65 | **NA** | NA | NA | NA | NA | NA | NA |
| 342 | 0.05 | 10 | 0.65 | **NA** | NA | NA | NA | NA | NA | NA |
| 343 | 0.05 | 2 | 0.70 | **NA** | NA | NA | NA | NA | NA | NA |
| 344 | 0.05 | 3 | 0.70 | **NA** | NA | NA | NA | NA | NA | NA |
| 345 | 0.05 | 4 | 0.70 | **NA** | NA | NA | NA | NA | NA | NA |
| 346 | 0.05 | 5 | 0.70 | **NA** | NA | NA | NA | NA | NA | NA |
| 347 | 0.05 | 6 | 0.70 | **NA** | NA | NA | NA | NA | NA | NA |
| 348 | 0.05 | 7 | 0.70 | **NA** | NA | NA | NA | NA | NA | NA |
| 349 | 0.05 | 8 | 0.70 | **NA** | NA | NA | NA | NA | NA | NA |
| 350 | 0.05 | 9 | 0.70 | **NA** | NA | NA | NA | NA | NA | NA |
| 351 | 0.05 | 10 | 0.70 | **NA** | NA | NA | NA | NA | NA | NA |
| 352 | 0.05 | 2 | 0.75 | **NA** | NA | NA | NA | NA | NA | NA |
| 353 | 0.05 | 3 | 0.75 | **NA** | NA | NA | NA | NA | NA | NA |
| 354 | 0.05 | 4 | 0.75 | **NA** | NA | NA | NA | NA | NA | NA |
| 355 | 0.05 | 5 | 0.75 | **NA** | NA | NA | NA | NA | NA | NA |
| 356 | 0.05 | 6 | 0.75 | **NA** | NA | NA | NA | NA | NA | NA |
| 357 | 0.05 | 7 | 0.75 | **NA** | NA | NA | NA | NA | NA | NA |
| 358 | 0.05 | 8 | 0.75 | **NA** | NA | NA | NA | NA | NA | NA |
| 359 | 0.05 | 9 | 0.75 | **NA** | NA | NA | NA | NA | NA | NA |
| 360 | 0.05 | 10 | 0.75 | **NA** | NA | NA | NA | NA | NA | NA |

**Table S4.** Possible combinations of the three meta-parameters (learning rate, tree complexity, bag fraction) for fitted 360 BRT models for *z*-value based on 10-fold cross validation (CV) and the associated model performance parameters. Numbers are sorted according to increasing CV deviance (in bold). RMSE represents root mean square error, and MAE represents mean absolute error.

| No. | Learning rate | Tree complexity | Bag fraction | **CV deviance** | Percentage of explained deviance (%) | CV correlation | Training data correlation | RMSE  (%) | MAE  (%) | *R*^2^ |
| --- | --- | --- | --- | --- | --- | --- | --- | --- | --- | --- |
| 1 | 0.001 | 1 | 0.5 | **0.001** | 26.723 | 0.378 | 0.463 | 0.025 | 0.019 | 0.192 |
| 2 | 0.001 | 2 | 0.5 | **0.001** | 26.723 | 0.378 | 0.463 | 0.025 | 0.019 | 0.192 |
| 3 | 0.001 | 3 | 0.5 | **0.001** | 26.723 | 0.378 | 0.463 | 0.025 | 0.019 | 0.192 |
| 4 | 0.001 | 4 | 0.5 | **0.001** | 26.723 | 0.378 | 0.463 | 0.025 | 0.019 | 0.192 |
| 5 | 0.001 | 5 | 0.5 | **0.001** | 26.723 | 0.378 | 0.463 | 0.025 | 0.019 | 0.192 |
| 6 | 0.001 | 6 | 0.5 | **0.001** | 26.723 | 0.378 | 0.463 | 0.025 | 0.019 | 0.192 |
| 7 | 0.001 | 7 | 0.5 | **0.001** | 26.723 | 0.378 | 0.463 | 0.025 | 0.019 | 0.192 |
| 8 | 0.001 | 8 | 0.5 | **0.001** | 26.723 | 0.378 | 0.463 | 0.025 | 0.019 | 0.192 |
| 9 | 0.001 | 9 | 0.5 | **0.001** | 26.723 | 0.378 | 0.463 | 0.025 | 0.019 | 0.192 |
| 10 | 0.001 | 10 | 0.5 | **0.001** | 26.723 | 0.378 | 0.463 | 0.025 | 0.019 | 0.192 |
| 11 | 0.0005 | 1 | 0.5 | **0.001** | 26.661 | 0.376 | 0.464 | 0.025 | 0.019 | 0.196 |
| 12 | 0.0005 | 2 | 0.5 | **0.001** | 26.661 | 0.376 | 0.464 | 0.025 | 0.019 | 0.196 |
| 13 | 0.0005 | 3 | 0.5 | **0.001** | 26.661 | 0.376 | 0.464 | 0.025 | 0.019 | 0.196 |
| 14 | 0.0005 | 4 | 0.5 | **0.001** | 26.661 | 0.376 | 0.464 | 0.025 | 0.019 | 0.196 |
| 15 | 0.0005 | 5 | 0.5 | **0.001** | 26.661 | 0.376 | 0.464 | 0.025 | 0.019 | 0.196 |
| 16 | 0.0005 | 6 | 0.5 | **0.001** | 26.661 | 0.376 | 0.464 | 0.025 | 0.019 | 0.196 |
| 17 | 0.0005 | 7 | 0.5 | **0.001** | 26.661 | 0.376 | 0.464 | 0.025 | 0.019 | 0.196 |
| 18 | 0.0005 | 8 | 0.5 | **0.001** | 26.661 | 0.376 | 0.464 | 0.025 | 0.019 | 0.196 |
| 19 | 0.0005 | 9 | 0.5 | **0.001** | 26.661 | 0.376 | 0.464 | 0.025 | 0.019 | 0.196 |
| 20 | 0.0005 | 10 | 0.5 | **0.001** | 26.661 | 0.376 | 0.464 | 0.025 | 0.019 | 0.196 |
| 21 | 0.0001 | 1 | 0.5 | **0.001** | 26.614 | 0.376 | 0.463 | 0.025 | 0.019 | 0.191 |
| 22 | 0.0001 | 2 | 0.5 | **0.001** | 26.614 | 0.376 | 0.463 | 0.025 | 0.019 | 0.191 |
| 23 | 0.0001 | 3 | 0.5 | **0.001** | 26.614 | 0.376 | 0.463 | 0.025 | 0.019 | 0.191 |
| 24 | 0.0001 | 4 | 0.5 | **0.001** | 26.614 | 0.376 | 0.463 | 0.025 | 0.019 | 0.191 |
| 25 | 0.0001 | 5 | 0.5 | **0.001** | 26.614 | 0.376 | 0.463 | 0.025 | 0.019 | 0.191 |
| 26 | 0.0001 | 6 | 0.5 | **0.001** | 26.614 | 0.376 | 0.463 | 0.025 | 0.019 | 0.191 |
| 27 | 0.0001 | 7 | 0.5 | **0.001** | 26.614 | 0.376 | 0.463 | 0.025 | 0.019 | 0.191 |
| 28 | 0.0001 | 8 | 0.5 | **0.001** | 26.614 | 0.376 | 0.463 | 0.025 | 0.019 | 0.191 |
| 29 | 0.0001 | 9 | 0.5 | **0.001** | 26.614 | 0.376 | 0.463 | 0.025 | 0.019 | 0.191 |
| 30 | 0.0001 | 10 | 0.5 | **0.001** | 26.614 | 0.376 | 0.463 | 0.025 | 0.019 | 0.191 |
| 31 | 0.0001 | 2 | 0.55 | **0.001** | 26.341 | 0.374 | 0.466 | 0.025 | 0.019 | 0.191 |
| 32 | 0.0001 | 3 | 0.55 | **0.001** | 26.341 | 0.374 | 0.466 | 0.025 | 0.019 | 0.191 |
| 33 | 0.0001 | 4 | 0.55 | **0.001** | 26.341 | 0.374 | 0.466 | 0.025 | 0.019 | 0.191 |
| 34 | 0.0001 | 5 | 0.55 | **0.001** | 26.341 | 0.374 | 0.466 | 0.025 | 0.019 | 0.191 |
| 35 | 0.0001 | 6 | 0.55 | **0.001** | 26.341 | 0.374 | 0.466 | 0.025 | 0.019 | 0.191 |
| 36 | 0.0001 | 7 | 0.55 | **0.001** | 26.341 | 0.374 | 0.466 | 0.025 | 0.019 | 0.191 |
| 37 | 0.0001 | 8 | 0.55 | **0.001** | 26.341 | 0.374 | 0.466 | 0.025 | 0.019 | 0.191 |
| 38 | 0.0001 | 9 | 0.55 | **0.001** | 26.341 | 0.374 | 0.466 | 0.025 | 0.019 | 0.191 |
| 39 | 0.0001 | 10 | 0.55 | **0.001** | 26.341 | 0.374 | 0.466 | 0.025 | 0.019 | 0.191 |
| 40 | 0.0001 | 1 | 0.55 | **0.001** | 26.341 | 0.374 | 0.464 | 0.025 | 0.019 | 0.19 |
| 41 | 0.0005 | 2 | 0.55 | **0.001** | 26.296 | 0.371 | 0.468 | 0.025 | 0.019 | 0.2 |
| 42 | 0.0005 | 3 | 0.55 | **0.001** | 26.296 | 0.371 | 0.468 | 0.025 | 0.019 | 0.2 |
| 43 | 0.0005 | 4 | 0.55 | **0.001** | 26.296 | 0.371 | 0.468 | 0.025 | 0.019 | 0.2 |
| 44 | 0.0005 | 5 | 0.55 | **0.001** | 26.296 | 0.371 | 0.468 | 0.025 | 0.019 | 0.2 |
| 45 | 0.0005 | 6 | 0.55 | **0.001** | 26.296 | 0.371 | 0.468 | 0.025 | 0.019 | 0.2 |
| 46 | 0.0005 | 7 | 0.55 | **0.001** | 26.296 | 0.371 | 0.468 | 0.025 | 0.019 | 0.2 |
| 47 | 0.0005 | 8 | 0.55 | **0.001** | 26.296 | 0.371 | 0.468 | 0.025 | 0.019 | 0.2 |
| 48 | 0.0005 | 9 | 0.55 | **0.001** | 26.296 | 0.371 | 0.468 | 0.025 | 0.019 | 0.2 |
| 49 | 0.0005 | 10 | 0.55 | **0.001** | 26.296 | 0.371 | 0.468 | 0.025 | 0.019 | 0.2 |
| 50 | 0.0005 | 1 | 0.55 | **0.001** | 26.296 | 0.371 | 0.466 | 0.025 | 0.019 | 0.199 |
| 51 | 0.001 | 2 | 0.55 | **0.001** | 26.24 | 0.371 | 0.468 | 0.025 | 0.019 | 0.199 |
| 52 | 0.001 | 3 | 0.55 | **0.001** | 26.24 | 0.371 | 0.468 | 0.025 | 0.019 | 0.199 |
| 53 | 0.001 | 4 | 0.55 | **0.001** | 26.24 | 0.371 | 0.468 | 0.025 | 0.019 | 0.199 |
| 54 | 0.001 | 5 | 0.55 | **0.001** | 26.24 | 0.371 | 0.468 | 0.025 | 0.019 | 0.199 |
| 55 | 0.001 | 6 | 0.55 | **0.001** | 26.24 | 0.371 | 0.468 | 0.025 | 0.019 | 0.199 |
| 56 | 0.001 | 7 | 0.55 | **0.001** | 26.24 | 0.371 | 0.468 | 0.025 | 0.019 | 0.199 |
| 57 | 0.001 | 8 | 0.55 | **0.001** | 26.24 | 0.371 | 0.468 | 0.025 | 0.019 | 0.199 |
| 58 | 0.001 | 9 | 0.55 | **0.001** | 26.24 | 0.371 | 0.468 | 0.025 | 0.019 | 0.199 |
| 59 | 0.001 | 10 | 0.55 | **0.001** | 26.24 | 0.371 | 0.468 | 0.025 | 0.019 | 0.199 |
| 60 | 0.001 | 1 | 0.55 | **0.001** | 26.24 | 0.371 | 0.466 | 0.025 | 0.019 | 0.198 |
| 61 | 0.0005 | 2 | 0.6 | **0.001** | 26.051 | 0.37 | 0.475 | 0.025 | 0.019 | 0.199 |
| 62 | 0.0005 | 3 | 0.6 | **0.001** | 26.051 | 0.37 | 0.475 | 0.025 | 0.019 | 0.199 |
| 63 | 0.0005 | 4 | 0.6 | **0.001** | 26.051 | 0.37 | 0.475 | 0.025 | 0.019 | 0.199 |
| 64 | 0.0005 | 5 | 0.6 | **0.001** | 26.051 | 0.37 | 0.475 | 0.025 | 0.019 | 0.199 |
| 65 | 0.0005 | 6 | 0.6 | **0.001** | 26.051 | 0.37 | 0.475 | 0.025 | 0.019 | 0.199 |
| 66 | 0.0005 | 7 | 0.6 | **0.001** | 26.051 | 0.37 | 0.475 | 0.025 | 0.019 | 0.199 |
| 67 | 0.0005 | 8 | 0.6 | **0.001** | 26.051 | 0.37 | 0.475 | 0.025 | 0.019 | 0.199 |
| 68 | 0.0005 | 9 | 0.6 | **0.001** | 26.051 | 0.37 | 0.475 | 0.025 | 0.019 | 0.199 |
| 69 | 0.0005 | 10 | 0.6 | **0.001** | 26.051 | 0.37 | 0.475 | 0.025 | 0.019 | 0.199 |
| 70 | 0.0005 | 1 | 0.6 | **0.001** | 26.048 | 0.371 | 0.467 | 0.025 | 0.019 | 0.197 |
| 71 | 0.0001 | 1 | 0.6 | **0.001** | 26.038 | 0.372 | 0.466 | 0.025 | 0.019 | 0.188 |
| 72 | 0.0001 | 2 | 0.6 | **0.001** | 26.02 | 0.371 | 0.475 | 0.025 | 0.019 | 0.195 |
| 73 | 0.0001 | 3 | 0.6 | **0.001** | 26.02 | 0.371 | 0.475 | 0.025 | 0.019 | 0.195 |
| 74 | 0.0001 | 4 | 0.6 | **0.001** | 26.02 | 0.371 | 0.475 | 0.025 | 0.019 | 0.195 |
| 75 | 0.0001 | 5 | 0.6 | **0.001** | 26.02 | 0.371 | 0.475 | 0.025 | 0.019 | 0.195 |
| 76 | 0.0001 | 6 | 0.6 | **0.001** | 26.02 | 0.371 | 0.475 | 0.025 | 0.019 | 0.195 |
| 77 | 0.0001 | 7 | 0.6 | **0.001** | 26.02 | 0.371 | 0.475 | 0.025 | 0.019 | 0.195 |
| 78 | 0.0001 | 8 | 0.6 | **0.001** | 26.02 | 0.371 | 0.475 | 0.025 | 0.019 | 0.195 |
| 79 | 0.0001 | 9 | 0.6 | **0.001** | 26.02 | 0.371 | 0.475 | 0.025 | 0.019 | 0.195 |
| 80 | 0.0001 | 10 | 0.6 | **0.001** | 26.02 | 0.371 | 0.475 | 0.025 | 0.019 | 0.195 |
| 81 | 0.001 | 1 | 0.6 | **0.001** | 25.984 | 0.373 | 0.467 | 0.025 | 0.019 | 0.191 |
| 82 | 0.001 | 2 | 0.6 | **0.001** | 25.978 | 0.371 | 0.477 | 0.025 | 0.019 | 0.199 |
| 83 | 0.001 | 3 | 0.6 | **0.001** | 25.978 | 0.371 | 0.477 | 0.025 | 0.019 | 0.199 |
| 84 | 0.001 | 4 | 0.6 | **0.001** | 25.978 | 0.371 | 0.477 | 0.025 | 0.019 | 0.199 |
| 85 | 0.001 | 5 | 0.6 | **0.001** | 25.978 | 0.371 | 0.477 | 0.025 | 0.019 | 0.199 |
| 86 | 0.001 | 6 | 0.6 | **0.001** | 25.978 | 0.371 | 0.477 | 0.025 | 0.019 | 0.199 |
| 87 | 0.001 | 7 | 0.6 | **0.001** | 25.978 | 0.371 | 0.477 | 0.025 | 0.019 | 0.199 |
| 88 | 0.001 | 8 | 0.6 | **0.001** | 25.978 | 0.371 | 0.477 | 0.025 | 0.019 | 0.199 |
| 89 | 0.001 | 9 | 0.6 | **0.001** | 25.978 | 0.371 | 0.477 | 0.025 | 0.019 | 0.199 |
| 90 | 0.001 | 10 | 0.6 | **0.001** | 25.978 | 0.371 | 0.477 | 0.025 | 0.019 | 0.199 |
| 91 | 0.001 | 1 | 0.65 | **0.001** | 25.945 | 0.371 | 0.467 | 0.025 | 0.019 | 0.191 |
| 92 | 0.0005 | 1 | 0.65 | **0.001** | 25.93 | 0.37 | 0.467 | 0.025 | 0.019 | 0.191 |
| 93 | 0.0001 | 1 | 0.65 | **0.001** | 25.883 | 0.369 | 0.466 | 0.025 | 0.019 | 0.188 |
| 94 | 0.0005 | 2 | 0.65 | **0.001** | 25.804 | 0.365 | 0.479 | 0.025 | 0.019 | 0.196 |
| 95 | 0.0005 | 3 | 0.65 | **0.001** | 25.804 | 0.365 | 0.479 | 0.025 | 0.019 | 0.196 |
| 96 | 0.0005 | 4 | 0.65 | **0.001** | 25.804 | 0.365 | 0.479 | 0.025 | 0.019 | 0.196 |
| 97 | 0.0005 | 5 | 0.65 | **0.001** | 25.804 | 0.365 | 0.479 | 0.025 | 0.019 | 0.196 |
| 98 | 0.0005 | 6 | 0.65 | **0.001** | 25.804 | 0.365 | 0.479 | 0.025 | 0.019 | 0.196 |
| 99 | 0.0005 | 7 | 0.65 | **0.001** | 25.804 | 0.365 | 0.479 | 0.025 | 0.019 | 0.196 |
| 100 | 0.0005 | 8 | 0.65 | **0.001** | 25.804 | 0.365 | 0.479 | 0.025 | 0.019 | 0.196 |
| 101 | 0.0005 | 9 | 0.65 | **0.001** | 25.804 | 0.365 | 0.479 | 0.025 | 0.019 | 0.196 |
| 102 | 0.0005 | 10 | 0.65 | **0.001** | 25.804 | 0.365 | 0.479 | 0.025 | 0.019 | 0.196 |
| 103 | 0.001 | 2 | 0.65 | **0.001** | 25.771 | 0.365 | 0.481 | 0.025 | 0.019 | 0.2 |
| 104 | 0.001 | 3 | 0.65 | **0.001** | 25.771 | 0.365 | 0.481 | 0.025 | 0.019 | 0.2 |
| 105 | 0.001 | 4 | 0.65 | **0.001** | 25.771 | 0.365 | 0.481 | 0.025 | 0.019 | 0.2 |
| 106 | 0.001 | 5 | 0.65 | **0.001** | 25.771 | 0.365 | 0.481 | 0.025 | 0.019 | 0.2 |
| 107 | 0.001 | 6 | 0.65 | **0.001** | 25.771 | 0.365 | 0.481 | 0.025 | 0.019 | 0.2 |
| 108 | 0.001 | 7 | 0.65 | **0.001** | 25.771 | 0.365 | 0.481 | 0.025 | 0.019 | 0.2 |
| 109 | 0.001 | 8 | 0.65 | **0.001** | 25.771 | 0.365 | 0.481 | 0.025 | 0.019 | 0.2 |
| 110 | 0.001 | 9 | 0.65 | **0.001** | 25.771 | 0.365 | 0.481 | 0.025 | 0.019 | 0.2 |
| 111 | 0.001 | 10 | 0.65 | **0.001** | 25.771 | 0.365 | 0.481 | 0.025 | 0.019 | 0.2 |
| 112 | 0.0005 | 1 | 0.7 | **0.001** | 25.755 | 0.367 | 0.467 | 0.025 | 0.019 | 0.191 |
| 113 | 0.0001 | 1 | 0.7 | **0.001** | 25.752 | 0.368 | 0.466 | 0.025 | 0.019 | 0.187 |
| 114 | 0.0001 | 2 | 0.65 | **0.001** | 25.732 | 0.364 | 0.48 | 0.025 | 0.019 | 0.197 |
| 115 | 0.0001 | 3 | 0.65 | **0.001** | 25.732 | 0.364 | 0.48 | 0.025 | 0.019 | 0.197 |
| 116 | 0.0001 | 4 | 0.65 | **0.001** | 25.732 | 0.364 | 0.48 | 0.025 | 0.019 | 0.197 |
| 117 | 0.0001 | 5 | 0.65 | **0.001** | 25.732 | 0.364 | 0.48 | 0.025 | 0.019 | 0.197 |
| 118 | 0.0001 | 6 | 0.65 | **0.001** | 25.732 | 0.364 | 0.48 | 0.025 | 0.019 | 0.197 |
| 119 | 0.0001 | 7 | 0.65 | **0.001** | 25.732 | 0.364 | 0.48 | 0.025 | 0.019 | 0.197 |
| 120 | 0.0001 | 8 | 0.65 | **0.001** | 25.732 | 0.364 | 0.48 | 0.025 | 0.019 | 0.197 |
| 121 | 0.0001 | 9 | 0.65 | **0.001** | 25.732 | 0.364 | 0.48 | 0.025 | 0.019 | 0.197 |
| 122 | 0.0001 | 10 | 0.65 | **0.001** | 25.732 | 0.364 | 0.48 | 0.025 | 0.019 | 0.197 |
| 123 | 0.001 | 1 | 0.7 | **0.001** | 25.712 | 0.367 | 0.467 | 0.025 | 0.019 | 0.192 |
| 124 | 0.0001 | 1 | 0.75 | **0.001** | 25.637 | 0.367 | 0.465 | 0.025 | 0.019 | 0.186 |
| 125 | 0.0005 | 1 | 0.75 | **0.001** | 25.636 | 0.366 | 0.467 | 0.025 | 0.019 | 0.191 |
| 126 | 0.0005 | 2 | 0.7 | **0.001** | 25.579 | 0.36 | 0.483 | 0.025 | 0.019 | 0.199 |
| 127 | 0.0005 | 3 | 0.7 | **0.001** | 25.579 | 0.36 | 0.483 | 0.025 | 0.019 | 0.199 |
| 128 | 0.0005 | 4 | 0.7 | **0.001** | 25.579 | 0.36 | 0.483 | 0.025 | 0.019 | 0.199 |
| 129 | 0.0005 | 5 | 0.7 | **0.001** | 25.579 | 0.36 | 0.483 | 0.025 | 0.019 | 0.199 |
| 130 | 0.0005 | 6 | 0.7 | **0.001** | 25.579 | 0.36 | 0.483 | 0.025 | 0.019 | 0.199 |
| 131 | 0.0005 | 7 | 0.7 | **0.001** | 25.579 | 0.36 | 0.483 | 0.025 | 0.019 | 0.199 |
| 132 | 0.0005 | 8 | 0.7 | **0.001** | 25.579 | 0.36 | 0.483 | 0.025 | 0.019 | 0.199 |
| 133 | 0.0005 | 9 | 0.7 | **0.001** | 25.579 | 0.36 | 0.483 | 0.025 | 0.019 | 0.199 |
| 134 | 0.0005 | 10 | 0.7 | **0.001** | 25.579 | 0.36 | 0.483 | 0.025 | 0.019 | 0.199 |
| 135 | 0.001 | 1 | 0.75 | **0.001** | 25.578 | 0.365 | 0.467 | 0.025 | 0.019 | 0.191 |
| 136 | 0.001 | 2 | 0.7 | **0.001** | 25.574 | 0.359 | 0.484 | 0.025 | 0.019 | 0.202 |
| 137 | 0.001 | 3 | 0.7 | **0.001** | 25.574 | 0.359 | 0.484 | 0.025 | 0.019 | 0.202 |
| 138 | 0.001 | 4 | 0.7 | **0.001** | 25.574 | 0.359 | 0.484 | 0.025 | 0.019 | 0.202 |
| 139 | 0.001 | 5 | 0.7 | **0.001** | 25.574 | 0.359 | 0.484 | 0.025 | 0.019 | 0.202 |
| 140 | 0.001 | 6 | 0.7 | **0.001** | 25.574 | 0.359 | 0.484 | 0.025 | 0.019 | 0.202 |
| 141 | 0.001 | 7 | 0.7 | **0.001** | 25.574 | 0.359 | 0.484 | 0.025 | 0.019 | 0.202 |
| 142 | 0.001 | 8 | 0.7 | **0.001** | 25.574 | 0.359 | 0.484 | 0.025 | 0.019 | 0.202 |
| 143 | 0.001 | 9 | 0.7 | **0.001** | 25.574 | 0.359 | 0.484 | 0.025 | 0.019 | 0.202 |
| 144 | 0.001 | 10 | 0.7 | **0.001** | 25.574 | 0.359 | 0.484 | 0.025 | 0.019 | 0.202 |
| 145 | 0.0001 | 2 | 0.7 | **0.001** | 25.524 | 0.361 | 0.483 | 0.025 | 0.019 | 0.197 |
| 146 | 0.0001 | 3 | 0.7 | **0.001** | 25.524 | 0.361 | 0.483 | 0.025 | 0.019 | 0.197 |
| 147 | 0.0001 | 4 | 0.7 | **0.001** | 25.524 | 0.361 | 0.483 | 0.025 | 0.019 | 0.197 |
| 148 | 0.0001 | 5 | 0.7 | **0.001** | 25.524 | 0.361 | 0.483 | 0.025 | 0.019 | 0.197 |
| 149 | 0.0001 | 6 | 0.7 | **0.001** | 25.524 | 0.361 | 0.483 | 0.025 | 0.019 | 0.197 |
| 150 | 0.0001 | 7 | 0.7 | **0.001** | 25.524 | 0.361 | 0.483 | 0.025 | 0.019 | 0.197 |
| 151 | 0.0001 | 8 | 0.7 | **0.001** | 25.524 | 0.361 | 0.483 | 0.025 | 0.019 | 0.197 |
| 152 | 0.0001 | 9 | 0.7 | **0.001** | 25.524 | 0.361 | 0.483 | 0.025 | 0.019 | 0.197 |
| 153 | 0.0001 | 10 | 0.7 | **0.001** | 25.524 | 0.361 | 0.483 | 0.025 | 0.019 | 0.197 |
| 154 | 0.0005 | 3 | 0.75 | **0.001** | 25.351 | 0.356 | 0.488 | 0.025 | 0.019 | 0.203 |
| 155 | 0.0005 | 4 | 0.75 | **0.001** | 25.351 | 0.356 | 0.488 | 0.025 | 0.019 | 0.203 |
| 156 | 0.0005 | 5 | 0.75 | **0.001** | 25.351 | 0.356 | 0.488 | 0.025 | 0.019 | 0.203 |
| 157 | 0.0005 | 6 | 0.75 | **0.001** | 25.351 | 0.356 | 0.488 | 0.025 | 0.019 | 0.203 |
| 158 | 0.0005 | 7 | 0.75 | **0.001** | 25.351 | 0.356 | 0.488 | 0.025 | 0.019 | 0.203 |
| 159 | 0.0005 | 8 | 0.75 | **0.001** | 25.351 | 0.356 | 0.488 | 0.025 | 0.019 | 0.203 |
| 160 | 0.0005 | 9 | 0.75 | **0.001** | 25.351 | 0.356 | 0.488 | 0.025 | 0.019 | 0.203 |
| 161 | 0.0005 | 10 | 0.75 | **0.001** | 25.351 | 0.356 | 0.488 | 0.025 | 0.019 | 0.203 |
| 162 | 0.0005 | 2 | 0.75 | **0.001** | 25.351 | 0.356 | 0.487 | 0.025 | 0.019 | 0.202 |
| 163 | 0.0001 | 3 | 0.75 | **0.001** | 25.332 | 0.357 | 0.485 | 0.025 | 0.019 | 0.198 |
| 164 | 0.0001 | 4 | 0.75 | **0.001** | 25.332 | 0.357 | 0.485 | 0.025 | 0.019 | 0.198 |
| 165 | 0.0001 | 5 | 0.75 | **0.001** | 25.332 | 0.357 | 0.485 | 0.025 | 0.019 | 0.198 |
| 166 | 0.0001 | 6 | 0.75 | **0.001** | 25.332 | 0.357 | 0.485 | 0.025 | 0.019 | 0.198 |
| 167 | 0.0001 | 7 | 0.75 | **0.001** | 25.332 | 0.357 | 0.485 | 0.025 | 0.019 | 0.198 |
| 168 | 0.0001 | 8 | 0.75 | **0.001** | 25.332 | 0.357 | 0.485 | 0.025 | 0.019 | 0.198 |
| 169 | 0.0001 | 9 | 0.75 | **0.001** | 25.332 | 0.357 | 0.485 | 0.025 | 0.019 | 0.198 |
| 170 | 0.0001 | 10 | 0.75 | **0.001** | 25.332 | 0.357 | 0.485 | 0.025 | 0.019 | 0.198 |
| 171 | 0.0001 | 2 | 0.75 | **0.001** | 25.332 | 0.357 | 0.485 | 0.025 | 0.019 | 0.197 |
| 172 | 0.001 | 3 | 0.75 | **0.001** | 25.282 | 0.356 | 0.487 | 0.025 | 0.019 | 0.205 |
| 173 | 0.001 | 4 | 0.75 | **0.001** | 25.282 | 0.356 | 0.487 | 0.025 | 0.019 | 0.205 |
| 174 | 0.001 | 5 | 0.75 | **0.001** | 25.282 | 0.356 | 0.487 | 0.025 | 0.019 | 0.205 |
| 175 | 0.001 | 6 | 0.75 | **0.001** | 25.282 | 0.356 | 0.487 | 0.025 | 0.019 | 0.205 |
| 176 | 0.001 | 7 | 0.75 | **0.001** | 25.282 | 0.356 | 0.487 | 0.025 | 0.019 | 0.205 |
| 177 | 0.001 | 8 | 0.75 | **0.001** | 25.282 | 0.356 | 0.487 | 0.025 | 0.019 | 0.205 |
| 178 | 0.001 | 9 | 0.75 | **0.001** | 25.282 | 0.356 | 0.487 | 0.025 | 0.019 | 0.205 |
| 179 | 0.001 | 10 | 0.75 | **0.001** | 25.282 | 0.356 | 0.487 | 0.025 | 0.019 | 0.205 |
| 180 | 0.001 | 2 | 0.75 | **0.001** | 25.282 | 0.356 | 0.486 | 0.025 | 0.019 | 0.204 |
| 181 | 0.005 | 1 | 0.5 | **NA** | NA | NA | NA | NA | NA | NA |
| 182 | 0.01 | 1 | 0.5 | **NA** | NA | NA | NA | NA | NA | NA |
| 183 | 0.05 | 1 | 0.5 | **NA** | NA | NA | NA | NA | NA | NA |
| 184 | 0.005 | 2 | 0.5 | **NA** | NA | NA | NA | NA | NA | NA |
| 185 | 0.01 | 2 | 0.5 | **NA** | NA | NA | NA | NA | NA | NA |
| 186 | 0.05 | 2 | 0.5 | **NA** | NA | NA | NA | NA | NA | NA |
| 187 | 0.005 | 3 | 0.5 | **NA** | NA | NA | NA | NA | NA | NA |
| 188 | 0.01 | 3 | 0.5 | **NA** | NA | NA | NA | NA | NA | NA |
| 189 | 0.05 | 3 | 0.5 | **NA** | NA | NA | NA | NA | NA | NA |
| 190 | 0.005 | 4 | 0.5 | **NA** | NA | NA | NA | NA | NA | NA |
| 191 | 0.01 | 4 | 0.5 | **NA** | NA | NA | NA | NA | NA | NA |
| 192 | 0.05 | 4 | 0.5 | **NA** | NA | NA | NA | NA | NA | NA |
| 193 | 0.005 | 5 | 0.5 | **NA** | NA | NA | NA | NA | NA | NA |
| 194 | 0.01 | 5 | 0.5 | **NA** | NA | NA | NA | NA | NA | NA |
| 195 | 0.05 | 5 | 0.5 | **NA** | NA | NA | NA | NA | NA | NA |
| 196 | 0.005 | 6 | 0.5 | **NA** | NA | NA | NA | NA | NA | NA |
| 197 | 0.01 | 6 | 0.5 | **NA** | NA | NA | NA | NA | NA | NA |
| 198 | 0.05 | 6 | 0.5 | **NA** | NA | NA | NA | NA | NA | NA |
| 199 | 0.005 | 7 | 0.5 | **NA** | NA | NA | NA | NA | NA | NA |
| 200 | 0.01 | 7 | 0.5 | **NA** | NA | NA | NA | NA | NA | NA |
| 201 | 0.05 | 7 | 0.5 | **NA** | NA | NA | NA | NA | NA | NA |
| 202 | 0.005 | 8 | 0.5 | **NA** | NA | NA | NA | NA | NA | NA |
| 203 | 0.01 | 8 | 0.5 | **NA** | NA | NA | NA | NA | NA | NA |
| 204 | 0.05 | 8 | 0.5 | **NA** | NA | NA | NA | NA | NA | NA |
| 205 | 0.005 | 9 | 0.5 | **NA** | NA | NA | NA | NA | NA | NA |
| 206 | 0.01 | 9 | 0.5 | **NA** | NA | NA | NA | NA | NA | NA |
| 207 | 0.05 | 9 | 0.5 | **NA** | NA | NA | NA | NA | NA | NA |
| 208 | 0.005 | 10 | 0.5 | **NA** | NA | NA | NA | NA | NA | NA |
| 209 | 0.01 | 10 | 0.5 | **NA** | NA | NA | NA | NA | NA | NA |
| 210 | 0.05 | 10 | 0.5 | **NA** | NA | NA | NA | NA | NA | NA |
| 211 | 0.005 | 1 | 0.55 | **NA** | NA | NA | NA | NA | NA | NA |
| 212 | 0.01 | 1 | 0.55 | **NA** | NA | NA | NA | NA | NA | NA |
| 213 | 0.05 | 1 | 0.55 | **NA** | NA | NA | NA | NA | NA | NA |
| 214 | 0.005 | 2 | 0.55 | **NA** | NA | NA | NA | NA | NA | NA |
| 215 | 0.01 | 2 | 0.55 | **NA** | NA | NA | NA | NA | NA | NA |
| 216 | 0.05 | 2 | 0.55 | **NA** | NA | NA | NA | NA | NA | NA |
| 217 | 0.005 | 3 | 0.55 | **NA** | NA | NA | NA | NA | NA | NA |
| 218 | 0.01 | 3 | 0.55 | **NA** | NA | NA | NA | NA | NA | NA |
| 219 | 0.05 | 3 | 0.55 | **NA** | NA | NA | NA | NA | NA | NA |
| 220 | 0.005 | 4 | 0.55 | **NA** | NA | NA | NA | NA | NA | NA |
| 221 | 0.01 | 4 | 0.55 | **NA** | NA | NA | NA | NA | NA | NA |
| 222 | 0.05 | 4 | 0.55 | **NA** | NA | NA | NA | NA | NA | NA |
| 223 | 0.005 | 5 | 0.55 | **NA** | NA | NA | NA | NA | NA | NA |
| 224 | 0.01 | 5 | 0.55 | **NA** | NA | NA | NA | NA | NA | NA |
| 225 | 0.05 | 5 | 0.55 | **NA** | NA | NA | NA | NA | NA | NA |
| 226 | 0.005 | 6 | 0.55 | **NA** | NA | NA | NA | NA | NA | NA |
| 227 | 0.01 | 6 | 0.55 | **NA** | NA | NA | NA | NA | NA | NA |
| 228 | 0.05 | 6 | 0.55 | **NA** | NA | NA | NA | NA | NA | NA |
| 229 | 0.005 | 7 | 0.55 | **NA** | NA | NA | NA | NA | NA | NA |
| 230 | 0.01 | 7 | 0.55 | **NA** | NA | NA | NA | NA | NA | NA |
| 231 | 0.05 | 7 | 0.55 | **NA** | NA | NA | NA | NA | NA | NA |
| 232 | 0.005 | 8 | 0.55 | **NA** | NA | NA | NA | NA | NA | NA |
| 233 | 0.01 | 8 | 0.55 | **NA** | NA | NA | NA | NA | NA | NA |
| 234 | 0.05 | 8 | 0.55 | **NA** | NA | NA | NA | NA | NA | NA |
| 235 | 0.005 | 9 | 0.55 | **NA** | NA | NA | NA | NA | NA | NA |
| 236 | 0.01 | 9 | 0.55 | **NA** | NA | NA | NA | NA | NA | NA |
| 237 | 0.05 | 9 | 0.55 | **NA** | NA | NA | NA | NA | NA | NA |
| 238 | 0.005 | 10 | 0.55 | **NA** | NA | NA | NA | NA | NA | NA |
| 239 | 0.01 | 10 | 0.55 | **NA** | NA | NA | NA | NA | NA | NA |
| 240 | 0.05 | 10 | 0.55 | **NA** | NA | NA | NA | NA | NA | NA |
| 241 | 0.005 | 1 | 0.6 | **NA** | NA | NA | NA | NA | NA | NA |
| 242 | 0.01 | 1 | 0.6 | **NA** | NA | NA | NA | NA | NA | NA |
| 243 | 0.05 | 1 | 0.6 | **NA** | NA | NA | NA | NA | NA | NA |
| 244 | 0.005 | 2 | 0.6 | **NA** | NA | NA | NA | NA | NA | NA |
| 245 | 0.01 | 2 | 0.6 | **NA** | NA | NA | NA | NA | NA | NA |
| 246 | 0.05 | 2 | 0.6 | **NA** | NA | NA | NA | NA | NA | NA |
| 247 | 0.005 | 3 | 0.6 | **NA** | NA | NA | NA | NA | NA | NA |
| 248 | 0.01 | 3 | 0.6 | **NA** | NA | NA | NA | NA | NA | NA |
| 249 | 0.05 | 3 | 0.6 | **NA** | NA | NA | NA | NA | NA | NA |
| 250 | 0.005 | 4 | 0.6 | **NA** | NA | NA | NA | NA | NA | NA |
| 251 | 0.01 | 4 | 0.6 | **NA** | NA | NA | NA | NA | NA | NA |
| 252 | 0.05 | 4 | 0.6 | **NA** | NA | NA | NA | NA | NA | NA |
| 253 | 0.005 | 5 | 0.6 | **NA** | NA | NA | NA | NA | NA | NA |
| 254 | 0.01 | 5 | 0.6 | **NA** | NA | NA | NA | NA | NA | NA |
| 255 | 0.05 | 5 | 0.6 | **NA** | NA | NA | NA | NA | NA | NA |
| 256 | 0.005 | 6 | 0.6 | **NA** | NA | NA | NA | NA | NA | NA |
| 257 | 0.01 | 6 | 0.6 | **NA** | NA | NA | NA | NA | NA | NA |
| 258 | 0.05 | 6 | 0.6 | **NA** | NA | NA | NA | NA | NA | NA |
| 259 | 0.005 | 7 | 0.6 | **NA** | NA | NA | NA | NA | NA | NA |
| 260 | 0.01 | 7 | 0.6 | **NA** | NA | NA | NA | NA | NA | NA |
| 261 | 0.05 | 7 | 0.6 | **NA** | NA | NA | NA | NA | NA | NA |
| 262 | 0.005 | 8 | 0.6 | **NA** | NA | NA | NA | NA | NA | NA |
| 263 | 0.01 | 8 | 0.6 | **NA** | NA | NA | NA | NA | NA | NA |
| 264 | 0.05 | 8 | 0.6 | **NA** | NA | NA | NA | NA | NA | NA |
| 265 | 0.005 | 9 | 0.6 | **NA** | NA | NA | NA | NA | NA | NA |
| 266 | 0.01 | 9 | 0.6 | **NA** | NA | NA | NA | NA | NA | NA |
| 267 | 0.05 | 9 | 0.6 | **NA** | NA | NA | NA | NA | NA | NA |
| 268 | 0.005 | 10 | 0.6 | **NA** | NA | NA | NA | NA | NA | NA |
| 269 | 0.01 | 10 | 0.6 | **NA** | NA | NA | NA | NA | NA | NA |
| 270 | 0.05 | 10 | 0.6 | **NA** | NA | NA | NA | NA | NA | NA |
| 271 | 0.005 | 1 | 0.65 | **NA** | NA | NA | NA | NA | NA | NA |
| 272 | 0.01 | 1 | 0.65 | **NA** | NA | NA | NA | NA | NA | NA |
| 273 | 0.05 | 1 | 0.65 | **NA** | NA | NA | NA | NA | NA | NA |
| 274 | 0.005 | 2 | 0.65 | **NA** | NA | NA | NA | NA | NA | NA |
| 275 | 0.01 | 2 | 0.65 | **NA** | NA | NA | NA | NA | NA | NA |
| 276 | 0.05 | 2 | 0.65 | **NA** | NA | NA | NA | NA | NA | NA |
| 277 | 0.005 | 3 | 0.65 | **NA** | NA | NA | NA | NA | NA | NA |
| 278 | 0.01 | 3 | 0.65 | **NA** | NA | NA | NA | NA | NA | NA |
| 279 | 0.05 | 3 | 0.65 | **NA** | NA | NA | NA | NA | NA | NA |
| 280 | 0.005 | 4 | 0.65 | **NA** | NA | NA | NA | NA | NA | NA |
| 281 | 0.01 | 4 | 0.65 | **NA** | NA | NA | NA | NA | NA | NA |
| 282 | 0.05 | 4 | 0.65 | **NA** | NA | NA | NA | NA | NA | NA |
| 283 | 0.005 | 5 | 0.65 | **NA** | NA | NA | NA | NA | NA | NA |
| 284 | 0.01 | 5 | 0.65 | **NA** | NA | NA | NA | NA | NA | NA |
| 285 | 0.05 | 5 | 0.65 | **NA** | NA | NA | NA | NA | NA | NA |
| 286 | 0.005 | 6 | 0.65 | **NA** | NA | NA | NA | NA | NA | NA |
| 287 | 0.01 | 6 | 0.65 | **NA** | NA | NA | NA | NA | NA | NA |
| 288 | 0.05 | 6 | 0.65 | **NA** | NA | NA | NA | NA | NA | NA |
| 289 | 0.005 | 7 | 0.65 | **NA** | NA | NA | NA | NA | NA | NA |
| 290 | 0.01 | 7 | 0.65 | **NA** | NA | NA | NA | NA | NA | NA |
| 291 | 0.05 | 7 | 0.65 | **NA** | NA | NA | NA | NA | NA | NA |
| 292 | 0.005 | 8 | 0.65 | **NA** | NA | NA | NA | NA | NA | NA |
| 293 | 0.01 | 8 | 0.65 | **NA** | NA | NA | NA | NA | NA | NA |
| 294 | 0.05 | 8 | 0.65 | **NA** | NA | NA | NA | NA | NA | NA |
| 295 | 0.005 | 9 | 0.65 | **NA** | NA | NA | NA | NA | NA | NA |
| 296 | 0.01 | 9 | 0.65 | **NA** | NA | NA | NA | NA | NA | NA |
| 297 | 0.05 | 9 | 0.65 | **NA** | NA | NA | NA | NA | NA | NA |
| 298 | 0.005 | 10 | 0.65 | **NA** | NA | NA | NA | NA | NA | NA |
| 299 | 0.01 | 10 | 0.65 | **NA** | NA | NA | NA | NA | NA | NA |
| 300 | 0.05 | 10 | 0.65 | **NA** | NA | NA | NA | NA | NA | NA |
| 301 | 0.005 | 1 | 0.7 | **NA** | NA | NA | NA | NA | NA | NA |
| 302 | 0.01 | 1 | 0.7 | **NA** | NA | NA | NA | NA | NA | NA |
| 303 | 0.05 | 1 | 0.7 | **NA** | NA | NA | NA | NA | NA | NA |
| 304 | 0.005 | 2 | 0.7 | **NA** | NA | NA | NA | NA | NA | NA |
| 305 | 0.01 | 2 | 0.7 | **NA** | NA | NA | NA | NA | NA | NA |
| 306 | 0.05 | 2 | 0.7 | **NA** | NA | NA | NA | NA | NA | NA |
| 307 | 0.005 | 3 | 0.7 | **NA** | NA | NA | NA | NA | NA | NA |
| 308 | 0.01 | 3 | 0.7 | **NA** | NA | NA | NA | NA | NA | NA |
| 309 | 0.05 | 3 | 0.7 | **NA** | NA | NA | NA | NA | NA | NA |
| 310 | 0.005 | 4 | 0.7 | **NA** | NA | NA | NA | NA | NA | NA |
| 311 | 0.01 | 4 | 0.7 | **NA** | NA | NA | NA | NA | NA | NA |
| 312 | 0.05 | 4 | 0.7 | **NA** | NA | NA | NA | NA | NA | NA |
| 313 | 0.005 | 5 | 0.7 | **NA** | NA | NA | NA | NA | NA | NA |
| 314 | 0.01 | 5 | 0.7 | **NA** | NA | NA | NA | NA | NA | NA |
| 315 | 0.05 | 5 | 0.7 | **NA** | NA | NA | NA | NA | NA | NA |
| 316 | 0.005 | 6 | 0.7 | **NA** | NA | NA | NA | NA | NA | NA |
| 317 | 0.01 | 6 | 0.7 | **NA** | NA | NA | NA | NA | NA | NA |
| 318 | 0.05 | 6 | 0.7 | **NA** | NA | NA | NA | NA | NA | NA |
| 319 | 0.005 | 7 | 0.7 | **NA** | NA | NA | NA | NA | NA | NA |
| 320 | 0.01 | 7 | 0.7 | **NA** | NA | NA | NA | NA | NA | NA |
| 321 | 0.05 | 7 | 0.7 | **NA** | NA | NA | NA | NA | NA | NA |
| 322 | 0.005 | 8 | 0.7 | **NA** | NA | NA | NA | NA | NA | NA |
| 323 | 0.01 | 8 | 0.7 | **NA** | NA | NA | NA | NA | NA | NA |
| 324 | 0.05 | 8 | 0.7 | **NA** | NA | NA | NA | NA | NA | NA |
| 325 | 0.005 | 9 | 0.7 | **NA** | NA | NA | NA | NA | NA | NA |
| 326 | 0.01 | 9 | 0.7 | **NA** | NA | NA | NA | NA | NA | NA |
| 327 | 0.05 | 9 | 0.7 | **NA** | NA | NA | NA | NA | NA | NA |
| 328 | 0.005 | 10 | 0.7 | **NA** | NA | NA | NA | NA | NA | NA |
| 329 | 0.01 | 10 | 0.7 | **NA** | NA | NA | NA | NA | NA | NA |
| 330 | 0.05 | 10 | 0.7 | **NA** | NA | NA | NA | NA | NA | NA |
| 331 | 0.005 | 1 | 0.75 | **NA** | NA | NA | NA | NA | NA | NA |
| 332 | 0.01 | 1 | 0.75 | **NA** | NA | NA | NA | NA | NA | NA |
| 333 | 0.05 | 1 | 0.75 | **NA** | NA | NA | NA | NA | NA | NA |
| 334 | 0.005 | 2 | 0.75 | **NA** | NA | NA | NA | NA | NA | NA |
| 335 | 0.01 | 2 | 0.75 | **NA** | NA | NA | NA | NA | NA | NA |
| 336 | 0.05 | 2 | 0.75 | **NA** | NA | NA | NA | NA | NA | NA |
| 337 | 0.005 | 3 | 0.75 | **NA** | NA | NA | NA | NA | NA | NA |
| 338 | 0.01 | 3 | 0.75 | **NA** | NA | NA | NA | NA | NA | NA |
| 339 | 0.05 | 3 | 0.75 | **NA** | NA | NA | NA | NA | NA | NA |
| 340 | 0.005 | 4 | 0.75 | **NA** | NA | NA | NA | NA | NA | NA |
| 341 | 0.01 | 4 | 0.75 | **NA** | NA | NA | NA | NA | NA | NA |
| 342 | 0.05 | 4 | 0.75 | **NA** | NA | NA | NA | NA | NA | NA |
| 343 | 0.005 | 5 | 0.75 | **NA** | NA | NA | NA | NA | NA | NA |
| 344 | 0.01 | 5 | 0.75 | **NA** | NA | NA | NA | NA | NA | NA |
| 345 | 0.05 | 5 | 0.75 | **NA** | NA | NA | NA | NA | NA | NA |
| 346 | 0.005 | 6 | 0.75 | **NA** | NA | NA | NA | NA | NA | NA |
| 347 | 0.01 | 6 | 0.75 | **NA** | NA | NA | NA | NA | NA | NA |
| 348 | 0.05 | 6 | 0.75 | **NA** | NA | NA | NA | NA | NA | NA |
| 349 | 0.005 | 7 | 0.75 | **NA** | NA | NA | NA | NA | NA | NA |
| 350 | 0.01 | 7 | 0.75 | **NA** | NA | NA | NA | NA | NA | NA |
| 351 | 0.05 | 7 | 0.75 | **NA** | NA | NA | NA | NA | NA | NA |
| 352 | 0.005 | 8 | 0.75 | **NA** | NA | NA | NA | NA | NA | NA |
| 353 | 0.01 | 8 | 0.75 | **NA** | NA | NA | NA | NA | NA | NA |
| 354 | 0.05 | 8 | 0.75 | **NA** | NA | NA | NA | NA | NA | NA |
| 355 | 0.005 | 9 | 0.75 | **NA** | NA | NA | NA | NA | NA | NA |
| 356 | 0.01 | 9 | 0.75 | **NA** | NA | NA | NA | NA | NA | NA |
| 357 | 0.05 | 9 | 0.75 | **NA** | NA | NA | NA | NA | NA | NA |
| 358 | 0.005 | 10 | 0.75 | **NA** | NA | NA | NA | NA | NA | NA |
| 359 | 0.01 | 10 | 0.75 | **NA** | NA | NA | NA | NA | NA | NA |
| 360 | 0.05 | 10 | 0.75 | **NA** | NA | NA | NA | NA | NA | NA |

**Table S5.** Possible combinations of the three meta-parameters (learning rate, tree complexity, bag fraction) for fitted 360 BRT models for species richness at 0.0001 m^2^ spatial scale based on 10-fold cross validation (CV) and the associated model performance parameters. Numbers are sorted according to increasing CV deviance (in bold). RMSE represents root mean square error, and MAE represents mean absolute error.

| No. | Learning rate | Tree complexity | Bag fraction | **CV deviance** | Percentage of explained deviance (%) | CV correlation | Training data correlation | RMSE  (%) | MAE  (%) | *R*^2^ |
| --- | --- | --- | --- | --- | --- | --- | --- | --- | --- | --- |
| 1 | 0.001 | 2 | 0.75 | **0.765** | 9.622 | 0.458 | 0.637 | 0.746 | 0.602 | 0.242 |
| 2 | 0.001 | 3 | 0.75 | **0.765** | 9.622 | 0.458 | 0.641 | 0.743 | 0.600 | 0.246 |
| 3 | 0.001 | 4 | 0.75 | **0.765** | 9.622 | 0.458 | 0.641 | 0.743 | 0.600 | 0.246 |
| 4 | 0.001 | 5 | 0.75 | **0.765** | 9.622 | 0.458 | 0.641 | 0.743 | 0.600 | 0.246 |
| 5 | 0.001 | 6 | 0.75 | **0.765** | 9.622 | 0.458 | 0.641 | 0.743 | 0.600 | 0.246 |
| 6 | 0.001 | 7 | 0.75 | **0.765** | 9.622 | 0.458 | 0.641 | 0.743 | 0.600 | 0.246 |
| 7 | 0.001 | 8 | 0.75 | **0.765** | 9.622 | 0.458 | 0.641 | 0.743 | 0.600 | 0.246 |
| 8 | 0.001 | 9 | 0.75 | **0.765** | 9.622 | 0.458 | 0.641 | 0.743 | 0.600 | 0.246 |
| 9 | 0.001 | 10 | 0.75 | **0.765** | 9.622 | 0.458 | 0.641 | 0.743 | 0.600 | 0.246 |
| 10 | 0.001 | 2 | 0.70 | **0.766** | 9.466 | 0.460 | 0.634 | 0.749 | 0.605 | 0.238 |
| 11 | 0.001 | 3 | 0.70 | **0.766** | 9.466 | 0.460 | 0.634 | 0.749 | 0.605 | 0.238 |
| 12 | 0.001 | 4 | 0.70 | **0.766** | 9.466 | 0.460 | 0.634 | 0.749 | 0.605 | 0.238 |
| 13 | 0.001 | 5 | 0.70 | **0.766** | 9.466 | 0.460 | 0.634 | 0.749 | 0.605 | 0.238 |
| 14 | 0.001 | 6 | 0.70 | **0.766** | 9.466 | 0.460 | 0.634 | 0.749 | 0.605 | 0.238 |
| 15 | 0.001 | 7 | 0.70 | **0.766** | 9.466 | 0.460 | 0.634 | 0.749 | 0.605 | 0.238 |
| 16 | 0.001 | 8 | 0.70 | **0.766** | 9.466 | 0.460 | 0.634 | 0.749 | 0.605 | 0.238 |
| 17 | 0.001 | 9 | 0.70 | **0.766** | 9.466 | 0.460 | 0.634 | 0.749 | 0.605 | 0.238 |
| 18 | 0.001 | 10 | 0.70 | **0.766** | 9.466 | 0.460 | 0.634 | 0.749 | 0.605 | 0.238 |
| 19 | 0.005 | 2 | 0.75 | **0.766** | 9.448 | 0.455 | 0.649 | 0.734 | 0.594 | 0.257 |
| 20 | 0.005 | 3 | 0.75 | **0.766** | 9.448 | 0.455 | 0.653 | 0.731 | 0.592 | 0.260 |
| 21 | 0.005 | 4 | 0.75 | **0.766** | 9.448 | 0.455 | 0.653 | 0.731 | 0.592 | 0.260 |
| 22 | 0.005 | 5 | 0.75 | **0.766** | 9.448 | 0.455 | 0.653 | 0.731 | 0.592 | 0.260 |
| 23 | 0.005 | 6 | 0.75 | **0.766** | 9.448 | 0.455 | 0.653 | 0.731 | 0.592 | 0.260 |
| 24 | 0.005 | 7 | 0.75 | **0.766** | 9.448 | 0.455 | 0.653 | 0.731 | 0.592 | 0.260 |
| 25 | 0.005 | 8 | 0.75 | **0.766** | 9.448 | 0.455 | 0.653 | 0.731 | 0.592 | 0.260 |
| 26 | 0.005 | 9 | 0.75 | **0.766** | 9.448 | 0.455 | 0.653 | 0.731 | 0.592 | 0.260 |
| 27 | 0.005 | 10 | 0.75 | **0.766** | 9.448 | 0.455 | 0.653 | 0.731 | 0.592 | 0.260 |
| 28 | 0.0005 | 2 | 0.70 | **0.769** | 9.136 | 0.458 | 0.634 | 0.748 | 0.604 | 0.239 |
| 29 | 0.0005 | 3 | 0.70 | **0.769** | 9.136 | 0.458 | 0.634 | 0.748 | 0.604 | 0.239 |
| 30 | 0.0005 | 4 | 0.70 | **0.769** | 9.136 | 0.458 | 0.634 | 0.748 | 0.604 | 0.239 |
| 31 | 0.0005 | 5 | 0.70 | **0.769** | 9.136 | 0.458 | 0.634 | 0.748 | 0.604 | 0.239 |
| 32 | 0.0005 | 6 | 0.70 | **0.769** | 9.136 | 0.458 | 0.634 | 0.748 | 0.604 | 0.239 |
| 33 | 0.0005 | 7 | 0.70 | **0.769** | 9.136 | 0.458 | 0.634 | 0.748 | 0.604 | 0.239 |
| 34 | 0.0005 | 8 | 0.70 | **0.769** | 9.136 | 0.458 | 0.634 | 0.748 | 0.604 | 0.239 |
| 35 | 0.0005 | 9 | 0.70 | **0.769** | 9.136 | 0.458 | 0.634 | 0.748 | 0.604 | 0.239 |
| 36 | 0.0005 | 10 | 0.70 | **0.769** | 9.136 | 0.458 | 0.634 | 0.748 | 0.604 | 0.239 |
| 37 | 0.0005 | 2 | 0.75 | **0.769** | 9.154 | 0.452 | 0.629 | 0.758 | 0.610 | 0.227 |
| 38 | 0.0005 | 3 | 0.75 | **0.769** | 9.154 | 0.452 | 0.633 | 0.755 | 0.608 | 0.231 |
| 39 | 0.0005 | 4 | 0.75 | **0.769** | 9.154 | 0.452 | 0.633 | 0.755 | 0.608 | 0.231 |
| 40 | 0.0005 | 5 | 0.75 | **0.769** | 9.154 | 0.452 | 0.633 | 0.755 | 0.608 | 0.231 |
| 41 | 0.0005 | 6 | 0.75 | **0.769** | 9.154 | 0.452 | 0.633 | 0.755 | 0.608 | 0.231 |
| 42 | 0.0005 | 7 | 0.75 | **0.769** | 9.154 | 0.452 | 0.633 | 0.755 | 0.608 | 0.231 |
| 43 | 0.0005 | 8 | 0.75 | **0.769** | 9.154 | 0.452 | 0.633 | 0.755 | 0.608 | 0.231 |
| 44 | 0.0005 | 9 | 0.75 | **0.769** | 9.154 | 0.452 | 0.633 | 0.755 | 0.608 | 0.231 |
| 45 | 0.0005 | 10 | 0.75 | **0.769** | 9.154 | 0.452 | 0.633 | 0.755 | 0.608 | 0.231 |
| 46 | 0.005 | 2 | 0.70 | **0.771** | 8.811 | 0.448 | 0.630 | 0.755 | 0.608 | 0.231 |
| 47 | 0.005 | 3 | 0.70 | **0.771** | 8.811 | 0.448 | 0.630 | 0.755 | 0.608 | 0.231 |
| 48 | 0.005 | 4 | 0.70 | **0.771** | 8.811 | 0.448 | 0.630 | 0.755 | 0.608 | 0.231 |
| 49 | 0.005 | 5 | 0.70 | **0.771** | 8.811 | 0.448 | 0.630 | 0.755 | 0.608 | 0.231 |
| 50 | 0.005 | 6 | 0.70 | **0.771** | 8.811 | 0.448 | 0.630 | 0.755 | 0.608 | 0.231 |
| 51 | 0.005 | 7 | 0.70 | **0.771** | 8.811 | 0.448 | 0.630 | 0.755 | 0.608 | 0.231 |
| 52 | 0.005 | 8 | 0.70 | **0.771** | 8.811 | 0.448 | 0.630 | 0.755 | 0.608 | 0.231 |
| 53 | 0.005 | 9 | 0.70 | **0.771** | 8.811 | 0.448 | 0.630 | 0.755 | 0.608 | 0.231 |
| 54 | 0.005 | 10 | 0.70 | **0.771** | 8.811 | 0.448 | 0.630 | 0.755 | 0.608 | 0.231 |
| 55 | 0.0001 | 2 | 0.75 | **0.771** | 8.868 | 0.449 | 0.607 | 0.786 | 0.623 | 0.190 |
| 56 | 0.0001 | 3 | 0.75 | **0.771** | 8.868 | 0.449 | 0.611 | 0.784 | 0.622 | 0.193 |
| 57 | 0.0001 | 4 | 0.75 | **0.771** | 8.868 | 0.449 | 0.611 | 0.784 | 0.622 | 0.193 |
| 58 | 0.0001 | 5 | 0.75 | **0.771** | 8.868 | 0.449 | 0.611 | 0.784 | 0.622 | 0.193 |
| 59 | 0.0001 | 6 | 0.75 | **0.771** | 8.868 | 0.449 | 0.611 | 0.784 | 0.622 | 0.193 |
| 60 | 0.0001 | 7 | 0.75 | **0.771** | 8.868 | 0.449 | 0.611 | 0.784 | 0.622 | 0.193 |
| 61 | 0.0001 | 8 | 0.75 | **0.771** | 8.868 | 0.449 | 0.611 | 0.784 | 0.622 | 0.193 |
| 62 | 0.0001 | 9 | 0.75 | **0.771** | 8.868 | 0.449 | 0.611 | 0.784 | 0.622 | 0.193 |
| 63 | 0.0001 | 10 | 0.75 | **0.771** | 8.868 | 0.449 | 0.611 | 0.784 | 0.622 | 0.193 |
| 64 | 0.001 | 2 | 0.65 | **0.773** | 8.660 | 0.456 | 0.636 | 0.740 | 0.602 | 0.250 |
| 65 | 0.001 | 3 | 0.65 | **0.773** | 8.660 | 0.456 | 0.636 | 0.740 | 0.602 | 0.250 |
| 66 | 0.001 | 4 | 0.65 | **0.773** | 8.660 | 0.456 | 0.636 | 0.740 | 0.602 | 0.250 |
| 67 | 0.001 | 5 | 0.65 | **0.773** | 8.660 | 0.456 | 0.636 | 0.740 | 0.602 | 0.250 |
| 68 | 0.001 | 6 | 0.65 | **0.773** | 8.660 | 0.456 | 0.636 | 0.740 | 0.602 | 0.250 |
| 69 | 0.001 | 7 | 0.65 | **0.773** | 8.660 | 0.456 | 0.636 | 0.740 | 0.602 | 0.250 |
| 70 | 0.001 | 8 | 0.65 | **0.773** | 8.660 | 0.456 | 0.636 | 0.740 | 0.602 | 0.250 |
| 71 | 0.001 | 9 | 0.65 | **0.773** | 8.660 | 0.456 | 0.636 | 0.740 | 0.602 | 0.250 |
| 72 | 0.001 | 10 | 0.65 | **0.773** | 8.660 | 0.456 | 0.636 | 0.740 | 0.602 | 0.250 |
| 73 | 0.0001 | 2 | 0.70 | **0.773** | 8.579 | 0.451 | 0.601 | 0.789 | 0.625 | 0.187 |
| 74 | 0.0001 | 3 | 0.70 | **0.773** | 8.579 | 0.451 | 0.601 | 0.789 | 0.625 | 0.187 |
| 75 | 0.0001 | 4 | 0.70 | **0.773** | 8.579 | 0.451 | 0.601 | 0.789 | 0.625 | 0.187 |
| 76 | 0.0001 | 5 | 0.70 | **0.773** | 8.579 | 0.451 | 0.601 | 0.789 | 0.625 | 0.187 |
| 77 | 0.0001 | 6 | 0.70 | **0.773** | 8.579 | 0.451 | 0.601 | 0.789 | 0.625 | 0.187 |
| 78 | 0.0001 | 7 | 0.70 | **0.773** | 8.579 | 0.451 | 0.601 | 0.789 | 0.625 | 0.187 |
| 79 | 0.0001 | 8 | 0.70 | **0.773** | 8.579 | 0.451 | 0.601 | 0.789 | 0.625 | 0.187 |
| 80 | 0.0001 | 9 | 0.70 | **0.773** | 8.579 | 0.451 | 0.601 | 0.789 | 0.625 | 0.187 |
| 81 | 0.0001 | 10 | 0.70 | **0.773** | 8.579 | 0.451 | 0.601 | 0.789 | 0.625 | 0.187 |
| 82 | 0.0005 | 2 | 0.65 | **0.774** | 8.507 | 0.453 | 0.621 | 0.756 | 0.611 | 0.230 |
| 83 | 0.0005 | 3 | 0.65 | **0.774** | 8.507 | 0.453 | 0.621 | 0.756 | 0.611 | 0.230 |
| 84 | 0.0005 | 4 | 0.65 | **0.774** | 8.507 | 0.453 | 0.621 | 0.756 | 0.611 | 0.230 |
| 85 | 0.0005 | 5 | 0.65 | **0.774** | 8.507 | 0.453 | 0.621 | 0.756 | 0.611 | 0.230 |
| 86 | 0.0005 | 6 | 0.65 | **0.774** | 8.507 | 0.453 | 0.621 | 0.756 | 0.611 | 0.230 |
| 87 | 0.0005 | 7 | 0.65 | **0.774** | 8.507 | 0.453 | 0.621 | 0.756 | 0.611 | 0.230 |
| 88 | 0.0005 | 8 | 0.65 | **0.774** | 8.507 | 0.453 | 0.621 | 0.756 | 0.611 | 0.230 |
| 89 | 0.0005 | 9 | 0.65 | **0.774** | 8.507 | 0.453 | 0.621 | 0.756 | 0.611 | 0.230 |
| 90 | 0.0005 | 10 | 0.65 | **0.774** | 8.507 | 0.453 | 0.621 | 0.756 | 0.611 | 0.230 |
| 91 | 0.01 | 2 | 0.65 | **0.775** | 8.450 | 0.452 | 0.639 | 0.730 | 0.593 | 0.261 |
| 92 | 0.01 | 3 | 0.65 | **0.775** | 8.450 | 0.452 | 0.639 | 0.730 | 0.593 | 0.261 |
| 93 | 0.01 | 4 | 0.65 | **0.775** | 8.450 | 0.452 | 0.639 | 0.730 | 0.593 | 0.261 |
| 94 | 0.01 | 5 | 0.65 | **0.775** | 8.450 | 0.452 | 0.639 | 0.730 | 0.593 | 0.261 |
| 95 | 0.01 | 6 | 0.65 | **0.775** | 8.450 | 0.452 | 0.639 | 0.730 | 0.593 | 0.261 |
| 96 | 0.01 | 7 | 0.65 | **0.775** | 8.450 | 0.452 | 0.639 | 0.730 | 0.593 | 0.261 |
| 97 | 0.01 | 8 | 0.65 | **0.775** | 8.450 | 0.452 | 0.639 | 0.730 | 0.593 | 0.261 |
| 98 | 0.01 | 9 | 0.65 | **0.775** | 8.450 | 0.452 | 0.639 | 0.730 | 0.593 | 0.261 |
| 99 | 0.01 | 10 | 0.65 | **0.775** | 8.450 | 0.452 | 0.639 | 0.730 | 0.593 | 0.261 |
| 100 | 0.005 | 2 | 0.65 | **0.777** | 8.211 | 0.447 | 0.637 | 0.739 | 0.600 | 0.250 |
| 101 | 0.005 | 3 | 0.65 | **0.777** | 8.211 | 0.447 | 0.637 | 0.739 | 0.600 | 0.250 |
| 102 | 0.005 | 4 | 0.65 | **0.777** | 8.211 | 0.447 | 0.637 | 0.739 | 0.600 | 0.250 |
| 103 | 0.005 | 5 | 0.65 | **0.777** | 8.211 | 0.447 | 0.637 | 0.739 | 0.600 | 0.250 |
| 104 | 0.005 | 6 | 0.65 | **0.777** | 8.211 | 0.447 | 0.637 | 0.739 | 0.600 | 0.250 |
| 105 | 0.005 | 7 | 0.65 | **0.777** | 8.211 | 0.447 | 0.637 | 0.739 | 0.600 | 0.250 |
| 106 | 0.005 | 8 | 0.65 | **0.777** | 8.211 | 0.447 | 0.637 | 0.739 | 0.600 | 0.250 |
| 107 | 0.005 | 9 | 0.65 | **0.777** | 8.211 | 0.447 | 0.637 | 0.739 | 0.600 | 0.250 |
| 108 | 0.005 | 10 | 0.65 | **0.777** | 8.211 | 0.447 | 0.637 | 0.739 | 0.600 | 0.250 |
| 109 | 0.0001 | 2 | 0.65 | **0.780** | 7.819 | 0.441 | 0.588 | 0.796 | 0.631 | 0.178 |
| 110 | 0.0001 | 3 | 0.65 | **0.780** | 7.819 | 0.441 | 0.588 | 0.796 | 0.631 | 0.178 |
| 111 | 0.0001 | 4 | 0.65 | **0.780** | 7.819 | 0.441 | 0.588 | 0.796 | 0.631 | 0.178 |
| 112 | 0.0001 | 5 | 0.65 | **0.780** | 7.819 | 0.441 | 0.588 | 0.796 | 0.631 | 0.178 |
| 113 | 0.0001 | 6 | 0.65 | **0.780** | 7.819 | 0.441 | 0.588 | 0.796 | 0.631 | 0.178 |
| 114 | 0.0001 | 7 | 0.65 | **0.780** | 7.819 | 0.441 | 0.588 | 0.796 | 0.631 | 0.178 |
| 115 | 0.0001 | 8 | 0.65 | **0.780** | 7.819 | 0.441 | 0.588 | 0.796 | 0.631 | 0.178 |
| 116 | 0.0001 | 9 | 0.65 | **0.780** | 7.819 | 0.441 | 0.588 | 0.796 | 0.631 | 0.178 |
| 117 | 0.0001 | 10 | 0.65 | **0.780** | 7.819 | 0.441 | 0.588 | 0.796 | 0.631 | 0.178 |
| 118 | 0.001 | 1 | 0.50 | **0.781** | 7.697 | 0.446 | 0.528 | 0.806 | 0.648 | 0.163 |
| 119 | 0.005 | 1 | 0.50 | **0.781** | 7.648 | 0.446 | 0.516 | 0.814 | 0.650 | 0.153 |
| 120 | 0.001 | 2 | 0.50 | **0.781** | 7.697 | 0.446 | 0.528 | 0.806 | 0.648 | 0.163 |
| 121 | 0.005 | 2 | 0.50 | **0.781** | 7.648 | 0.446 | 0.516 | 0.814 | 0.650 | 0.153 |
| 122 | 0.001 | 3 | 0.50 | **0.781** | 7.697 | 0.446 | 0.528 | 0.806 | 0.648 | 0.163 |
| 123 | 0.005 | 3 | 0.50 | **0.781** | 7.648 | 0.446 | 0.516 | 0.814 | 0.650 | 0.153 |
| 124 | 0.001 | 4 | 0.50 | **0.781** | 7.697 | 0.446 | 0.528 | 0.806 | 0.648 | 0.163 |
| 125 | 0.005 | 4 | 0.50 | **0.781** | 7.648 | 0.446 | 0.516 | 0.814 | 0.650 | 0.153 |
| 126 | 0.001 | 5 | 0.50 | **0.781** | 7.697 | 0.446 | 0.528 | 0.806 | 0.648 | 0.163 |
| 127 | 0.005 | 5 | 0.50 | **0.781** | 7.648 | 0.446 | 0.516 | 0.814 | 0.650 | 0.153 |
| 128 | 0.001 | 6 | 0.50 | **0.781** | 7.697 | 0.446 | 0.528 | 0.806 | 0.648 | 0.163 |
| 129 | 0.005 | 6 | 0.50 | **0.781** | 7.648 | 0.446 | 0.516 | 0.814 | 0.650 | 0.153 |
| 130 | 0.001 | 7 | 0.50 | **0.781** | 7.697 | 0.446 | 0.528 | 0.806 | 0.648 | 0.163 |
| 131 | 0.005 | 7 | 0.50 | **0.781** | 7.648 | 0.446 | 0.516 | 0.814 | 0.650 | 0.153 |
| 132 | 0.001 | 8 | 0.50 | **0.781** | 7.697 | 0.446 | 0.528 | 0.806 | 0.648 | 0.163 |
| 133 | 0.005 | 8 | 0.50 | **0.781** | 7.648 | 0.446 | 0.516 | 0.814 | 0.650 | 0.153 |
| 134 | 0.001 | 9 | 0.50 | **0.781** | 7.697 | 0.446 | 0.528 | 0.806 | 0.648 | 0.163 |
| 135 | 0.005 | 9 | 0.50 | **0.781** | 7.648 | 0.446 | 0.516 | 0.814 | 0.650 | 0.153 |
| 136 | 0.001 | 10 | 0.50 | **0.781** | 7.697 | 0.446 | 0.528 | 0.806 | 0.648 | 0.163 |
| 137 | 0.005 | 10 | 0.50 | **0.781** | 7.648 | 0.446 | 0.516 | 0.814 | 0.650 | 0.153 |
| 138 | 0.001 | 2 | 0.60 | **0.781** | 7.677 | 0.450 | 0.605 | 0.763 | 0.619 | 0.220 |
| 139 | 0.001 | 3 | 0.60 | **0.781** | 7.677 | 0.450 | 0.605 | 0.763 | 0.619 | 0.220 |
| 140 | 0.001 | 4 | 0.60 | **0.781** | 7.677 | 0.450 | 0.605 | 0.763 | 0.619 | 0.220 |
| 141 | 0.001 | 5 | 0.60 | **0.781** | 7.677 | 0.450 | 0.605 | 0.763 | 0.619 | 0.220 |
| 142 | 0.001 | 6 | 0.60 | **0.781** | 7.677 | 0.450 | 0.605 | 0.763 | 0.619 | 0.220 |
| 143 | 0.001 | 7 | 0.60 | **0.781** | 7.677 | 0.450 | 0.605 | 0.763 | 0.619 | 0.220 |
| 144 | 0.001 | 8 | 0.60 | **0.781** | 7.677 | 0.450 | 0.605 | 0.763 | 0.619 | 0.220 |
| 145 | 0.001 | 9 | 0.60 | **0.781** | 7.677 | 0.450 | 0.605 | 0.763 | 0.619 | 0.220 |
| 146 | 0.001 | 10 | 0.60 | **0.781** | 7.677 | 0.450 | 0.605 | 0.763 | 0.619 | 0.220 |
| 147 | 0.01 | 1 | 0.50 | **0.782** | 7.533 | 0.456 | 0.562 | 0.777 | 0.630 | 0.202 |
| 148 | 0.01 | 2 | 0.50 | **0.782** | 7.533 | 0.456 | 0.562 | 0.777 | 0.630 | 0.202 |
| 149 | 0.01 | 3 | 0.50 | **0.782** | 7.533 | 0.456 | 0.562 | 0.777 | 0.630 | 0.202 |
| 150 | 0.01 | 4 | 0.50 | **0.782** | 7.533 | 0.456 | 0.562 | 0.777 | 0.630 | 0.202 |
| 151 | 0.01 | 5 | 0.50 | **0.782** | 7.533 | 0.456 | 0.562 | 0.777 | 0.630 | 0.202 |
| 152 | 0.01 | 6 | 0.50 | **0.782** | 7.533 | 0.456 | 0.562 | 0.777 | 0.630 | 0.202 |
| 153 | 0.01 | 7 | 0.50 | **0.782** | 7.533 | 0.456 | 0.562 | 0.777 | 0.630 | 0.202 |
| 154 | 0.01 | 8 | 0.50 | **0.782** | 7.533 | 0.456 | 0.562 | 0.777 | 0.630 | 0.202 |
| 155 | 0.01 | 9 | 0.50 | **0.782** | 7.533 | 0.456 | 0.562 | 0.777 | 0.630 | 0.202 |
| 156 | 0.01 | 10 | 0.50 | **0.782** | 7.533 | 0.456 | 0.562 | 0.777 | 0.630 | 0.202 |
| 157 | 0.0005 | 2 | 0.60 | **0.782** | 7.556 | 0.453 | 0.610 | 0.758 | 0.617 | 0.226 |
| 158 | 0.0005 | 3 | 0.60 | **0.782** | 7.556 | 0.453 | 0.610 | 0.758 | 0.617 | 0.226 |
| 159 | 0.0005 | 4 | 0.60 | **0.782** | 7.556 | 0.453 | 0.610 | 0.758 | 0.617 | 0.226 |
| 160 | 0.0005 | 5 | 0.60 | **0.782** | 7.556 | 0.453 | 0.610 | 0.758 | 0.617 | 0.226 |
| 161 | 0.0005 | 6 | 0.60 | **0.782** | 7.556 | 0.453 | 0.610 | 0.758 | 0.617 | 0.226 |
| 162 | 0.0005 | 7 | 0.60 | **0.782** | 7.556 | 0.453 | 0.610 | 0.758 | 0.617 | 0.226 |
| 163 | 0.0005 | 8 | 0.60 | **0.782** | 7.556 | 0.453 | 0.610 | 0.758 | 0.617 | 0.226 |
| 164 | 0.0005 | 9 | 0.60 | **0.782** | 7.556 | 0.453 | 0.610 | 0.758 | 0.617 | 0.226 |
| 165 | 0.0005 | 10 | 0.60 | **0.782** | 7.556 | 0.453 | 0.610 | 0.758 | 0.617 | 0.226 |
| 166 | 0.0005 | 1 | 0.50 | **0.783** | 7.403 | 0.441 | 0.520 | 0.810 | 0.652 | 0.158 |
| 167 | 0.0005 | 2 | 0.50 | **0.783** | 7.403 | 0.441 | 0.520 | 0.810 | 0.652 | 0.158 |
| 168 | 0.0005 | 3 | 0.50 | **0.783** | 7.403 | 0.441 | 0.520 | 0.810 | 0.652 | 0.158 |
| 169 | 0.0005 | 4 | 0.50 | **0.783** | 7.403 | 0.441 | 0.520 | 0.810 | 0.652 | 0.158 |
| 170 | 0.0005 | 5 | 0.50 | **0.783** | 7.403 | 0.441 | 0.520 | 0.810 | 0.652 | 0.158 |
| 171 | 0.0005 | 6 | 0.50 | **0.783** | 7.403 | 0.441 | 0.520 | 0.810 | 0.652 | 0.158 |
| 172 | 0.0005 | 7 | 0.50 | **0.783** | 7.403 | 0.441 | 0.520 | 0.810 | 0.652 | 0.158 |
| 173 | 0.0005 | 8 | 0.50 | **0.783** | 7.403 | 0.441 | 0.520 | 0.810 | 0.652 | 0.158 |
| 174 | 0.0005 | 9 | 0.50 | **0.783** | 7.403 | 0.441 | 0.520 | 0.810 | 0.652 | 0.158 |
| 175 | 0.0005 | 10 | 0.50 | **0.783** | 7.403 | 0.441 | 0.520 | 0.810 | 0.652 | 0.158 |
| 176 | 0.0005 | 1 | 0.55 | **0.783** | 7.457 | 0.449 | 0.542 | 0.800 | 0.645 | 0.173 |
| 177 | 0.001 | 1 | 0.55 | **0.783** | 7.428 | 0.443 | 0.530 | 0.809 | 0.650 | 0.161 |
| 178 | 0.0005 | 2 | 0.55 | **0.783** | 7.457 | 0.449 | 0.563 | 0.787 | 0.637 | 0.189 |
| 179 | 0.001 | 2 | 0.55 | **0.783** | 7.428 | 0.443 | 0.550 | 0.798 | 0.643 | 0.175 |
| 180 | 0.0005 | 3 | 0.55 | **0.783** | 7.457 | 0.449 | 0.563 | 0.787 | 0.637 | 0.189 |
| 181 | 0.001 | 3 | 0.55 | **0.783** | 7.428 | 0.443 | 0.550 | 0.798 | 0.643 | 0.175 |
| 182 | 0.0005 | 4 | 0.55 | **0.783** | 7.457 | 0.449 | 0.563 | 0.787 | 0.637 | 0.189 |
| 183 | 0.001 | 4 | 0.55 | **0.783** | 7.428 | 0.443 | 0.550 | 0.798 | 0.643 | 0.175 |
| 184 | 0.0005 | 5 | 0.55 | **0.783** | 7.457 | 0.449 | 0.563 | 0.787 | 0.637 | 0.189 |
| 185 | 0.001 | 5 | 0.55 | **0.783** | 7.428 | 0.443 | 0.550 | 0.798 | 0.643 | 0.175 |
| 186 | 0.0005 | 6 | 0.55 | **0.783** | 7.457 | 0.449 | 0.563 | 0.787 | 0.637 | 0.189 |
| 187 | 0.001 | 6 | 0.55 | **0.783** | 7.428 | 0.443 | 0.550 | 0.798 | 0.643 | 0.175 |
| 188 | 0.0005 | 7 | 0.55 | **0.783** | 7.457 | 0.449 | 0.563 | 0.787 | 0.637 | 0.189 |
| 189 | 0.001 | 7 | 0.55 | **0.783** | 7.428 | 0.443 | 0.550 | 0.798 | 0.643 | 0.175 |
| 190 | 0.0005 | 8 | 0.55 | **0.783** | 7.457 | 0.449 | 0.563 | 0.787 | 0.637 | 0.189 |
| 191 | 0.001 | 8 | 0.55 | **0.783** | 7.428 | 0.443 | 0.550 | 0.798 | 0.643 | 0.175 |
| 192 | 0.0005 | 9 | 0.55 | **0.783** | 7.457 | 0.449 | 0.563 | 0.787 | 0.637 | 0.189 |
| 193 | 0.001 | 9 | 0.55 | **0.783** | 7.428 | 0.443 | 0.550 | 0.798 | 0.643 | 0.175 |
| 194 | 0.0005 | 10 | 0.55 | **0.783** | 7.457 | 0.449 | 0.563 | 0.787 | 0.637 | 0.189 |
| 195 | 0.001 | 10 | 0.55 | **0.783** | 7.428 | 0.443 | 0.550 | 0.798 | 0.643 | 0.175 |
| 196 | 0.01 | 1 | 0.60 | **0.783** | 7.409 | 0.451 | 0.560 | 0.792 | 0.637 | 0.183 |
| 197 | 0.005 | 1 | 0.55 | **0.785** | 7.177 | 0.444 | 0.536 | 0.804 | 0.645 | 0.168 |
| 198 | 0.005 | 2 | 0.55 | **0.785** | 7.177 | 0.444 | 0.558 | 0.791 | 0.637 | 0.184 |
| 199 | 0.005 | 3 | 0.55 | **0.785** | 7.177 | 0.444 | 0.558 | 0.791 | 0.637 | 0.184 |
| 200 | 0.005 | 4 | 0.55 | **0.785** | 7.177 | 0.444 | 0.558 | 0.791 | 0.637 | 0.184 |
| 201 | 0.005 | 5 | 0.55 | **0.785** | 7.177 | 0.444 | 0.558 | 0.791 | 0.637 | 0.184 |
| 202 | 0.005 | 6 | 0.55 | **0.785** | 7.177 | 0.444 | 0.558 | 0.791 | 0.637 | 0.184 |
| 203 | 0.005 | 7 | 0.55 | **0.785** | 7.177 | 0.444 | 0.558 | 0.791 | 0.637 | 0.184 |
| 204 | 0.005 | 8 | 0.55 | **0.785** | 7.177 | 0.444 | 0.558 | 0.791 | 0.637 | 0.184 |
| 205 | 0.005 | 9 | 0.55 | **0.785** | 7.177 | 0.444 | 0.558 | 0.791 | 0.637 | 0.184 |
| 206 | 0.005 | 10 | 0.55 | **0.785** | 7.177 | 0.444 | 0.558 | 0.791 | 0.637 | 0.184 |
| 207 | 0.001 | 1 | 0.60 | **0.785** | 7.188 | 0.443 | 0.544 | 0.802 | 0.646 | 0.170 |
| 208 | 0.005 | 2 | 0.60 | **0.785** | 7.172 | 0.445 | 0.609 | 0.760 | 0.615 | 0.224 |
| 209 | 0.005 | 3 | 0.60 | **0.785** | 7.172 | 0.445 | 0.609 | 0.760 | 0.615 | 0.224 |
| 210 | 0.005 | 4 | 0.60 | **0.785** | 7.172 | 0.445 | 0.609 | 0.760 | 0.615 | 0.224 |
| 211 | 0.005 | 5 | 0.60 | **0.785** | 7.172 | 0.445 | 0.609 | 0.760 | 0.615 | 0.224 |
| 212 | 0.005 | 6 | 0.60 | **0.785** | 7.172 | 0.445 | 0.609 | 0.760 | 0.615 | 0.224 |
| 213 | 0.005 | 7 | 0.60 | **0.785** | 7.172 | 0.445 | 0.609 | 0.760 | 0.615 | 0.224 |
| 214 | 0.005 | 8 | 0.60 | **0.785** | 7.172 | 0.445 | 0.609 | 0.760 | 0.615 | 0.224 |
| 215 | 0.005 | 9 | 0.60 | **0.785** | 7.172 | 0.445 | 0.609 | 0.760 | 0.615 | 0.224 |
| 216 | 0.005 | 10 | 0.60 | **0.785** | 7.172 | 0.445 | 0.609 | 0.760 | 0.615 | 0.224 |
| 217 | 0.0005 | 1 | 0.60 | **0.786** | 7.049 | 0.447 | 0.557 | 0.792 | 0.642 | 0.183 |
| 218 | 0.01 | 1 | 0.55 | **0.787** | 6.947 | 0.449 | 0.570 | 0.772 | 0.627 | 0.209 |
| 219 | 0.01 | 2 | 0.55 | **0.787** | 6.947 | 0.449 | 0.589 | 0.760 | 0.619 | 0.225 |
| 220 | 0.01 | 3 | 0.55 | **0.787** | 6.947 | 0.449 | 0.589 | 0.760 | 0.619 | 0.225 |
| 221 | 0.01 | 4 | 0.55 | **0.787** | 6.947 | 0.449 | 0.589 | 0.760 | 0.619 | 0.225 |
| 222 | 0.01 | 5 | 0.55 | **0.787** | 6.947 | 0.449 | 0.589 | 0.760 | 0.619 | 0.225 |
| 223 | 0.01 | 6 | 0.55 | **0.787** | 6.947 | 0.449 | 0.589 | 0.760 | 0.619 | 0.225 |
| 224 | 0.01 | 7 | 0.55 | **0.787** | 6.947 | 0.449 | 0.589 | 0.760 | 0.619 | 0.225 |
| 225 | 0.01 | 8 | 0.55 | **0.787** | 6.947 | 0.449 | 0.589 | 0.760 | 0.619 | 0.225 |
| 226 | 0.01 | 9 | 0.55 | **0.787** | 6.947 | 0.449 | 0.589 | 0.760 | 0.619 | 0.225 |
| 227 | 0.01 | 10 | 0.55 | **0.787** | 6.947 | 0.449 | 0.589 | 0.760 | 0.619 | 0.225 |
| 228 | 0.005 | 1 | 0.60 | **0.788** | 6.888 | 0.437 | 0.535 | 0.809 | 0.646 | 0.160 |
| 229 | 0.0005 | 1 | 0.65 | **0.788** | 6.801 | 0.442 | 0.566 | 0.789 | 0.638 | 0.187 |
| 230 | 0.001 | 1 | 0.65 | **0.788** | 6.838 | 0.442 | 0.560 | 0.794 | 0.641 | 0.180 |
| 231 | 0.005 | 1 | 0.65 | **0.789** | 6.722 | 0.440 | 0.576 | 0.782 | 0.634 | 0.197 |
| 232 | 0.0001 | 2 | 0.60 | **0.790** | 6.658 | 0.423 | 0.558 | 0.810 | 0.641 | 0.159 |
| 233 | 0.0001 | 3 | 0.60 | **0.790** | 6.658 | 0.423 | 0.558 | 0.810 | 0.641 | 0.159 |
| 234 | 0.0001 | 4 | 0.60 | **0.790** | 6.658 | 0.423 | 0.558 | 0.810 | 0.641 | 0.159 |
| 235 | 0.0001 | 5 | 0.60 | **0.790** | 6.658 | 0.423 | 0.558 | 0.810 | 0.641 | 0.159 |
| 236 | 0.0001 | 6 | 0.60 | **0.790** | 6.658 | 0.423 | 0.558 | 0.810 | 0.641 | 0.159 |
| 237 | 0.0001 | 7 | 0.60 | **0.790** | 6.658 | 0.423 | 0.558 | 0.810 | 0.641 | 0.159 |
| 238 | 0.0001 | 8 | 0.60 | **0.790** | 6.658 | 0.423 | 0.558 | 0.810 | 0.641 | 0.159 |
| 239 | 0.0001 | 9 | 0.60 | **0.790** | 6.658 | 0.423 | 0.558 | 0.810 | 0.641 | 0.159 |
| 240 | 0.0001 | 10 | 0.60 | **0.790** | 6.658 | 0.423 | 0.558 | 0.810 | 0.641 | 0.159 |
| 241 | 0.0001 | 1 | 0.50 | **0.791** | 6.530 | 0.416 | 0.473 | 0.841 | 0.663 | 0.115 |
| 242 | 0.0001 | 2 | 0.50 | **0.791** | 6.530 | 0.416 | 0.473 | 0.841 | 0.663 | 0.115 |
| 243 | 0.0001 | 3 | 0.50 | **0.791** | 6.530 | 0.416 | 0.473 | 0.841 | 0.663 | 0.115 |
| 244 | 0.0001 | 4 | 0.50 | **0.791** | 6.530 | 0.416 | 0.473 | 0.841 | 0.663 | 0.115 |
| 245 | 0.0001 | 5 | 0.50 | **0.791** | 6.530 | 0.416 | 0.473 | 0.841 | 0.663 | 0.115 |
| 246 | 0.0001 | 6 | 0.50 | **0.791** | 6.530 | 0.416 | 0.473 | 0.841 | 0.663 | 0.115 |
| 247 | 0.0001 | 7 | 0.50 | **0.791** | 6.530 | 0.416 | 0.473 | 0.841 | 0.663 | 0.115 |
| 248 | 0.0001 | 8 | 0.50 | **0.791** | 6.530 | 0.416 | 0.473 | 0.841 | 0.663 | 0.115 |
| 249 | 0.0001 | 9 | 0.50 | **0.791** | 6.530 | 0.416 | 0.473 | 0.841 | 0.663 | 0.115 |
| 250 | 0.0001 | 10 | 0.50 | **0.791** | 6.530 | 0.416 | 0.473 | 0.841 | 0.663 | 0.115 |
| 251 | 0.0001 | 1 | 0.55 | **0.793** | 6.283 | 0.413 | 0.476 | 0.841 | 0.662 | 0.115 |
| 252 | 0.0001 | 2 | 0.55 | **0.793** | 6.283 | 0.413 | 0.502 | 0.833 | 0.656 | 0.127 |
| 253 | 0.0001 | 3 | 0.55 | **0.793** | 6.283 | 0.413 | 0.502 | 0.833 | 0.656 | 0.127 |
| 254 | 0.0001 | 4 | 0.55 | **0.793** | 6.283 | 0.413 | 0.502 | 0.833 | 0.656 | 0.127 |
| 255 | 0.0001 | 5 | 0.55 | **0.793** | 6.283 | 0.413 | 0.502 | 0.833 | 0.656 | 0.127 |
| 256 | 0.0001 | 6 | 0.55 | **0.793** | 6.283 | 0.413 | 0.502 | 0.833 | 0.656 | 0.127 |
| 257 | 0.0001 | 7 | 0.55 | **0.793** | 6.283 | 0.413 | 0.502 | 0.833 | 0.656 | 0.127 |
| 258 | 0.0001 | 8 | 0.55 | **0.793** | 6.283 | 0.413 | 0.502 | 0.833 | 0.656 | 0.127 |
| 259 | 0.0001 | 9 | 0.55 | **0.793** | 6.283 | 0.413 | 0.502 | 0.833 | 0.656 | 0.127 |
| 260 | 0.0001 | 10 | 0.55 | **0.793** | 6.283 | 0.413 | 0.502 | 0.833 | 0.656 | 0.127 |
| 261 | 0.01 | 1 | 0.65 | **0.793** | 6.280 | 0.429 | 0.568 | 0.788 | 0.638 | 0.188 |
| 262 | 0.005 | 1 | 0.70 | **0.793** | 6.238 | 0.435 | 0.574 | 0.782 | 0.633 | 0.197 |
| 263 | 0.0005 | 1 | 0.70 | **0.794** | 6.179 | 0.433 | 0.566 | 0.791 | 0.639 | 0.184 |
| 264 | 0.001 | 1 | 0.70 | **0.794** | 6.144 | 0.431 | 0.563 | 0.794 | 0.640 | 0.180 |
| 265 | 0.0001 | 1 | 0.60 | **0.795** | 6.000 | 0.408 | 0.481 | 0.840 | 0.660 | 0.117 |
| 266 | 0.0001 | 1 | 0.65 | **0.801** | 5.275 | 0.388 | 0.461 | 0.852 | 0.661 | 0.100 |
| 267 | 0.001 | 1 | 0.75 | **0.802** | 5.207 | 0.417 | 0.561 | 0.795 | 0.640 | 0.178 |
| 268 | 0.0005 | 1 | 0.75 | **0.804** | 4.998 | 0.409 | 0.545 | 0.805 | 0.645 | 0.166 |
| 269 | 0.0001 | 1 | 0.70 | **0.805** | 4.799 | 0.373 | 0.452 | 0.856 | 0.661 | 0.095 |
| 270 | 0.0001 | 1 | 0.75 | **0.810** | 4.275 | 0.354 | 0.432 | 0.864 | 0.662 | 0.083 |
| 271 | 0.05 | 1 | 0.50 | **NA** | NA | NA | NA | NA | NA | NA |
| 272 | 0.05 | 2 | 0.50 | **NA** | NA | NA | NA | NA | NA | NA |
| 273 | 0.05 | 3 | 0.50 | **NA** | NA | NA | NA | NA | NA | NA |
| 274 | 0.05 | 4 | 0.50 | **NA** | NA | NA | NA | NA | NA | NA |
| 275 | 0.05 | 5 | 0.50 | **NA** | NA | NA | NA | NA | NA | NA |
| 276 | 0.05 | 6 | 0.50 | **NA** | NA | NA | NA | NA | NA | NA |
| 277 | 0.05 | 7 | 0.50 | **NA** | NA | NA | NA | NA | NA | NA |
| 278 | 0.05 | 8 | 0.50 | **NA** | NA | NA | NA | NA | NA | NA |
| 279 | 0.05 | 9 | 0.50 | **NA** | NA | NA | NA | NA | NA | NA |
| 280 | 0.05 | 10 | 0.50 | **NA** | NA | NA | NA | NA | NA | NA |
| 281 | 0.05 | 1 | 0.55 | **NA** | NA | NA | NA | NA | NA | NA |
| 282 | 0.05 | 2 | 0.55 | **NA** | NA | NA | NA | NA | NA | NA |
| 283 | 0.05 | 3 | 0.55 | **NA** | NA | NA | NA | NA | NA | NA |
| 284 | 0.05 | 4 | 0.55 | **NA** | NA | NA | NA | NA | NA | NA |
| 285 | 0.05 | 5 | 0.55 | **NA** | NA | NA | NA | NA | NA | NA |
| 286 | 0.05 | 6 | 0.55 | **NA** | NA | NA | NA | NA | NA | NA |
| 287 | 0.05 | 7 | 0.55 | **NA** | NA | NA | NA | NA | NA | NA |
| 288 | 0.05 | 8 | 0.55 | **NA** | NA | NA | NA | NA | NA | NA |
| 289 | 0.05 | 9 | 0.55 | **NA** | NA | NA | NA | NA | NA | NA |
| 290 | 0.05 | 10 | 0.55 | **NA** | NA | NA | NA | NA | NA | NA |
| 291 | 0.05 | 1 | 0.60 | **NA** | NA | NA | NA | NA | NA | NA |
| 292 | 0.01 | 2 | 0.60 | **NA** | NA | NA | NA | NA | NA | NA |
| 293 | 0.05 | 2 | 0.60 | **NA** | NA | NA | NA | NA | NA | NA |
| 294 | 0.01 | 3 | 0.60 | **NA** | NA | NA | NA | NA | NA | NA |
| 295 | 0.05 | 3 | 0.60 | **NA** | NA | NA | NA | NA | NA | NA |
| 296 | 0.01 | 4 | 0.60 | **NA** | NA | NA | NA | NA | NA | NA |
| 297 | 0.05 | 4 | 0.60 | **NA** | NA | NA | NA | NA | NA | NA |
| 298 | 0.01 | 5 | 0.60 | **NA** | NA | NA | NA | NA | NA | NA |
| 299 | 0.05 | 5 | 0.60 | **NA** | NA | NA | NA | NA | NA | NA |
| 300 | 0.01 | 6 | 0.60 | **NA** | NA | NA | NA | NA | NA | NA |
| 301 | 0.05 | 6 | 0.60 | **NA** | NA | NA | NA | NA | NA | NA |
| 302 | 0.01 | 7 | 0.60 | **NA** | NA | NA | NA | NA | NA | NA |
| 303 | 0.05 | 7 | 0.60 | **NA** | NA | NA | NA | NA | NA | NA |
| 304 | 0.01 | 8 | 0.60 | **NA** | NA | NA | NA | NA | NA | NA |
| 305 | 0.05 | 8 | 0.60 | **NA** | NA | NA | NA | NA | NA | NA |
| 306 | 0.01 | 9 | 0.60 | **NA** | NA | NA | NA | NA | NA | NA |
| 307 | 0.05 | 9 | 0.60 | **NA** | NA | NA | NA | NA | NA | NA |
| 308 | 0.01 | 10 | 0.60 | **NA** | NA | NA | NA | NA | NA | NA |
| 309 | 0.05 | 10 | 0.60 | **NA** | NA | NA | NA | NA | NA | NA |
| 310 | 0.05 | 1 | 0.65 | **NA** | NA | NA | NA | NA | NA | NA |
| 311 | 0.05 | 2 | 0.65 | **NA** | NA | NA | NA | NA | NA | NA |
| 312 | 0.05 | 3 | 0.65 | **NA** | NA | NA | NA | NA | NA | NA |
| 313 | 0.05 | 4 | 0.65 | **NA** | NA | NA | NA | NA | NA | NA |
| 314 | 0.05 | 5 | 0.65 | **NA** | NA | NA | NA | NA | NA | NA |
| 315 | 0.05 | 6 | 0.65 | **NA** | NA | NA | NA | NA | NA | NA |
| 316 | 0.05 | 7 | 0.65 | **NA** | NA | NA | NA | NA | NA | NA |
| 317 | 0.05 | 8 | 0.65 | **NA** | NA | NA | NA | NA | NA | NA |
| 318 | 0.05 | 9 | 0.65 | **NA** | NA | NA | NA | NA | NA | NA |
| 319 | 0.05 | 10 | 0.65 | **NA** | NA | NA | NA | NA | NA | NA |
| 320 | 0.01 | 1 | 0.70 | **NA** | NA | NA | NA | NA | NA | NA |
| 321 | 0.05 | 1 | 0.70 | **NA** | NA | NA | NA | NA | NA | NA |
| 322 | 0.01 | 2 | 0.70 | **NA** | NA | NA | NA | NA | NA | NA |
| 323 | 0.05 | 2 | 0.70 | **NA** | NA | NA | NA | NA | NA | NA |
| 324 | 0.01 | 3 | 0.70 | **NA** | NA | NA | NA | NA | NA | NA |
| 325 | 0.05 | 3 | 0.70 | **NA** | NA | NA | NA | NA | NA | NA |
| 326 | 0.01 | 4 | 0.70 | **NA** | NA | NA | NA | NA | NA | NA |
| 327 | 0.05 | 4 | 0.70 | **NA** | NA | NA | NA | NA | NA | NA |
| 328 | 0.01 | 5 | 0.70 | **NA** | NA | NA | NA | NA | NA | NA |
| 329 | 0.05 | 5 | 0.70 | **NA** | NA | NA | NA | NA | NA | NA |
| 330 | 0.01 | 6 | 0.70 | **NA** | NA | NA | NA | NA | NA | NA |
| 331 | 0.05 | 6 | 0.70 | **NA** | NA | NA | NA | NA | NA | NA |
| 332 | 0.01 | 7 | 0.70 | **NA** | NA | NA | NA | NA | NA | NA |
| 333 | 0.05 | 7 | 0.70 | **NA** | NA | NA | NA | NA | NA | NA |
| 334 | 0.01 | 8 | 0.70 | **NA** | NA | NA | NA | NA | NA | NA |
| 335 | 0.05 | 8 | 0.70 | **NA** | NA | NA | NA | NA | NA | NA |
| 336 | 0.01 | 9 | 0.70 | **NA** | NA | NA | NA | NA | NA | NA |
| 337 | 0.05 | 9 | 0.70 | **NA** | NA | NA | NA | NA | NA | NA |
| 338 | 0.01 | 10 | 0.70 | **NA** | NA | NA | NA | NA | NA | NA |
| 339 | 0.05 | 10 | 0.70 | **NA** | NA | NA | NA | NA | NA | NA |
| 340 | 0.005 | 1 | 0.75 | **NA** | NA | NA | NA | NA | NA | NA |
| 341 | 0.01 | 1 | 0.75 | **NA** | NA | NA | NA | NA | NA | NA |
| 342 | 0.05 | 1 | 0.75 | **NA** | NA | NA | NA | NA | NA | NA |
| 343 | 0.01 | 2 | 0.75 | **NA** | NA | NA | NA | NA | NA | NA |
| 344 | 0.05 | 2 | 0.75 | **NA** | NA | NA | NA | NA | NA | NA |
| 345 | 0.01 | 3 | 0.75 | **NA** | NA | NA | NA | NA | NA | NA |
| 346 | 0.05 | 3 | 0.75 | **NA** | NA | NA | NA | NA | NA | NA |
| 347 | 0.01 | 4 | 0.75 | **NA** | NA | NA | NA | NA | NA | NA |
| 348 | 0.05 | 4 | 0.75 | **NA** | NA | NA | NA | NA | NA | NA |
| 349 | 0.01 | 5 | 0.75 | **NA** | NA | NA | NA | NA | NA | NA |
| 350 | 0.05 | 5 | 0.75 | **NA** | NA | NA | NA | NA | NA | NA |
| 351 | 0.01 | 6 | 0.75 | **NA** | NA | NA | NA | NA | NA | NA |
| 352 | 0.05 | 6 | 0.75 | **NA** | NA | NA | NA | NA | NA | NA |
| 353 | 0.01 | 7 | 0.75 | **NA** | NA | NA | NA | NA | NA | NA |
| 354 | 0.05 | 7 | 0.75 | **NA** | NA | NA | NA | NA | NA | NA |
| 355 | 0.01 | 8 | 0.75 | **NA** | NA | NA | NA | NA | NA | NA |
| 356 | 0.05 | 8 | 0.75 | **NA** | NA | NA | NA | NA | NA | NA |
| 357 | 0.01 | 9 | 0.75 | **NA** | NA | NA | NA | NA | NA | NA |
| 358 | 0.05 | 9 | 0.75 | **NA** | NA | NA | NA | NA | NA | NA |
| 359 | 0.01 | 10 | 0.75 | **NA** | NA | NA | NA | NA | NA | NA |
| 360 | 0.05 | 10 | 0.75 | **NA** | NA | NA | NA | NA | NA | NA |

**Table S6.** Possible combinations of the three meta-parameters (learning rate, tree complexity, bag fraction) for fitted 360 BRT models for species richness at 0.01 m^2^ spatial scale based on 10-fold cross validation (CV) and the associated model performance parameters. Numbers are sorted according to increasing CV deviance (in bold). RMSE represents root mean square error, and MAE represents mean absolute error.

| No. | Learning rate | Tree complexity | Bag fraction | **CV deviance** | Percentage of explained deviance (%) | CV correlation | Training data correlation | RMSE  (%) | MAE  (%) | *R*^2^ |
| --- | --- | --- | --- | --- | --- | --- | --- | --- | --- | --- |
| 1 | 0.0001 | 3 | 0.75 | **3.945** | 0.025 | 0.001 | 0.495 | 1.894 | 1.579 | 0.030 |
| 2 | 0.0001 | 4 | 0.75 | **3.945** | 0.025 | 0.001 | 0.495 | 1.894 | 1.579 | 0.030 |
| 3 | 0.0001 | 5 | 0.75 | **3.945** | 0.025 | 0.001 | 0.495 | 1.894 | 1.579 | 0.030 |
| 4 | 0.0001 | 6 | 0.75 | **3.945** | 0.025 | 0.001 | 0.495 | 1.894 | 1.579 | 0.030 |
| 5 | 0.0001 | 7 | 0.75 | **3.945** | 0.025 | 0.001 | 0.495 | 1.894 | 1.579 | 0.030 |
| 6 | 0.0001 | 8 | 0.75 | **3.945** | 0.025 | 0.001 | 0.495 | 1.894 | 1.579 | 0.030 |
| 7 | 0.0001 | 9 | 0.75 | **3.945** | 0.025 | 0.001 | 0.495 | 1.894 | 1.579 | 0.030 |
| 8 | 0.0001 | 10 | 0.75 | **3.945** | 0.025 | 0.001 | 0.495 | 1.894 | 1.579 | 0.030 |
| 9 | 0.0001 | 2 | 0.75 | **3.945** | 0.025 | 0.001 | 0.483 | 1.895 | 1.579 | 0.028 |
| 10 | 0.0001 | 1 | 0.50 | **3.944** | 0.051 | 0.000 | 0.349 | 1.896 | 1.581 | 0.028 |
| 11 | 0.0001 | 2 | 0.50 | **3.944** | 0.051 | 0.000 | 0.349 | 1.896 | 1.581 | 0.028 |
| 12 | 0.0001 | 3 | 0.50 | **3.944** | 0.051 | 0.000 | 0.349 | 1.896 | 1.581 | 0.028 |
| 13 | 0.0001 | 4 | 0.50 | **3.944** | 0.051 | 0.000 | 0.349 | 1.896 | 1.581 | 0.028 |
| 14 | 0.0001 | 5 | 0.50 | **3.944** | 0.051 | 0.000 | 0.349 | 1.896 | 1.581 | 0.028 |
| 15 | 0.0001 | 6 | 0.50 | **3.944** | 0.051 | 0.000 | 0.349 | 1.896 | 1.581 | 0.028 |
| 16 | 0.0001 | 7 | 0.50 | **3.944** | 0.051 | 0.000 | 0.349 | 1.896 | 1.581 | 0.028 |
| 17 | 0.0001 | 8 | 0.50 | **3.944** | 0.051 | 0.000 | 0.349 | 1.896 | 1.581 | 0.028 |
| 18 | 0.0001 | 9 | 0.50 | **3.944** | 0.051 | 0.000 | 0.349 | 1.896 | 1.581 | 0.028 |
| 19 | 0.0001 | 10 | 0.50 | **3.944** | 0.051 | 0.000 | 0.349 | 1.896 | 1.581 | 0.028 |
| 20 | 0.0005 | 1 | 0.50 | **NA** | NA | NA | NA | NA | NA | NA |
| 21 | 0.001 | 1 | 0.50 | **NA** | NA | NA | NA | NA | NA | NA |
| 22 | 0.005 | 1 | 0.50 | **NA** | NA | NA | NA | NA | NA | NA |
| 23 | 0.01 | 1 | 0.50 | **NA** | NA | NA | NA | NA | NA | NA |
| 24 | 0.05 | 1 | 0.50 | **NA** | NA | NA | NA | NA | NA | NA |
| 25 | 0.0005 | 2 | 0.50 | **NA** | NA | NA | NA | NA | NA | NA |
| 26 | 0.001 | 2 | 0.50 | **NA** | NA | NA | NA | NA | NA | NA |
| 27 | 0.005 | 2 | 0.50 | **NA** | NA | NA | NA | NA | NA | NA |
| 28 | 0.01 | 2 | 0.50 | **NA** | NA | NA | NA | NA | NA | NA |
| 29 | 0.05 | 2 | 0.50 | **NA** | NA | NA | NA | NA | NA | NA |
| 30 | 0.0005 | 3 | 0.50 | **NA** | NA | NA | NA | NA | NA | NA |
| 31 | 0.001 | 3 | 0.50 | **NA** | NA | NA | NA | NA | NA | NA |
| 32 | 0.005 | 3 | 0.50 | **NA** | NA | NA | NA | NA | NA | NA |
| 33 | 0.01 | 3 | 0.50 | **NA** | NA | NA | NA | NA | NA | NA |
| 34 | 0.05 | 3 | 0.50 | **NA** | NA | NA | NA | NA | NA | NA |
| 35 | 0.0005 | 4 | 0.50 | **NA** | NA | NA | NA | NA | NA | NA |
| 36 | 0.001 | 4 | 0.50 | **NA** | NA | NA | NA | NA | NA | NA |
| 37 | 0.005 | 4 | 0.50 | **NA** | NA | NA | NA | NA | NA | NA |
| 38 | 0.01 | 4 | 0.50 | **NA** | NA | NA | NA | NA | NA | NA |
| 39 | 0.05 | 4 | 0.50 | **NA** | NA | NA | NA | NA | NA | NA |
| 40 | 0.0005 | 5 | 0.50 | **NA** | NA | NA | NA | NA | NA | NA |
| 41 | 0.001 | 5 | 0.50 | **NA** | NA | NA | NA | NA | NA | NA |
| 42 | 0.005 | 5 | 0.50 | **NA** | NA | NA | NA | NA | NA | NA |
| 43 | 0.01 | 5 | 0.50 | **NA** | NA | NA | NA | NA | NA | NA |
| 44 | 0.05 | 5 | 0.50 | **NA** | NA | NA | NA | NA | NA | NA |
| 45 | 0.0005 | 6 | 0.50 | **NA** | NA | NA | NA | NA | NA | NA |
| 46 | 0.001 | 6 | 0.50 | **NA** | NA | NA | NA | NA | NA | NA |
| 47 | 0.005 | 6 | 0.50 | **NA** | NA | NA | NA | NA | NA | NA |
| 48 | 0.01 | 6 | 0.50 | **NA** | NA | NA | NA | NA | NA | NA |
| 49 | 0.05 | 6 | 0.50 | **NA** | NA | NA | NA | NA | NA | NA |
| 50 | 0.0005 | 7 | 0.50 | **NA** | NA | NA | NA | NA | NA | NA |
| 51 | 0.001 | 7 | 0.50 | **NA** | NA | NA | NA | NA | NA | NA |
| 52 | 0.005 | 7 | 0.50 | **NA** | NA | NA | NA | NA | NA | NA |
| 53 | 0.01 | 7 | 0.50 | **NA** | NA | NA | NA | NA | NA | NA |
| 54 | 0.05 | 7 | 0.50 | **NA** | NA | NA | NA | NA | NA | NA |
| 55 | 0.0005 | 8 | 0.50 | **NA** | NA | NA | NA | NA | NA | NA |
| 56 | 0.001 | 8 | 0.50 | **NA** | NA | NA | NA | NA | NA | NA |
| 57 | 0.005 | 8 | 0.50 | **NA** | NA | NA | NA | NA | NA | NA |
| 58 | 0.01 | 8 | 0.50 | **NA** | NA | NA | NA | NA | NA | NA |
| 59 | 0.05 | 8 | 0.50 | **NA** | NA | NA | NA | NA | NA | NA |
| 60 | 0.0005 | 9 | 0.50 | **NA** | NA | NA | NA | NA | NA | NA |
| 61 | 0.001 | 9 | 0.50 | **NA** | NA | NA | NA | NA | NA | NA |
| 62 | 0.005 | 9 | 0.50 | **NA** | NA | NA | NA | NA | NA | NA |
| 63 | 0.01 | 9 | 0.50 | **NA** | NA | NA | NA | NA | NA | NA |
| 64 | 0.05 | 9 | 0.50 | **NA** | NA | NA | NA | NA | NA | NA |
| 65 | 0.0005 | 10 | 0.50 | **NA** | NA | NA | NA | NA | NA | NA |
| 66 | 0.001 | 10 | 0.50 | **NA** | NA | NA | NA | NA | NA | NA |
| 67 | 0.005 | 10 | 0.50 | **NA** | NA | NA | NA | NA | NA | NA |
| 68 | 0.01 | 10 | 0.50 | **NA** | NA | NA | NA | NA | NA | NA |
| 69 | 0.05 | 10 | 0.50 | **NA** | NA | NA | NA | NA | NA | NA |
| 70 | 0.0001 | 1 | 0.55 | **NA** | NA | NA | NA | NA | NA | NA |
| 71 | 0.0005 | 1 | 0.55 | **NA** | NA | NA | NA | NA | NA | NA |
| 72 | 0.001 | 1 | 0.55 | **NA** | NA | NA | NA | NA | NA | NA |
| 73 | 0.005 | 1 | 0.55 | **NA** | NA | NA | NA | NA | NA | NA |
| 74 | 0.01 | 1 | 0.55 | **NA** | NA | NA | NA | NA | NA | NA |
| 75 | 0.05 | 1 | 0.55 | **NA** | NA | NA | NA | NA | NA | NA |
| 76 | 0.0001 | 2 | 0.55 | **NA** | NA | NA | NA | NA | NA | NA |
| 77 | 0.0005 | 2 | 0.55 | **NA** | NA | NA | NA | NA | NA | NA |
| 78 | 0.001 | 2 | 0.55 | **NA** | NA | NA | NA | NA | NA | NA |
| 79 | 0.005 | 2 | 0.55 | **NA** | NA | NA | NA | NA | NA | NA |
| 80 | 0.01 | 2 | 0.55 | **NA** | NA | NA | NA | NA | NA | NA |
| 81 | 0.05 | 2 | 0.55 | **NA** | NA | NA | NA | NA | NA | NA |
| 82 | 0.0001 | 3 | 0.55 | **NA** | NA | NA | NA | NA | NA | NA |
| 83 | 0.0005 | 3 | 0.55 | **NA** | NA | NA | NA | NA | NA | NA |
| 84 | 0.001 | 3 | 0.55 | **NA** | NA | NA | NA | NA | NA | NA |
| 85 | 0.005 | 3 | 0.55 | **NA** | NA | NA | NA | NA | NA | NA |
| 86 | 0.01 | 3 | 0.55 | **NA** | NA | NA | NA | NA | NA | NA |
| 87 | 0.05 | 3 | 0.55 | **NA** | NA | NA | NA | NA | NA | NA |
| 88 | 0.0001 | 4 | 0.55 | **NA** | NA | NA | NA | NA | NA | NA |
| 89 | 0.0005 | 4 | 0.55 | **NA** | NA | NA | NA | NA | NA | NA |
| 90 | 0.001 | 4 | 0.55 | **NA** | NA | NA | NA | NA | NA | NA |
| 91 | 0.005 | 4 | 0.55 | **NA** | NA | NA | NA | NA | NA | NA |
| 92 | 0.01 | 4 | 0.55 | **NA** | NA | NA | NA | NA | NA | NA |
| 93 | 0.05 | 4 | 0.55 | **NA** | NA | NA | NA | NA | NA | NA |
| 94 | 0.0001 | 5 | 0.55 | **NA** | NA | NA | NA | NA | NA | NA |
| 95 | 0.0005 | 5 | 0.55 | **NA** | NA | NA | NA | NA | NA | NA |
| 96 | 0.001 | 5 | 0.55 | **NA** | NA | NA | NA | NA | NA | NA |
| 97 | 0.005 | 5 | 0.55 | **NA** | NA | NA | NA | NA | NA | NA |
| 98 | 0.01 | 5 | 0.55 | **NA** | NA | NA | NA | NA | NA | NA |
| 99 | 0.05 | 5 | 0.55 | **NA** | NA | NA | NA | NA | NA | NA |
| 100 | 0.0001 | 6 | 0.55 | **NA** | NA | NA | NA | NA | NA | NA |
| 101 | 0.0005 | 6 | 0.55 | **NA** | NA | NA | NA | NA | NA | NA |
| 102 | 0.001 | 6 | 0.55 | **NA** | NA | NA | NA | NA | NA | NA |
| 103 | 0.005 | 6 | 0.55 | **NA** | NA | NA | NA | NA | NA | NA |
| 104 | 0.01 | 6 | 0.55 | **NA** | NA | NA | NA | NA | NA | NA |
| 105 | 0.05 | 6 | 0.55 | **NA** | NA | NA | NA | NA | NA | NA |
| 106 | 0.0001 | 7 | 0.55 | **NA** | NA | NA | NA | NA | NA | NA |
| 107 | 0.0005 | 7 | 0.55 | **NA** | NA | NA | NA | NA | NA | NA |
| 108 | 0.001 | 7 | 0.55 | **NA** | NA | NA | NA | NA | NA | NA |
| 109 | 0.005 | 7 | 0.55 | **NA** | NA | NA | NA | NA | NA | NA |
| 110 | 0.01 | 7 | 0.55 | **NA** | NA | NA | NA | NA | NA | NA |
| 111 | 0.05 | 7 | 0.55 | **NA** | NA | NA | NA | NA | NA | NA |
| 112 | 0.0001 | 8 | 0.55 | **NA** | NA | NA | NA | NA | NA | NA |
| 113 | 0.0005 | 8 | 0.55 | **NA** | NA | NA | NA | NA | NA | NA |
| 114 | 0.001 | 8 | 0.55 | **NA** | NA | NA | NA | NA | NA | NA |
| 115 | 0.005 | 8 | 0.55 | **NA** | NA | NA | NA | NA | NA | NA |
| 116 | 0.01 | 8 | 0.55 | **NA** | NA | NA | NA | NA | NA | NA |
| 117 | 0.05 | 8 | 0.55 | **NA** | NA | NA | NA | NA | NA | NA |
| 118 | 0.0001 | 9 | 0.55 | **NA** | NA | NA | NA | NA | NA | NA |
| 119 | 0.0005 | 9 | 0.55 | **NA** | NA | NA | NA | NA | NA | NA |
| 120 | 0.001 | 9 | 0.55 | **NA** | NA | NA | NA | NA | NA | NA |
| 121 | 0.005 | 9 | 0.55 | **NA** | NA | NA | NA | NA | NA | NA |
| 122 | 0.01 | 9 | 0.55 | **NA** | NA | NA | NA | NA | NA | NA |
| 123 | 0.05 | 9 | 0.55 | **NA** | NA | NA | NA | NA | NA | NA |
| 124 | 0.0001 | 10 | 0.55 | **NA** | NA | NA | NA | NA | NA | NA |
| 125 | 0.0005 | 10 | 0.55 | **NA** | NA | NA | NA | NA | NA | NA |
| 126 | 0.001 | 10 | 0.55 | **NA** | NA | NA | NA | NA | NA | NA |
| 127 | 0.005 | 10 | 0.55 | **NA** | NA | NA | NA | NA | NA | NA |
| 128 | 0.01 | 10 | 0.55 | **NA** | NA | NA | NA | NA | NA | NA |
| 129 | 0.05 | 10 | 0.55 | **NA** | NA | NA | NA | NA | NA | NA |
| 130 | 0.0001 | 1 | 0.60 | **NA** | NA | NA | NA | NA | NA | NA |
| 131 | 0.0005 | 1 | 0.60 | **NA** | NA | NA | NA | NA | NA | NA |
| 132 | 0.001 | 1 | 0.60 | **NA** | NA | NA | NA | NA | NA | NA |
| 133 | 0.005 | 1 | 0.60 | **NA** | NA | NA | NA | NA | NA | NA |
| 134 | 0.01 | 1 | 0.60 | **NA** | NA | NA | NA | NA | NA | NA |
| 135 | 0.05 | 1 | 0.60 | **NA** | NA | NA | NA | NA | NA | NA |
| 136 | 0.0001 | 2 | 0.60 | **NA** | NA | NA | NA | NA | NA | NA |
| 137 | 0.0005 | 2 | 0.60 | **NA** | NA | NA | NA | NA | NA | NA |
| 138 | 0.001 | 2 | 0.60 | **NA** | NA | NA | NA | NA | NA | NA |
| 139 | 0.005 | 2 | 0.60 | **NA** | NA | NA | NA | NA | NA | NA |
| 140 | 0.01 | 2 | 0.60 | **NA** | NA | NA | NA | NA | NA | NA |
| 141 | 0.05 | 2 | 0.60 | **NA** | NA | NA | NA | NA | NA | NA |
| 142 | 0.0001 | 3 | 0.60 | **NA** | NA | NA | NA | NA | NA | NA |
| 143 | 0.0005 | 3 | 0.60 | **NA** | NA | NA | NA | NA | NA | NA |
| 144 | 0.001 | 3 | 0.60 | **NA** | NA | NA | NA | NA | NA | NA |
| 145 | 0.005 | 3 | 0.60 | **NA** | NA | NA | NA | NA | NA | NA |
| 146 | 0.01 | 3 | 0.60 | **NA** | NA | NA | NA | NA | NA | NA |
| 147 | 0.05 | 3 | 0.60 | **NA** | NA | NA | NA | NA | NA | NA |
| 148 | 0.0001 | 4 | 0.60 | **NA** | NA | NA | NA | NA | NA | NA |
| 149 | 0.0005 | 4 | 0.60 | **NA** | NA | NA | NA | NA | NA | NA |
| 150 | 0.001 | 4 | 0.60 | **NA** | NA | NA | NA | NA | NA | NA |
| 151 | 0.005 | 4 | 0.60 | **NA** | NA | NA | NA | NA | NA | NA |
| 152 | 0.01 | 4 | 0.60 | **NA** | NA | NA | NA | NA | NA | NA |
| 153 | 0.05 | 4 | 0.60 | **NA** | NA | NA | NA | NA | NA | NA |
| 154 | 0.0001 | 5 | 0.60 | **NA** | NA | NA | NA | NA | NA | NA |
| 155 | 0.0005 | 5 | 0.60 | **NA** | NA | NA | NA | NA | NA | NA |
| 156 | 0.001 | 5 | 0.60 | **NA** | NA | NA | NA | NA | NA | NA |
| 157 | 0.005 | 5 | 0.60 | **NA** | NA | NA | NA | NA | NA | NA |
| 158 | 0.01 | 5 | 0.60 | **NA** | NA | NA | NA | NA | NA | NA |
| 159 | 0.05 | 5 | 0.60 | **NA** | NA | NA | NA | NA | NA | NA |
| 160 | 0.0001 | 6 | 0.60 | **NA** | NA | NA | NA | NA | NA | NA |
| 161 | 0.0005 | 6 | 0.60 | **NA** | NA | NA | NA | NA | NA | NA |
| 162 | 0.001 | 6 | 0.60 | **NA** | NA | NA | NA | NA | NA | NA |
| 163 | 0.005 | 6 | 0.60 | **NA** | NA | NA | NA | NA | NA | NA |
| 164 | 0.01 | 6 | 0.60 | **NA** | NA | NA | NA | NA | NA | NA |
| 165 | 0.05 | 6 | 0.60 | **NA** | NA | NA | NA | NA | NA | NA |
| 166 | 0.0001 | 7 | 0.60 | **NA** | NA | NA | NA | NA | NA | NA |
| 167 | 0.0005 | 7 | 0.60 | **NA** | NA | NA | NA | NA | NA | NA |
| 168 | 0.001 | 7 | 0.60 | **NA** | NA | NA | NA | NA | NA | NA |
| 169 | 0.005 | 7 | 0.60 | **NA** | NA | NA | NA | NA | NA | NA |
| 170 | 0.01 | 7 | 0.60 | **NA** | NA | NA | NA | NA | NA | NA |
| 171 | 0.05 | 7 | 0.60 | **NA** | NA | NA | NA | NA | NA | NA |
| 172 | 0.0001 | 8 | 0.60 | **NA** | NA | NA | NA | NA | NA | NA |
| 173 | 0.0005 | 8 | 0.60 | **NA** | NA | NA | NA | NA | NA | NA |
| 174 | 0.001 | 8 | 0.60 | **NA** | NA | NA | NA | NA | NA | NA |
| 175 | 0.005 | 8 | 0.60 | **NA** | NA | NA | NA | NA | NA | NA |
| 176 | 0.01 | 8 | 0.60 | **NA** | NA | NA | NA | NA | NA | NA |
| 177 | 0.05 | 8 | 0.60 | **NA** | NA | NA | NA | NA | NA | NA |
| 178 | 0.0001 | 9 | 0.60 | **NA** | NA | NA | NA | NA | NA | NA |
| 179 | 0.0005 | 9 | 0.60 | **NA** | NA | NA | NA | NA | NA | NA |
| 180 | 0.001 | 9 | 0.60 | **NA** | NA | NA | NA | NA | NA | NA |
| 181 | 0.005 | 9 | 0.60 | **NA** | NA | NA | NA | NA | NA | NA |
| 182 | 0.01 | 9 | 0.60 | **NA** | NA | NA | NA | NA | NA | NA |
| 183 | 0.05 | 9 | 0.60 | **NA** | NA | NA | NA | NA | NA | NA |
| 184 | 0.0001 | 10 | 0.60 | **NA** | NA | NA | NA | NA | NA | NA |
| 185 | 0.0005 | 10 | 0.60 | **NA** | NA | NA | NA | NA | NA | NA |
| 186 | 0.001 | 10 | 0.60 | **NA** | NA | NA | NA | NA | NA | NA |
| 187 | 0.005 | 10 | 0.60 | **NA** | NA | NA | NA | NA | NA | NA |
| 188 | 0.01 | 10 | 0.60 | **NA** | NA | NA | NA | NA | NA | NA |
| 189 | 0.05 | 10 | 0.60 | **NA** | NA | NA | NA | NA | NA | NA |
| 190 | 0.0001 | 1 | 0.65 | **NA** | NA | NA | NA | NA | NA | NA |
| 191 | 0.0005 | 1 | 0.65 | **NA** | NA | NA | NA | NA | NA | NA |
| 192 | 0.001 | 1 | 0.65 | **NA** | NA | NA | NA | NA | NA | NA |
| 193 | 0.005 | 1 | 0.65 | **NA** | NA | NA | NA | NA | NA | NA |
| 194 | 0.01 | 1 | 0.65 | **NA** | NA | NA | NA | NA | NA | NA |
| 195 | 0.05 | 1 | 0.65 | **NA** | NA | NA | NA | NA | NA | NA |
| 196 | 0.0001 | 2 | 0.65 | **NA** | NA | NA | NA | NA | NA | NA |
| 197 | 0.0005 | 2 | 0.65 | **NA** | NA | NA | NA | NA | NA | NA |
| 198 | 0.001 | 2 | 0.65 | **NA** | NA | NA | NA | NA | NA | NA |
| 199 | 0.005 | 2 | 0.65 | **NA** | NA | NA | NA | NA | NA | NA |
| 200 | 0.01 | 2 | 0.65 | **NA** | NA | NA | NA | NA | NA | NA |
| 201 | 0.05 | 2 | 0.65 | **NA** | NA | NA | NA | NA | NA | NA |
| 202 | 0.0001 | 3 | 0.65 | **NA** | NA | NA | NA | NA | NA | NA |
| 203 | 0.0005 | 3 | 0.65 | **NA** | NA | NA | NA | NA | NA | NA |
| 204 | 0.001 | 3 | 0.65 | **NA** | NA | NA | NA | NA | NA | NA |
| 205 | 0.005 | 3 | 0.65 | **NA** | NA | NA | NA | NA | NA | NA |
| 206 | 0.01 | 3 | 0.65 | **NA** | NA | NA | NA | NA | NA | NA |
| 207 | 0.05 | 3 | 0.65 | **NA** | NA | NA | NA | NA | NA | NA |
| 208 | 0.0001 | 4 | 0.65 | **NA** | NA | NA | NA | NA | NA | NA |
| 209 | 0.0005 | 4 | 0.65 | **NA** | NA | NA | NA | NA | NA | NA |
| 210 | 0.001 | 4 | 0.65 | **NA** | NA | NA | NA | NA | NA | NA |
| 211 | 0.005 | 4 | 0.65 | **NA** | NA | NA | NA | NA | NA | NA |
| 212 | 0.01 | 4 | 0.65 | **NA** | NA | NA | NA | NA | NA | NA |
| 213 | 0.05 | 4 | 0.65 | **NA** | NA | NA | NA | NA | NA | NA |
| 214 | 0.0001 | 5 | 0.65 | **NA** | NA | NA | NA | NA | NA | NA |
| 215 | 0.0005 | 5 | 0.65 | **NA** | NA | NA | NA | NA | NA | NA |
| 216 | 0.001 | 5 | 0.65 | **NA** | NA | NA | NA | NA | NA | NA |
| 217 | 0.005 | 5 | 0.65 | **NA** | NA | NA | NA | NA | NA | NA |
| 218 | 0.01 | 5 | 0.65 | **NA** | NA | NA | NA | NA | NA | NA |
| 219 | 0.05 | 5 | 0.65 | **NA** | NA | NA | NA | NA | NA | NA |
| 220 | 0.0001 | 6 | 0.65 | **NA** | NA | NA | NA | NA | NA | NA |
| 221 | 0.0005 | 6 | 0.65 | **NA** | NA | NA | NA | NA | NA | NA |
| 222 | 0.001 | 6 | 0.65 | **NA** | NA | NA | NA | NA | NA | NA |
| 223 | 0.005 | 6 | 0.65 | **NA** | NA | NA | NA | NA | NA | NA |
| 224 | 0.01 | 6 | 0.65 | **NA** | NA | NA | NA | NA | NA | NA |
| 225 | 0.05 | 6 | 0.65 | **NA** | NA | NA | NA | NA | NA | NA |
| 226 | 0.0001 | 7 | 0.65 | **NA** | NA | NA | NA | NA | NA | NA |
| 227 | 0.0005 | 7 | 0.65 | **NA** | NA | NA | NA | NA | NA | NA |
| 228 | 0.001 | 7 | 0.65 | **NA** | NA | NA | NA | NA | NA | NA |
| 229 | 0.005 | 7 | 0.65 | **NA** | NA | NA | NA | NA | NA | NA |
| 230 | 0.01 | 7 | 0.65 | **NA** | NA | NA | NA | NA | NA | NA |
| 231 | 0.05 | 7 | 0.65 | **NA** | NA | NA | NA | NA | NA | NA |
| 232 | 0.0001 | 8 | 0.65 | **NA** | NA | NA | NA | NA | NA | NA |
| 233 | 0.0005 | 8 | 0.65 | **NA** | NA | NA | NA | NA | NA | NA |
| 234 | 0.001 | 8 | 0.65 | **NA** | NA | NA | NA | NA | NA | NA |
| 235 | 0.005 | 8 | 0.65 | **NA** | NA | NA | NA | NA | NA | NA |
| 236 | 0.01 | 8 | 0.65 | **NA** | NA | NA | NA | NA | NA | NA |
| 237 | 0.05 | 8 | 0.65 | **NA** | NA | NA | NA | NA | NA | NA |
| 238 | 0.0001 | 9 | 0.65 | **NA** | NA | NA | NA | NA | NA | NA |
| 239 | 0.0005 | 9 | 0.65 | **NA** | NA | NA | NA | NA | NA | NA |
| 240 | 0.001 | 9 | 0.65 | **NA** | NA | NA | NA | NA | NA | NA |
| 241 | 0.005 | 9 | 0.65 | **NA** | NA | NA | NA | NA | NA | NA |
| 242 | 0.01 | 9 | 0.65 | **NA** | NA | NA | NA | NA | NA | NA |
| 243 | 0.05 | 9 | 0.65 | **NA** | NA | NA | NA | NA | NA | NA |
| 244 | 0.0001 | 10 | 0.65 | **NA** | NA | NA | NA | NA | NA | NA |
| 245 | 0.0005 | 10 | 0.65 | **NA** | NA | NA | NA | NA | NA | NA |
| 246 | 0.001 | 10 | 0.65 | **NA** | NA | NA | NA | NA | NA | NA |
| 247 | 0.005 | 10 | 0.65 | **NA** | NA | NA | NA | NA | NA | NA |
| 248 | 0.01 | 10 | 0.65 | **NA** | NA | NA | NA | NA | NA | NA |
| 249 | 0.05 | 10 | 0.65 | **NA** | NA | NA | NA | NA | NA | NA |
| 250 | 0.0001 | 1 | 0.70 | **NA** | NA | NA | NA | NA | NA | NA |
| 251 | 0.0005 | 1 | 0.70 | **NA** | NA | NA | NA | NA | NA | NA |
| 252 | 0.001 | 1 | 0.70 | **NA** | NA | NA | NA | NA | NA | NA |
| 253 | 0.005 | 1 | 0.70 | **NA** | NA | NA | NA | NA | NA | NA |
| 254 | 0.01 | 1 | 0.70 | **NA** | NA | NA | NA | NA | NA | NA |
| 255 | 0.05 | 1 | 0.70 | **NA** | NA | NA | NA | NA | NA | NA |
| 256 | 0.0001 | 2 | 0.70 | **NA** | NA | NA | NA | NA | NA | NA |
| 257 | 0.0005 | 2 | 0.70 | **NA** | NA | NA | NA | NA | NA | NA |
| 258 | 0.001 | 2 | 0.70 | **NA** | NA | NA | NA | NA | NA | NA |
| 259 | 0.005 | 2 | 0.70 | **NA** | NA | NA | NA | NA | NA | NA |
| 260 | 0.01 | 2 | 0.70 | **NA** | NA | NA | NA | NA | NA | NA |
| 261 | 0.05 | 2 | 0.70 | **NA** | NA | NA | NA | NA | NA | NA |
| 262 | 0.0001 | 3 | 0.70 | **NA** | NA | NA | NA | NA | NA | NA |
| 263 | 0.0005 | 3 | 0.70 | **NA** | NA | NA | NA | NA | NA | NA |
| 264 | 0.001 | 3 | 0.70 | **NA** | NA | NA | NA | NA | NA | NA |
| 265 | 0.005 | 3 | 0.70 | **NA** | NA | NA | NA | NA | NA | NA |
| 266 | 0.01 | 3 | 0.70 | **NA** | NA | NA | NA | NA | NA | NA |
| 267 | 0.05 | 3 | 0.70 | **NA** | NA | NA | NA | NA | NA | NA |
| 268 | 0.0001 | 4 | 0.70 | **NA** | NA | NA | NA | NA | NA | NA |
| 269 | 0.0005 | 4 | 0.70 | **NA** | NA | NA | NA | NA | NA | NA |
| 270 | 0.001 | 4 | 0.70 | **NA** | NA | NA | NA | NA | NA | NA |
| 271 | 0.005 | 4 | 0.70 | **NA** | NA | NA | NA | NA | NA | NA |
| 272 | 0.01 | 4 | 0.70 | **NA** | NA | NA | NA | NA | NA | NA |
| 273 | 0.05 | 4 | 0.70 | **NA** | NA | NA | NA | NA | NA | NA |
| 274 | 0.0001 | 5 | 0.70 | **NA** | NA | NA | NA | NA | NA | NA |
| 275 | 0.0005 | 5 | 0.70 | **NA** | NA | NA | NA | NA | NA | NA |
| 276 | 0.001 | 5 | 0.70 | **NA** | NA | NA | NA | NA | NA | NA |
| 277 | 0.005 | 5 | 0.70 | **NA** | NA | NA | NA | NA | NA | NA |
| 278 | 0.01 | 5 | 0.70 | **NA** | NA | NA | NA | NA | NA | NA |
| 279 | 0.05 | 5 | 0.70 | **NA** | NA | NA | NA | NA | NA | NA |
| 280 | 0.0001 | 6 | 0.70 | **NA** | NA | NA | NA | NA | NA | NA |
| 281 | 0.0005 | 6 | 0.70 | **NA** | NA | NA | NA | NA | NA | NA |
| 282 | 0.001 | 6 | 0.70 | **NA** | NA | NA | NA | NA | NA | NA |
| 283 | 0.005 | 6 | 0.70 | **NA** | NA | NA | NA | NA | NA | NA |
| 284 | 0.01 | 6 | 0.70 | **NA** | NA | NA | NA | NA | NA | NA |
| 285 | 0.05 | 6 | 0.70 | **NA** | NA | NA | NA | NA | NA | NA |
| 286 | 0.0001 | 7 | 0.70 | **NA** | NA | NA | NA | NA | NA | NA |
| 287 | 0.0005 | 7 | 0.70 | **NA** | NA | NA | NA | NA | NA | NA |
| 288 | 0.001 | 7 | 0.70 | **NA** | NA | NA | NA | NA | NA | NA |
| 289 | 0.005 | 7 | 0.70 | **NA** | NA | NA | NA | NA | NA | NA |
| 290 | 0.01 | 7 | 0.70 | **NA** | NA | NA | NA | NA | NA | NA |
| 291 | 0.05 | 7 | 0.70 | **NA** | NA | NA | NA | NA | NA | NA |
| 292 | 0.0001 | 8 | 0.70 | **NA** | NA | NA | NA | NA | NA | NA |
| 293 | 0.0005 | 8 | 0.70 | **NA** | NA | NA | NA | NA | NA | NA |
| 294 | 0.001 | 8 | 0.70 | **NA** | NA | NA | NA | NA | NA | NA |
| 295 | 0.005 | 8 | 0.70 | **NA** | NA | NA | NA | NA | NA | NA |
| 296 | 0.01 | 8 | 0.70 | **NA** | NA | NA | NA | NA | NA | NA |
| 297 | 0.05 | 8 | 0.70 | **NA** | NA | NA | NA | NA | NA | NA |
| 298 | 0.0001 | 9 | 0.70 | **NA** | NA | NA | NA | NA | NA | NA |
| 299 | 0.0005 | 9 | 0.70 | **NA** | NA | NA | NA | NA | NA | NA |
| 300 | 0.001 | 9 | 0.70 | **NA** | NA | NA | NA | NA | NA | NA |
| 301 | 0.005 | 9 | 0.70 | **NA** | NA | NA | NA | NA | NA | NA |
| 302 | 0.01 | 9 | 0.70 | **NA** | NA | NA | NA | NA | NA | NA |
| 303 | 0.05 | 9 | 0.70 | **NA** | NA | NA | NA | NA | NA | NA |
| 304 | 0.0001 | 10 | 0.70 | **NA** | NA | NA | NA | NA | NA | NA |
| 305 | 0.0005 | 10 | 0.70 | **NA** | NA | NA | NA | NA | NA | NA |
| 306 | 0.001 | 10 | 0.70 | **NA** | NA | NA | NA | NA | NA | NA |
| 307 | 0.005 | 10 | 0.70 | **NA** | NA | NA | NA | NA | NA | NA |
| 308 | 0.01 | 10 | 0.70 | **NA** | NA | NA | NA | NA | NA | NA |
| 309 | 0.05 | 10 | 0.70 | **NA** | NA | NA | NA | NA | NA | NA |
| 310 | 0.0001 | 1 | 0.75 | **NA** | NA | NA | NA | NA | NA | NA |
| 311 | 0.0005 | 1 | 0.75 | **NA** | NA | NA | NA | NA | NA | NA |
| 312 | 0.001 | 1 | 0.75 | **NA** | NA | NA | NA | NA | NA | NA |
| 313 | 0.005 | 1 | 0.75 | **NA** | NA | NA | NA | NA | NA | NA |
| 314 | 0.01 | 1 | 0.75 | **NA** | NA | NA | NA | NA | NA | NA |
| 315 | 0.05 | 1 | 0.75 | **NA** | NA | NA | NA | NA | NA | NA |
| 316 | 0.0005 | 2 | 0.75 | **NA** | NA | NA | NA | NA | NA | NA |
| 317 | 0.001 | 2 | 0.75 | **NA** | NA | NA | NA | NA | NA | NA |
| 318 | 0.005 | 2 | 0.75 | **NA** | NA | NA | NA | NA | NA | NA |
| 319 | 0.01 | 2 | 0.75 | **NA** | NA | NA | NA | NA | NA | NA |
| 320 | 0.05 | 2 | 0.75 | **NA** | NA | NA | NA | NA | NA | NA |
| 321 | 0.0005 | 3 | 0.75 | **NA** | NA | NA | NA | NA | NA | NA |
| 322 | 0.001 | 3 | 0.75 | **NA** | NA | NA | NA | NA | NA | NA |
| 323 | 0.005 | 3 | 0.75 | **NA** | NA | NA | NA | NA | NA | NA |
| 324 | 0.01 | 3 | 0.75 | **NA** | NA | NA | NA | NA | NA | NA |
| 325 | 0.05 | 3 | 0.75 | **NA** | NA | NA | NA | NA | NA | NA |
| 326 | 0.0005 | 4 | 0.75 | **NA** | NA | NA | NA | NA | NA | NA |
| 327 | 0.001 | 4 | 0.75 | **NA** | NA | NA | NA | NA | NA | NA |
| 328 | 0.005 | 4 | 0.75 | **NA** | NA | NA | NA | NA | NA | NA |
| 329 | 0.01 | 4 | 0.75 | **NA** | NA | NA | NA | NA | NA | NA |
| 330 | 0.05 | 4 | 0.75 | **NA** | NA | NA | NA | NA | NA | NA |
| 331 | 0.0005 | 5 | 0.75 | **NA** | NA | NA | NA | NA | NA | NA |
| 332 | 0.001 | 5 | 0.75 | **NA** | NA | NA | NA | NA | NA | NA |
| 333 | 0.005 | 5 | 0.75 | **NA** | NA | NA | NA | NA | NA | NA |
| 334 | 0.01 | 5 | 0.75 | **NA** | NA | NA | NA | NA | NA | NA |
| 335 | 0.05 | 5 | 0.75 | **NA** | NA | NA | NA | NA | NA | NA |
| 336 | 0.0005 | 6 | 0.75 | **NA** | NA | NA | NA | NA | NA | NA |
| 337 | 0.001 | 6 | 0.75 | **NA** | NA | NA | NA | NA | NA | NA |
| 338 | 0.005 | 6 | 0.75 | **NA** | NA | NA | NA | NA | NA | NA |
| 339 | 0.01 | 6 | 0.75 | **NA** | NA | NA | NA | NA | NA | NA |
| 340 | 0.05 | 6 | 0.75 | **NA** | NA | NA | NA | NA | NA | NA |
| 341 | 0.0005 | 7 | 0.75 | **NA** | NA | NA | NA | NA | NA | NA |
| 342 | 0.001 | 7 | 0.75 | **NA** | NA | NA | NA | NA | NA | NA |
| 343 | 0.005 | 7 | 0.75 | **NA** | NA | NA | NA | NA | NA | NA |
| 344 | 0.01 | 7 | 0.75 | **NA** | NA | NA | NA | NA | NA | NA |
| 345 | 0.05 | 7 | 0.75 | **NA** | NA | NA | NA | NA | NA | NA |
| 346 | 0.0005 | 8 | 0.75 | **NA** | NA | NA | NA | NA | NA | NA |
| 347 | 0.001 | 8 | 0.75 | **NA** | NA | NA | NA | NA | NA | NA |
| 348 | 0.005 | 8 | 0.75 | **NA** | NA | NA | NA | NA | NA | NA |
| 349 | 0.01 | 8 | 0.75 | **NA** | NA | NA | NA | NA | NA | NA |
| 350 | 0.05 | 8 | 0.75 | **NA** | NA | NA | NA | NA | NA | NA |
| 351 | 0.0005 | 9 | 0.75 | **NA** | NA | NA | NA | NA | NA | NA |
| 352 | 0.001 | 9 | 0.75 | **NA** | NA | NA | NA | NA | NA | NA |
| 353 | 0.005 | 9 | 0.75 | **NA** | NA | NA | NA | NA | NA | NA |
| 354 | 0.01 | 9 | 0.75 | **NA** | NA | NA | NA | NA | NA | NA |
| 355 | 0.05 | 9 | 0.75 | **NA** | NA | NA | NA | NA | NA | NA |
| 356 | 0.0005 | 10 | 0.75 | **NA** | NA | NA | NA | NA | NA | NA |
| 357 | 0.001 | 10 | 0.75 | **NA** | NA | NA | NA | NA | NA | NA |
| 358 | 0.005 | 10 | 0.75 | **NA** | NA | NA | NA | NA | NA | NA |
| 359 | 0.01 | 10 | 0.75 | **NA** | NA | NA | NA | NA | NA | NA |
| 360 | 0.05 | 10 | 0.75 | **NA** | NA | NA | NA | NA | NA | NA |

**Table S7.** Possible combinations of the three meta-parameters (learning rate, tree complexity, bag fraction) for fitted 360 BRT models for species richness at 0.25 m^2^ spatial scale based on 10-fold cross validation (CV) and the associated model performance parameters. Numbers are sorted according to increasing CV deviance (in bold). RMSE represents root mean square error, and MAE represents mean absolute error.

| No. | Learning rate | Tree complexity | Bag fraction | **CV deviance** | Percentage of explained deviance (%) | CV correlation | Training data correlation | RMSE  (%) | MAE  (%) | *R*^2^ |
| --- | --- | --- | --- | --- | --- | --- | --- | --- | --- | --- |
| 1 | 0.005 | 1 | 0.75 | **6.986** | 10.656 | 0.232 | 0.567 | 2.390 | 1.934 | 0.270 |
| 2 | 0.005 | 1 | 0.70 | **6.988** | 10.622 | 0.236 | 0.563 | 2.395 | 1.941 | 0.266 |
| 3 | 0.001 | 1 | 0.75 | **6.991** | 10.593 | 0.222 | 0.576 | 2.368 | 1.923 | 0.283 |
| 4 | 0.0005 | 1 | 0.75 | **6.993** | 10.566 | 0.220 | 0.576 | 2.359 | 1.919 | 0.288 |
| 5 | 0.005 | 1 | 0.65 | **6.995** | 10.533 | 0.242 | 0.557 | 2.399 | 1.947 | 0.264 |
| 6 | 0.0001 | 1 | 0.75 | **7.003** | 10.431 | 0.228 | 0.566 | 2.401 | 1.944 | 0.263 |
| 7 | 0.005 | 2 | 0.65 | **7.016** | 10.272 | 0.248 | 0.621 | 2.300 | 1.867 | 0.324 |
| 8 | 0.005 | 3 | 0.65 | **7.016** | 10.272 | 0.248 | 0.621 | 2.300 | 1.867 | 0.324 |
| 9 | 0.005 | 4 | 0.65 | **7.016** | 10.272 | 0.248 | 0.621 | 2.300 | 1.867 | 0.324 |
| 10 | 0.005 | 5 | 0.65 | **7.016** | 10.272 | 0.248 | 0.621 | 2.300 | 1.867 | 0.324 |
| 11 | 0.005 | 6 | 0.65 | **7.016** | 10.272 | 0.248 | 0.621 | 2.300 | 1.867 | 0.324 |
| 12 | 0.005 | 7 | 0.65 | **7.016** | 10.272 | 0.248 | 0.621 | 2.300 | 1.867 | 0.324 |
| 13 | 0.005 | 8 | 0.65 | **7.016** | 10.272 | 0.248 | 0.621 | 2.300 | 1.867 | 0.324 |
| 14 | 0.005 | 9 | 0.65 | **7.016** | 10.272 | 0.248 | 0.621 | 2.300 | 1.867 | 0.324 |
| 15 | 0.005 | 10 | 0.65 | **7.016** | 10.272 | 0.248 | 0.621 | 2.300 | 1.867 | 0.324 |
| 16 | 0.0005 | 1 | 0.70 | **7.021** | 10.206 | 0.229 | 0.568 | 2.385 | 1.938 | 0.272 |
| 17 | 0.001 | 1 | 0.70 | **7.032** | 10.069 | 0.224 | 0.572 | 2.369 | 1.928 | 0.282 |
| 18 | 0.0001 | 1 | 0.70 | **7.034** | 10.039 | 0.231 | 0.562 | 2.410 | 1.953 | 0.257 |
| 19 | 0.0005 | 1 | 0.65 | **7.038** | 9.982 | 0.233 | 0.560 | 2.399 | 1.951 | 0.264 |
| 20 | 0.005 | 1 | 0.60 | **7.044** | 9.913 | 0.242 | 0.557 | 2.404 | 1.952 | 0.261 |
| 21 | 0.005 | 2 | 0.60 | **7.049** | 9.850 | 0.242 | 0.602 | 2.343 | 1.905 | 0.298 |
| 22 | 0.005 | 3 | 0.60 | **7.049** | 9.850 | 0.242 | 0.602 | 2.343 | 1.905 | 0.298 |
| 23 | 0.005 | 4 | 0.60 | **7.049** | 9.850 | 0.242 | 0.602 | 2.343 | 1.905 | 0.298 |
| 24 | 0.005 | 5 | 0.60 | **7.049** | 9.850 | 0.242 | 0.602 | 2.343 | 1.905 | 0.298 |
| 25 | 0.005 | 6 | 0.60 | **7.049** | 9.850 | 0.242 | 0.602 | 2.343 | 1.905 | 0.298 |
| 26 | 0.005 | 7 | 0.60 | **7.049** | 9.850 | 0.242 | 0.602 | 2.343 | 1.905 | 0.298 |
| 27 | 0.005 | 8 | 0.60 | **7.049** | 9.850 | 0.242 | 0.602 | 2.343 | 1.905 | 0.298 |
| 28 | 0.005 | 9 | 0.60 | **7.049** | 9.850 | 0.242 | 0.602 | 2.343 | 1.905 | 0.298 |
| 29 | 0.005 | 10 | 0.60 | **7.049** | 9.850 | 0.242 | 0.602 | 2.343 | 1.905 | 0.298 |
| 30 | 0.0001 | 1 | 0.65 | **7.049** | 9.850 | 0.238 | 0.555 | 2.418 | 1.961 | 0.252 |
| 31 | 0.005 | 1 | 0.50 | **7.050** | 9.834 | 0.246 | 0.560 | 2.367 | 1.935 | 0.283 |
| 32 | 0.005 | 2 | 0.50 | **7.050** | 9.834 | 0.246 | 0.560 | 2.367 | 1.935 | 0.283 |
| 33 | 0.005 | 3 | 0.50 | **7.050** | 9.834 | 0.246 | 0.560 | 2.367 | 1.935 | 0.283 |
| 34 | 0.005 | 4 | 0.50 | **7.050** | 9.834 | 0.246 | 0.560 | 2.367 | 1.935 | 0.283 |
| 35 | 0.005 | 5 | 0.50 | **7.050** | 9.834 | 0.246 | 0.560 | 2.367 | 1.935 | 0.283 |
| 36 | 0.005 | 6 | 0.50 | **7.050** | 9.834 | 0.246 | 0.560 | 2.367 | 1.935 | 0.283 |
| 37 | 0.005 | 7 | 0.50 | **7.050** | 9.834 | 0.246 | 0.560 | 2.367 | 1.935 | 0.283 |
| 38 | 0.005 | 8 | 0.50 | **7.050** | 9.834 | 0.246 | 0.560 | 2.367 | 1.935 | 0.283 |
| 39 | 0.005 | 9 | 0.50 | **7.050** | 9.834 | 0.246 | 0.560 | 2.367 | 1.935 | 0.283 |
| 40 | 0.005 | 10 | 0.50 | **7.050** | 9.834 | 0.246 | 0.560 | 2.367 | 1.935 | 0.283 |
| 41 | 0.001 | 1 | 0.65 | **7.050** | 9.841 | 0.236 | 0.562 | 2.392 | 1.944 | 0.268 |
| 42 | 0.001 | 1 | 0.60 | **7.060** | 9.708 | 0.244 | 0.557 | 2.401 | 1.953 | 0.263 |
| 43 | 0.0005 | 1 | 0.60 | **7.061** | 9.699 | 0.238 | 0.555 | 2.406 | 1.958 | 0.260 |
| 44 | 0.0005 | 2 | 0.65 | **7.062** | 9.686 | 0.240 | 0.621 | 2.305 | 1.875 | 0.320 |
| 45 | 0.0005 | 3 | 0.65 | **7.062** | 9.686 | 0.240 | 0.621 | 2.305 | 1.875 | 0.320 |
| 46 | 0.0005 | 4 | 0.65 | **7.062** | 9.686 | 0.240 | 0.621 | 2.305 | 1.875 | 0.320 |
| 47 | 0.0005 | 5 | 0.65 | **7.062** | 9.686 | 0.240 | 0.621 | 2.305 | 1.875 | 0.320 |
| 48 | 0.0005 | 6 | 0.65 | **7.062** | 9.686 | 0.240 | 0.621 | 2.305 | 1.875 | 0.320 |
| 49 | 0.0005 | 7 | 0.65 | **7.062** | 9.686 | 0.240 | 0.621 | 2.305 | 1.875 | 0.320 |
| 50 | 0.0005 | 8 | 0.65 | **7.062** | 9.686 | 0.240 | 0.621 | 2.305 | 1.875 | 0.320 |
| 51 | 0.0005 | 9 | 0.65 | **7.062** | 9.686 | 0.240 | 0.621 | 2.305 | 1.875 | 0.320 |
| 52 | 0.0005 | 10 | 0.65 | **7.062** | 9.686 | 0.240 | 0.621 | 2.305 | 1.875 | 0.320 |
| 53 | 0.01 | 1 | 0.50 | **7.063** | 9.665 | 0.271 | 0.576 | 2.327 | 1.909 | 0.308 |
| 54 | 0.01 | 2 | 0.50 | **7.063** | 9.665 | 0.271 | 0.576 | 2.327 | 1.909 | 0.308 |
| 55 | 0.01 | 3 | 0.50 | **7.063** | 9.665 | 0.271 | 0.576 | 2.327 | 1.909 | 0.308 |
| 56 | 0.01 | 4 | 0.50 | **7.063** | 9.665 | 0.271 | 0.576 | 2.327 | 1.909 | 0.308 |
| 57 | 0.01 | 5 | 0.50 | **7.063** | 9.665 | 0.271 | 0.576 | 2.327 | 1.909 | 0.308 |
| 58 | 0.01 | 6 | 0.50 | **7.063** | 9.665 | 0.271 | 0.576 | 2.327 | 1.909 | 0.308 |
| 59 | 0.01 | 7 | 0.50 | **7.063** | 9.665 | 0.271 | 0.576 | 2.327 | 1.909 | 0.308 |
| 60 | 0.01 | 8 | 0.50 | **7.063** | 9.665 | 0.271 | 0.576 | 2.327 | 1.909 | 0.308 |
| 61 | 0.01 | 9 | 0.50 | **7.063** | 9.665 | 0.271 | 0.576 | 2.327 | 1.909 | 0.308 |
| 62 | 0.01 | 10 | 0.50 | **7.063** | 9.665 | 0.271 | 0.576 | 2.327 | 1.909 | 0.308 |
| 63 | 0.001 | 2 | 0.65 | **7.064** | 9.652 | 0.241 | 0.622 | 2.296 | 1.870 | 0.326 |
| 64 | 0.001 | 3 | 0.65 | **7.064** | 9.652 | 0.241 | 0.622 | 2.296 | 1.870 | 0.326 |
| 65 | 0.001 | 4 | 0.65 | **7.064** | 9.652 | 0.241 | 0.622 | 2.296 | 1.870 | 0.326 |
| 66 | 0.001 | 5 | 0.65 | **7.064** | 9.652 | 0.241 | 0.622 | 2.296 | 1.870 | 0.326 |
| 67 | 0.001 | 6 | 0.65 | **7.064** | 9.652 | 0.241 | 0.622 | 2.296 | 1.870 | 0.326 |
| 68 | 0.001 | 7 | 0.65 | **7.064** | 9.652 | 0.241 | 0.622 | 2.296 | 1.870 | 0.326 |
| 69 | 0.001 | 8 | 0.65 | **7.064** | 9.652 | 0.241 | 0.622 | 2.296 | 1.870 | 0.326 |
| 70 | 0.001 | 9 | 0.65 | **7.064** | 9.652 | 0.241 | 0.622 | 2.296 | 1.870 | 0.326 |
| 71 | 0.001 | 10 | 0.65 | **7.064** | 9.652 | 0.241 | 0.622 | 2.296 | 1.870 | 0.326 |
| 72 | 0.001 | 2 | 0.60 | **7.065** | 9.646 | 0.245 | 0.597 | 2.340 | 1.904 | 0.300 |
| 73 | 0.001 | 3 | 0.60 | **7.065** | 9.646 | 0.245 | 0.597 | 2.340 | 1.904 | 0.300 |
| 74 | 0.001 | 4 | 0.60 | **7.065** | 9.646 | 0.245 | 0.597 | 2.340 | 1.904 | 0.300 |
| 75 | 0.001 | 5 | 0.60 | **7.065** | 9.646 | 0.245 | 0.597 | 2.340 | 1.904 | 0.300 |
| 76 | 0.001 | 6 | 0.60 | **7.065** | 9.646 | 0.245 | 0.597 | 2.340 | 1.904 | 0.300 |
| 77 | 0.001 | 7 | 0.60 | **7.065** | 9.646 | 0.245 | 0.597 | 2.340 | 1.904 | 0.300 |
| 78 | 0.001 | 8 | 0.60 | **7.065** | 9.646 | 0.245 | 0.597 | 2.340 | 1.904 | 0.300 |
| 79 | 0.001 | 9 | 0.60 | **7.065** | 9.646 | 0.245 | 0.597 | 2.340 | 1.904 | 0.300 |
| 80 | 0.001 | 10 | 0.60 | **7.065** | 9.646 | 0.245 | 0.597 | 2.340 | 1.904 | 0.300 |
| 81 | 0.0005 | 2 | 0.60 | **7.072** | 9.560 | 0.241 | 0.607 | 2.307 | 1.884 | 0.319 |
| 82 | 0.0005 | 3 | 0.60 | **7.072** | 9.560 | 0.241 | 0.607 | 2.307 | 1.884 | 0.319 |
| 83 | 0.0005 | 4 | 0.60 | **7.072** | 9.560 | 0.241 | 0.607 | 2.307 | 1.884 | 0.319 |
| 84 | 0.0005 | 5 | 0.60 | **7.072** | 9.560 | 0.241 | 0.607 | 2.307 | 1.884 | 0.319 |
| 85 | 0.0005 | 6 | 0.60 | **7.072** | 9.560 | 0.241 | 0.607 | 2.307 | 1.884 | 0.319 |
| 86 | 0.0005 | 7 | 0.60 | **7.072** | 9.560 | 0.241 | 0.607 | 2.307 | 1.884 | 0.319 |
| 87 | 0.0005 | 8 | 0.60 | **7.072** | 9.560 | 0.241 | 0.607 | 2.307 | 1.884 | 0.319 |
| 88 | 0.0005 | 9 | 0.60 | **7.072** | 9.560 | 0.241 | 0.607 | 2.307 | 1.884 | 0.319 |
| 89 | 0.0005 | 10 | 0.60 | **7.072** | 9.560 | 0.241 | 0.607 | 2.307 | 1.884 | 0.319 |
| 90 | 0.0001 | 2 | 0.65 | **7.072** | 9.557 | 0.242 | 0.614 | 2.329 | 1.892 | 0.306 |
| 91 | 0.0001 | 3 | 0.65 | **7.072** | 9.557 | 0.242 | 0.614 | 2.329 | 1.892 | 0.306 |
| 92 | 0.0001 | 4 | 0.65 | **7.072** | 9.557 | 0.242 | 0.614 | 2.329 | 1.892 | 0.306 |
| 93 | 0.0001 | 5 | 0.65 | **7.072** | 9.557 | 0.242 | 0.614 | 2.329 | 1.892 | 0.306 |
| 94 | 0.0001 | 6 | 0.65 | **7.072** | 9.557 | 0.242 | 0.614 | 2.329 | 1.892 | 0.306 |
| 95 | 0.0001 | 7 | 0.65 | **7.072** | 9.557 | 0.242 | 0.614 | 2.329 | 1.892 | 0.306 |
| 96 | 0.0001 | 8 | 0.65 | **7.072** | 9.557 | 0.242 | 0.614 | 2.329 | 1.892 | 0.306 |
| 97 | 0.0001 | 9 | 0.65 | **7.072** | 9.557 | 0.242 | 0.614 | 2.329 | 1.892 | 0.306 |
| 98 | 0.0001 | 10 | 0.65 | **7.072** | 9.557 | 0.242 | 0.614 | 2.329 | 1.892 | 0.306 |
| 99 | 0.005 | 1 | 0.55 | **7.073** | 9.547 | 0.252 | 0.545 | 2.418 | 1.966 | 0.252 |
| 100 | 0.005 | 2 | 0.55 | **7.073** | 9.547 | 0.252 | 0.557 | 2.403 | 1.952 | 0.261 |
| 101 | 0.005 | 3 | 0.55 | **7.073** | 9.547 | 0.252 | 0.557 | 2.403 | 1.952 | 0.261 |
| 102 | 0.005 | 4 | 0.55 | **7.073** | 9.547 | 0.252 | 0.557 | 2.403 | 1.952 | 0.261 |
| 103 | 0.005 | 5 | 0.55 | **7.073** | 9.547 | 0.252 | 0.557 | 2.403 | 1.952 | 0.261 |
| 104 | 0.005 | 6 | 0.55 | **7.073** | 9.547 | 0.252 | 0.557 | 2.403 | 1.952 | 0.261 |
| 105 | 0.005 | 7 | 0.55 | **7.073** | 9.547 | 0.252 | 0.557 | 2.403 | 1.952 | 0.261 |
| 106 | 0.005 | 8 | 0.55 | **7.073** | 9.547 | 0.252 | 0.557 | 2.403 | 1.952 | 0.261 |
| 107 | 0.005 | 9 | 0.55 | **7.073** | 9.547 | 0.252 | 0.557 | 2.403 | 1.952 | 0.261 |
| 108 | 0.005 | 10 | 0.55 | **7.073** | 9.547 | 0.252 | 0.557 | 2.403 | 1.952 | 0.261 |
| 109 | 0.0005 | 1 | 0.55 | **7.074** | 9.527 | 0.241 | 0.555 | 2.394 | 1.954 | 0.267 |
| 110 | 0.0005 | 2 | 0.55 | **7.074** | 9.527 | 0.241 | 0.569 | 2.375 | 1.939 | 0.279 |
| 111 | 0.0005 | 3 | 0.55 | **7.074** | 9.527 | 0.241 | 0.569 | 2.375 | 1.939 | 0.279 |
| 112 | 0.0005 | 4 | 0.55 | **7.074** | 9.527 | 0.241 | 0.569 | 2.375 | 1.939 | 0.279 |
| 113 | 0.0005 | 5 | 0.55 | **7.074** | 9.527 | 0.241 | 0.569 | 2.375 | 1.939 | 0.279 |
| 114 | 0.0005 | 6 | 0.55 | **7.074** | 9.527 | 0.241 | 0.569 | 2.375 | 1.939 | 0.279 |
| 115 | 0.0005 | 7 | 0.55 | **7.074** | 9.527 | 0.241 | 0.569 | 2.375 | 1.939 | 0.279 |
| 116 | 0.0005 | 8 | 0.55 | **7.074** | 9.527 | 0.241 | 0.569 | 2.375 | 1.939 | 0.279 |
| 117 | 0.0005 | 9 | 0.55 | **7.074** | 9.527 | 0.241 | 0.569 | 2.375 | 1.939 | 0.279 |
| 118 | 0.0005 | 10 | 0.55 | **7.074** | 9.527 | 0.241 | 0.569 | 2.375 | 1.939 | 0.279 |
| 119 | 0.001 | 1 | 0.55 | **7.076** | 9.501 | 0.241 | 0.559 | 2.384 | 1.947 | 0.273 |
| 120 | 0.001 | 2 | 0.55 | **7.076** | 9.501 | 0.241 | 0.573 | 2.365 | 1.933 | 0.285 |
| 121 | 0.001 | 3 | 0.55 | **7.076** | 9.501 | 0.241 | 0.573 | 2.365 | 1.933 | 0.285 |
| 122 | 0.001 | 4 | 0.55 | **7.076** | 9.501 | 0.241 | 0.573 | 2.365 | 1.933 | 0.285 |
| 123 | 0.001 | 5 | 0.55 | **7.076** | 9.501 | 0.241 | 0.573 | 2.365 | 1.933 | 0.285 |
| 124 | 0.001 | 6 | 0.55 | **7.076** | 9.501 | 0.241 | 0.573 | 2.365 | 1.933 | 0.285 |
| 125 | 0.001 | 7 | 0.55 | **7.076** | 9.501 | 0.241 | 0.573 | 2.365 | 1.933 | 0.285 |
| 126 | 0.001 | 8 | 0.55 | **7.076** | 9.501 | 0.241 | 0.573 | 2.365 | 1.933 | 0.285 |
| 127 | 0.001 | 9 | 0.55 | **7.076** | 9.501 | 0.241 | 0.573 | 2.365 | 1.933 | 0.285 |
| 128 | 0.001 | 10 | 0.55 | **7.076** | 9.501 | 0.241 | 0.573 | 2.365 | 1.933 | 0.285 |
| 129 | 0.0005 | 2 | 0.70 | **7.076** | 9.503 | 0.230 | 0.634 | 2.273 | 1.853 | 0.339 |
| 130 | 0.0005 | 3 | 0.70 | **7.076** | 9.503 | 0.230 | 0.634 | 2.273 | 1.853 | 0.339 |
| 131 | 0.0005 | 4 | 0.70 | **7.076** | 9.503 | 0.230 | 0.634 | 2.273 | 1.853 | 0.339 |
| 132 | 0.0005 | 5 | 0.70 | **7.076** | 9.503 | 0.230 | 0.634 | 2.273 | 1.853 | 0.339 |
| 133 | 0.0005 | 6 | 0.70 | **7.076** | 9.503 | 0.230 | 0.634 | 2.273 | 1.853 | 0.339 |
| 134 | 0.0005 | 7 | 0.70 | **7.076** | 9.503 | 0.230 | 0.634 | 2.273 | 1.853 | 0.339 |
| 135 | 0.0005 | 8 | 0.70 | **7.076** | 9.503 | 0.230 | 0.634 | 2.273 | 1.853 | 0.339 |
| 136 | 0.0005 | 9 | 0.70 | **7.076** | 9.503 | 0.230 | 0.634 | 2.273 | 1.853 | 0.339 |
| 137 | 0.0005 | 10 | 0.70 | **7.076** | 9.503 | 0.230 | 0.634 | 2.273 | 1.853 | 0.339 |
| 138 | 0.001 | 2 | 0.70 | **7.079** | 9.466 | 0.232 | 0.629 | 2.302 | 1.872 | 0.322 |
| 139 | 0.001 | 3 | 0.70 | **7.079** | 9.466 | 0.232 | 0.629 | 2.302 | 1.872 | 0.322 |
| 140 | 0.001 | 4 | 0.70 | **7.079** | 9.466 | 0.232 | 0.629 | 2.302 | 1.872 | 0.322 |
| 141 | 0.001 | 5 | 0.70 | **7.079** | 9.466 | 0.232 | 0.629 | 2.302 | 1.872 | 0.322 |
| 142 | 0.001 | 6 | 0.70 | **7.079** | 9.466 | 0.232 | 0.629 | 2.302 | 1.872 | 0.322 |
| 143 | 0.001 | 7 | 0.70 | **7.079** | 9.466 | 0.232 | 0.629 | 2.302 | 1.872 | 0.322 |
| 144 | 0.001 | 8 | 0.70 | **7.079** | 9.466 | 0.232 | 0.629 | 2.302 | 1.872 | 0.322 |
| 145 | 0.001 | 9 | 0.70 | **7.079** | 9.466 | 0.232 | 0.629 | 2.302 | 1.872 | 0.322 |
| 146 | 0.001 | 10 | 0.70 | **7.079** | 9.466 | 0.232 | 0.629 | 2.302 | 1.872 | 0.322 |
| 147 | 0.0001 | 2 | 0.70 | **7.084** | 9.404 | 0.233 | 0.623 | 2.322 | 1.887 | 0.311 |
| 148 | 0.0001 | 3 | 0.70 | **7.084** | 9.404 | 0.233 | 0.623 | 2.322 | 1.887 | 0.311 |
| 149 | 0.0001 | 4 | 0.70 | **7.084** | 9.404 | 0.233 | 0.623 | 2.322 | 1.887 | 0.311 |
| 150 | 0.0001 | 5 | 0.70 | **7.084** | 9.404 | 0.233 | 0.623 | 2.322 | 1.887 | 0.311 |
| 151 | 0.0001 | 6 | 0.70 | **7.084** | 9.404 | 0.233 | 0.623 | 2.322 | 1.887 | 0.311 |
| 152 | 0.0001 | 7 | 0.70 | **7.084** | 9.404 | 0.233 | 0.623 | 2.322 | 1.887 | 0.311 |
| 153 | 0.0001 | 8 | 0.70 | **7.084** | 9.404 | 0.233 | 0.623 | 2.322 | 1.887 | 0.311 |
| 154 | 0.0001 | 9 | 0.70 | **7.084** | 9.404 | 0.233 | 0.623 | 2.322 | 1.887 | 0.311 |
| 155 | 0.0001 | 10 | 0.70 | **7.084** | 9.404 | 0.233 | 0.623 | 2.322 | 1.887 | 0.311 |
| 156 | 0.001 | 2 | 0.75 | **7.085** | 9.393 | 0.224 | 0.629 | 2.310 | 1.880 | 0.317 |
| 157 | 0.001 | 3 | 0.75 | **7.085** | 9.393 | 0.224 | 0.637 | 2.288 | 1.863 | 0.330 |
| 158 | 0.001 | 4 | 0.75 | **7.085** | 9.393 | 0.224 | 0.637 | 2.288 | 1.863 | 0.330 |
| 159 | 0.001 | 5 | 0.75 | **7.085** | 9.393 | 0.224 | 0.637 | 2.288 | 1.863 | 0.330 |
| 160 | 0.001 | 6 | 0.75 | **7.085** | 9.393 | 0.224 | 0.637 | 2.288 | 1.863 | 0.330 |
| 161 | 0.001 | 7 | 0.75 | **7.085** | 9.393 | 0.224 | 0.637 | 2.288 | 1.863 | 0.330 |
| 162 | 0.001 | 8 | 0.75 | **7.085** | 9.393 | 0.224 | 0.637 | 2.288 | 1.863 | 0.330 |
| 163 | 0.001 | 9 | 0.75 | **7.085** | 9.393 | 0.224 | 0.637 | 2.288 | 1.863 | 0.330 |
| 164 | 0.001 | 10 | 0.75 | **7.085** | 9.393 | 0.224 | 0.637 | 2.288 | 1.863 | 0.330 |
| 165 | 0.0001 | 1 | 0.60 | **7.092** | 9.295 | 0.240 | 0.546 | 2.437 | 1.976 | 0.240 |
| 166 | 0.0005 | 2 | 0.75 | **7.093** | 9.290 | 0.224 | 0.634 | 2.281 | 1.862 | 0.335 |
| 167 | 0.0005 | 3 | 0.75 | **7.093** | 9.290 | 0.224 | 0.643 | 2.258 | 1.845 | 0.348 |
| 168 | 0.0005 | 4 | 0.75 | **7.093** | 9.290 | 0.224 | 0.643 | 2.258 | 1.845 | 0.348 |
| 169 | 0.0005 | 5 | 0.75 | **7.093** | 9.290 | 0.224 | 0.643 | 2.258 | 1.845 | 0.348 |
| 170 | 0.0005 | 6 | 0.75 | **7.093** | 9.290 | 0.224 | 0.643 | 2.258 | 1.845 | 0.348 |
| 171 | 0.0005 | 7 | 0.75 | **7.093** | 9.290 | 0.224 | 0.643 | 2.258 | 1.845 | 0.348 |
| 172 | 0.0005 | 8 | 0.75 | **7.093** | 9.290 | 0.224 | 0.643 | 2.258 | 1.845 | 0.348 |
| 173 | 0.0005 | 9 | 0.75 | **7.093** | 9.290 | 0.224 | 0.643 | 2.258 | 1.845 | 0.348 |
| 174 | 0.0005 | 10 | 0.75 | **7.093** | 9.290 | 0.224 | 0.643 | 2.258 | 1.845 | 0.348 |
| 175 | 0.0001 | 1 | 0.55 | **7.098** | 9.221 | 0.243 | 0.539 | 2.443 | 1.982 | 0.236 |
| 176 | 0.0001 | 2 | 0.55 | **7.098** | 9.221 | 0.243 | 0.551 | 2.429 | 1.970 | 0.245 |
| 177 | 0.0001 | 3 | 0.55 | **7.098** | 9.221 | 0.243 | 0.551 | 2.429 | 1.970 | 0.245 |
| 178 | 0.0001 | 4 | 0.55 | **7.098** | 9.221 | 0.243 | 0.551 | 2.429 | 1.970 | 0.245 |
| 179 | 0.0001 | 5 | 0.55 | **7.098** | 9.221 | 0.243 | 0.551 | 2.429 | 1.970 | 0.245 |
| 180 | 0.0001 | 6 | 0.55 | **7.098** | 9.221 | 0.243 | 0.551 | 2.429 | 1.970 | 0.245 |
| 181 | 0.0001 | 7 | 0.55 | **7.098** | 9.221 | 0.243 | 0.551 | 2.429 | 1.970 | 0.245 |
| 182 | 0.0001 | 8 | 0.55 | **7.098** | 9.221 | 0.243 | 0.551 | 2.429 | 1.970 | 0.245 |
| 183 | 0.0001 | 9 | 0.55 | **7.098** | 9.221 | 0.243 | 0.551 | 2.429 | 1.970 | 0.245 |
| 184 | 0.0001 | 10 | 0.55 | **7.098** | 9.221 | 0.243 | 0.551 | 2.429 | 1.970 | 0.245 |
| 185 | 0.0001 | 2 | 0.75 | **7.098** | 9.223 | 0.225 | 0.63 | 2.308 | 1.880 | 0.319 |
| 186 | 0.0001 | 3 | 0.75 | **7.098** | 9.223 | 0.225 | 0.639 | 2.285 | 1.863 | 0.332 |
| 187 | 0.0001 | 4 | 0.75 | **7.098** | 9.223 | 0.225 | 0.639 | 2.285 | 1.863 | 0.332 |
| 188 | 0.0001 | 5 | 0.75 | **7.098** | 9.223 | 0.225 | 0.639 | 2.285 | 1.863 | 0.332 |
| 189 | 0.0001 | 6 | 0.75 | **7.098** | 9.223 | 0.225 | 0.639 | 2.285 | 1.863 | 0.332 |
| 190 | 0.0001 | 7 | 0.75 | **7.098** | 9.223 | 0.225 | 0.639 | 2.285 | 1.863 | 0.332 |
| 191 | 0.0001 | 8 | 0.75 | **7.098** | 9.223 | 0.225 | 0.639 | 2.285 | 1.863 | 0.332 |
| 192 | 0.0001 | 9 | 0.75 | **7.098** | 9.223 | 0.225 | 0.639 | 2.285 | 1.863 | 0.332 |
| 193 | 0.0001 | 10 | 0.75 | **7.098** | 9.223 | 0.225 | 0.639 | 2.285 | 1.863 | 0.332 |
| 194 | 0.005 | 2 | 0.70 | **7.101** | 9.186 | 0.227 | 0.628 | 2.279 | 1.855 | 0.336 |
| 195 | 0.005 | 3 | 0.70 | **7.101** | 9.186 | 0.227 | 0.628 | 2.279 | 1.855 | 0.336 |
| 196 | 0.005 | 4 | 0.70 | **7.101** | 9.186 | 0.227 | 0.628 | 2.279 | 1.855 | 0.336 |
| 197 | 0.005 | 5 | 0.70 | **7.101** | 9.186 | 0.227 | 0.628 | 2.279 | 1.855 | 0.336 |
| 198 | 0.005 | 6 | 0.70 | **7.101** | 9.186 | 0.227 | 0.628 | 2.279 | 1.855 | 0.336 |
| 199 | 0.005 | 7 | 0.70 | **7.101** | 9.186 | 0.227 | 0.628 | 2.279 | 1.855 | 0.336 |
| 200 | 0.005 | 8 | 0.70 | **7.101** | 9.186 | 0.227 | 0.628 | 2.279 | 1.855 | 0.336 |
| 201 | 0.005 | 9 | 0.70 | **7.101** | 9.186 | 0.227 | 0.628 | 2.279 | 1.855 | 0.336 |
| 202 | 0.005 | 10 | 0.70 | **7.101** | 9.186 | 0.227 | 0.628 | 2.279 | 1.855 | 0.336 |
| 203 | 0.0001 | 2 | 0.60 | **7.103** | 9.157 | 0.238 | 0.587 | 2.384 | 1.932 | 0.273 |
| 204 | 0.0001 | 3 | 0.60 | **7.103** | 9.157 | 0.238 | 0.587 | 2.384 | 1.932 | 0.273 |
| 205 | 0.0001 | 4 | 0.60 | **7.103** | 9.157 | 0.238 | 0.587 | 2.384 | 1.932 | 0.273 |
| 206 | 0.0001 | 5 | 0.60 | **7.103** | 9.157 | 0.238 | 0.587 | 2.384 | 1.932 | 0.273 |
| 207 | 0.0001 | 6 | 0.60 | **7.103** | 9.157 | 0.238 | 0.587 | 2.384 | 1.932 | 0.273 |
| 208 | 0.0001 | 7 | 0.60 | **7.103** | 9.157 | 0.238 | 0.587 | 2.384 | 1.932 | 0.273 |
| 209 | 0.0001 | 8 | 0.60 | **7.103** | 9.157 | 0.238 | 0.587 | 2.384 | 1.932 | 0.273 |
| 210 | 0.0001 | 9 | 0.60 | **7.103** | 9.157 | 0.238 | 0.587 | 2.384 | 1.932 | 0.273 |
| 211 | 0.0001 | 10 | 0.60 | **7.103** | 9.157 | 0.238 | 0.587 | 2.384 | 1.932 | 0.273 |
| 212 | 0.0005 | 1 | 0.50 | **7.107** | 9.104 | 0.241 | 0.550 | 2.401 | 1.959 | 0.263 |
| 213 | 0.0005 | 2 | 0.50 | **7.107** | 9.104 | 0.241 | 0.550 | 2.401 | 1.959 | 0.263 |
| 214 | 0.0005 | 3 | 0.50 | **7.107** | 9.104 | 0.241 | 0.550 | 2.401 | 1.959 | 0.263 |
| 215 | 0.0005 | 4 | 0.50 | **7.107** | 9.104 | 0.241 | 0.550 | 2.401 | 1.959 | 0.263 |
| 216 | 0.0005 | 5 | 0.50 | **7.107** | 9.104 | 0.241 | 0.550 | 2.401 | 1.959 | 0.263 |
| 217 | 0.0005 | 6 | 0.50 | **7.107** | 9.104 | 0.241 | 0.550 | 2.401 | 1.959 | 0.263 |
| 218 | 0.0005 | 7 | 0.50 | **7.107** | 9.104 | 0.241 | 0.550 | 2.401 | 1.959 | 0.263 |
| 219 | 0.0005 | 8 | 0.50 | **7.107** | 9.104 | 0.241 | 0.550 | 2.401 | 1.959 | 0.263 |
| 220 | 0.0005 | 9 | 0.50 | **7.107** | 9.104 | 0.241 | 0.550 | 2.401 | 1.959 | 0.263 |
| 221 | 0.0005 | 10 | 0.50 | **7.107** | 9.104 | 0.241 | 0.550 | 2.401 | 1.959 | 0.263 |
| 222 | 0.001 | 1 | 0.50 | **7.114** | 9.020 | 0.237 | 0.548 | 2.399 | 1.956 | 0.264 |
| 223 | 0.001 | 2 | 0.50 | **7.114** | 9.020 | 0.237 | 0.548 | 2.399 | 1.956 | 0.264 |
| 224 | 0.001 | 3 | 0.50 | **7.114** | 9.020 | 0.237 | 0.548 | 2.399 | 1.956 | 0.264 |
| 225 | 0.001 | 4 | 0.50 | **7.114** | 9.020 | 0.237 | 0.548 | 2.399 | 1.956 | 0.264 |
| 226 | 0.001 | 5 | 0.50 | **7.114** | 9.020 | 0.237 | 0.548 | 2.399 | 1.956 | 0.264 |
| 227 | 0.001 | 6 | 0.50 | **7.114** | 9.020 | 0.237 | 0.548 | 2.399 | 1.956 | 0.264 |
| 228 | 0.001 | 7 | 0.50 | **7.114** | 9.020 | 0.237 | 0.548 | 2.399 | 1.956 | 0.264 |
| 229 | 0.001 | 8 | 0.50 | **7.114** | 9.020 | 0.237 | 0.548 | 2.399 | 1.956 | 0.264 |
| 230 | 0.001 | 9 | 0.50 | **7.114** | 9.020 | 0.237 | 0.548 | 2.399 | 1.956 | 0.264 |
| 231 | 0.001 | 10 | 0.50 | **7.114** | 9.020 | 0.237 | 0.548 | 2.399 | 1.956 | 0.264 |
| 232 | 0.005 | 2 | 0.75 | **7.122** | 8.911 | 0.217 | 0.642 | 2.259 | 1.840 | 0.348 |
| 233 | 0.005 | 3 | 0.75 | **7.122** | 8.911 | 0.217 | 0.648 | 2.240 | 1.828 | 0.358 |
| 234 | 0.005 | 4 | 0.75 | **7.122** | 8.911 | 0.217 | 0.648 | 2.240 | 1.828 | 0.358 |
| 235 | 0.005 | 5 | 0.75 | **7.122** | 8.911 | 0.217 | 0.648 | 2.240 | 1.828 | 0.358 |
| 236 | 0.005 | 6 | 0.75 | **7.122** | 8.911 | 0.217 | 0.648 | 2.240 | 1.828 | 0.358 |
| 237 | 0.005 | 7 | 0.75 | **7.122** | 8.911 | 0.217 | 0.648 | 2.240 | 1.828 | 0.358 |
| 238 | 0.005 | 8 | 0.75 | **7.122** | 8.911 | 0.217 | 0.648 | 2.240 | 1.828 | 0.358 |
| 239 | 0.005 | 9 | 0.75 | **7.122** | 8.911 | 0.217 | 0.648 | 2.240 | 1.828 | 0.358 |
| 240 | 0.005 | 10 | 0.75 | **7.122** | 8.911 | 0.217 | 0.648 | 2.240 | 1.828 | 0.358 |
| 241 | 0.0001 | 1 | 0.50 | **7.132** | 8.782 | 0.244 | 0.531 | 2.462 | 1.996 | 0.225 |
| 242 | 0.0001 | 2 | 0.50 | **7.132** | 8.782 | 0.244 | 0.531 | 2.462 | 1.996 | 0.225 |
| 243 | 0.0001 | 3 | 0.50 | **7.132** | 8.782 | 0.244 | 0.531 | 2.462 | 1.996 | 0.225 |
| 244 | 0.0001 | 4 | 0.50 | **7.132** | 8.782 | 0.244 | 0.531 | 2.462 | 1.996 | 0.225 |
| 245 | 0.0001 | 5 | 0.50 | **7.132** | 8.782 | 0.244 | 0.531 | 2.462 | 1.996 | 0.225 |
| 246 | 0.0001 | 6 | 0.50 | **7.132** | 8.782 | 0.244 | 0.531 | 2.462 | 1.996 | 0.225 |
| 247 | 0.0001 | 7 | 0.50 | **7.132** | 8.782 | 0.244 | 0.531 | 2.462 | 1.996 | 0.225 |
| 248 | 0.0001 | 8 | 0.50 | **7.132** | 8.782 | 0.244 | 0.531 | 2.462 | 1.996 | 0.225 |
| 249 | 0.0001 | 9 | 0.50 | **7.132** | 8.782 | 0.244 | 0.531 | 2.462 | 1.996 | 0.225 |
| 250 | 0.0001 | 10 | 0.50 | **7.132** | 8.782 | 0.244 | 0.531 | 2.462 | 1.996 | 0.225 |
| 251 | 0.05 | 1 | 0.50 | **NA** | NA | NA | NA | NA | NA | NA |
| 252 | 0.05 | 2 | 0.50 | **NA** | NA | NA | NA | NA | NA | NA |
| 253 | 0.05 | 3 | 0.50 | **NA** | NA | NA | NA | NA | NA | NA |
| 254 | 0.05 | 4 | 0.50 | **NA** | NA | NA | NA | NA | NA | NA |
| 255 | 0.05 | 5 | 0.50 | **NA** | NA | NA | NA | NA | NA | NA |
| 256 | 0.05 | 6 | 0.50 | **NA** | NA | NA | NA | NA | NA | NA |
| 257 | 0.05 | 7 | 0.50 | **NA** | NA | NA | NA | NA | NA | NA |
| 258 | 0.05 | 8 | 0.50 | **NA** | NA | NA | NA | NA | NA | NA |
| 259 | 0.05 | 9 | 0.50 | **NA** | NA | NA | NA | NA | NA | NA |
| 260 | 0.05 | 10 | 0.50 | **NA** | NA | NA | NA | NA | NA | NA |
| 261 | 0.01 | 1 | 0.55 | **NA** | NA | NA | NA | NA | NA | NA |
| 262 | 0.05 | 1 | 0.55 | **NA** | NA | NA | NA | NA | NA | NA |
| 263 | 0.01 | 2 | 0.55 | **NA** | NA | NA | NA | NA | NA | NA |
| 264 | 0.05 | 2 | 0.55 | **NA** | NA | NA | NA | NA | NA | NA |
| 265 | 0.01 | 3 | 0.55 | **NA** | NA | NA | NA | NA | NA | NA |
| 266 | 0.05 | 3 | 0.55 | **NA** | NA | NA | NA | NA | NA | NA |
| 267 | 0.01 | 4 | 0.55 | **NA** | NA | NA | NA | NA | NA | NA |
| 268 | 0.05 | 4 | 0.55 | **NA** | NA | NA | NA | NA | NA | NA |
| 269 | 0.01 | 5 | 0.55 | **NA** | NA | NA | NA | NA | NA | NA |
| 270 | 0.05 | 5 | 0.55 | **NA** | NA | NA | NA | NA | NA | NA |
| 271 | 0.01 | 6 | 0.55 | **NA** | NA | NA | NA | NA | NA | NA |
| 272 | 0.05 | 6 | 0.55 | **NA** | NA | NA | NA | NA | NA | NA |
| 273 | 0.01 | 7 | 0.55 | **NA** | NA | NA | NA | NA | NA | NA |
| 274 | 0.05 | 7 | 0.55 | **NA** | NA | NA | NA | NA | NA | NA |
| 275 | 0.01 | 8 | 0.55 | **NA** | NA | NA | NA | NA | NA | NA |
| 276 | 0.05 | 8 | 0.55 | **NA** | NA | NA | NA | NA | NA | NA |
| 277 | 0.01 | 9 | 0.55 | **NA** | NA | NA | NA | NA | NA | NA |
| 278 | 0.05 | 9 | 0.55 | **NA** | NA | NA | NA | NA | NA | NA |
| 279 | 0.01 | 10 | 0.55 | **NA** | NA | NA | NA | NA | NA | NA |
| 280 | 0.05 | 10 | 0.55 | **NA** | NA | NA | NA | NA | NA | NA |
| 281 | 0.01 | 1 | 0.60 | **NA** | NA | NA | NA | NA | NA | NA |
| 282 | 0.05 | 1 | 0.60 | **NA** | NA | NA | NA | NA | NA | NA |
| 283 | 0.01 | 2 | 0.60 | **NA** | NA | NA | NA | NA | NA | NA |
| 284 | 0.05 | 2 | 0.60 | **NA** | NA | NA | NA | NA | NA | NA |
| 285 | 0.01 | 3 | 0.60 | **NA** | NA | NA | NA | NA | NA | NA |
| 286 | 0.05 | 3 | 0.60 | **NA** | NA | NA | NA | NA | NA | NA |
| 287 | 0.01 | 4 | 0.60 | **NA** | NA | NA | NA | NA | NA | NA |
| 288 | 0.05 | 4 | 0.60 | **NA** | NA | NA | NA | NA | NA | NA |
| 289 | 0.01 | 5 | 0.60 | **NA** | NA | NA | NA | NA | NA | NA |
| 290 | 0.05 | 5 | 0.60 | **NA** | NA | NA | NA | NA | NA | NA |
| 291 | 0.01 | 6 | 0.60 | **NA** | NA | NA | NA | NA | NA | NA |
| 292 | 0.05 | 6 | 0.60 | **NA** | NA | NA | NA | NA | NA | NA |
| 293 | 0.01 | 7 | 0.60 | **NA** | NA | NA | NA | NA | NA | NA |
| 294 | 0.05 | 7 | 0.60 | **NA** | NA | NA | NA | NA | NA | NA |
| 295 | 0.01 | 8 | 0.60 | **NA** | NA | NA | NA | NA | NA | NA |
| 296 | 0.05 | 8 | 0.60 | **NA** | NA | NA | NA | NA | NA | NA |
| 297 | 0.01 | 9 | 0.60 | **NA** | NA | NA | NA | NA | NA | NA |
| 298 | 0.05 | 9 | 0.60 | **NA** | NA | NA | NA | NA | NA | NA |
| 299 | 0.01 | 10 | 0.60 | **NA** | NA | NA | NA | NA | NA | NA |
| 300 | 0.05 | 10 | 0.60 | **NA** | NA | NA | NA | NA | NA | NA |
| 301 | 0.01 | 1 | 0.65 | **NA** | NA | NA | NA | NA | NA | NA |
| 302 | 0.05 | 1 | 0.65 | **NA** | NA | NA | NA | NA | NA | NA |
| 303 | 0.01 | 2 | 0.65 | **NA** | NA | NA | NA | NA | NA | NA |
| 304 | 0.05 | 2 | 0.65 | **NA** | NA | NA | NA | NA | NA | NA |
| 305 | 0.01 | 3 | 0.65 | **NA** | NA | NA | NA | NA | NA | NA |
| 306 | 0.05 | 3 | 0.65 | **NA** | NA | NA | NA | NA | NA | NA |
| 307 | 0.01 | 4 | 0.65 | **NA** | NA | NA | NA | NA | NA | NA |
| 308 | 0.05 | 4 | 0.65 | **NA** | NA | NA | NA | NA | NA | NA |
| 309 | 0.01 | 5 | 0.65 | **NA** | NA | NA | NA | NA | NA | NA |
| 310 | 0.05 | 5 | 0.65 | **NA** | NA | NA | NA | NA | NA | NA |
| 311 | 0.01 | 6 | 0.65 | **NA** | NA | NA | NA | NA | NA | NA |
| 312 | 0.05 | 6 | 0.65 | **NA** | NA | NA | NA | NA | NA | NA |
| 313 | 0.01 | 7 | 0.65 | **NA** | NA | NA | NA | NA | NA | NA |
| 314 | 0.05 | 7 | 0.65 | **NA** | NA | NA | NA | NA | NA | NA |
| 315 | 0.01 | 8 | 0.65 | **NA** | NA | NA | NA | NA | NA | NA |
| 316 | 0.05 | 8 | 0.65 | **NA** | NA | NA | NA | NA | NA | NA |
| 317 | 0.01 | 9 | 0.65 | **NA** | NA | NA | NA | NA | NA | NA |
| 318 | 0.05 | 9 | 0.65 | **NA** | NA | NA | NA | NA | NA | NA |
| 319 | 0.01 | 10 | 0.65 | **NA** | NA | NA | NA | NA | NA | NA |
| 320 | 0.05 | 10 | 0.65 | **NA** | NA | NA | NA | NA | NA | NA |
| 321 | 0.01 | 1 | 0.70 | **NA** | NA | NA | NA | NA | NA | NA |
| 322 | 0.05 | 1 | 0.70 | **NA** | NA | NA | NA | NA | NA | NA |
| 323 | 0.01 | 2 | 0.70 | **NA** | NA | NA | NA | NA | NA | NA |
| 324 | 0.05 | 2 | 0.70 | **NA** | NA | NA | NA | NA | NA | NA |
| 325 | 0.01 | 3 | 0.70 | **NA** | NA | NA | NA | NA | NA | NA |
| 326 | 0.05 | 3 | 0.70 | **NA** | NA | NA | NA | NA | NA | NA |
| 327 | 0.01 | 4 | 0.70 | **NA** | NA | NA | NA | NA | NA | NA |
| 328 | 0.05 | 4 | 0.70 | **NA** | NA | NA | NA | NA | NA | NA |
| 329 | 0.01 | 5 | 0.70 | **NA** | NA | NA | NA | NA | NA | NA |
| 330 | 0.05 | 5 | 0.70 | **NA** | NA | NA | NA | NA | NA | NA |
| 331 | 0.01 | 6 | 0.70 | **NA** | NA | NA | NA | NA | NA | NA |
| 332 | 0.05 | 6 | 0.70 | **NA** | NA | NA | NA | NA | NA | NA |
| 333 | 0.01 | 7 | 0.70 | **NA** | NA | NA | NA | NA | NA | NA |
| 334 | 0.05 | 7 | 0.70 | **NA** | NA | NA | NA | NA | NA | NA |
| 335 | 0.01 | 8 | 0.70 | **NA** | NA | NA | NA | NA | NA | NA |
| 336 | 0.05 | 8 | 0.70 | **NA** | NA | NA | NA | NA | NA | NA |
| 337 | 0.01 | 9 | 0.70 | **NA** | NA | NA | NA | NA | NA | NA |
| 338 | 0.05 | 9 | 0.70 | **NA** | NA | NA | NA | NA | NA | NA |
| 339 | 0.01 | 10 | 0.70 | **NA** | NA | NA | NA | NA | NA | NA |
| 340 | 0.05 | 10 | 0.70 | **NA** | NA | NA | NA | NA | NA | NA |
| 341 | 0.01 | 1 | 0.75 | **NA** | NA | NA | NA | NA | NA | NA |
| 342 | 0.05 | 1 | 0.75 | **NA** | NA | NA | NA | NA | NA | NA |
| 343 | 0.01 | 2 | 0.75 | **NA** | NA | NA | NA | NA | NA | NA |
| 344 | 0.05 | 2 | 0.75 | **NA** | NA | NA | NA | NA | NA | NA |
| 345 | 0.01 | 3 | 0.75 | **NA** | NA | NA | NA | NA | NA | NA |
| 346 | 0.05 | 3 | 0.75 | **NA** | NA | NA | NA | NA | NA | NA |
| 347 | 0.01 | 4 | 0.75 | **NA** | NA | NA | NA | NA | NA | NA |
| 348 | 0.05 | 4 | 0.75 | **NA** | NA | NA | NA | NA | NA | NA |
| 349 | 0.01 | 5 | 0.75 | **NA** | NA | NA | NA | NA | NA | NA |
| 350 | 0.05 | 5 | 0.75 | **NA** | NA | NA | NA | NA | NA | NA |
| 351 | 0.01 | 6 | 0.75 | **NA** | NA | NA | NA | NA | NA | NA |
| 352 | 0.05 | 6 | 0.75 | **NA** | NA | NA | NA | NA | NA | NA |
| 353 | 0.01 | 7 | 0.75 | **NA** | NA | NA | NA | NA | NA | NA |
| 354 | 0.05 | 7 | 0.75 | **NA** | NA | NA | NA | NA | NA | NA |
| 355 | 0.01 | 8 | 0.75 | **NA** | NA | NA | NA | NA | NA | NA |
| 356 | 0.05 | 8 | 0.75 | **NA** | NA | NA | NA | NA | NA | NA |
| 357 | 0.01 | 9 | 0.75 | **NA** | NA | NA | NA | NA | NA | NA |
| 358 | 0.05 | 9 | 0.75 | **NA** | NA | NA | NA | NA | NA | NA |
| 359 | 0.01 | 10 | 0.75 | **NA** | NA | NA | NA | NA | NA | NA |
| 360 | 0.05 | 10 | 0.75 | **NA** | NA | NA | NA | NA | NA | NA |

**Table S8.** Possible combinations of the three meta-parameters (learning rate, tree complexity, bag fraction) for fitted 360 BRT models for species richness at 1 m^2^ spatial scale based on 10-fold cross validation (CV) and the associated model performance parameters. Numbers are sorted according to increasing CV deviance (in bold). RMSE represents root mean square error, and MAE represents mean absolute error.

| No. | Learning rate | Tree complexity | Bag fraction | **CV deviance** | Percentage of explained deviance (%) | CV correlation | Training data correlation | RMSE  (%) | MAE  (%) | *R*^2^ |
| --- | --- | --- | --- | --- | --- | --- | --- | --- | --- | --- |
| 1 | 0.01 | 1 | 0.75 | **10.716** | 26.713 | 0.452 | 0.858 | 1.992 | 1.644 | 0.690 |
| 2 | 0.005 | 1 | 0.75 | **10.750** | 26.482 | 0.437 | 0.845 | 2.087 | 1.727 | 0.664 |
| 3 | 0.05 | 2 | 0.65 | **10.771** | 26.335 | 0.439 | 0.852 | 2.044 | 1.648 | 0.676 |
| 4 | 0.05 | 3 | 0.65 | **10.771** | 26.335 | 0.439 | 0.852 | 2.044 | 1.648 | 0.676 |
| 5 | 0.05 | 4 | 0.65 | **10.771** | 26.335 | 0.439 | 0.852 | 2.044 | 1.648 | 0.676 |
| 6 | 0.05 | 5 | 0.65 | **10.771** | 26.335 | 0.439 | 0.852 | 2.044 | 1.648 | 0.676 |
| 7 | 0.05 | 6 | 0.65 | **10.771** | 26.335 | 0.439 | 0.852 | 2.044 | 1.648 | 0.676 |
| 8 | 0.05 | 7 | 0.65 | **10.771** | 26.335 | 0.439 | 0.852 | 2.044 | 1.648 | 0.676 |
| 9 | 0.05 | 8 | 0.65 | **10.771** | 26.335 | 0.439 | 0.852 | 2.044 | 1.648 | 0.676 |
| 10 | 0.05 | 9 | 0.65 | **10.771** | 26.335 | 0.439 | 0.852 | 2.044 | 1.648 | 0.676 |
| 11 | 0.05 | 10 | 0.65 | **10.771** | 26.335 | 0.439 | 0.852 | 2.044 | 1.648 | 0.676 |
| 12 | 0.005 | 1 | 0.70 | **10.892** | 25.513 | 0.445 | 0.853 | 2.031 | 1.655 | 0.680 |
| 13 | 0.01 | 1 | 0.70 | **10.928** | 25.260 | 0.445 | 0.855 | 2.015 | 1.638 | 0.683 |
| 14 | 0.01 | 2 | 0.70 | **11.042** | 24.483 | 0.409 | 0.853 | 2.039 | 1.666 | 0.678 |
| 15 | 0.01 | 3 | 0.70 | **11.042** | 24.483 | 0.409 | 0.853 | 2.039 | 1.666 | 0.678 |
| 16 | 0.01 | 4 | 0.70 | **11.042** | 24.483 | 0.409 | 0.853 | 2.039 | 1.666 | 0.678 |
| 17 | 0.01 | 5 | 0.70 | **11.042** | 24.483 | 0.409 | 0.853 | 2.039 | 1.666 | 0.678 |
| 18 | 0.01 | 6 | 0.70 | **11.042** | 24.483 | 0.409 | 0.853 | 2.039 | 1.666 | 0.678 |
| 19 | 0.01 | 7 | 0.70 | **11.042** | 24.483 | 0.409 | 0.853 | 2.039 | 1.666 | 0.678 |
| 20 | 0.01 | 8 | 0.70 | **11.042** | 24.483 | 0.409 | 0.853 | 2.039 | 1.666 | 0.678 |
| 21 | 0.01 | 9 | 0.70 | **11.042** | 24.483 | 0.409 | 0.853 | 2.039 | 1.666 | 0.678 |
| 22 | 0.01 | 10 | 0.70 | **11.042** | 24.483 | 0.409 | 0.853 | 2.039 | 1.666 | 0.678 |
| 23 | 0.005 | 2 | 0.75 | **11.058** | 24.372 | 0.402 | 0.844 | 2.097 | 1.736 | 0.662 |
| 24 | 0.005 | 3 | 0.75 | **11.058** | 24.372 | 0.402 | 0.847 | 2.071 | 1.715 | 0.669 |
| 25 | 0.005 | 4 | 0.75 | **11.058** | 24.372 | 0.402 | 0.847 | 2.071 | 1.715 | 0.669 |
| 26 | 0.005 | 5 | 0.75 | **11.058** | 24.372 | 0.402 | 0.847 | 2.071 | 1.715 | 0.669 |
| 27 | 0.005 | 6 | 0.75 | **11.058** | 24.372 | 0.402 | 0.847 | 2.071 | 1.715 | 0.669 |
| 28 | 0.005 | 7 | 0.75 | **11.058** | 24.372 | 0.402 | 0.847 | 2.071 | 1.715 | 0.669 |
| 29 | 0.005 | 8 | 0.75 | **11.058** | 24.372 | 0.402 | 0.847 | 2.071 | 1.715 | 0.669 |
| 30 | 0.005 | 9 | 0.75 | **11.058** | 24.372 | 0.402 | 0.847 | 2.071 | 1.715 | 0.669 |
| 31 | 0.005 | 10 | 0.75 | **11.058** | 24.372 | 0.402 | 0.847 | 2.071 | 1.715 | 0.669 |
| 32 | 0.01 | 2 | 0.75 | **11.130** | 23.881 | 0.398 | 0.848 | 2.066 | 1.706 | 0.670 |
| 33 | 0.01 | 3 | 0.75 | **11.130** | 23.881 | 0.398 | 0.853 | 2.036 | 1.686 | 0.679 |
| 34 | 0.01 | 4 | 0.75 | **11.130** | 23.881 | 0.398 | 0.853 | 2.036 | 1.686 | 0.679 |
| 35 | 0.01 | 5 | 0.75 | **11.130** | 23.881 | 0.398 | 0.853 | 2.036 | 1.686 | 0.679 |
| 36 | 0.01 | 6 | 0.75 | **11.130** | 23.881 | 0.398 | 0.853 | 2.036 | 1.686 | 0.679 |
| 37 | 0.01 | 7 | 0.75 | **11.130** | 23.881 | 0.398 | 0.853 | 2.036 | 1.686 | 0.679 |
| 38 | 0.01 | 8 | 0.75 | **11.130** | 23.881 | 0.398 | 0.853 | 2.036 | 1.686 | 0.679 |
| 39 | 0.01 | 9 | 0.75 | **11.130** | 23.881 | 0.398 | 0.853 | 2.036 | 1.686 | 0.679 |
| 40 | 0.01 | 10 | 0.75 | **11.130** | 23.881 | 0.398 | 0.853 | 2.036 | 1.686 | 0.679 |
| 41 | 0.001 | 2 | 0.75 | **11.132** | 23.869 | 0.387 | 0.814 | 2.287 | 1.862 | 0.608 |
| 42 | 0.001 | 3 | 0.75 | **11.132** | 23.869 | 0.387 | 0.821 | 2.249 | 1.835 | 0.619 |
| 43 | 0.001 | 4 | 0.75 | **11.132** | 23.869 | 0.387 | 0.821 | 2.249 | 1.835 | 0.619 |
| 44 | 0.001 | 5 | 0.75 | **11.132** | 23.869 | 0.387 | 0.821 | 2.249 | 1.835 | 0.619 |
| 45 | 0.001 | 6 | 0.75 | **11.132** | 23.869 | 0.387 | 0.821 | 2.249 | 1.835 | 0.619 |
| 46 | 0.001 | 7 | 0.75 | **11.132** | 23.869 | 0.387 | 0.821 | 2.249 | 1.835 | 0.619 |
| 47 | 0.001 | 8 | 0.75 | **11.132** | 23.869 | 0.387 | 0.821 | 2.249 | 1.835 | 0.619 |
| 48 | 0.001 | 9 | 0.75 | **11.132** | 23.869 | 0.387 | 0.821 | 2.249 | 1.835 | 0.619 |
| 49 | 0.001 | 10 | 0.75 | **11.132** | 23.869 | 0.387 | 0.821 | 2.249 | 1.835 | 0.619 |
| 50 | 0.001 | 2 | 0.70 | **11.133** | 23.862 | 0.396 | 0.826 | 2.212 | 1.795 | 0.629 |
| 51 | 0.001 | 3 | 0.70 | **11.133** | 23.862 | 0.396 | 0.826 | 2.212 | 1.795 | 0.629 |
| 52 | 0.001 | 4 | 0.70 | **11.133** | 23.862 | 0.396 | 0.826 | 2.212 | 1.795 | 0.629 |
| 53 | 0.001 | 5 | 0.70 | **11.133** | 23.862 | 0.396 | 0.826 | 2.212 | 1.795 | 0.629 |
| 54 | 0.001 | 6 | 0.70 | **11.133** | 23.862 | 0.396 | 0.826 | 2.212 | 1.795 | 0.629 |
| 55 | 0.001 | 7 | 0.70 | **11.133** | 23.862 | 0.396 | 0.826 | 2.212 | 1.795 | 0.629 |
| 56 | 0.001 | 8 | 0.70 | **11.133** | 23.862 | 0.396 | 0.826 | 2.212 | 1.795 | 0.629 |
| 57 | 0.001 | 9 | 0.70 | **11.133** | 23.862 | 0.396 | 0.826 | 2.212 | 1.795 | 0.629 |
| 58 | 0.001 | 10 | 0.70 | **11.133** | 23.862 | 0.396 | 0.826 | 2.212 | 1.795 | 0.629 |
| 59 | 0.005 | 2 | 0.70 | **11.170** | 23.609 | 0.397 | 0.838 | 2.133 | 1.745 | 0.652 |
| 60 | 0.005 | 3 | 0.70 | **11.170** | 23.609 | 0.397 | 0.838 | 2.133 | 1.745 | 0.652 |
| 61 | 0.005 | 4 | 0.70 | **11.170** | 23.609 | 0.397 | 0.838 | 2.133 | 1.745 | 0.652 |
| 62 | 0.005 | 5 | 0.70 | **11.170** | 23.609 | 0.397 | 0.838 | 2.133 | 1.745 | 0.652 |
| 63 | 0.005 | 6 | 0.70 | **11.170** | 23.609 | 0.397 | 0.838 | 2.133 | 1.745 | 0.652 |
| 64 | 0.005 | 7 | 0.70 | **11.170** | 23.609 | 0.397 | 0.838 | 2.133 | 1.745 | 0.652 |
| 65 | 0.005 | 8 | 0.70 | **11.170** | 23.609 | 0.397 | 0.838 | 2.133 | 1.745 | 0.652 |
| 66 | 0.005 | 9 | 0.70 | **11.170** | 23.609 | 0.397 | 0.838 | 2.133 | 1.745 | 0.652 |
| 67 | 0.005 | 10 | 0.70 | **11.170** | 23.609 | 0.397 | 0.838 | 2.133 | 1.745 | 0.652 |
| 68 | 0.01 | 2 | 0.65 | **11.179** | 23.545 | 0.408 | 0.870 | 1.914 | 1.545 | 0.709 |
| 69 | 0.01 | 3 | 0.65 | **11.179** | 23.545 | 0.408 | 0.870 | 1.914 | 1.545 | 0.709 |
| 70 | 0.01 | 4 | 0.65 | **11.179** | 23.545 | 0.408 | 0.870 | 1.914 | 1.545 | 0.709 |
| 71 | 0.01 | 5 | 0.65 | **11.179** | 23.545 | 0.408 | 0.870 | 1.914 | 1.545 | 0.709 |
| 72 | 0.01 | 6 | 0.65 | **11.179** | 23.545 | 0.408 | 0.870 | 1.914 | 1.545 | 0.709 |
| 73 | 0.01 | 7 | 0.65 | **11.179** | 23.545 | 0.408 | 0.870 | 1.914 | 1.545 | 0.709 |
| 74 | 0.01 | 8 | 0.65 | **11.179** | 23.545 | 0.408 | 0.870 | 1.914 | 1.545 | 0.709 |
| 75 | 0.01 | 9 | 0.65 | **11.179** | 23.545 | 0.408 | 0.870 | 1.914 | 1.545 | 0.709 |
| 76 | 0.01 | 10 | 0.65 | **11.179** | 23.545 | 0.408 | 0.870 | 1.914 | 1.545 | 0.709 |
| 77 | 0.01 | 1 | 0.65 | **11.209** | 23.342 | 0.438 | 0.856 | 2.009 | 1.612 | 0.685 |
| 78 | 0.0005 | 2 | 0.75 | **11.212** | 23.319 | 0.379 | 0.795 | 2.398 | 1.943 | 0.575 |
| 79 | 0.0005 | 3 | 0.75 | **11.212** | 23.319 | 0.379 | 0.801 | 2.362 | 1.917 | 0.585 |
| 80 | 0.0005 | 4 | 0.75 | **11.212** | 23.319 | 0.379 | 0.801 | 2.362 | 1.917 | 0.585 |
| 81 | 0.0005 | 5 | 0.75 | **11.212** | 23.319 | 0.379 | 0.801 | 2.362 | 1.917 | 0.585 |
| 82 | 0.0005 | 6 | 0.75 | **11.212** | 23.319 | 0.379 | 0.801 | 2.362 | 1.917 | 0.585 |
| 83 | 0.0005 | 7 | 0.75 | **11.212** | 23.319 | 0.379 | 0.801 | 2.362 | 1.917 | 0.585 |
| 84 | 0.0005 | 8 | 0.75 | **11.212** | 23.319 | 0.379 | 0.801 | 2.362 | 1.917 | 0.585 |
| 85 | 0.0005 | 9 | 0.75 | **11.212** | 23.319 | 0.379 | 0.801 | 2.362 | 1.917 | 0.585 |
| 86 | 0.0005 | 10 | 0.75 | **11.212** | 23.319 | 0.379 | 0.801 | 2.362 | 1.917 | 0.585 |
| 87 | 0.0005 | 2 | 0.70 | **11.279** | 22.863 | 0.393 | 0.767 | 2.545 | 2.052 | 0.527 |
| 88 | 0.0005 | 3 | 0.70 | **11.279** | 22.863 | 0.393 | 0.767 | 2.545 | 2.052 | 0.527 |
| 89 | 0.0005 | 4 | 0.70 | **11.279** | 22.863 | 0.393 | 0.767 | 2.545 | 2.052 | 0.527 |
| 90 | 0.0005 | 5 | 0.70 | **11.279** | 22.863 | 0.393 | 0.767 | 2.545 | 2.052 | 0.527 |
| 91 | 0.0005 | 6 | 0.70 | **11.279** | 22.863 | 0.393 | 0.767 | 2.545 | 2.052 | 0.527 |
| 92 | 0.0005 | 7 | 0.70 | **11.279** | 22.863 | 0.393 | 0.767 | 2.545 | 2.052 | 0.527 |
| 93 | 0.0005 | 8 | 0.70 | **11.279** | 22.863 | 0.393 | 0.767 | 2.545 | 2.052 | 0.527 |
| 94 | 0.0005 | 9 | 0.70 | **11.279** | 22.863 | 0.393 | 0.767 | 2.545 | 2.052 | 0.527 |
| 95 | 0.0005 | 10 | 0.70 | **11.279** | 22.863 | 0.393 | 0.767 | 2.545 | 2.052 | 0.527 |
| 96 | 0.0001 | 2 | 0.75 | **11.350** | 22.380 | 0.384 | 0.748 | 2.673 | 2.171 | 0.484 |
| 97 | 0.0001 | 3 | 0.75 | **11.350** | 22.380 | 0.384 | 0.756 | 2.633 | 2.141 | 0.498 |
| 98 | 0.0001 | 4 | 0.75 | **11.350** | 22.380 | 0.384 | 0.756 | 2.633 | 2.141 | 0.498 |
| 99 | 0.0001 | 5 | 0.75 | **11.350** | 22.380 | 0.384 | 0.756 | 2.633 | 2.141 | 0.498 |
| 100 | 0.0001 | 6 | 0.75 | **11.350** | 22.380 | 0.384 | 0.756 | 2.633 | 2.141 | 0.498 |
| 101 | 0.0001 | 7 | 0.75 | **11.350** | 22.380 | 0.384 | 0.756 | 2.633 | 2.141 | 0.498 |
| 102 | 0.0001 | 8 | 0.75 | **11.350** | 22.380 | 0.384 | 0.756 | 2.633 | 2.141 | 0.498 |
| 103 | 0.0001 | 9 | 0.75 | **11.350** | 22.380 | 0.384 | 0.756 | 2.633 | 2.141 | 0.498 |
| 104 | 0.0001 | 10 | 0.75 | **11.350** | 22.380 | 0.384 | 0.756 | 2.633 | 2.141 | 0.498 |
| 105 | 0.0001 | 2 | 0.70 | **11.398** | 22.050 | 0.391 | 0.737 | 2.719 | 2.190 | 0.469 |
| 106 | 0.0001 | 3 | 0.70 | **11.398** | 22.050 | 0.391 | 0.737 | 2.719 | 2.190 | 0.469 |
| 107 | 0.0001 | 4 | 0.70 | **11.398** | 22.050 | 0.391 | 0.737 | 2.719 | 2.190 | 0.469 |
| 108 | 0.0001 | 5 | 0.70 | **11.398** | 22.050 | 0.391 | 0.737 | 2.719 | 2.190 | 0.469 |
| 109 | 0.0001 | 6 | 0.70 | **11.398** | 22.050 | 0.391 | 0.737 | 2.719 | 2.190 | 0.469 |
| 110 | 0.0001 | 7 | 0.70 | **11.398** | 22.050 | 0.391 | 0.737 | 2.719 | 2.190 | 0.469 |
| 111 | 0.0001 | 8 | 0.70 | **11.398** | 22.050 | 0.391 | 0.737 | 2.719 | 2.190 | 0.469 |
| 112 | 0.0001 | 9 | 0.70 | **11.398** | 22.050 | 0.391 | 0.737 | 2.719 | 2.190 | 0.469 |
| 113 | 0.0001 | 10 | 0.70 | **11.398** | 22.050 | 0.391 | 0.737 | 2.719 | 2.190 | 0.469 |
| 114 | 0.0005 | 2 | 0.65 | **11.406** | 21.996 | 0.402 | 0.756 | 2.594 | 2.081 | 0.511 |
| 115 | 0.0005 | 3 | 0.65 | **11.406** | 21.996 | 0.402 | 0.756 | 2.594 | 2.081 | 0.511 |
| 116 | 0.0005 | 4 | 0.65 | **11.406** | 21.996 | 0.402 | 0.756 | 2.594 | 2.081 | 0.511 |
| 117 | 0.0005 | 5 | 0.65 | **11.406** | 21.996 | 0.402 | 0.756 | 2.594 | 2.081 | 0.511 |
| 118 | 0.0005 | 6 | 0.65 | **11.406** | 21.996 | 0.402 | 0.756 | 2.594 | 2.081 | 0.511 |
| 119 | 0.0005 | 7 | 0.65 | **11.406** | 21.996 | 0.402 | 0.756 | 2.594 | 2.081 | 0.511 |
| 120 | 0.0005 | 8 | 0.65 | **11.406** | 21.996 | 0.402 | 0.756 | 2.594 | 2.081 | 0.511 |
| 121 | 0.0005 | 9 | 0.65 | **11.406** | 21.996 | 0.402 | 0.756 | 2.594 | 2.081 | 0.511 |
| 122 | 0.0005 | 10 | 0.65 | **11.406** | 21.996 | 0.402 | 0.756 | 2.594 | 2.081 | 0.511 |
| 123 | 0.001 | 1 | 0.75 | **11.412** | 21.953 | 0.401 | 0.735 | 2.667 | 2.156 | 0.486 |
| 124 | 0.001 | 2 | 0.65 | **11.413** | 21.946 | 0.404 | 0.768 | 2.531 | 2.027 | 0.532 |
| 125 | 0.001 | 3 | 0.65 | **11.413** | 21.946 | 0.404 | 0.768 | 2.531 | 2.027 | 0.532 |
| 126 | 0.001 | 4 | 0.65 | **11.413** | 21.946 | 0.404 | 0.768 | 2.531 | 2.027 | 0.532 |
| 127 | 0.001 | 5 | 0.65 | **11.413** | 21.946 | 0.404 | 0.768 | 2.531 | 2.027 | 0.532 |
| 128 | 0.001 | 6 | 0.65 | **11.413** | 21.946 | 0.404 | 0.768 | 2.531 | 2.027 | 0.532 |
| 129 | 0.001 | 7 | 0.65 | **11.413** | 21.946 | 0.404 | 0.768 | 2.531 | 2.027 | 0.532 |
| 130 | 0.001 | 8 | 0.65 | **11.413** | 21.946 | 0.404 | 0.768 | 2.531 | 2.027 | 0.532 |
| 131 | 0.001 | 9 | 0.65 | **11.413** | 21.946 | 0.404 | 0.768 | 2.531 | 2.027 | 0.532 |
| 132 | 0.001 | 10 | 0.65 | **11.413** | 21.946 | 0.404 | 0.768 | 2.531 | 2.027 | 0.532 |
| 133 | 0.005 | 2 | 0.65 | **11.455** | 21.662 | 0.407 | 0.746 | 2.646 | 2.120 | 0.493 |
| 134 | 0.005 | 3 | 0.65 | **11.455** | 21.662 | 0.407 | 0.746 | 2.646 | 2.120 | 0.493 |
| 135 | 0.005 | 4 | 0.65 | **11.455** | 21.662 | 0.407 | 0.746 | 2.646 | 2.120 | 0.493 |
| 136 | 0.005 | 5 | 0.65 | **11.455** | 21.662 | 0.407 | 0.746 | 2.646 | 2.120 | 0.493 |
| 137 | 0.005 | 6 | 0.65 | **11.455** | 21.662 | 0.407 | 0.746 | 2.646 | 2.120 | 0.493 |
| 138 | 0.005 | 7 | 0.65 | **11.455** | 21.662 | 0.407 | 0.746 | 2.646 | 2.120 | 0.493 |
| 139 | 0.005 | 8 | 0.65 | **11.455** | 21.662 | 0.407 | 0.746 | 2.646 | 2.120 | 0.493 |
| 140 | 0.005 | 9 | 0.65 | **11.455** | 21.662 | 0.407 | 0.746 | 2.646 | 2.120 | 0.493 |
| 141 | 0.005 | 10 | 0.65 | **11.455** | 21.662 | 0.407 | 0.746 | 2.646 | 2.120 | 0.493 |
| 142 | 0.0005 | 1 | 0.75 | **11.467** | 21.576 | 0.390 | 0.709 | 2.772 | 2.233 | 0.449 |
| 143 | 0.01 | 2 | 0.60 | **11.516** | 21.243 | 0.384 | 0.713 | 2.778 | 2.221 | 0.447 |
| 144 | 0.01 | 3 | 0.60 | **11.516** | 21.243 | 0.384 | 0.713 | 2.778 | 2.221 | 0.447 |
| 145 | 0.01 | 4 | 0.60 | **11.516** | 21.243 | 0.384 | 0.713 | 2.778 | 2.221 | 0.447 |
| 146 | 0.01 | 5 | 0.60 | **11.516** | 21.243 | 0.384 | 0.713 | 2.778 | 2.221 | 0.447 |
| 147 | 0.01 | 6 | 0.60 | **11.516** | 21.243 | 0.384 | 0.713 | 2.778 | 2.221 | 0.447 |
| 148 | 0.01 | 7 | 0.60 | **11.516** | 21.243 | 0.384 | 0.713 | 2.778 | 2.221 | 0.447 |
| 149 | 0.01 | 8 | 0.60 | **11.516** | 21.243 | 0.384 | 0.713 | 2.778 | 2.221 | 0.447 |
| 150 | 0.01 | 9 | 0.60 | **11.516** | 21.243 | 0.384 | 0.713 | 2.778 | 2.221 | 0.447 |
| 151 | 0.01 | 10 | 0.60 | **11.516** | 21.243 | 0.384 | 0.713 | 2.778 | 2.221 | 0.447 |
| 152 | 0.0001 | 2 | 0.65 | **11.516** | 21.245 | 0.382 | 0.720 | 2.791 | 2.235 | 0.442 |
| 153 | 0.0001 | 3 | 0.65 | **11.516** | 21.245 | 0.382 | 0.720 | 2.791 | 2.235 | 0.442 |
| 154 | 0.0001 | 4 | 0.65 | **11.516** | 21.245 | 0.382 | 0.720 | 2.791 | 2.235 | 0.442 |
| 155 | 0.0001 | 5 | 0.65 | **11.516** | 21.245 | 0.382 | 0.720 | 2.791 | 2.235 | 0.442 |
| 156 | 0.0001 | 6 | 0.65 | **11.516** | 21.245 | 0.382 | 0.720 | 2.791 | 2.235 | 0.442 |
| 157 | 0.0001 | 7 | 0.65 | **11.516** | 21.245 | 0.382 | 0.720 | 2.791 | 2.235 | 0.442 |
| 158 | 0.0001 | 8 | 0.65 | **11.516** | 21.245 | 0.382 | 0.720 | 2.791 | 2.235 | 0.442 |
| 159 | 0.0001 | 9 | 0.65 | **11.516** | 21.245 | 0.382 | 0.720 | 2.791 | 2.235 | 0.442 |
| 160 | 0.0001 | 10 | 0.65 | **11.516** | 21.245 | 0.382 | 0.720 | 2.791 | 2.235 | 0.442 |
| 161 | 0.001 | 1 | 0.70 | **11.555** | 20.974 | 0.406 | 0.722 | 2.713 | 2.175 | 0.470 |
| 162 | 0.0005 | 1 | 0.70 | **11.569** | 20.880 | 0.382 | 0.693 | 2.833 | 2.271 | 0.427 |
| 163 | 0.0001 | 1 | 0.75 | **11.626** | 20.492 | 0.382 | 0.670 | 2.949 | 2.373 | 0.383 |
| 164 | 0.0005 | 1 | 0.65 | **11.640** | 20.394 | 0.379 | 0.682 | 2.868 | 2.294 | 0.415 |
| 165 | 0.0005 | 2 | 0.60 | **11.656** | 20.287 | 0.369 | 0.719 | 2.752 | 2.195 | 0.456 |
| 166 | 0.0005 | 3 | 0.60 | **11.656** | 20.287 | 0.369 | 0.719 | 2.752 | 2.195 | 0.456 |
| 167 | 0.0005 | 4 | 0.60 | **11.656** | 20.287 | 0.369 | 0.719 | 2.752 | 2.195 | 0.456 |
| 168 | 0.0005 | 5 | 0.60 | **11.656** | 20.287 | 0.369 | 0.719 | 2.752 | 2.195 | 0.456 |
| 169 | 0.0005 | 6 | 0.60 | **11.656** | 20.287 | 0.369 | 0.719 | 2.752 | 2.195 | 0.456 |
| 170 | 0.0005 | 7 | 0.60 | **11.656** | 20.287 | 0.369 | 0.719 | 2.752 | 2.195 | 0.456 |
| 171 | 0.0005 | 8 | 0.60 | **11.656** | 20.287 | 0.369 | 0.719 | 2.752 | 2.195 | 0.456 |
| 172 | 0.0005 | 9 | 0.60 | **11.656** | 20.287 | 0.369 | 0.719 | 2.752 | 2.195 | 0.456 |
| 173 | 0.0005 | 10 | 0.60 | **11.656** | 20.287 | 0.369 | 0.719 | 2.752 | 2.195 | 0.456 |
| 174 | 0.005 | 2 | 0.60 | **11.658** | 20.268 | 0.381 | 0.711 | 2.784 | 2.213 | 0.445 |
| 175 | 0.005 | 3 | 0.60 | **11.658** | 20.268 | 0.381 | 0.711 | 2.784 | 2.213 | 0.445 |
| 176 | 0.005 | 4 | 0.60 | **11.658** | 20.268 | 0.381 | 0.711 | 2.784 | 2.213 | 0.445 |
| 177 | 0.005 | 5 | 0.60 | **11.658** | 20.268 | 0.381 | 0.711 | 2.784 | 2.213 | 0.445 |
| 178 | 0.005 | 6 | 0.60 | **11.658** | 20.268 | 0.381 | 0.711 | 2.784 | 2.213 | 0.445 |
| 179 | 0.005 | 7 | 0.60 | **11.658** | 20.268 | 0.381 | 0.711 | 2.784 | 2.213 | 0.445 |
| 180 | 0.005 | 8 | 0.60 | **11.658** | 20.268 | 0.381 | 0.711 | 2.784 | 2.213 | 0.445 |
| 181 | 0.005 | 9 | 0.60 | **11.658** | 20.268 | 0.381 | 0.711 | 2.784 | 2.213 | 0.445 |
| 182 | 0.005 | 10 | 0.60 | **11.658** | 20.268 | 0.381 | 0.711 | 2.784 | 2.213 | 0.445 |
| 183 | 0.01 | 1 | 0.55 | **11.663** | 20.236 | 0.381 | 0.682 | 2.852 | 2.277 | 0.420 |
| 184 | 0.01 | 2 | 0.55 | **11.663** | 20.236 | 0.381 | 0.702 | 2.786 | 2.229 | 0.444 |
| 185 | 0.01 | 3 | 0.55 | **11.663** | 20.236 | 0.381 | 0.702 | 2.786 | 2.229 | 0.444 |
| 186 | 0.01 | 4 | 0.55 | **11.663** | 20.236 | 0.381 | 0.702 | 2.786 | 2.229 | 0.444 |
| 187 | 0.01 | 5 | 0.55 | **11.663** | 20.236 | 0.381 | 0.702 | 2.786 | 2.229 | 0.444 |
| 188 | 0.01 | 6 | 0.55 | **11.663** | 20.236 | 0.381 | 0.702 | 2.786 | 2.229 | 0.444 |
| 189 | 0.01 | 7 | 0.55 | **11.663** | 20.236 | 0.381 | 0.702 | 2.786 | 2.229 | 0.444 |
| 190 | 0.01 | 8 | 0.55 | **11.663** | 20.236 | 0.381 | 0.702 | 2.786 | 2.229 | 0.444 |
| 191 | 0.01 | 9 | 0.55 | **11.663** | 20.236 | 0.381 | 0.702 | 2.786 | 2.229 | 0.444 |
| 192 | 0.01 | 10 | 0.55 | **11.663** | 20.236 | 0.381 | 0.702 | 2.786 | 2.229 | 0.444 |
| 193 | 0.001 | 2 | 0.60 | **11.680** | 20.119 | 0.368 | 0.721 | 2.739 | 2.191 | 0.461 |
| 194 | 0.001 | 3 | 0.60 | **11.680** | 20.119 | 0.368 | 0.721 | 2.739 | 2.191 | 0.461 |
| 195 | 0.001 | 4 | 0.60 | **11.680** | 20.119 | 0.368 | 0.721 | 2.739 | 2.191 | 0.461 |
| 196 | 0.001 | 5 | 0.60 | **11.680** | 20.119 | 0.368 | 0.721 | 2.739 | 2.191 | 0.461 |
| 197 | 0.001 | 6 | 0.60 | **11.680** | 20.119 | 0.368 | 0.721 | 2.739 | 2.191 | 0.461 |
| 198 | 0.001 | 7 | 0.60 | **11.680** | 20.119 | 0.368 | 0.721 | 2.739 | 2.191 | 0.461 |
| 199 | 0.001 | 8 | 0.60 | **11.680** | 20.119 | 0.368 | 0.721 | 2.739 | 2.191 | 0.461 |
| 200 | 0.001 | 9 | 0.60 | **11.680** | 20.119 | 0.368 | 0.721 | 2.739 | 2.191 | 0.461 |
| 201 | 0.001 | 10 | 0.60 | **11.680** | 20.119 | 0.368 | 0.721 | 2.739 | 2.191 | 0.461 |
| 202 | 0.001 | 1 | 0.65 | **11.681** | 20.111 | 0.380 | 0.683 | 2.865 | 2.289 | 0.416 |
| 203 | 0.0001 | 1 | 0.70 | **11.690** | 20.055 | 0.380 | 0.661 | 2.978 | 2.388 | 0.372 |
| 204 | 0.005 | 1 | 0.65 | **11.694** | 20.022 | 0.372 | 0.663 | 2.953 | 2.364 | 0.382 |
| 205 | 0.0005 | 1 | 0.60 | **11.733** | 19.757 | 0.366 | 0.664 | 2.934 | 2.341 | 0.389 |
| 206 | 0.0001 | 1 | 0.65 | **11.746** | 19.667 | 0.380 | 0.649 | 3.018 | 2.411 | 0.357 |
| 207 | 0.0001 | 2 | 0.60 | **11.749** | 19.650 | 0.352 | 0.691 | 2.896 | 2.301 | 0.403 |
| 208 | 0.0001 | 3 | 0.60 | **11.749** | 19.650 | 0.352 | 0.691 | 2.896 | 2.301 | 0.403 |
| 209 | 0.0001 | 4 | 0.60 | **11.749** | 19.650 | 0.352 | 0.691 | 2.896 | 2.301 | 0.403 |
| 210 | 0.0001 | 5 | 0.60 | **11.749** | 19.650 | 0.352 | 0.691 | 2.896 | 2.301 | 0.403 |
| 211 | 0.0001 | 6 | 0.60 | **11.749** | 19.650 | 0.352 | 0.691 | 2.896 | 2.301 | 0.403 |
| 212 | 0.0001 | 7 | 0.60 | **11.749** | 19.650 | 0.352 | 0.691 | 2.896 | 2.301 | 0.403 |
| 213 | 0.0001 | 8 | 0.60 | **11.749** | 19.650 | 0.352 | 0.691 | 2.896 | 2.301 | 0.403 |
| 214 | 0.0001 | 9 | 0.60 | **11.749** | 19.650 | 0.352 | 0.691 | 2.896 | 2.301 | 0.403 |
| 215 | 0.0001 | 10 | 0.60 | **11.749** | 19.650 | 0.352 | 0.691 | 2.896 | 2.301 | 0.403 |
| 216 | 0.005 | 1 | 0.60 | **11.753** | 19.619 | 0.368 | 0.652 | 2.981 | 2.373 | 0.371 |
| 217 | 0.001 | 1 | 0.60 | **11.760** | 19.572 | 0.365 | 0.669 | 2.912 | 2.327 | 0.398 |
| 218 | 0.005 | 1 | 0.55 | **11.762** | 19.561 | 0.370 | 0.670 | 2.886 | 2.316 | 0.408 |
| 219 | 0.005 | 2 | 0.55 | **11.762** | 19.561 | 0.370 | 0.689 | 2.826 | 2.270 | 0.430 |
| 220 | 0.005 | 3 | 0.55 | **11.762** | 19.561 | 0.370 | 0.689 | 2.826 | 2.270 | 0.430 |
| 221 | 0.005 | 4 | 0.55 | **11.762** | 19.561 | 0.370 | 0.689 | 2.826 | 2.270 | 0.430 |
| 222 | 0.005 | 5 | 0.55 | **11.762** | 19.561 | 0.370 | 0.689 | 2.826 | 2.270 | 0.430 |
| 223 | 0.005 | 6 | 0.55 | **11.762** | 19.561 | 0.370 | 0.689 | 2.826 | 2.270 | 0.430 |
| 224 | 0.005 | 7 | 0.55 | **11.762** | 19.561 | 0.370 | 0.689 | 2.826 | 2.270 | 0.430 |
| 225 | 0.005 | 8 | 0.55 | **11.762** | 19.561 | 0.370 | 0.689 | 2.826 | 2.270 | 0.430 |
| 226 | 0.005 | 9 | 0.55 | **11.762** | 19.561 | 0.370 | 0.689 | 2.826 | 2.270 | 0.430 |
| 227 | 0.005 | 10 | 0.55 | **11.762** | 19.561 | 0.370 | 0.689 | 2.826 | 2.270 | 0.430 |
| 228 | 0.0005 | 1 | 0.55 | **11.771** | 19.496 | 0.357 | 0.659 | 2.946 | 2.348 | 0.385 |
| 229 | 0.0005 | 2 | 0.55 | **11.771** | 19.496 | 0.357 | 0.676 | 2.894 | 2.305 | 0.404 |
| 230 | 0.0005 | 3 | 0.55 | **11.771** | 19.496 | 0.357 | 0.676 | 2.894 | 2.305 | 0.404 |
| 231 | 0.0005 | 4 | 0.55 | **11.771** | 19.496 | 0.357 | 0.676 | 2.894 | 2.305 | 0.404 |
| 232 | 0.0005 | 5 | 0.55 | **11.771** | 19.496 | 0.357 | 0.676 | 2.894 | 2.305 | 0.404 |
| 233 | 0.0005 | 6 | 0.55 | **11.771** | 19.496 | 0.357 | 0.676 | 2.894 | 2.305 | 0.404 |
| 234 | 0.0005 | 7 | 0.55 | **11.771** | 19.496 | 0.357 | 0.676 | 2.894 | 2.305 | 0.404 |
| 235 | 0.0005 | 8 | 0.55 | **11.771** | 19.496 | 0.357 | 0.676 | 2.894 | 2.305 | 0.404 |
| 236 | 0.0005 | 9 | 0.55 | **11.771** | 19.496 | 0.357 | 0.676 | 2.894 | 2.305 | 0.404 |
| 237 | 0.0005 | 10 | 0.55 | **11.771** | 19.496 | 0.357 | 0.676 | 2.894 | 2.305 | 0.404 |
| 238 | 0.001 | 1 | 0.55 | **11.779** | 19.441 | 0.354 | 0.661 | 2.938 | 2.338 | 0.388 |
| 239 | 0.001 | 2 | 0.55 | **11.779** | 19.441 | 0.354 | 0.678 | 2.886 | 2.295 | 0.407 |
| 240 | 0.001 | 3 | 0.55 | **11.779** | 19.441 | 0.354 | 0.678 | 2.886 | 2.295 | 0.407 |
| 241 | 0.001 | 4 | 0.55 | **11.779** | 19.441 | 0.354 | 0.678 | 2.886 | 2.295 | 0.407 |
| 242 | 0.001 | 5 | 0.55 | **11.779** | 19.441 | 0.354 | 0.678 | 2.886 | 2.295 | 0.407 |
| 243 | 0.001 | 6 | 0.55 | **11.779** | 19.441 | 0.354 | 0.678 | 2.886 | 2.295 | 0.407 |
| 244 | 0.001 | 7 | 0.55 | **11.779** | 19.441 | 0.354 | 0.678 | 2.886 | 2.295 | 0.407 |
| 245 | 0.001 | 8 | 0.55 | **11.779** | 19.441 | 0.354 | 0.678 | 2.886 | 2.295 | 0.407 |
| 246 | 0.001 | 9 | 0.55 | **11.779** | 19.441 | 0.354 | 0.678 | 2.886 | 2.295 | 0.407 |
| 247 | 0.001 | 10 | 0.55 | **11.779** | 19.441 | 0.354 | 0.678 | 2.886 | 2.295 | 0.407 |
| 248 | 0.01 | 1 | 0.50 | **11.794** | 19.339 | 0.359 | 0.665 | 2.895 | 2.312 | 0.404 |
| 249 | 0.01 | 2 | 0.50 | **11.794** | 19.339 | 0.359 | 0.665 | 2.895 | 2.312 | 0.404 |
| 250 | 0.01 | 3 | 0.50 | **11.794** | 19.339 | 0.359 | 0.665 | 2.895 | 2.312 | 0.404 |
| 251 | 0.01 | 4 | 0.50 | **11.794** | 19.339 | 0.359 | 0.665 | 2.895 | 2.312 | 0.404 |
| 252 | 0.01 | 5 | 0.50 | **11.794** | 19.339 | 0.359 | 0.665 | 2.895 | 2.312 | 0.404 |
| 253 | 0.01 | 6 | 0.50 | **11.794** | 19.339 | 0.359 | 0.665 | 2.895 | 2.312 | 0.404 |
| 254 | 0.01 | 7 | 0.50 | **11.794** | 19.339 | 0.359 | 0.665 | 2.895 | 2.312 | 0.404 |
| 255 | 0.01 | 8 | 0.50 | **11.794** | 19.339 | 0.359 | 0.665 | 2.895 | 2.312 | 0.404 |
| 256 | 0.01 | 9 | 0.50 | **11.794** | 19.339 | 0.359 | 0.665 | 2.895 | 2.312 | 0.404 |
| 257 | 0.01 | 10 | 0.50 | **11.794** | 19.339 | 0.359 | 0.665 | 2.895 | 2.312 | 0.404 |
| 258 | 0.0001 | 1 | 0.60 | **11.819** | 19.171 | 0.365 | 0.640 | 3.042 | 2.420 | 0.347 |
| 259 | 0.0001 | 1 | 0.55 | **11.896** | 18.645 | 0.355 | 0.631 | 3.067 | 2.432 | 0.338 |
| 260 | 0.0001 | 2 | 0.55 | **11.896** | 18.645 | 0.355 | 0.648 | 3.025 | 2.396 | 0.354 |
| 261 | 0.0001 | 3 | 0.55 | **11.896** | 18.645 | 0.355 | 0.648 | 3.025 | 2.396 | 0.354 |
| 262 | 0.0001 | 4 | 0.55 | **11.896** | 18.645 | 0.355 | 0.648 | 3.025 | 2.396 | 0.354 |
| 263 | 0.0001 | 5 | 0.55 | **11.896** | 18.645 | 0.355 | 0.648 | 3.025 | 2.396 | 0.354 |
| 264 | 0.0001 | 6 | 0.55 | **11.896** | 18.645 | 0.355 | 0.648 | 3.025 | 2.396 | 0.354 |
| 265 | 0.0001 | 7 | 0.55 | **11.896** | 18.645 | 0.355 | 0.648 | 3.025 | 2.396 | 0.354 |
| 266 | 0.0001 | 8 | 0.55 | **11.896** | 18.645 | 0.355 | 0.648 | 3.025 | 2.396 | 0.354 |
| 267 | 0.0001 | 9 | 0.55 | **11.896** | 18.645 | 0.355 | 0.648 | 3.025 | 2.396 | 0.354 |
| 268 | 0.0001 | 10 | 0.55 | **11.896** | 18.645 | 0.355 | 0.648 | 3.025 | 2.396 | 0.354 |
| 269 | 0.0005 | 1 | 0.50 | **11.897** | 18.636 | 0.346 | 0.658 | 2.941 | 2.344 | 0.386 |
| 270 | 0.0005 | 2 | 0.50 | **11.897** | 18.636 | 0.346 | 0.658 | 2.941 | 2.344 | 0.386 |
| 271 | 0.0005 | 3 | 0.50 | **11.897** | 18.636 | 0.346 | 0.658 | 2.941 | 2.344 | 0.386 |
| 272 | 0.0005 | 4 | 0.50 | **11.897** | 18.636 | 0.346 | 0.658 | 2.941 | 2.344 | 0.386 |
| 273 | 0.0005 | 5 | 0.50 | **11.897** | 18.636 | 0.346 | 0.658 | 2.941 | 2.344 | 0.386 |
| 274 | 0.0005 | 6 | 0.50 | **11.897** | 18.636 | 0.346 | 0.658 | 2.941 | 2.344 | 0.386 |
| 275 | 0.0005 | 7 | 0.50 | **11.897** | 18.636 | 0.346 | 0.658 | 2.941 | 2.344 | 0.386 |
| 276 | 0.0005 | 8 | 0.50 | **11.897** | 18.636 | 0.346 | 0.658 | 2.941 | 2.344 | 0.386 |
| 277 | 0.0005 | 9 | 0.50 | **11.897** | 18.636 | 0.346 | 0.658 | 2.941 | 2.344 | 0.386 |
| 278 | 0.0005 | 10 | 0.50 | **11.897** | 18.636 | 0.346 | 0.658 | 2.941 | 2.344 | 0.386 |
| 279 | 0.005 | 1 | 0.50 | **11.900** | 18.613 | 0.355 | 0.659 | 2.923 | 2.334 | 0.394 |
| 280 | 0.005 | 2 | 0.50 | **11.900** | 18.613 | 0.355 | 0.659 | 2.923 | 2.334 | 0.394 |
| 281 | 0.005 | 3 | 0.50 | **11.900** | 18.613 | 0.355 | 0.659 | 2.923 | 2.334 | 0.394 |
| 282 | 0.005 | 4 | 0.50 | **11.900** | 18.613 | 0.355 | 0.659 | 2.923 | 2.334 | 0.394 |
| 283 | 0.005 | 5 | 0.50 | **11.900** | 18.613 | 0.355 | 0.659 | 2.923 | 2.334 | 0.394 |
| 284 | 0.005 | 6 | 0.50 | **11.900** | 18.613 | 0.355 | 0.659 | 2.923 | 2.334 | 0.394 |
| 285 | 0.005 | 7 | 0.50 | **11.900** | 18.613 | 0.355 | 0.659 | 2.923 | 2.334 | 0.394 |
| 286 | 0.005 | 8 | 0.50 | **11.900** | 18.613 | 0.355 | 0.659 | 2.923 | 2.334 | 0.394 |
| 287 | 0.005 | 9 | 0.50 | **11.900** | 18.613 | 0.355 | 0.659 | 2.923 | 2.334 | 0.394 |
| 288 | 0.005 | 10 | 0.50 | **11.900** | 18.613 | 0.355 | 0.659 | 2.923 | 2.334 | 0.394 |
| 289 | 0.001 | 1 | 0.50 | **11.927** | 18.428 | 0.337 | 0.653 | 2.957 | 2.353 | 0.380 |
| 290 | 0.001 | 2 | 0.50 | **11.927** | 18.428 | 0.337 | 0.653 | 2.957 | 2.353 | 0.380 |
| 291 | 0.001 | 3 | 0.50 | **11.927** | 18.428 | 0.337 | 0.653 | 2.957 | 2.353 | 0.380 |
| 292 | 0.001 | 4 | 0.50 | **11.927** | 18.428 | 0.337 | 0.653 | 2.957 | 2.353 | 0.380 |
| 293 | 0.001 | 5 | 0.50 | **11.927** | 18.428 | 0.337 | 0.653 | 2.957 | 2.353 | 0.380 |
| 294 | 0.001 | 6 | 0.50 | **11.927** | 18.428 | 0.337 | 0.653 | 2.957 | 2.353 | 0.380 |
| 295 | 0.001 | 7 | 0.50 | **11.927** | 18.428 | 0.337 | 0.653 | 2.957 | 2.353 | 0.380 |
| 296 | 0.001 | 8 | 0.50 | **11.927** | 18.428 | 0.337 | 0.653 | 2.957 | 2.353 | 0.380 |
| 297 | 0.001 | 9 | 0.50 | **11.927** | 18.428 | 0.337 | 0.653 | 2.957 | 2.353 | 0.380 |
| 298 | 0.001 | 10 | 0.50 | **11.927** | 18.428 | 0.337 | 0.653 | 2.957 | 2.353 | 0.380 |
| 299 | 0.0001 | 1 | 0.50 | **12.008** | 17.878 | 0.346 | 0.626 | 3.080 | 2.442 | 0.332 |
| 300 | 0.0001 | 2 | 0.50 | **12.008** | 17.878 | 0.346 | 0.626 | 3.080 | 2.442 | 0.332 |
| 301 | 0.0001 | 3 | 0.50 | **12.008** | 17.878 | 0.346 | 0.626 | 3.080 | 2.442 | 0.332 |
| 302 | 0.0001 | 4 | 0.50 | **12.008** | 17.878 | 0.346 | 0.626 | 3.080 | 2.442 | 0.332 |
| 303 | 0.0001 | 5 | 0.50 | **12.008** | 17.878 | 0.346 | 0.626 | 3.080 | 2.442 | 0.332 |
| 304 | 0.0001 | 6 | 0.50 | **12.008** | 17.878 | 0.346 | 0.626 | 3.080 | 2.442 | 0.332 |
| 305 | 0.0001 | 7 | 0.50 | **12.008** | 17.878 | 0.346 | 0.626 | 3.080 | 2.442 | 0.332 |
| 306 | 0.0001 | 8 | 0.50 | **12.008** | 17.878 | 0.346 | 0.626 | 3.080 | 2.442 | 0.332 |
| 307 | 0.0001 | 9 | 0.50 | **12.008** | 17.878 | 0.346 | 0.626 | 3.080 | 2.442 | 0.332 |
| 308 | 0.0001 | 10 | 0.50 | **12.008** | 17.878 | 0.346 | 0.626 | 3.080 | 2.442 | 0.332 |
| 309 | 0.05 | 1 | 0.50 | **NA** | NA | NA | NA | NA | NA | NA |
| 310 | 0.05 | 2 | 0.50 | **NA** | NA | NA | NA | NA | NA | NA |
| 311 | 0.05 | 3 | 0.50 | **NA** | NA | NA | NA | NA | NA | NA |
| 312 | 0.05 | 4 | 0.50 | **NA** | NA | NA | NA | NA | NA | NA |
| 313 | 0.05 | 5 | 0.50 | **NA** | NA | NA | NA | NA | NA | NA |
| 314 | 0.05 | 6 | 0.50 | **NA** | NA | NA | NA | NA | NA | NA |
| 315 | 0.05 | 7 | 0.50 | **NA** | NA | NA | NA | NA | NA | NA |
| 316 | 0.05 | 8 | 0.50 | **NA** | NA | NA | NA | NA | NA | NA |
| 317 | 0.05 | 9 | 0.50 | **NA** | NA | NA | NA | NA | NA | NA |
| 318 | 0.05 | 10 | 0.50 | **NA** | NA | NA | NA | NA | NA | NA |
| 319 | 0.05 | 1 | 0.55 | **NA** | NA | NA | NA | NA | NA | NA |
| 320 | 0.05 | 2 | 0.55 | **NA** | NA | NA | NA | NA | NA | NA |
| 321 | 0.05 | 3 | 0.55 | **NA** | NA | NA | NA | NA | NA | NA |
| 322 | 0.05 | 4 | 0.55 | **NA** | NA | NA | NA | NA | NA | NA |
| 323 | 0.05 | 5 | 0.55 | **NA** | NA | NA | NA | NA | NA | NA |
| 324 | 0.05 | 6 | 0.55 | **NA** | NA | NA | NA | NA | NA | NA |
| 325 | 0.05 | 7 | 0.55 | **NA** | NA | NA | NA | NA | NA | NA |
| 326 | 0.05 | 8 | 0.55 | **NA** | NA | NA | NA | NA | NA | NA |
| 327 | 0.05 | 9 | 0.55 | **NA** | NA | NA | NA | NA | NA | NA |
| 328 | 0.05 | 10 | 0.55 | **NA** | NA | NA | NA | NA | NA | NA |
| 329 | 0.01 | 1 | 0.60 | **NA** | NA | NA | NA | NA | NA | NA |
| 330 | 0.05 | 1 | 0.60 | **NA** | NA | NA | NA | NA | NA | NA |
| 331 | 0.05 | 2 | 0.60 | **NA** | NA | NA | NA | NA | NA | NA |
| 332 | 0.05 | 3 | 0.60 | **NA** | NA | NA | NA | NA | NA | NA |
| 333 | 0.05 | 4 | 0.60 | **NA** | NA | NA | NA | NA | NA | NA |
| 334 | 0.05 | 5 | 0.60 | **NA** | NA | NA | NA | NA | NA | NA |
| 335 | 0.05 | 6 | 0.60 | **NA** | NA | NA | NA | NA | NA | NA |
| 336 | 0.05 | 7 | 0.60 | **NA** | NA | NA | NA | NA | NA | NA |
| 337 | 0.05 | 8 | 0.60 | **NA** | NA | NA | NA | NA | NA | NA |
| 338 | 0.05 | 9 | 0.60 | **NA** | NA | NA | NA | NA | NA | NA |
| 339 | 0.05 | 10 | 0.60 | **NA** | NA | NA | NA | NA | NA | NA |
| 340 | 0.05 | 1 | 0.65 | **NA** | NA | NA | NA | NA | NA | NA |
| 341 | 0.05 | 1 | 0.70 | **NA** | NA | NA | NA | NA | NA | NA |
| 342 | 0.05 | 2 | 0.70 | **NA** | NA | NA | NA | NA | NA | NA |
| 343 | 0.05 | 3 | 0.70 | **NA** | NA | NA | NA | NA | NA | NA |
| 344 | 0.05 | 4 | 0.70 | **NA** | NA | NA | NA | NA | NA | NA |
| 345 | 0.05 | 5 | 0.70 | **NA** | NA | NA | NA | NA | NA | NA |
| 346 | 0.05 | 6 | 0.70 | **NA** | NA | NA | NA | NA | NA | NA |
| 347 | 0.05 | 7 | 0.70 | **NA** | NA | NA | NA | NA | NA | NA |
| 348 | 0.05 | 8 | 0.70 | **NA** | NA | NA | NA | NA | NA | NA |
| 349 | 0.05 | 9 | 0.70 | **NA** | NA | NA | NA | NA | NA | NA |
| 350 | 0.05 | 10 | 0.70 | **NA** | NA | NA | NA | NA | NA | NA |
| 351 | 0.05 | 1 | 0.75 | **NA** | NA | NA | NA | NA | NA | NA |
| 352 | 0.05 | 2 | 0.75 | **NA** | NA | NA | NA | NA | NA | NA |
| 353 | 0.05 | 3 | 0.75 | **NA** | NA | NA | NA | NA | NA | NA |
| 354 | 0.05 | 4 | 0.75 | **NA** | NA | NA | NA | NA | NA | NA |
| 355 | 0.05 | 5 | 0.75 | **NA** | NA | NA | NA | NA | NA | NA |
| 356 | 0.05 | 6 | 0.75 | **NA** | NA | NA | NA | NA | NA | NA |
| 357 | 0.05 | 7 | 0.75 | **NA** | NA | NA | NA | NA | NA | NA |
| 358 | 0.05 | 8 | 0.75 | **NA** | NA | NA | NA | NA | NA | NA |
| 359 | 0.05 | 9 | 0.75 | **NA** | NA | NA | NA | NA | NA | NA |
| 360 | 0.05 | 10 | 0.75 | **NA** | NA | NA | NA | NA | NA | NA |

**Table S9.** Possible combinations of the three meta-parameters (learning rate, tree complexity, bag fraction) for fitted 360 BRT models for species richness at 25 m^2^ spatial scale based on 10-fold cross validation (CV) and the associated model performance parameters. Numbers are sorted according to increasing CV deviance (in bold). RMSE represents root mean square error, and MAE represents mean absolute error.

| No. | Learning rate | Tree complexity | Bag fraction | **CV deviance** | Percentage of explained deviance (%) | CV correlation | Training data correlation | RMSE  (%) | MAE  (%) | *R*^2^ |
| --- | --- | --- | --- | --- | --- | --- | --- | --- | --- | --- |
| 1 | 0.05 | 2 | 0.60 | **12.383** | 46.421 | 0.703 | 0.895 | 2.180 | 1.677 | 0.794 |
| 2 | 0.05 | 3 | 0.60 | **12.383** | 46.421 | 0.703 | 0.895 | 2.180 | 1.677 | 0.794 |
| 3 | 0.05 | 4 | 0.60 | **12.383** | 46.421 | 0.703 | 0.895 | 2.180 | 1.677 | 0.794 |
| 4 | 0.05 | 5 | 0.60 | **12.383** | 46.421 | 0.703 | 0.895 | 2.180 | 1.677 | 0.794 |
| 5 | 0.05 | 6 | 0.60 | **12.383** | 46.421 | 0.703 | 0.895 | 2.180 | 1.677 | 0.794 |
| 6 | 0.05 | 7 | 0.60 | **12.383** | 46.421 | 0.703 | 0.895 | 2.180 | 1.677 | 0.794 |
| 7 | 0.05 | 8 | 0.60 | **12.383** | 46.421 | 0.703 | 0.895 | 2.180 | 1.677 | 0.794 |
| 8 | 0.05 | 9 | 0.60 | **12.383** | 46.421 | 0.703 | 0.895 | 2.180 | 1.677 | 0.794 |
| 9 | 0.05 | 10 | 0.60 | **12.383** | 46.421 | 0.703 | 0.895 | 2.180 | 1.677 | 0.794 |
| 10 | 0.01 | 2 | 0.60 | **12.634** | 45.332 | 0.693 | 0.903 | 2.087 | 1.595 | 0.812 |
| 11 | 0.01 | 3 | 0.60 | **12.634** | 45.332 | 0.693 | 0.903 | 2.087 | 1.595 | 0.812 |
| 12 | 0.01 | 4 | 0.60 | **12.634** | 45.332 | 0.693 | 0.903 | 2.087 | 1.595 | 0.812 |
| 13 | 0.01 | 5 | 0.60 | **12.634** | 45.332 | 0.693 | 0.903 | 2.087 | 1.595 | 0.812 |
| 14 | 0.01 | 6 | 0.60 | **12.634** | 45.332 | 0.693 | 0.903 | 2.087 | 1.595 | 0.812 |
| 15 | 0.01 | 7 | 0.60 | **12.634** | 45.332 | 0.693 | 0.903 | 2.087 | 1.595 | 0.812 |
| 16 | 0.01 | 8 | 0.60 | **12.634** | 45.332 | 0.693 | 0.903 | 2.087 | 1.595 | 0.812 |
| 17 | 0.01 | 9 | 0.60 | **12.634** | 45.332 | 0.693 | 0.903 | 2.087 | 1.595 | 0.812 |
| 18 | 0.01 | 10 | 0.60 | **12.634** | 45.332 | 0.693 | 0.903 | 2.087 | 1.595 | 0.812 |
| 19 | 0.005 | 2 | 0.65 | **12.655** | 45.243 | 0.682 | 0.891 | 2.208 | 1.716 | 0.789 |
| 20 | 0.005 | 3 | 0.65 | **12.655** | 45.243 | 0.682 | 0.891 | 2.208 | 1.716 | 0.789 |
| 21 | 0.005 | 4 | 0.65 | **12.655** | 45.243 | 0.682 | 0.891 | 2.208 | 1.716 | 0.789 |
| 22 | 0.005 | 5 | 0.65 | **12.655** | 45.243 | 0.682 | 0.891 | 2.208 | 1.716 | 0.789 |
| 23 | 0.005 | 6 | 0.65 | **12.655** | 45.243 | 0.682 | 0.891 | 2.208 | 1.716 | 0.789 |
| 24 | 0.005 | 7 | 0.65 | **12.655** | 45.243 | 0.682 | 0.891 | 2.208 | 1.716 | 0.789 |
| 25 | 0.005 | 8 | 0.65 | **12.655** | 45.243 | 0.682 | 0.891 | 2.208 | 1.716 | 0.789 |
| 26 | 0.005 | 9 | 0.65 | **12.655** | 45.243 | 0.682 | 0.891 | 2.208 | 1.716 | 0.789 |
| 27 | 0.005 | 10 | 0.65 | **12.655** | 45.243 | 0.682 | 0.891 | 2.208 | 1.716 | 0.789 |
| 28 | 0.05 | 2 | 0.65 | **12.694** | 45.075 | 0.686 | 0.886 | 2.259 | 1.761 | 0.779 |
| 29 | 0.05 | 3 | 0.65 | **12.694** | 45.075 | 0.686 | 0.886 | 2.259 | 1.761 | 0.779 |
| 30 | 0.05 | 4 | 0.65 | **12.694** | 45.075 | 0.686 | 0.886 | 2.259 | 1.761 | 0.779 |
| 31 | 0.05 | 5 | 0.65 | **12.694** | 45.075 | 0.686 | 0.886 | 2.259 | 1.761 | 0.779 |
| 32 | 0.05 | 6 | 0.65 | **12.694** | 45.075 | 0.686 | 0.886 | 2.259 | 1.761 | 0.779 |
| 33 | 0.05 | 7 | 0.65 | **12.694** | 45.075 | 0.686 | 0.886 | 2.259 | 1.761 | 0.779 |
| 34 | 0.05 | 8 | 0.65 | **12.694** | 45.075 | 0.686 | 0.886 | 2.259 | 1.761 | 0.779 |
| 35 | 0.05 | 9 | 0.65 | **12.694** | 45.075 | 0.686 | 0.886 | 2.259 | 1.761 | 0.779 |
| 36 | 0.05 | 10 | 0.65 | **12.694** | 45.075 | 0.686 | 0.886 | 2.259 | 1.761 | 0.779 |
| 37 | 0.001 | 2 | 0.65 | **12.695** | 45.071 | 0.681 | 0.891 | 2.219 | 1.719 | 0.787 |
| 38 | 0.001 | 3 | 0.65 | **12.695** | 45.071 | 0.681 | 0.891 | 2.219 | 1.719 | 0.787 |
| 39 | 0.001 | 4 | 0.65 | **12.695** | 45.071 | 0.681 | 0.891 | 2.219 | 1.719 | 0.787 |
| 40 | 0.001 | 5 | 0.65 | **12.695** | 45.071 | 0.681 | 0.891 | 2.219 | 1.719 | 0.787 |
| 41 | 0.001 | 6 | 0.65 | **12.695** | 45.071 | 0.681 | 0.891 | 2.219 | 1.719 | 0.787 |
| 42 | 0.001 | 7 | 0.65 | **12.695** | 45.071 | 0.681 | 0.891 | 2.219 | 1.719 | 0.787 |
| 43 | 0.001 | 8 | 0.65 | **12.695** | 45.071 | 0.681 | 0.891 | 2.219 | 1.719 | 0.787 |
| 44 | 0.001 | 9 | 0.65 | **12.695** | 45.071 | 0.681 | 0.891 | 2.219 | 1.719 | 0.787 |
| 45 | 0.001 | 10 | 0.65 | **12.695** | 45.071 | 0.681 | 0.891 | 2.219 | 1.719 | 0.787 |
| 46 | 0.01 | 2 | 0.65 | **12.711** | 44.999 | 0.680 | 0.895 | 2.167 | 1.673 | 0.797 |
| 47 | 0.01 | 3 | 0.65 | **12.711** | 44.999 | 0.680 | 0.895 | 2.167 | 1.673 | 0.797 |
| 48 | 0.01 | 4 | 0.65 | **12.711** | 44.999 | 0.680 | 0.895 | 2.167 | 1.673 | 0.797 |
| 49 | 0.01 | 5 | 0.65 | **12.711** | 44.999 | 0.680 | 0.895 | 2.167 | 1.673 | 0.797 |
| 50 | 0.01 | 6 | 0.65 | **12.711** | 44.999 | 0.680 | 0.895 | 2.167 | 1.673 | 0.797 |
| 51 | 0.01 | 7 | 0.65 | **12.711** | 44.999 | 0.680 | 0.895 | 2.167 | 1.673 | 0.797 |
| 52 | 0.01 | 8 | 0.65 | **12.711** | 44.999 | 0.680 | 0.895 | 2.167 | 1.673 | 0.797 |
| 53 | 0.01 | 9 | 0.65 | **12.711** | 44.999 | 0.680 | 0.895 | 2.167 | 1.673 | 0.797 |
| 54 | 0.01 | 10 | 0.65 | **12.711** | 44.999 | 0.680 | 0.895 | 2.167 | 1.673 | 0.797 |
| 55 | 0.001 | 2 | 0.60 | **12.794** | 44.641 | 0.681 | 0.890 | 2.224 | 1.716 | 0.786 |
| 56 | 0.001 | 3 | 0.60 | **12.794** | 44.641 | 0.681 | 0.890 | 2.224 | 1.716 | 0.786 |
| 57 | 0.001 | 4 | 0.60 | **12.794** | 44.641 | 0.681 | 0.890 | 2.224 | 1.716 | 0.786 |
| 58 | 0.001 | 5 | 0.60 | **12.794** | 44.641 | 0.681 | 0.890 | 2.224 | 1.716 | 0.786 |
| 59 | 0.001 | 6 | 0.60 | **12.794** | 44.641 | 0.681 | 0.890 | 2.224 | 1.716 | 0.786 |
| 60 | 0.001 | 7 | 0.60 | **12.794** | 44.641 | 0.681 | 0.890 | 2.224 | 1.716 | 0.786 |
| 61 | 0.001 | 8 | 0.60 | **12.794** | 44.641 | 0.681 | 0.890 | 2.224 | 1.716 | 0.786 |
| 62 | 0.001 | 9 | 0.60 | **12.794** | 44.641 | 0.681 | 0.890 | 2.224 | 1.716 | 0.786 |
| 63 | 0.001 | 10 | 0.60 | **12.794** | 44.641 | 0.681 | 0.890 | 2.224 | 1.716 | 0.786 |
| 64 | 0.005 | 1 | 0.65 | **12.806** | 44.588 | 0.680 | 0.873 | 2.392 | 1.853 | 0.752 |
| 65 | 0.0005 | 2 | 0.65 | **12.814** | 44.553 | 0.673 | 0.882 | 2.308 | 1.786 | 0.770 |
| 66 | 0.0005 | 3 | 0.65 | **12.814** | 44.553 | 0.673 | 0.882 | 2.308 | 1.786 | 0.770 |
| 67 | 0.0005 | 4 | 0.65 | **12.814** | 44.553 | 0.673 | 0.882 | 2.308 | 1.786 | 0.770 |
| 68 | 0.0005 | 5 | 0.65 | **12.814** | 44.553 | 0.673 | 0.882 | 2.308 | 1.786 | 0.770 |
| 69 | 0.0005 | 6 | 0.65 | **12.814** | 44.553 | 0.673 | 0.882 | 2.308 | 1.786 | 0.770 |
| 70 | 0.0005 | 7 | 0.65 | **12.814** | 44.553 | 0.673 | 0.882 | 2.308 | 1.786 | 0.770 |
| 71 | 0.0005 | 8 | 0.65 | **12.814** | 44.553 | 0.673 | 0.882 | 2.308 | 1.786 | 0.770 |
| 72 | 0.0005 | 9 | 0.65 | **12.814** | 44.553 | 0.673 | 0.882 | 2.308 | 1.786 | 0.770 |
| 73 | 0.0005 | 10 | 0.65 | **12.814** | 44.553 | 0.673 | 0.882 | 2.308 | 1.786 | 0.770 |
| 74 | 0.01 | 1 | 0.60 | **12.815** | 44.551 | 0.683 | 0.879 | 2.334 | 1.809 | 0.764 |
| 75 | 0.01 | 1 | 0.65 | **12.815** | 44.550 | 0.683 | 0.885 | 2.282 | 1.755 | 0.775 |
| 76 | 0.005 | 2 | 0.60 | **12.851** | 44.395 | 0.675 | 0.884 | 2.285 | 1.765 | 0.774 |
| 77 | 0.005 | 3 | 0.60 | **12.851** | 44.395 | 0.675 | 0.884 | 2.285 | 1.765 | 0.774 |
| 78 | 0.005 | 4 | 0.60 | **12.851** | 44.395 | 0.675 | 0.884 | 2.285 | 1.765 | 0.774 |
| 79 | 0.005 | 5 | 0.60 | **12.851** | 44.395 | 0.675 | 0.884 | 2.285 | 1.765 | 0.774 |
| 80 | 0.005 | 6 | 0.60 | **12.851** | 44.395 | 0.675 | 0.884 | 2.285 | 1.765 | 0.774 |
| 81 | 0.005 | 7 | 0.60 | **12.851** | 44.395 | 0.675 | 0.884 | 2.285 | 1.765 | 0.774 |
| 82 | 0.005 | 8 | 0.60 | **12.851** | 44.395 | 0.675 | 0.884 | 2.285 | 1.765 | 0.774 |
| 83 | 0.005 | 9 | 0.60 | **12.851** | 44.395 | 0.675 | 0.884 | 2.285 | 1.765 | 0.774 |
| 84 | 0.005 | 10 | 0.60 | **12.851** | 44.395 | 0.675 | 0.884 | 2.285 | 1.765 | 0.774 |
| 85 | 0.05 | 1 | 0.65 | **12.860** | 44.355 | 0.685 | 0.877 | 2.369 | 1.839 | 0.757 |
| 86 | 0.005 | 1 | 0.60 | **12.866** | 44.331 | 0.688 | 0.882 | 2.306 | 1.788 | 0.770 |
| 87 | 0.005 | 2 | 0.70 | **12.870** | 44.313 | 0.671 | 0.897 | 2.152 | 1.660 | 0.800 |
| 88 | 0.005 | 3 | 0.70 | **12.870** | 44.313 | 0.671 | 0.897 | 2.152 | 1.660 | 0.800 |
| 89 | 0.005 | 4 | 0.70 | **12.870** | 44.313 | 0.671 | 0.897 | 2.152 | 1.660 | 0.800 |
| 90 | 0.005 | 5 | 0.70 | **12.870** | 44.313 | 0.671 | 0.897 | 2.152 | 1.660 | 0.800 |
| 91 | 0.005 | 6 | 0.70 | **12.870** | 44.313 | 0.671 | 0.897 | 2.152 | 1.660 | 0.800 |
| 92 | 0.005 | 7 | 0.70 | **12.870** | 44.313 | 0.671 | 0.897 | 2.152 | 1.660 | 0.800 |
| 93 | 0.005 | 8 | 0.70 | **12.870** | 44.313 | 0.671 | 0.897 | 2.152 | 1.660 | 0.800 |
| 94 | 0.005 | 9 | 0.70 | **12.870** | 44.313 | 0.671 | 0.897 | 2.152 | 1.660 | 0.800 |
| 95 | 0.005 | 10 | 0.70 | **12.870** | 44.313 | 0.671 | 0.897 | 2.152 | 1.660 | 0.800 |
| 96 | 0.001 | 2 | 0.70 | **12.894** | 44.207 | 0.668 | 0.890 | 2.219 | 1.720 | 0.787 |
| 97 | 0.001 | 3 | 0.70 | **12.894** | 44.207 | 0.668 | 0.890 | 2.219 | 1.720 | 0.787 |
| 98 | 0.001 | 4 | 0.70 | **12.894** | 44.207 | 0.668 | 0.890 | 2.219 | 1.720 | 0.787 |
| 99 | 0.001 | 5 | 0.70 | **12.894** | 44.207 | 0.668 | 0.890 | 2.219 | 1.720 | 0.787 |
| 100 | 0.001 | 6 | 0.70 | **12.894** | 44.207 | 0.668 | 0.890 | 2.219 | 1.720 | 0.787 |
| 101 | 0.001 | 7 | 0.70 | **12.894** | 44.207 | 0.668 | 0.890 | 2.219 | 1.720 | 0.787 |
| 102 | 0.001 | 8 | 0.70 | **12.894** | 44.207 | 0.668 | 0.890 | 2.219 | 1.720 | 0.787 |
| 103 | 0.001 | 9 | 0.70 | **12.894** | 44.207 | 0.668 | 0.890 | 2.219 | 1.720 | 0.787 |
| 104 | 0.001 | 10 | 0.70 | **12.894** | 44.207 | 0.668 | 0.890 | 2.219 | 1.720 | 0.787 |
| 105 | 0.01 | 1 | 0.70 | **12.955** | 43.945 | 0.682 | 0.889 | 2.230 | 1.739 | 0.785 |
| 106 | 0.005 | 1 | 0.70 | **12.977** | 43.848 | 0.676 | 0.881 | 2.323 | 1.804 | 0.767 |
| 107 | 0.01 | 2 | 0.70 | **12.978** | 43.843 | 0.667 | 0.892 | 2.198 | 1.714 | 0.791 |
| 108 | 0.01 | 3 | 0.70 | **12.978** | 43.843 | 0.667 | 0.892 | 2.198 | 1.714 | 0.791 |
| 109 | 0.01 | 4 | 0.70 | **12.978** | 43.843 | 0.667 | 0.892 | 2.198 | 1.714 | 0.791 |
| 110 | 0.01 | 5 | 0.70 | **12.978** | 43.843 | 0.667 | 0.892 | 2.198 | 1.714 | 0.791 |
| 111 | 0.01 | 6 | 0.70 | **12.978** | 43.843 | 0.667 | 0.892 | 2.198 | 1.714 | 0.791 |
| 112 | 0.01 | 7 | 0.70 | **12.978** | 43.843 | 0.667 | 0.892 | 2.198 | 1.714 | 0.791 |
| 113 | 0.01 | 8 | 0.70 | **12.978** | 43.843 | 0.667 | 0.892 | 2.198 | 1.714 | 0.791 |
| 114 | 0.01 | 9 | 0.70 | **12.978** | 43.843 | 0.667 | 0.892 | 2.198 | 1.714 | 0.791 |
| 115 | 0.01 | 10 | 0.70 | **12.978** | 43.843 | 0.667 | 0.892 | 2.198 | 1.714 | 0.791 |
| 116 | 0.05 | 1 | 0.70 | **12.987** | 43.806 | 0.684 | 0.875 | 2.379 | 1.827 | 0.755 |
| 117 | 0.0005 | 2 | 0.70 | **13.045** | 43.557 | 0.658 | 0.878 | 2.350 | 1.829 | 0.761 |
| 118 | 0.0005 | 3 | 0.70 | **13.045** | 43.557 | 0.658 | 0.878 | 2.350 | 1.829 | 0.761 |
| 119 | 0.0005 | 4 | 0.70 | **13.045** | 43.557 | 0.658 | 0.878 | 2.350 | 1.829 | 0.761 |
| 120 | 0.0005 | 5 | 0.70 | **13.045** | 43.557 | 0.658 | 0.878 | 2.350 | 1.829 | 0.761 |
| 121 | 0.0005 | 6 | 0.70 | **13.045** | 43.557 | 0.658 | 0.878 | 2.350 | 1.829 | 0.761 |
| 122 | 0.0005 | 7 | 0.70 | **13.045** | 43.557 | 0.658 | 0.878 | 2.350 | 1.829 | 0.761 |
| 123 | 0.0005 | 8 | 0.70 | **13.045** | 43.557 | 0.658 | 0.878 | 2.350 | 1.829 | 0.761 |
| 124 | 0.0005 | 9 | 0.70 | **13.045** | 43.557 | 0.658 | 0.878 | 2.350 | 1.829 | 0.761 |
| 125 | 0.0005 | 10 | 0.70 | **13.045** | 43.557 | 0.658 | 0.878 | 2.350 | 1.829 | 0.761 |
| 126 | 0.001 | 1 | 0.65 | **13.088** | 43.369 | 0.667 | 0.863 | 2.483 | 1.916 | 0.733 |
| 127 | 0.001 | 1 | 0.60 | **13.150** | 43.099 | 0.667 | 0.860 | 2.511 | 1.929 | 0.727 |
| 128 | 0.005 | 2 | 0.75 | **13.159** | 43.063 | 0.653 | 0.888 | 2.238 | 1.741 | 0.783 |
| 129 | 0.005 | 3 | 0.75 | **13.159** | 43.063 | 0.653 | 0.891 | 2.206 | 1.716 | 0.789 |
| 130 | 0.005 | 4 | 0.75 | **13.159** | 43.063 | 0.653 | 0.891 | 2.206 | 1.716 | 0.789 |
| 131 | 0.005 | 5 | 0.75 | **13.159** | 43.063 | 0.653 | 0.891 | 2.206 | 1.716 | 0.789 |
| 132 | 0.005 | 6 | 0.75 | **13.159** | 43.063 | 0.653 | 0.891 | 2.206 | 1.716 | 0.789 |
| 133 | 0.005 | 7 | 0.75 | **13.159** | 43.063 | 0.653 | 0.891 | 2.206 | 1.716 | 0.789 |
| 134 | 0.005 | 8 | 0.75 | **13.159** | 43.063 | 0.653 | 0.891 | 2.206 | 1.716 | 0.789 |
| 135 | 0.005 | 9 | 0.75 | **13.159** | 43.063 | 0.653 | 0.891 | 2.206 | 1.716 | 0.789 |
| 136 | 0.005 | 10 | 0.75 | **13.159** | 43.063 | 0.653 | 0.891 | 2.206 | 1.716 | 0.789 |
| 137 | 0.05 | 2 | 0.70 | **13.167** | 43.026 | 0.666 | 0.890 | 2.213 | 1.730 | 0.788 |
| 138 | 0.05 | 3 | 0.70 | **13.167** | 43.026 | 0.666 | 0.890 | 2.213 | 1.730 | 0.788 |
| 139 | 0.05 | 4 | 0.70 | **13.167** | 43.026 | 0.666 | 0.890 | 2.213 | 1.730 | 0.788 |
| 140 | 0.05 | 5 | 0.70 | **13.167** | 43.026 | 0.666 | 0.890 | 2.213 | 1.730 | 0.788 |
| 141 | 0.05 | 6 | 0.70 | **13.167** | 43.026 | 0.666 | 0.890 | 2.213 | 1.730 | 0.788 |
| 142 | 0.05 | 7 | 0.70 | **13.167** | 43.026 | 0.666 | 0.890 | 2.213 | 1.730 | 0.788 |
| 143 | 0.05 | 8 | 0.70 | **13.167** | 43.026 | 0.666 | 0.890 | 2.213 | 1.730 | 0.788 |
| 144 | 0.05 | 9 | 0.70 | **13.167** | 43.026 | 0.666 | 0.890 | 2.213 | 1.730 | 0.788 |
| 145 | 0.05 | 10 | 0.70 | **13.167** | 43.026 | 0.666 | 0.890 | 2.213 | 1.730 | 0.788 |
| 146 | 0.05 | 1 | 0.70 | **13.183** | 42.957 | 0.672 | 0.879 | 2.340 | 1.828 | 0.763 |
| 147 | 0.001 | 1 | 0.70 | **13.217** | 42.809 | 0.658 | 0.864 | 2.477 | 1.921 | 0.735 |
| 148 | 0.0005 | 2 | 0.60 | **13.234** | 42.739 | 0.661 | 0.872 | 2.402 | 1.848 | 0.750 |
| 149 | 0.0005 | 3 | 0.60 | **13.234** | 42.739 | 0.661 | 0.872 | 2.402 | 1.848 | 0.750 |
| 150 | 0.0005 | 4 | 0.60 | **13.234** | 42.739 | 0.661 | 0.872 | 2.402 | 1.848 | 0.750 |
| 151 | 0.0005 | 5 | 0.60 | **13.234** | 42.739 | 0.661 | 0.872 | 2.402 | 1.848 | 0.750 |
| 152 | 0.0005 | 6 | 0.60 | **13.234** | 42.739 | 0.661 | 0.872 | 2.402 | 1.848 | 0.750 |
| 153 | 0.0005 | 7 | 0.60 | **13.234** | 42.739 | 0.661 | 0.872 | 2.402 | 1.848 | 0.750 |
| 154 | 0.0005 | 8 | 0.60 | **13.234** | 42.739 | 0.661 | 0.872 | 2.402 | 1.848 | 0.750 |
| 155 | 0.0005 | 9 | 0.60 | **13.234** | 42.739 | 0.661 | 0.872 | 2.402 | 1.848 | 0.750 |
| 156 | 0.0005 | 10 | 0.60 | **13.234** | 42.739 | 0.661 | 0.872 | 2.402 | 1.848 | 0.750 |
| 157 | 0.01 | 1 | 0.75 | **13.241** | 42.709 | 0.676 | 0.890 | 2.232 | 1.740 | 0.784 |
| 158 | 0.005 | 1 | 0.75 | **13.244** | 42.696 | 0.674 | 0.890 | 2.230 | 1.739 | 0.785 |
| 159 | 0.001 | 2 | 0.75 | **13.257** | 42.636 | 0.651 | 0.884 | 2.282 | 1.782 | 0.775 |
| 160 | 0.001 | 3 | 0.75 | **13.257** | 42.636 | 0.651 | 0.888 | 2.248 | 1.758 | 0.781 |
| 161 | 0.001 | 4 | 0.75 | **13.257** | 42.636 | 0.651 | 0.888 | 2.248 | 1.758 | 0.781 |
| 162 | 0.001 | 5 | 0.75 | **13.257** | 42.636 | 0.651 | 0.888 | 2.248 | 1.758 | 0.781 |
| 163 | 0.001 | 6 | 0.75 | **13.257** | 42.636 | 0.651 | 0.888 | 2.248 | 1.758 | 0.781 |
| 164 | 0.001 | 7 | 0.75 | **13.257** | 42.636 | 0.651 | 0.888 | 2.248 | 1.758 | 0.781 |
| 165 | 0.001 | 8 | 0.75 | **13.257** | 42.636 | 0.651 | 0.888 | 2.248 | 1.758 | 0.781 |
| 166 | 0.001 | 9 | 0.75 | **13.257** | 42.636 | 0.651 | 0.888 | 2.248 | 1.758 | 0.781 |
| 167 | 0.001 | 10 | 0.75 | **13.257** | 42.636 | 0.651 | 0.888 | 2.248 | 1.758 | 0.781 |
| 168 | 0.01 | 2 | 0.75 | **13.314** | 42.390 | 0.650 | 0.884 | 2.285 | 1.781 | 0.774 |
| 169 | 0.01 | 3 | 0.75 | **13.314** | 42.390 | 0.650 | 0.887 | 2.251 | 1.757 | 0.781 |
| 170 | 0.01 | 4 | 0.75 | **13.314** | 42.390 | 0.650 | 0.887 | 2.251 | 1.757 | 0.781 |
| 171 | 0.01 | 5 | 0.75 | **13.314** | 42.390 | 0.650 | 0.887 | 2.251 | 1.757 | 0.781 |
| 172 | 0.01 | 6 | 0.75 | **13.314** | 42.390 | 0.650 | 0.887 | 2.251 | 1.757 | 0.781 |
| 173 | 0.01 | 7 | 0.75 | **13.314** | 42.390 | 0.650 | 0.887 | 2.251 | 1.757 | 0.781 |
| 174 | 0.01 | 8 | 0.75 | **13.314** | 42.390 | 0.650 | 0.887 | 2.251 | 1.757 | 0.781 |
| 175 | 0.01 | 9 | 0.75 | **13.314** | 42.390 | 0.650 | 0.887 | 2.251 | 1.757 | 0.781 |
| 176 | 0.01 | 10 | 0.75 | **13.314** | 42.390 | 0.650 | 0.887 | 2.251 | 1.757 | 0.781 |
| 177 | 0.0005 | 2 | 0.75 | **13.322** | 42.355 | 0.645 | 0.877 | 2.354 | 1.839 | 0.760 |
| 178 | 0.0005 | 3 | 0.75 | **13.322** | 42.355 | 0.645 | 0.881 | 2.314 | 1.812 | 0.768 |
| 179 | 0.0005 | 4 | 0.75 | **13.322** | 42.355 | 0.645 | 0.881 | 2.314 | 1.812 | 0.768 |
| 180 | 0.0005 | 5 | 0.75 | **13.322** | 42.355 | 0.645 | 0.881 | 2.314 | 1.812 | 0.768 |
| 181 | 0.0005 | 6 | 0.75 | **13.322** | 42.355 | 0.645 | 0.881 | 2.314 | 1.812 | 0.768 |
| 182 | 0.0005 | 7 | 0.75 | **13.322** | 42.355 | 0.645 | 0.881 | 2.314 | 1.812 | 0.768 |
| 183 | 0.0005 | 8 | 0.75 | **13.322** | 42.355 | 0.645 | 0.881 | 2.314 | 1.812 | 0.768 |
| 184 | 0.0005 | 9 | 0.75 | **13.322** | 42.355 | 0.645 | 0.881 | 2.314 | 1.812 | 0.768 |
| 185 | 0.0005 | 10 | 0.75 | **13.322** | 42.355 | 0.645 | 0.881 | 2.314 | 1.812 | 0.768 |
| 186 | 0.01 | 1 | 0.55 | **13.343** | 42.268 | 0.675 | 0.876 | 2.355 | 1.829 | 0.760 |
| 187 | 0.01 | 2 | 0.55 | **13.343** | 42.268 | 0.675 | 0.890 | 2.228 | 1.735 | 0.785 |
| 188 | 0.01 | 3 | 0.55 | **13.343** | 42.268 | 0.675 | 0.890 | 2.228 | 1.735 | 0.785 |
| 189 | 0.01 | 4 | 0.55 | **13.343** | 42.268 | 0.675 | 0.890 | 2.228 | 1.735 | 0.785 |
| 190 | 0.01 | 5 | 0.55 | **13.343** | 42.268 | 0.675 | 0.890 | 2.228 | 1.735 | 0.785 |
| 191 | 0.01 | 6 | 0.55 | **13.343** | 42.268 | 0.675 | 0.890 | 2.228 | 1.735 | 0.785 |
| 192 | 0.01 | 7 | 0.55 | **13.343** | 42.268 | 0.675 | 0.890 | 2.228 | 1.735 | 0.785 |
| 193 | 0.01 | 8 | 0.55 | **13.343** | 42.268 | 0.675 | 0.890 | 2.228 | 1.735 | 0.785 |
| 194 | 0.01 | 9 | 0.55 | **13.343** | 42.268 | 0.675 | 0.890 | 2.228 | 1.735 | 0.785 |
| 195 | 0.01 | 10 | 0.55 | **13.343** | 42.268 | 0.675 | 0.890 | 2.228 | 1.735 | 0.785 |
| 196 | 0.05 | 1 | 0.55 | **13.353** | 42.223 | 0.671 | 0.874 | 2.404 | 1.829 | 0.750 |
| 197 | 0.05 | 2 | 0.55 | **13.353** | 42.223 | 0.671 | 0.884 | 2.304 | 1.768 | 0.770 |
| 198 | 0.05 | 3 | 0.55 | **13.353** | 42.223 | 0.671 | 0.884 | 2.304 | 1.768 | 0.770 |
| 199 | 0.05 | 4 | 0.55 | **13.353** | 42.223 | 0.671 | 0.884 | 2.304 | 1.768 | 0.770 |
| 200 | 0.05 | 5 | 0.55 | **13.353** | 42.223 | 0.671 | 0.884 | 2.304 | 1.768 | 0.770 |
| 201 | 0.05 | 6 | 0.55 | **13.353** | 42.223 | 0.671 | 0.884 | 2.304 | 1.768 | 0.770 |
| 202 | 0.05 | 7 | 0.55 | **13.353** | 42.223 | 0.671 | 0.884 | 2.304 | 1.768 | 0.770 |
| 203 | 0.05 | 8 | 0.55 | **13.353** | 42.223 | 0.671 | 0.884 | 2.304 | 1.768 | 0.770 |
| 204 | 0.05 | 9 | 0.55 | **13.353** | 42.223 | 0.671 | 0.884 | 2.304 | 1.768 | 0.770 |
| 205 | 0.05 | 10 | 0.55 | **13.353** | 42.223 | 0.671 | 0.884 | 2.304 | 1.768 | 0.770 |
| 206 | 0.005 | 1 | 0.55 | **13.365** | 42.169 | 0.674 | 0.876 | 2.358 | 1.823 | 0.760 |
| 207 | 0.005 | 2 | 0.55 | **13.365** | 42.169 | 0.674 | 0.889 | 2.231 | 1.740 | 0.785 |
| 208 | 0.005 | 3 | 0.55 | **13.365** | 42.169 | 0.674 | 0.889 | 2.231 | 1.740 | 0.785 |
| 209 | 0.005 | 4 | 0.55 | **13.365** | 42.169 | 0.674 | 0.889 | 2.231 | 1.740 | 0.785 |
| 210 | 0.005 | 5 | 0.55 | **13.365** | 42.169 | 0.674 | 0.889 | 2.231 | 1.740 | 0.785 |
| 211 | 0.005 | 6 | 0.55 | **13.365** | 42.169 | 0.674 | 0.889 | 2.231 | 1.740 | 0.785 |
| 212 | 0.005 | 7 | 0.55 | **13.365** | 42.169 | 0.674 | 0.889 | 2.231 | 1.740 | 0.785 |
| 213 | 0.005 | 8 | 0.55 | **13.365** | 42.169 | 0.674 | 0.889 | 2.231 | 1.740 | 0.785 |
| 214 | 0.005 | 9 | 0.55 | **13.365** | 42.169 | 0.674 | 0.889 | 2.231 | 1.740 | 0.785 |
| 215 | 0.005 | 10 | 0.55 | **13.365** | 42.169 | 0.674 | 0.889 | 2.231 | 1.740 | 0.785 |
| 216 | 0.05 | 2 | 0.75 | **13.383** | 42.091 | 0.663 | 0.894 | 2.176 | 1.683 | 0.795 |
| 217 | 0.05 | 3 | 0.75 | **13.383** | 42.091 | 0.663 | 0.897 | 2.149 | 1.666 | 0.800 |
| 218 | 0.05 | 4 | 0.75 | **13.383** | 42.091 | 0.663 | 0.897 | 2.149 | 1.666 | 0.800 |
| 219 | 0.05 | 5 | 0.75 | **13.383** | 42.091 | 0.663 | 0.897 | 2.149 | 1.666 | 0.800 |
| 220 | 0.05 | 6 | 0.75 | **13.383** | 42.091 | 0.663 | 0.897 | 2.149 | 1.666 | 0.800 |
| 221 | 0.05 | 7 | 0.75 | **13.383** | 42.091 | 0.663 | 0.897 | 2.149 | 1.666 | 0.800 |
| 222 | 0.05 | 8 | 0.75 | **13.383** | 42.091 | 0.663 | 0.897 | 2.149 | 1.666 | 0.800 |
| 223 | 0.05 | 9 | 0.75 | **13.383** | 42.091 | 0.663 | 0.897 | 2.149 | 1.666 | 0.800 |
| 224 | 0.05 | 10 | 0.75 | **13.383** | 42.091 | 0.663 | 0.897 | 2.149 | 1.666 | 0.800 |
| 225 | 0.001 | 1 | 0.75 | **13.445** | 41.825 | 0.654 | 0.871 | 2.416 | 1.884 | 0.747 |
| 226 | 0.05 | 1 | 0.75 | **13.563** | 41.316 | 0.655 | 0.869 | 2.438 | 1.879 | 0.743 |
| 227 | 0.001 | 1 | 0.55 | **13.604** | 41.138 | 0.654 | 0.848 | 2.601 | 2.000 | 0.707 |
| 228 | 0.001 | 2 | 0.55 | **13.604** | 41.138 | 0.654 | 0.865 | 2.466 | 1.888 | 0.737 |
| 229 | 0.001 | 3 | 0.55 | **13.604** | 41.138 | 0.654 | 0.865 | 2.466 | 1.888 | 0.737 |
| 230 | 0.001 | 4 | 0.55 | **13.604** | 41.138 | 0.654 | 0.865 | 2.466 | 1.888 | 0.737 |
| 231 | 0.001 | 5 | 0.55 | **13.604** | 41.138 | 0.654 | 0.865 | 2.466 | 1.888 | 0.737 |
| 232 | 0.001 | 6 | 0.55 | **13.604** | 41.138 | 0.654 | 0.865 | 2.466 | 1.888 | 0.737 |
| 233 | 0.001 | 7 | 0.55 | **13.604** | 41.138 | 0.654 | 0.865 | 2.466 | 1.888 | 0.737 |
| 234 | 0.001 | 8 | 0.55 | **13.604** | 41.138 | 0.654 | 0.865 | 2.466 | 1.888 | 0.737 |
| 235 | 0.001 | 9 | 0.55 | **13.604** | 41.138 | 0.654 | 0.865 | 2.466 | 1.888 | 0.737 |
| 236 | 0.001 | 10 | 0.55 | **13.604** | 41.138 | 0.654 | 0.865 | 2.466 | 1.888 | 0.737 |
| 237 | 0.0005 | 1 | 0.65 | **13.656** | 40.910 | 0.643 | 0.831 | 2.738 | 2.105 | 0.676 |
| 238 | 0.0005 | 1 | 0.70 | **13.763** | 40.448 | 0.636 | 0.835 | 2.711 | 2.088 | 0.682 |
| 239 | 0.0005 | 1 | 0.60 | **13.876** | 39.959 | 0.637 | 0.816 | 2.839 | 2.188 | 0.651 |
| 240 | 0.0005 | 1 | 0.75 | **14.025** | 39.317 | 0.623 | 0.833 | 2.724 | 2.100 | 0.679 |
| 241 | 0.005 | 1 | 0.50 | **14.033** | 39.280 | 0.650 | 0.868 | 2.427 | 1.890 | 0.745 |
| 242 | 0.005 | 2 | 0.50 | **14.033** | 39.280 | 0.650 | 0.868 | 2.427 | 1.890 | 0.745 |
| 243 | 0.005 | 3 | 0.50 | **14.033** | 39.280 | 0.650 | 0.868 | 2.427 | 1.890 | 0.745 |
| 244 | 0.005 | 4 | 0.50 | **14.033** | 39.280 | 0.650 | 0.868 | 2.427 | 1.890 | 0.745 |
| 245 | 0.005 | 5 | 0.50 | **14.033** | 39.280 | 0.650 | 0.868 | 2.427 | 1.890 | 0.745 |
| 246 | 0.005 | 6 | 0.50 | **14.033** | 39.280 | 0.650 | 0.868 | 2.427 | 1.890 | 0.745 |
| 247 | 0.005 | 7 | 0.50 | **14.033** | 39.280 | 0.650 | 0.868 | 2.427 | 1.890 | 0.745 |
| 248 | 0.005 | 8 | 0.50 | **14.033** | 39.280 | 0.650 | 0.868 | 2.427 | 1.890 | 0.745 |
| 249 | 0.005 | 9 | 0.50 | **14.033** | 39.280 | 0.650 | 0.868 | 2.427 | 1.890 | 0.745 |
| 250 | 0.005 | 10 | 0.50 | **14.033** | 39.280 | 0.650 | 0.868 | 2.427 | 1.890 | 0.745 |
| 251 | 0.01 | 1 | 0.50 | **14.060** | 39.161 | 0.648 | 0.869 | 2.418 | 1.888 | 0.747 |
| 252 | 0.01 | 2 | 0.50 | **14.060** | 39.161 | 0.648 | 0.869 | 2.418 | 1.888 | 0.747 |
| 253 | 0.01 | 3 | 0.50 | **14.060** | 39.161 | 0.648 | 0.869 | 2.418 | 1.888 | 0.747 |
| 254 | 0.01 | 4 | 0.50 | **14.060** | 39.161 | 0.648 | 0.869 | 2.418 | 1.888 | 0.747 |
| 255 | 0.01 | 5 | 0.50 | **14.060** | 39.161 | 0.648 | 0.869 | 2.418 | 1.888 | 0.747 |
| 256 | 0.01 | 6 | 0.50 | **14.060** | 39.161 | 0.648 | 0.869 | 2.418 | 1.888 | 0.747 |
| 257 | 0.01 | 7 | 0.50 | **14.060** | 39.161 | 0.648 | 0.869 | 2.418 | 1.888 | 0.747 |
| 258 | 0.01 | 8 | 0.50 | **14.060** | 39.161 | 0.648 | 0.869 | 2.418 | 1.888 | 0.747 |
| 259 | 0.01 | 9 | 0.50 | **14.060** | 39.161 | 0.648 | 0.869 | 2.418 | 1.888 | 0.747 |
| 260 | 0.01 | 10 | 0.50 | **14.060** | 39.161 | 0.648 | 0.869 | 2.418 | 1.888 | 0.747 |
| 261 | 0.0005 | 1 | 0.55 | **14.109** | 38.952 | 0.631 | 0.811 | 2.868 | 2.208 | 0.644 |
| 262 | 0.0005 | 2 | 0.55 | **14.109** | 38.952 | 0.631 | 0.830 | 2.745 | 2.109 | 0.674 |
| 263 | 0.0005 | 3 | 0.55 | **14.109** | 38.952 | 0.631 | 0.830 | 2.745 | 2.109 | 0.674 |
| 264 | 0.0005 | 4 | 0.55 | **14.109** | 38.952 | 0.631 | 0.830 | 2.745 | 2.109 | 0.674 |
| 265 | 0.0005 | 5 | 0.55 | **14.109** | 38.952 | 0.631 | 0.830 | 2.745 | 2.109 | 0.674 |
| 266 | 0.0005 | 6 | 0.55 | **14.109** | 38.952 | 0.631 | 0.830 | 2.745 | 2.109 | 0.674 |
| 267 | 0.0005 | 7 | 0.55 | **14.109** | 38.952 | 0.631 | 0.830 | 2.745 | 2.109 | 0.674 |
| 268 | 0.0005 | 8 | 0.55 | **14.109** | 38.952 | 0.631 | 0.830 | 2.745 | 2.109 | 0.674 |
| 269 | 0.0005 | 9 | 0.55 | **14.109** | 38.952 | 0.631 | 0.830 | 2.745 | 2.109 | 0.674 |
| 270 | 0.0005 | 10 | 0.55 | **14.109** | 38.952 | 0.631 | 0.830 | 2.745 | 2.109 | 0.674 |
| 271 | 0.0001 | 2 | 0.70 | **14.230** | 38.430 | 0.619 | 0.819 | 2.840 | 2.172 | 0.651 |
| 272 | 0.0001 | 3 | 0.70 | **14.230** | 38.430 | 0.619 | 0.819 | 2.840 | 2.172 | 0.651 |
| 273 | 0.0001 | 4 | 0.70 | **14.230** | 38.430 | 0.619 | 0.819 | 2.840 | 2.172 | 0.651 |
| 274 | 0.0001 | 5 | 0.70 | **14.230** | 38.430 | 0.619 | 0.819 | 2.840 | 2.172 | 0.651 |
| 275 | 0.0001 | 6 | 0.70 | **14.230** | 38.430 | 0.619 | 0.819 | 2.840 | 2.172 | 0.651 |
| 276 | 0.0001 | 7 | 0.70 | **14.230** | 38.430 | 0.619 | 0.819 | 2.840 | 2.172 | 0.651 |
| 277 | 0.0001 | 8 | 0.70 | **14.230** | 38.430 | 0.619 | 0.819 | 2.840 | 2.172 | 0.651 |
| 278 | 0.0001 | 9 | 0.70 | **14.230** | 38.430 | 0.619 | 0.819 | 2.840 | 2.172 | 0.651 |
| 279 | 0.0001 | 10 | 0.70 | **14.230** | 38.430 | 0.619 | 0.819 | 2.840 | 2.172 | 0.651 |
| 280 | 0.05 | 1 | 0.50 | **14.273** | 38.243 | 0.651 | 0.880 | 2.324 | 1.795 | 0.766 |
| 281 | 0.05 | 2 | 0.50 | **14.273** | 38.243 | 0.651 | 0.880 | 2.324 | 1.795 | 0.766 |
| 282 | 0.05 | 3 | 0.50 | **14.273** | 38.243 | 0.651 | 0.880 | 2.324 | 1.795 | 0.766 |
| 283 | 0.05 | 4 | 0.50 | **14.273** | 38.243 | 0.651 | 0.880 | 2.324 | 1.795 | 0.766 |
| 284 | 0.05 | 5 | 0.50 | **14.273** | 38.243 | 0.651 | 0.880 | 2.324 | 1.795 | 0.766 |
| 285 | 0.05 | 6 | 0.50 | **14.273** | 38.243 | 0.651 | 0.880 | 2.324 | 1.795 | 0.766 |
| 286 | 0.05 | 7 | 0.50 | **14.273** | 38.243 | 0.651 | 0.880 | 2.324 | 1.795 | 0.766 |
| 287 | 0.05 | 8 | 0.50 | **14.273** | 38.243 | 0.651 | 0.880 | 2.324 | 1.795 | 0.766 |
| 288 | 0.05 | 9 | 0.50 | **14.273** | 38.243 | 0.651 | 0.880 | 2.324 | 1.795 | 0.766 |
| 289 | 0.05 | 10 | 0.50 | **14.273** | 38.243 | 0.651 | 0.880 | 2.324 | 1.795 | 0.766 |
| 290 | 0.0001 | 2 | 0.75 | **14.428** | 37.571 | 0.608 | 0.819 | 2.846 | 2.188 | 0.650 |
| 291 | 0.0001 | 3 | 0.75 | **14.428** | 37.571 | 0.608 | 0.827 | 2.791 | 2.149 | 0.663 |
| 292 | 0.0001 | 4 | 0.75 | **14.428** | 37.571 | 0.608 | 0.827 | 2.791 | 2.149 | 0.663 |
| 293 | 0.0001 | 5 | 0.75 | **14.428** | 37.571 | 0.608 | 0.827 | 2.791 | 2.149 | 0.663 |
| 294 | 0.0001 | 6 | 0.75 | **14.428** | 37.571 | 0.608 | 0.827 | 2.791 | 2.149 | 0.663 |
| 295 | 0.0001 | 7 | 0.75 | **14.428** | 37.571 | 0.608 | 0.827 | 2.791 | 2.149 | 0.663 |
| 296 | 0.0001 | 8 | 0.75 | **14.428** | 37.571 | 0.608 | 0.827 | 2.791 | 2.149 | 0.663 |
| 297 | 0.0001 | 9 | 0.75 | **14.428** | 37.571 | 0.608 | 0.827 | 2.791 | 2.149 | 0.663 |
| 298 | 0.0001 | 10 | 0.75 | **14.428** | 37.571 | 0.608 | 0.827 | 2.791 | 2.149 | 0.663 |
| 299 | 0.0001 | 2 | 0.65 | **14.446** | 37.492 | 0.618 | 0.810 | 2.906 | 2.206 | 0.635 |
| 300 | 0.0001 | 3 | 0.65 | **14.446** | 37.492 | 0.618 | 0.810 | 2.906 | 2.206 | 0.635 |
| 301 | 0.0001 | 4 | 0.65 | **14.446** | 37.492 | 0.618 | 0.810 | 2.906 | 2.206 | 0.635 |
| 302 | 0.0001 | 5 | 0.65 | **14.446** | 37.492 | 0.618 | 0.810 | 2.906 | 2.206 | 0.635 |
| 303 | 0.0001 | 6 | 0.65 | **14.446** | 37.492 | 0.618 | 0.810 | 2.906 | 2.206 | 0.635 |
| 304 | 0.0001 | 7 | 0.65 | **14.446** | 37.492 | 0.618 | 0.810 | 2.906 | 2.206 | 0.635 |
| 305 | 0.0001 | 8 | 0.65 | **14.446** | 37.492 | 0.618 | 0.810 | 2.906 | 2.206 | 0.635 |
| 306 | 0.0001 | 9 | 0.65 | **14.446** | 37.492 | 0.618 | 0.810 | 2.906 | 2.206 | 0.635 |
| 307 | 0.0001 | 10 | 0.65 | **14.446** | 37.492 | 0.618 | 0.810 | 2.906 | 2.206 | 0.635 |
| 308 | 0.0005 | 1 | 0.50 | **14.757** | 36.148 | 0.607 | 0.796 | 2.966 | 2.284 | 0.619 |
| 309 | 0.0005 | 2 | 0.50 | **14.757** | 36.148 | 0.607 | 0.796 | 2.966 | 2.284 | 0.619 |
| 310 | 0.0005 | 3 | 0.50 | **14.757** | 36.148 | 0.607 | 0.796 | 2.966 | 2.284 | 0.619 |
| 311 | 0.0005 | 4 | 0.50 | **14.757** | 36.148 | 0.607 | 0.796 | 2.966 | 2.284 | 0.619 |
| 312 | 0.0005 | 5 | 0.50 | **14.757** | 36.148 | 0.607 | 0.796 | 2.966 | 2.284 | 0.619 |
| 313 | 0.0005 | 6 | 0.50 | **14.757** | 36.148 | 0.607 | 0.796 | 2.966 | 2.284 | 0.619 |
| 314 | 0.0005 | 7 | 0.50 | **14.757** | 36.148 | 0.607 | 0.796 | 2.966 | 2.284 | 0.619 |
| 315 | 0.0005 | 8 | 0.50 | **14.757** | 36.148 | 0.607 | 0.796 | 2.966 | 2.284 | 0.619 |
| 316 | 0.0005 | 9 | 0.50 | **14.757** | 36.148 | 0.607 | 0.796 | 2.966 | 2.284 | 0.619 |
| 317 | 0.0005 | 10 | 0.50 | **14.757** | 36.148 | 0.607 | 0.796 | 2.966 | 2.284 | 0.619 |
| 318 | 0.0001 | 2 | 0.60 | **14.793** | 35.992 | 0.606 | 0.794 | 3.009 | 2.270 | 0.608 |
| 319 | 0.0001 | 3 | 0.60 | **14.793** | 35.992 | 0.606 | 0.794 | 3.009 | 2.270 | 0.608 |
| 320 | 0.0001 | 4 | 0.60 | **14.793** | 35.992 | 0.606 | 0.794 | 3.009 | 2.270 | 0.608 |
| 321 | 0.0001 | 5 | 0.60 | **14.793** | 35.992 | 0.606 | 0.794 | 3.009 | 2.270 | 0.608 |
| 322 | 0.0001 | 6 | 0.60 | **14.793** | 35.992 | 0.606 | 0.794 | 3.009 | 2.270 | 0.608 |
| 323 | 0.0001 | 7 | 0.60 | **14.793** | 35.992 | 0.606 | 0.794 | 3.009 | 2.270 | 0.608 |
| 324 | 0.0001 | 8 | 0.60 | **14.793** | 35.992 | 0.606 | 0.794 | 3.009 | 2.270 | 0.608 |
| 325 | 0.0001 | 9 | 0.60 | **14.793** | 35.992 | 0.606 | 0.794 | 3.009 | 2.270 | 0.608 |
| 326 | 0.0001 | 10 | 0.60 | **14.793** | 35.992 | 0.606 | 0.794 | 3.009 | 2.270 | 0.608 |
| 327 | 0.001 | 1 | 0.50 | **14.818** | 35.883 | 0.605 | 0.795 | 2.974 | 2.289 | 0.617 |
| 328 | 0.001 | 2 | 0.50 | **14.818** | 35.883 | 0.605 | 0.795 | 2.974 | 2.289 | 0.617 |
| 329 | 0.001 | 3 | 0.50 | **14.818** | 35.883 | 0.605 | 0.795 | 2.974 | 2.289 | 0.617 |
| 330 | 0.001 | 4 | 0.50 | **14.818** | 35.883 | 0.605 | 0.795 | 2.974 | 2.289 | 0.617 |
| 331 | 0.001 | 5 | 0.50 | **14.818** | 35.883 | 0.605 | 0.795 | 2.974 | 2.289 | 0.617 |
| 332 | 0.001 | 6 | 0.50 | **14.818** | 35.883 | 0.605 | 0.795 | 2.974 | 2.289 | 0.617 |
| 333 | 0.001 | 7 | 0.50 | **14.818** | 35.883 | 0.605 | 0.795 | 2.974 | 2.289 | 0.617 |
| 334 | 0.001 | 8 | 0.50 | **14.818** | 35.883 | 0.605 | 0.795 | 2.974 | 2.289 | 0.617 |
| 335 | 0.001 | 9 | 0.50 | **14.818** | 35.883 | 0.605 | 0.795 | 2.974 | 2.289 | 0.617 |
| 336 | 0.001 | 10 | 0.50 | **14.818** | 35.883 | 0.605 | 0.795 | 2.974 | 2.289 | 0.617 |
| 337 | 0.0001 | 1 | 0.65 | **15.058** | 34.845 | 0.596 | 0.754 | 3.242 | 2.465 | 0.545 |
| 338 | 0.0001 | 1 | 0.60 | **15.157** | 34.415 | 0.594 | 0.747 | 3.282 | 2.498 | 0.534 |
| 339 | 0.0001 | 1 | 0.70 | **15.314** | 33.736 | 0.584 | 0.744 | 3.301 | 2.515 | 0.529 |
| 340 | 0.0001 | 1 | 0.55 | **15.379** | 33.457 | 0.587 | 0.742 | 3.311 | 2.523 | 0.526 |
| 341 | 0.0001 | 2 | 0.55 | **15.379** | 33.457 | 0.587 | 0.756 | 3.237 | 2.466 | 0.547 |
| 342 | 0.0001 | 3 | 0.55 | **15.379** | 33.457 | 0.587 | 0.756 | 3.237 | 2.466 | 0.547 |
| 343 | 0.0001 | 4 | 0.55 | **15.379** | 33.457 | 0.587 | 0.756 | 3.237 | 2.466 | 0.547 |
| 344 | 0.0001 | 5 | 0.55 | **15.379** | 33.457 | 0.587 | 0.756 | 3.237 | 2.466 | 0.547 |
| 345 | 0.0001 | 6 | 0.55 | **15.379** | 33.457 | 0.587 | 0.756 | 3.237 | 2.466 | 0.547 |
| 346 | 0.0001 | 7 | 0.55 | **15.379** | 33.457 | 0.587 | 0.756 | 3.237 | 2.466 | 0.547 |
| 347 | 0.0001 | 8 | 0.55 | **15.379** | 33.457 | 0.587 | 0.756 | 3.237 | 2.466 | 0.547 |
| 348 | 0.0001 | 9 | 0.55 | **15.379** | 33.457 | 0.587 | 0.756 | 3.237 | 2.466 | 0.547 |
| 349 | 0.0001 | 10 | 0.55 | **15.379** | 33.457 | 0.587 | 0.756 | 3.237 | 2.466 | 0.547 |
| 350 | 0.0001 | 1 | 0.75 | **15.532** | 32.793 | 0.575 | 0.740 | 3.326 | 2.542 | 0.521 |
| 351 | 0.0001 | 1 | 0.50 | **15.758** | 31.818 | 0.574 | 0.740 | 3.316 | 2.527 | 0.524 |
| 352 | 0.0001 | 2 | 0.50 | **15.758** | 31.818 | 0.574 | 0.740 | 3.316 | 2.527 | 0.524 |
| 353 | 0.0001 | 3 | 0.50 | **15.758** | 31.818 | 0.574 | 0.740 | 3.316 | 2.527 | 0.524 |
| 354 | 0.0001 | 4 | 0.50 | **15.758** | 31.818 | 0.574 | 0.740 | 3.316 | 2.527 | 0.524 |
| 355 | 0.0001 | 5 | 0.50 | **15.758** | 31.818 | 0.574 | 0.740 | 3.316 | 2.527 | 0.524 |
| 356 | 0.0001 | 6 | 0.50 | **15.758** | 31.818 | 0.574 | 0.740 | 3.316 | 2.527 | 0.524 |
| 357 | 0.0001 | 7 | 0.50 | **15.758** | 31.818 | 0.574 | 0.740 | 3.316 | 2.527 | 0.524 |
| 358 | 0.0001 | 8 | 0.50 | **15.758** | 31.818 | 0.574 | 0.740 | 3.316 | 2.527 | 0.524 |
| 359 | 0.0001 | 9 | 0.50 | **15.758** | 31.818 | 0.574 | 0.740 | 3.316 | 2.527 | 0.524 |
| 360 | 0.0001 | 10 | 0.50 | **15.758** | 31.818 | 0.574 | 0.740 | 3.316 | 2.527 | 0.524 |

**Table S10.** Possible combinations of the three meta-parameters (learning rate, tree complexity, bag fraction) for fitted 360 BRT models for species richness at 100 m^2^ spatial scale based on 10-fold cross validation (CV) and the associated model performance parameters. Numbers are sorted according to increasing CV deviance (in bold). RMSE represents root mean square error, and MAE represents mean absolute error.

| No. | Learning rate | Tree complexity | Bag fraction | **CV deviance** | Percentage of explained deviance (%) | CV correlation | Training data correlation | RMSE  (%) | MAE  (%) | *R*^2^ |
| --- | --- | --- | --- | --- | --- | --- | --- | --- | --- | --- |
| 1 | 0.05 | 1 | 0.60 | **15.461** | 43.388 | 0.700 | 0.887 | 2.424 | 1.709 | 0.785 |
| 2 | 0.005 | 2 | 0.60 | **15.507** | 43.217 | 0.698 | 0.886 | 2.440 | 1.711 | 0.782 |
| 3 | 0.005 | 3 | 0.60 | **15.507** | 43.217 | 0.698 | 0.886 | 2.440 | 1.711 | 0.782 |
| 4 | 0.005 | 4 | 0.60 | **15.507** | 43.217 | 0.698 | 0.886 | 2.440 | 1.711 | 0.782 |
| 5 | 0.005 | 5 | 0.60 | **15.507** | 43.217 | 0.698 | 0.886 | 2.440 | 1.711 | 0.782 |
| 6 | 0.005 | 6 | 0.60 | **15.507** | 43.217 | 0.698 | 0.886 | 2.440 | 1.711 | 0.782 |
| 7 | 0.005 | 7 | 0.60 | **15.507** | 43.217 | 0.698 | 0.886 | 2.440 | 1.711 | 0.782 |
| 8 | 0.005 | 8 | 0.60 | **15.507** | 43.217 | 0.698 | 0.886 | 2.440 | 1.711 | 0.782 |
| 9 | 0.005 | 9 | 0.60 | **15.507** | 43.217 | 0.698 | 0.886 | 2.440 | 1.711 | 0.782 |
| 10 | 0.005 | 10 | 0.60 | **15.507** | 43.217 | 0.698 | 0.886 | 2.440 | 1.711 | 0.782 |
| 11 | 0.01 | 1 | 0.65 | **15.524** | 43.158 | 0.699 | 0.885 | 2.449 | 1.729 | 0.780 |
| 12 | 0.01 | 2 | 0.60 | **15.575** | 42.971 | 0.699 | 0.887 | 2.427 | 1.687 | 0.784 |
| 13 | 0.01 | 3 | 0.60 | **15.575** | 42.971 | 0.699 | 0.887 | 2.427 | 1.687 | 0.784 |
| 14 | 0.01 | 4 | 0.60 | **15.575** | 42.971 | 0.699 | 0.887 | 2.427 | 1.687 | 0.784 |
| 15 | 0.01 | 5 | 0.60 | **15.575** | 42.971 | 0.699 | 0.887 | 2.427 | 1.687 | 0.784 |
| 16 | 0.01 | 6 | 0.60 | **15.575** | 42.971 | 0.699 | 0.887 | 2.427 | 1.687 | 0.784 |
| 17 | 0.01 | 7 | 0.60 | **15.575** | 42.971 | 0.699 | 0.887 | 2.427 | 1.687 | 0.784 |
| 18 | 0.01 | 8 | 0.60 | **15.575** | 42.971 | 0.699 | 0.887 | 2.427 | 1.687 | 0.784 |
| 19 | 0.01 | 9 | 0.60 | **15.575** | 42.971 | 0.699 | 0.887 | 2.427 | 1.687 | 0.784 |
| 20 | 0.01 | 10 | 0.60 | **15.575** | 42.971 | 0.699 | 0.887 | 2.427 | 1.687 | 0.784 |
| 21 | 0.005 | 1 | 0.70 | **15.601** | 42.873 | 0.694 | 0.878 | 2.520 | 1.765 | 0.767 |
| 22 | 0.005 | 1 | 0.65 | **15.643** | 42.721 | 0.698 | 0.873 | 2.577 | 1.798 | 0.757 |
| 23 | 0.01 | 1 | 0.70 | **15.648** | 42.703 | 0.693 | 0.886 | 2.440 | 1.727 | 0.782 |
| 24 | 0.05 | 1 | 0.65 | **15.684** | 42.572 | 0.695 | 0.888 | 2.421 | 1.696 | 0.785 |
| 25 | 0.01 | 1 | 0.75 | **15.729** | 42.405 | 0.690 | 0.886 | 2.441 | 1.711 | 0.782 |
| 26 | 0.01 | 1 | 0.60 | **15.783** | 42.207 | 0.695 | 0.884 | 2.464 | 1.733 | 0.778 |
| 27 | 0.05 | 2 | 0.60 | **15.785** | 42.200 | 0.698 | 0.886 | 2.438 | 1.701 | 0.782 |
| 28 | 0.05 | 3 | 0.60 | **15.785** | 42.200 | 0.698 | 0.886 | 2.438 | 1.701 | 0.782 |
| 29 | 0.05 | 4 | 0.60 | **15.785** | 42.200 | 0.698 | 0.886 | 2.438 | 1.701 | 0.782 |
| 30 | 0.05 | 5 | 0.60 | **15.785** | 42.200 | 0.698 | 0.886 | 2.438 | 1.701 | 0.782 |
| 31 | 0.05 | 6 | 0.60 | **15.785** | 42.200 | 0.698 | 0.886 | 2.438 | 1.701 | 0.782 |
| 32 | 0.05 | 7 | 0.60 | **15.785** | 42.200 | 0.698 | 0.886 | 2.438 | 1.701 | 0.782 |
| 33 | 0.05 | 8 | 0.60 | **15.785** | 42.200 | 0.698 | 0.886 | 2.438 | 1.701 | 0.782 |
| 34 | 0.05 | 9 | 0.60 | **15.785** | 42.200 | 0.698 | 0.886 | 2.438 | 1.701 | 0.782 |
| 35 | 0.05 | 10 | 0.60 | **15.785** | 42.200 | 0.698 | 0.886 | 2.438 | 1.701 | 0.782 |
| 36 | 0.001 | 1 | 0.65 | **15.786** | 42.197 | 0.697 | 0.864 | 2.668 | 1.873 | 0.739 |
| 37 | 0.005 | 1 | 0.75 | **15.792** | 42.175 | 0.688 | 0.877 | 2.531 | 1.764 | 0.765 |
| 38 | 0.001 | 2 | 0.60 | **15.835** | 42.017 | 0.699 | 0.872 | 2.588 | 1.796 | 0.755 |
| 39 | 0.001 | 3 | 0.60 | **15.835** | 42.017 | 0.699 | 0.872 | 2.588 | 1.796 | 0.755 |
| 40 | 0.001 | 4 | 0.60 | **15.835** | 42.017 | 0.699 | 0.872 | 2.588 | 1.796 | 0.755 |
| 41 | 0.001 | 5 | 0.60 | **15.835** | 42.017 | 0.699 | 0.872 | 2.588 | 1.796 | 0.755 |
| 42 | 0.001 | 6 | 0.60 | **15.835** | 42.017 | 0.699 | 0.872 | 2.588 | 1.796 | 0.755 |
| 43 | 0.001 | 7 | 0.60 | **15.835** | 42.017 | 0.699 | 0.872 | 2.588 | 1.796 | 0.755 |
| 44 | 0.001 | 8 | 0.60 | **15.835** | 42.017 | 0.699 | 0.872 | 2.588 | 1.796 | 0.755 |
| 45 | 0.001 | 9 | 0.60 | **15.835** | 42.017 | 0.699 | 0.872 | 2.588 | 1.796 | 0.755 |
| 46 | 0.001 | 10 | 0.60 | **15.835** | 42.017 | 0.699 | 0.872 | 2.588 | 1.796 | 0.755 |
| 47 | 0.001 | 1 | 0.70 | **15.846** | 41.976 | 0.694 | 0.862 | 2.681 | 1.877 | 0.737 |
| 48 | 0.05 | 2 | 0.65 | **15.848** | 41.972 | 0.697 | 0.880 | 2.501 | 1.762 | 0.771 |
| 49 | 0.05 | 3 | 0.65 | **15.848** | 41.972 | 0.697 | 0.880 | 2.501 | 1.762 | 0.771 |
| 50 | 0.05 | 4 | 0.65 | **15.848** | 41.972 | 0.697 | 0.880 | 2.501 | 1.762 | 0.771 |
| 51 | 0.05 | 5 | 0.65 | **15.848** | 41.972 | 0.697 | 0.880 | 2.501 | 1.762 | 0.771 |
| 52 | 0.05 | 6 | 0.65 | **15.848** | 41.972 | 0.697 | 0.880 | 2.501 | 1.762 | 0.771 |
| 53 | 0.05 | 7 | 0.65 | **15.848** | 41.972 | 0.697 | 0.880 | 2.501 | 1.762 | 0.771 |
| 54 | 0.05 | 8 | 0.65 | **15.848** | 41.972 | 0.697 | 0.880 | 2.501 | 1.762 | 0.771 |
| 55 | 0.05 | 9 | 0.65 | **15.848** | 41.972 | 0.697 | 0.880 | 2.501 | 1.762 | 0.771 |
| 56 | 0.05 | 10 | 0.65 | **15.848** | 41.972 | 0.697 | 0.880 | 2.501 | 1.762 | 0.771 |
| 57 | 0.005 | 1 | 0.60 | **15.864** | 41.912 | 0.696 | 0.867 | 2.633 | 1.834 | 0.746 |
| 58 | 0.001 | 2 | 0.65 | **15.871** | 41.887 | 0.697 | 0.876 | 2.550 | 1.777 | 0.762 |
| 59 | 0.001 | 3 | 0.65 | **15.871** | 41.887 | 0.697 | 0.876 | 2.550 | 1.777 | 0.762 |
| 60 | 0.001 | 4 | 0.65 | **15.871** | 41.887 | 0.697 | 0.876 | 2.550 | 1.777 | 0.762 |
| 61 | 0.001 | 5 | 0.65 | **15.871** | 41.887 | 0.697 | 0.876 | 2.550 | 1.777 | 0.762 |
| 62 | 0.001 | 6 | 0.65 | **15.871** | 41.887 | 0.697 | 0.876 | 2.550 | 1.777 | 0.762 |
| 63 | 0.001 | 7 | 0.65 | **15.871** | 41.887 | 0.697 | 0.876 | 2.550 | 1.777 | 0.762 |
| 64 | 0.001 | 8 | 0.65 | **15.871** | 41.887 | 0.697 | 0.876 | 2.550 | 1.777 | 0.762 |
| 65 | 0.001 | 9 | 0.65 | **15.871** | 41.887 | 0.697 | 0.876 | 2.550 | 1.777 | 0.762 |
| 66 | 0.001 | 10 | 0.65 | **15.871** | 41.887 | 0.697 | 0.876 | 2.550 | 1.777 | 0.762 |
| 67 | 0.05 | 1 | 0.75 | **15.916** | 41.720 | 0.686 | 0.887 | 2.423 | 1.702 | 0.785 |
| 68 | 0.05 | 1 | 0.70 | **15.919** | 41.709 | 0.692 | 0.888 | 2.418 | 1.704 | 0.786 |
| 69 | 0.005 | 2 | 0.65 | **15.925** | 41.688 | 0.696 | 0.887 | 2.426 | 1.698 | 0.785 |
| 70 | 0.005 | 3 | 0.65 | **15.925** | 41.688 | 0.696 | 0.887 | 2.426 | 1.698 | 0.785 |
| 71 | 0.005 | 4 | 0.65 | **15.925** | 41.688 | 0.696 | 0.887 | 2.426 | 1.698 | 0.785 |
| 72 | 0.005 | 5 | 0.65 | **15.925** | 41.688 | 0.696 | 0.887 | 2.426 | 1.698 | 0.785 |
| 73 | 0.005 | 6 | 0.65 | **15.925** | 41.688 | 0.696 | 0.887 | 2.426 | 1.698 | 0.785 |
| 74 | 0.005 | 7 | 0.65 | **15.925** | 41.688 | 0.696 | 0.887 | 2.426 | 1.698 | 0.785 |
| 75 | 0.005 | 8 | 0.65 | **15.925** | 41.688 | 0.696 | 0.887 | 2.426 | 1.698 | 0.785 |
| 76 | 0.005 | 9 | 0.65 | **15.925** | 41.688 | 0.696 | 0.887 | 2.426 | 1.698 | 0.785 |
| 77 | 0.005 | 10 | 0.65 | **15.925** | 41.688 | 0.696 | 0.887 | 2.426 | 1.698 | 0.785 |
| 78 | 0.01 | 2 | 0.65 | **16.009** | 41.380 | 0.695 | 0.878 | 2.527 | 1.760 | 0.766 |
| 79 | 0.01 | 3 | 0.65 | **16.009** | 41.380 | 0.695 | 0.878 | 2.527 | 1.760 | 0.766 |
| 80 | 0.01 | 4 | 0.65 | **16.009** | 41.380 | 0.695 | 0.878 | 2.527 | 1.760 | 0.766 |
| 81 | 0.01 | 5 | 0.65 | **16.009** | 41.380 | 0.695 | 0.878 | 2.527 | 1.760 | 0.766 |
| 82 | 0.01 | 6 | 0.65 | **16.009** | 41.380 | 0.695 | 0.878 | 2.527 | 1.760 | 0.766 |
| 83 | 0.01 | 7 | 0.65 | **16.009** | 41.380 | 0.695 | 0.878 | 2.527 | 1.760 | 0.766 |
| 84 | 0.01 | 8 | 0.65 | **16.009** | 41.380 | 0.695 | 0.878 | 2.527 | 1.760 | 0.766 |
| 85 | 0.01 | 9 | 0.65 | **16.009** | 41.380 | 0.695 | 0.878 | 2.527 | 1.760 | 0.766 |
| 86 | 0.01 | 10 | 0.65 | **16.009** | 41.380 | 0.695 | 0.878 | 2.527 | 1.760 | 0.766 |
| 87 | 0.001 | 1 | 0.75 | **16.009** | 41.379 | 0.686 | 0.863 | 2.677 | 1.877 | 0.738 |
| 88 | 0.01 | 1 | 0.55 | **16.012** | 41.368 | 0.696 | 0.885 | 2.457 | 1.700 | 0.779 |
| 89 | 0.01 | 2 | 0.55 | **16.012** | 41.368 | 0.696 | 0.893 | 2.367 | 1.645 | 0.795 |
| 90 | 0.01 | 3 | 0.55 | **16.012** | 41.368 | 0.696 | 0.893 | 2.367 | 1.645 | 0.795 |
| 91 | 0.01 | 4 | 0.55 | **16.012** | 41.368 | 0.696 | 0.893 | 2.367 | 1.645 | 0.795 |
| 92 | 0.01 | 5 | 0.55 | **16.012** | 41.368 | 0.696 | 0.893 | 2.367 | 1.645 | 0.795 |
| 93 | 0.01 | 6 | 0.55 | **16.012** | 41.368 | 0.696 | 0.893 | 2.367 | 1.645 | 0.795 |
| 94 | 0.01 | 7 | 0.55 | **16.012** | 41.368 | 0.696 | 0.893 | 2.367 | 1.645 | 0.795 |
| 95 | 0.01 | 8 | 0.55 | **16.012** | 41.368 | 0.696 | 0.893 | 2.367 | 1.645 | 0.795 |
| 96 | 0.01 | 9 | 0.55 | **16.012** | 41.368 | 0.696 | 0.893 | 2.367 | 1.645 | 0.795 |
| 97 | 0.01 | 10 | 0.55 | **16.012** | 41.368 | 0.696 | 0.893 | 2.367 | 1.645 | 0.795 |
| 98 | 0.05 | 1 | 0.55 | **16.024** | 41.327 | 0.697 | 0.883 | 2.467 | 1.740 | 0.777 |
| 99 | 0.05 | 2 | 0.55 | **16.024** | 41.327 | 0.697 | 0.890 | 2.388 | 1.701 | 0.791 |
| 100 | 0.05 | 3 | 0.55 | **16.024** | 41.327 | 0.697 | 0.890 | 2.388 | 1.701 | 0.791 |
| 101 | 0.05 | 4 | 0.55 | **16.024** | 41.327 | 0.697 | 0.890 | 2.388 | 1.701 | 0.791 |
| 102 | 0.05 | 5 | 0.55 | **16.024** | 41.327 | 0.697 | 0.890 | 2.388 | 1.701 | 0.791 |
| 103 | 0.05 | 6 | 0.55 | **16.024** | 41.327 | 0.697 | 0.890 | 2.388 | 1.701 | 0.791 |
| 104 | 0.05 | 7 | 0.55 | **16.024** | 41.327 | 0.697 | 0.890 | 2.388 | 1.701 | 0.791 |
| 105 | 0.05 | 8 | 0.55 | **16.024** | 41.327 | 0.697 | 0.890 | 2.388 | 1.701 | 0.791 |
| 106 | 0.05 | 9 | 0.55 | **16.024** | 41.327 | 0.697 | 0.890 | 2.388 | 1.701 | 0.791 |
| 107 | 0.05 | 10 | 0.55 | **16.024** | 41.327 | 0.697 | 0.890 | 2.388 | 1.701 | 0.791 |
| 108 | 0.0005 | 2 | 0.65 | **16.037** | 41.278 | 0.695 | 0.868 | 2.629 | 1.830 | 0.747 |
| 109 | 0.0005 | 3 | 0.65 | **16.037** | 41.278 | 0.695 | 0.868 | 2.629 | 1.830 | 0.747 |
| 110 | 0.0005 | 4 | 0.65 | **16.037** | 41.278 | 0.695 | 0.868 | 2.629 | 1.830 | 0.747 |
| 111 | 0.0005 | 5 | 0.65 | **16.037** | 41.278 | 0.695 | 0.868 | 2.629 | 1.830 | 0.747 |
| 112 | 0.0005 | 6 | 0.65 | **16.037** | 41.278 | 0.695 | 0.868 | 2.629 | 1.830 | 0.747 |
| 113 | 0.0005 | 7 | 0.65 | **16.037** | 41.278 | 0.695 | 0.868 | 2.629 | 1.830 | 0.747 |
| 114 | 0.0005 | 8 | 0.65 | **16.037** | 41.278 | 0.695 | 0.868 | 2.629 | 1.830 | 0.747 |
| 115 | 0.0005 | 9 | 0.65 | **16.037** | 41.278 | 0.695 | 0.868 | 2.629 | 1.830 | 0.747 |
| 116 | 0.0005 | 10 | 0.65 | **16.037** | 41.278 | 0.695 | 0.868 | 2.629 | 1.830 | 0.747 |
| 117 | 0.0005 | 2 | 0.60 | **16.085** | 41.101 | 0.697 | 0.861 | 2.699 | 1.884 | 0.733 |
| 118 | 0.0005 | 3 | 0.60 | **16.085** | 41.101 | 0.697 | 0.861 | 2.699 | 1.884 | 0.733 |
| 119 | 0.0005 | 4 | 0.60 | **16.085** | 41.101 | 0.697 | 0.861 | 2.699 | 1.884 | 0.733 |
| 120 | 0.0005 | 5 | 0.60 | **16.085** | 41.101 | 0.697 | 0.861 | 2.699 | 1.884 | 0.733 |
| 121 | 0.0005 | 6 | 0.60 | **16.085** | 41.101 | 0.697 | 0.861 | 2.699 | 1.884 | 0.733 |
| 122 | 0.0005 | 7 | 0.60 | **16.085** | 41.101 | 0.697 | 0.861 | 2.699 | 1.884 | 0.733 |
| 123 | 0.0005 | 8 | 0.60 | **16.085** | 41.101 | 0.697 | 0.861 | 2.699 | 1.884 | 0.733 |
| 124 | 0.0005 | 9 | 0.60 | **16.085** | 41.101 | 0.697 | 0.861 | 2.699 | 1.884 | 0.733 |
| 125 | 0.0005 | 10 | 0.60 | **16.085** | 41.101 | 0.697 | 0.861 | 2.699 | 1.884 | 0.733 |
| 126 | 0.005 | 2 | 0.70 | **16.21** | 40.643 | 0.690 | 0.875 | 2.554 | 1.793 | 0.761 |
| 127 | 0.005 | 3 | 0.70 | **16.21** | 40.643 | 0.690 | 0.875 | 2.554 | 1.793 | 0.761 |
| 128 | 0.005 | 4 | 0.70 | **16.21** | 40.643 | 0.690 | 0.875 | 2.554 | 1.793 | 0.761 |
| 129 | 0.005 | 5 | 0.70 | **16.21** | 40.643 | 0.690 | 0.875 | 2.554 | 1.793 | 0.761 |
| 130 | 0.005 | 6 | 0.70 | **16.21** | 40.643 | 0.690 | 0.875 | 2.554 | 1.793 | 0.761 |
| 131 | 0.005 | 7 | 0.70 | **16.21** | 40.643 | 0.690 | 0.875 | 2.554 | 1.793 | 0.761 |
| 132 | 0.005 | 8 | 0.70 | **16.21** | 40.643 | 0.690 | 0.875 | 2.554 | 1.793 | 0.761 |
| 133 | 0.005 | 9 | 0.70 | **16.21** | 40.643 | 0.690 | 0.875 | 2.554 | 1.793 | 0.761 |
| 134 | 0.005 | 10 | 0.70 | **16.21** | 40.643 | 0.690 | 0.875 | 2.554 | 1.793 | 0.761 |
| 135 | 0.005 | 1 | 0.55 | **16.287** | 40.364 | 0.693 | 0.861 | 2.697 | 1.889 | 0.734 |
| 136 | 0.005 | 2 | 0.55 | **16.287** | 40.364 | 0.693 | 0.873 | 2.577 | 1.791 | 0.757 |
| 137 | 0.005 | 3 | 0.55 | **16.287** | 40.364 | 0.693 | 0.873 | 2.577 | 1.791 | 0.757 |
| 138 | 0.005 | 4 | 0.55 | **16.287** | 40.364 | 0.693 | 0.873 | 2.577 | 1.791 | 0.757 |
| 139 | 0.005 | 5 | 0.55 | **16.287** | 40.364 | 0.693 | 0.873 | 2.577 | 1.791 | 0.757 |
| 140 | 0.005 | 6 | 0.55 | **16.287** | 40.364 | 0.693 | 0.873 | 2.577 | 1.791 | 0.757 |
| 141 | 0.005 | 7 | 0.55 | **16.287** | 40.364 | 0.693 | 0.873 | 2.577 | 1.791 | 0.757 |
| 142 | 0.005 | 8 | 0.55 | **16.287** | 40.364 | 0.693 | 0.873 | 2.577 | 1.791 | 0.757 |
| 143 | 0.005 | 9 | 0.55 | **16.287** | 40.364 | 0.693 | 0.873 | 2.577 | 1.791 | 0.757 |
| 144 | 0.005 | 10 | 0.55 | **16.287** | 40.364 | 0.693 | 0.873 | 2.577 | 1.791 | 0.757 |
| 145 | 0.001 | 1 | 0.60 | **16.317** | 40.252 | 0.692 | 0.840 | 2.872 | 2.065 | 0.698 |
| 146 | 0.0005 | 2 | 0.70 | **16.328** | 40.212 | 0.689 | 0.868 | 2.627 | 1.833 | 0.747 |
| 147 | 0.0005 | 3 | 0.70 | **16.328** | 40.212 | 0.689 | 0.868 | 2.627 | 1.833 | 0.747 |
| 148 | 0.0005 | 4 | 0.70 | **16.328** | 40.212 | 0.689 | 0.868 | 2.627 | 1.833 | 0.747 |
| 149 | 0.0005 | 5 | 0.70 | **16.328** | 40.212 | 0.689 | 0.868 | 2.627 | 1.833 | 0.747 |
| 150 | 0.0005 | 6 | 0.70 | **16.328** | 40.212 | 0.689 | 0.868 | 2.627 | 1.833 | 0.747 |
| 151 | 0.0005 | 7 | 0.70 | **16.328** | 40.212 | 0.689 | 0.868 | 2.627 | 1.833 | 0.747 |
| 152 | 0.0005 | 8 | 0.70 | **16.328** | 40.212 | 0.689 | 0.868 | 2.627 | 1.833 | 0.747 |
| 153 | 0.0005 | 9 | 0.70 | **16.328** | 40.212 | 0.689 | 0.868 | 2.627 | 1.833 | 0.747 |
| 154 | 0.0005 | 10 | 0.70 | **16.328** | 40.212 | 0.689 | 0.868 | 2.627 | 1.833 | 0.747 |
| 155 | 0.001 | 2 | 0.70 | **16.369** | 40.063 | 0.689 | 0.872 | 2.590 | 1.812 | 0.754 |
| 156 | 0.001 | 3 | 0.70 | **16.369** | 40.063 | 0.689 | 0.872 | 2.590 | 1.812 | 0.754 |
| 157 | 0.001 | 4 | 0.70 | **16.369** | 40.063 | 0.689 | 0.872 | 2.590 | 1.812 | 0.754 |
| 158 | 0.001 | 5 | 0.70 | **16.369** | 40.063 | 0.689 | 0.872 | 2.590 | 1.812 | 0.754 |
| 159 | 0.001 | 6 | 0.70 | **16.369** | 40.063 | 0.689 | 0.872 | 2.590 | 1.812 | 0.754 |
| 160 | 0.001 | 7 | 0.70 | **16.369** | 40.063 | 0.689 | 0.872 | 2.590 | 1.812 | 0.754 |
| 161 | 0.001 | 8 | 0.70 | **16.369** | 40.063 | 0.689 | 0.872 | 2.590 | 1.812 | 0.754 |
| 162 | 0.001 | 9 | 0.70 | **16.369** | 40.063 | 0.689 | 0.872 | 2.590 | 1.812 | 0.754 |
| 163 | 0.001 | 10 | 0.70 | **16.369** | 40.063 | 0.689 | 0.872 | 2.590 | 1.812 | 0.754 |
| 164 | 0.01 | 2 | 0.70 | **16.384** | 40.007 | 0.687 | 0.881 | 2.499 | 1.753 | 0.771 |
| 165 | 0.01 | 3 | 0.70 | **16.384** | 40.007 | 0.687 | 0.881 | 2.499 | 1.753 | 0.771 |
| 166 | 0.01 | 4 | 0.70 | **16.384** | 40.007 | 0.687 | 0.881 | 2.499 | 1.753 | 0.771 |
| 167 | 0.01 | 5 | 0.70 | **16.384** | 40.007 | 0.687 | 0.881 | 2.499 | 1.753 | 0.771 |
| 168 | 0.01 | 6 | 0.70 | **16.384** | 40.007 | 0.687 | 0.881 | 2.499 | 1.753 | 0.771 |
| 169 | 0.01 | 7 | 0.70 | **16.384** | 40.007 | 0.687 | 0.881 | 2.499 | 1.753 | 0.771 |
| 170 | 0.01 | 8 | 0.70 | **16.384** | 40.007 | 0.687 | 0.881 | 2.499 | 1.753 | 0.771 |
| 171 | 0.01 | 9 | 0.70 | **16.384** | 40.007 | 0.687 | 0.881 | 2.499 | 1.753 | 0.771 |
| 172 | 0.01 | 10 | 0.70 | **16.384** | 40.007 | 0.687 | 0.881 | 2.499 | 1.753 | 0.771 |
| 173 | 0.0005 | 1 | 0.65 | **16.486** | 39.632 | 0.69 | 0.832 | 2.945 | 2.132 | 0.682 |
| 174 | 0.0005 | 1 | 0.70 | **16.579** | 39.294 | 0.687 | 0.831 | 2.948 | 2.143 | 0.682 |
| 175 | 0.0005 | 1 | 0.60 | **16.585** | 39.271 | 0.688 | 0.820 | 3.030 | 2.197 | 0.664 |
| 176 | 0.01 | 1 | 0.50 | **16.634** | 39.093 | 0.690 | 0.860 | 2.698 | 1.893 | 0.733 |
| 177 | 0.01 | 2 | 0.50 | **16.634** | 39.093 | 0.690 | 0.860 | 2.698 | 1.893 | 0.733 |
| 178 | 0.01 | 3 | 0.50 | **16.634** | 39.093 | 0.690 | 0.860 | 2.698 | 1.893 | 0.733 |
| 179 | 0.01 | 4 | 0.50 | **16.634** | 39.093 | 0.690 | 0.860 | 2.698 | 1.893 | 0.733 |
| 180 | 0.01 | 5 | 0.50 | **16.634** | 39.093 | 0.690 | 0.860 | 2.698 | 1.893 | 0.733 |
| 181 | 0.01 | 6 | 0.50 | **16.634** | 39.093 | 0.690 | 0.860 | 2.698 | 1.893 | 0.733 |
| 182 | 0.01 | 7 | 0.50 | **16.634** | 39.093 | 0.690 | 0.860 | 2.698 | 1.893 | 0.733 |
| 183 | 0.01 | 8 | 0.50 | **16.634** | 39.093 | 0.690 | 0.860 | 2.698 | 1.893 | 0.733 |
| 184 | 0.01 | 9 | 0.50 | **16.634** | 39.093 | 0.690 | 0.860 | 2.698 | 1.893 | 0.733 |
| 185 | 0.01 | 10 | 0.50 | **16.634** | 39.093 | 0.690 | 0.860 | 2.698 | 1.893 | 0.733 |
| 186 | 0.0005 | 1 | 0.75 | **16.654** | 39.020 | 0.681 | 0.836 | 2.909 | 2.112 | 0.690 |
| 187 | 0.001 | 1 | 0.55 | **16.698** | 38.859 | 0.686 | 0.829 | 2.962 | 2.130 | 0.679 |
| 188 | 0.001 | 2 | 0.55 | **16.698** | 38.859 | 0.686 | 0.845 | 2.833 | 2.016 | 0.706 |
| 189 | 0.001 | 3 | 0.55 | **16.698** | 38.859 | 0.686 | 0.845 | 2.833 | 2.016 | 0.706 |
| 190 | 0.001 | 4 | 0.55 | **16.698** | 38.859 | 0.686 | 0.845 | 2.833 | 2.016 | 0.706 |
| 191 | 0.001 | 5 | 0.55 | **16.698** | 38.859 | 0.686 | 0.845 | 2.833 | 2.016 | 0.706 |
| 192 | 0.001 | 6 | 0.55 | **16.698** | 38.859 | 0.686 | 0.845 | 2.833 | 2.016 | 0.706 |
| 193 | 0.001 | 7 | 0.55 | **16.698** | 38.859 | 0.686 | 0.845 | 2.833 | 2.016 | 0.706 |
| 194 | 0.001 | 8 | 0.55 | **16.698** | 38.859 | 0.686 | 0.845 | 2.833 | 2.016 | 0.706 |
| 195 | 0.001 | 9 | 0.55 | **16.698** | 38.859 | 0.686 | 0.845 | 2.833 | 2.016 | 0.706 |
| 196 | 0.001 | 10 | 0.55 | **16.698** | 38.859 | 0.686 | 0.845 | 2.833 | 2.016 | 0.706 |
| 197 | 0.001 | 2 | 0.75 | **16.778** | 38.565 | 0.679 | 0.873 | 2.579 | 1.808 | 0.756 |
| 198 | 0.001 | 3 | 0.75 | **16.778** | 38.565 | 0.679 | 0.876 | 2.545 | 1.785 | 0.763 |
| 199 | 0.001 | 4 | 0.75 | **16.778** | 38.565 | 0.679 | 0.876 | 2.545 | 1.785 | 0.763 |
| 200 | 0.001 | 5 | 0.75 | **16.778** | 38.565 | 0.679 | 0.876 | 2.545 | 1.785 | 0.763 |
| 201 | 0.001 | 6 | 0.75 | **16.778** | 38.565 | 0.679 | 0.876 | 2.545 | 1.785 | 0.763 |
| 202 | 0.001 | 7 | 0.75 | **16.778** | 38.565 | 0.679 | 0.876 | 2.545 | 1.785 | 0.763 |
| 203 | 0.001 | 8 | 0.75 | **16.778** | 38.565 | 0.679 | 0.876 | 2.545 | 1.785 | 0.763 |
| 204 | 0.001 | 9 | 0.75 | **16.778** | 38.565 | 0.679 | 0.876 | 2.545 | 1.785 | 0.763 |
| 205 | 0.001 | 10 | 0.75 | **16.778** | 38.565 | 0.679 | 0.876 | 2.545 | 1.785 | 0.763 |
| 206 | 0.0005 | 2 | 0.75 | **16.847** | 38.313 | 0.676 | 0.866 | 2.650 | 1.851 | 0.743 |
| 207 | 0.0005 | 3 | 0.75 | **16.847** | 38.313 | 0.676 | 0.870 | 2.613 | 1.827 | 0.750 |
| 208 | 0.0005 | 4 | 0.75 | **16.847** | 38.313 | 0.676 | 0.870 | 2.613 | 1.827 | 0.750 |
| 209 | 0.0005 | 5 | 0.75 | **16.847** | 38.313 | 0.676 | 0.870 | 2.613 | 1.827 | 0.750 |
| 210 | 0.0005 | 6 | 0.75 | **16.847** | 38.313 | 0.676 | 0.870 | 2.613 | 1.827 | 0.750 |
| 211 | 0.0005 | 7 | 0.75 | **16.847** | 38.313 | 0.676 | 0.870 | 2.613 | 1.827 | 0.750 |
| 212 | 0.0005 | 8 | 0.75 | **16.847** | 38.313 | 0.676 | 0.870 | 2.613 | 1.827 | 0.750 |
| 213 | 0.0005 | 9 | 0.75 | **16.847** | 38.313 | 0.676 | 0.870 | 2.613 | 1.827 | 0.750 |
| 214 | 0.0005 | 10 | 0.75 | **16.847** | 38.313 | 0.676 | 0.870 | 2.613 | 1.827 | 0.750 |
| 215 | 0.005 | 2 | 0.75 | **16.881** | 38.187 | 0.675 | 0.876 | 2.548 | 1.783 | 0.762 |
| 216 | 0.005 | 3 | 0.75 | **16.881** | 38.187 | 0.675 | 0.879 | 2.515 | 1.759 | 0.768 |
| 217 | 0.005 | 4 | 0.75 | **16.881** | 38.187 | 0.675 | 0.879 | 2.515 | 1.759 | 0.768 |
| 218 | 0.005 | 5 | 0.75 | **16.881** | 38.187 | 0.675 | 0.879 | 2.515 | 1.759 | 0.768 |
| 219 | 0.005 | 6 | 0.75 | **16.881** | 38.187 | 0.675 | 0.879 | 2.515 | 1.759 | 0.768 |
| 220 | 0.005 | 7 | 0.75 | **16.881** | 38.187 | 0.675 | 0.879 | 2.515 | 1.759 | 0.768 |
| 221 | 0.005 | 8 | 0.75 | **16.881** | 38.187 | 0.675 | 0.879 | 2.515 | 1.759 | 0.768 |
| 222 | 0.005 | 9 | 0.75 | **16.881** | 38.187 | 0.675 | 0.879 | 2.515 | 1.759 | 0.768 |
| 223 | 0.005 | 10 | 0.75 | **16.881** | 38.187 | 0.675 | 0.879 | 2.515 | 1.759 | 0.768 |
| 224 | 0.01 | 2 | 0.75 | **16.921** | 38.040 | 0.676 | 0.873 | 2.571 | 1.806 | 0.758 |
| 225 | 0.01 | 3 | 0.75 | **16.921** | 38.040 | 0.676 | 0.878 | 2.529 | 1.773 | 0.766 |
| 226 | 0.01 | 4 | 0.75 | **16.921** | 38.040 | 0.676 | 0.878 | 2.529 | 1.773 | 0.766 |
| 227 | 0.01 | 5 | 0.75 | **16.921** | 38.040 | 0.676 | 0.878 | 2.529 | 1.773 | 0.766 |
| 228 | 0.01 | 6 | 0.75 | **16.921** | 38.040 | 0.676 | 0.878 | 2.529 | 1.773 | 0.766 |
| 229 | 0.01 | 7 | 0.75 | **16.921** | 38.040 | 0.676 | 0.878 | 2.529 | 1.773 | 0.766 |
| 230 | 0.01 | 8 | 0.75 | **16.921** | 38.040 | 0.676 | 0.878 | 2.529 | 1.773 | 0.766 |
| 231 | 0.01 | 9 | 0.75 | **16.921** | 38.040 | 0.676 | 0.878 | 2.529 | 1.773 | 0.766 |
| 232 | 0.01 | 10 | 0.75 | **16.921** | 38.040 | 0.676 | 0.878 | 2.529 | 1.773 | 0.766 |
| 233 | 0.005 | 1 | 0.50 | **16.952** | 37.926 | 0.681 | 0.820 | 3.021 | 2.178 | 0.666 |
| 234 | 0.005 | 2 | 0.50 | **16.952** | 37.926 | 0.681 | 0.820 | 3.021 | 2.178 | 0.666 |
| 235 | 0.005 | 3 | 0.50 | **16.952** | 37.926 | 0.681 | 0.820 | 3.021 | 2.178 | 0.666 |
| 236 | 0.005 | 4 | 0.50 | **16.952** | 37.926 | 0.681 | 0.820 | 3.021 | 2.178 | 0.666 |
| 237 | 0.005 | 5 | 0.50 | **16.952** | 37.926 | 0.681 | 0.820 | 3.021 | 2.178 | 0.666 |
| 238 | 0.005 | 6 | 0.50 | **16.952** | 37.926 | 0.681 | 0.820 | 3.021 | 2.178 | 0.666 |
| 239 | 0.005 | 7 | 0.50 | **16.952** | 37.926 | 0.681 | 0.820 | 3.021 | 2.178 | 0.666 |
| 240 | 0.005 | 8 | 0.50 | **16.952** | 37.926 | 0.681 | 0.820 | 3.021 | 2.178 | 0.666 |
| 241 | 0.005 | 9 | 0.50 | **16.952** | 37.926 | 0.681 | 0.820 | 3.021 | 2.178 | 0.666 |
| 242 | 0.005 | 10 | 0.50 | **16.952** | 37.926 | 0.681 | 0.820 | 3.021 | 2.178 | 0.666 |
| 243 | 0.001 | 1 | 0.50 | **16.969** | 37.866 | 0.674 | 0.802 | 3.151 | 2.279 | 0.636 |
| 244 | 0.001 | 2 | 0.50 | **16.969** | 37.866 | 0.674 | 0.802 | 3.151 | 2.279 | 0.636 |
| 245 | 0.001 | 3 | 0.50 | **16.969** | 37.866 | 0.674 | 0.802 | 3.151 | 2.279 | 0.636 |
| 246 | 0.001 | 4 | 0.50 | **16.969** | 37.866 | 0.674 | 0.802 | 3.151 | 2.279 | 0.636 |
| 247 | 0.001 | 5 | 0.50 | **16.969** | 37.866 | 0.674 | 0.802 | 3.151 | 2.279 | 0.636 |
| 248 | 0.001 | 6 | 0.50 | **16.969** | 37.866 | 0.674 | 0.802 | 3.151 | 2.279 | 0.636 |
| 249 | 0.001 | 7 | 0.50 | **16.969** | 37.866 | 0.674 | 0.802 | 3.151 | 2.279 | 0.636 |
| 250 | 0.001 | 8 | 0.50 | **16.969** | 37.866 | 0.674 | 0.802 | 3.151 | 2.279 | 0.636 |
| 251 | 0.001 | 9 | 0.50 | **16.969** | 37.866 | 0.674 | 0.802 | 3.151 | 2.279 | 0.636 |
| 252 | 0.001 | 10 | 0.50 | **16.969** | 37.866 | 0.674 | 0.802 | 3.151 | 2.279 | 0.636 |
| 253 | 0.0005 | 1 | 0.55 | **17.148** | 37.212 | 0.672 | 0.789 | 3.255 | 2.368 | 0.612 |
| 254 | 0.0005 | 2 | 0.55 | **17.148** | 37.212 | 0.672 | 0.803 | 3.161 | 2.293 | 0.634 |
| 255 | 0.0005 | 3 | 0.55 | **17.148** | 37.212 | 0.672 | 0.803 | 3.161 | 2.293 | 0.634 |
| 256 | 0.0005 | 4 | 0.55 | **17.148** | 37.212 | 0.672 | 0.803 | 3.161 | 2.293 | 0.634 |
| 257 | 0.0005 | 5 | 0.55 | **17.148** | 37.212 | 0.672 | 0.803 | 3.161 | 2.293 | 0.634 |
| 258 | 0.0005 | 6 | 0.55 | **17.148** | 37.212 | 0.672 | 0.803 | 3.161 | 2.293 | 0.634 |
| 259 | 0.0005 | 7 | 0.55 | **17.148** | 37.212 | 0.672 | 0.803 | 3.161 | 2.293 | 0.634 |
| 260 | 0.0005 | 8 | 0.55 | **17.148** | 37.212 | 0.672 | 0.803 | 3.161 | 2.293 | 0.634 |
| 261 | 0.0005 | 9 | 0.55 | **17.148** | 37.212 | 0.672 | 0.803 | 3.161 | 2.293 | 0.634 |
| 262 | 0.0005 | 10 | 0.55 | **17.148** | 37.212 | 0.672 | 0.803 | 3.161 | 2.293 | 0.634 |
| 263 | 0.0005 | 1 | 0.50 | **17.225** | 36.928 | 0.666 | 0.781 | 3.299 | 2.401 | 0.601 |
| 264 | 0.0005 | 2 | 0.50 | **17.225** | 36.928 | 0.666 | 0.781 | 3.299 | 2.401 | 0.601 |
| 265 | 0.0005 | 3 | 0.50 | **17.225** | 36.928 | 0.666 | 0.781 | 3.299 | 2.401 | 0.601 |
| 266 | 0.0005 | 4 | 0.50 | **17.225** | 36.928 | 0.666 | 0.781 | 3.299 | 2.401 | 0.601 |
| 267 | 0.0005 | 5 | 0.50 | **17.225** | 36.928 | 0.666 | 0.781 | 3.299 | 2.401 | 0.601 |
| 268 | 0.0005 | 6 | 0.50 | **17.225** | 36.928 | 0.666 | 0.781 | 3.299 | 2.401 | 0.601 |
| 269 | 0.0005 | 7 | 0.50 | **17.225** | 36.928 | 0.666 | 0.781 | 3.299 | 2.401 | 0.601 |
| 270 | 0.0005 | 8 | 0.50 | **17.225** | 36.928 | 0.666 | 0.781 | 3.299 | 2.401 | 0.601 |
| 271 | 0.0005 | 9 | 0.50 | **17.225** | 36.928 | 0.666 | 0.781 | 3.299 | 2.401 | 0.601 |
| 272 | 0.0005 | 10 | 0.50 | **17.225** | 36.928 | 0.666 | 0.781 | 3.299 | 2.401 | 0.601 |
| 273 | 0.0001 | 2 | 0.65 | **17.64** | 35.408 | 0.669 | 0.797 | 3.259 | 2.401 | 0.611 |
| 274 | 0.0001 | 3 | 0.65 | **17.64** | 35.408 | 0.669 | 0.797 | 3.259 | 2.401 | 0.611 |
| 275 | 0.0001 | 4 | 0.65 | **17.64** | 35.408 | 0.669 | 0.797 | 3.259 | 2.401 | 0.611 |
| 276 | 0.0001 | 5 | 0.65 | **17.64** | 35.408 | 0.669 | 0.797 | 3.259 | 2.401 | 0.611 |
| 277 | 0.0001 | 6 | 0.65 | **17.64** | 35.408 | 0.669 | 0.797 | 3.259 | 2.401 | 0.611 |
| 278 | 0.0001 | 7 | 0.65 | **17.64** | 35.408 | 0.669 | 0.797 | 3.259 | 2.401 | 0.611 |
| 279 | 0.0001 | 8 | 0.65 | **17.64** | 35.408 | 0.669 | 0.797 | 3.259 | 2.401 | 0.611 |
| 280 | 0.0001 | 9 | 0.65 | **17.64** | 35.408 | 0.669 | 0.797 | 3.259 | 2.401 | 0.611 |
| 281 | 0.0001 | 10 | 0.65 | **17.64** | 35.408 | 0.669 | 0.797 | 3.259 | 2.401 | 0.611 |
| 282 | 0.0001 | 2 | 0.60 | **17.699** | 35.192 | 0.665 | 0.785 | 3.333 | 2.474 | 0.593 |
| 283 | 0.0001 | 3 | 0.60 | **17.699** | 35.192 | 0.665 | 0.785 | 3.333 | 2.474 | 0.593 |
| 284 | 0.0001 | 4 | 0.60 | **17.699** | 35.192 | 0.665 | 0.785 | 3.333 | 2.474 | 0.593 |
| 285 | 0.0001 | 5 | 0.60 | **17.699** | 35.192 | 0.665 | 0.785 | 3.333 | 2.474 | 0.593 |
| 286 | 0.0001 | 6 | 0.60 | **17.699** | 35.192 | 0.665 | 0.785 | 3.333 | 2.474 | 0.593 |
| 287 | 0.0001 | 7 | 0.60 | **17.699** | 35.192 | 0.665 | 0.785 | 3.333 | 2.474 | 0.593 |
| 288 | 0.0001 | 8 | 0.60 | **17.699** | 35.192 | 0.665 | 0.785 | 3.333 | 2.474 | 0.593 |
| 289 | 0.0001 | 9 | 0.60 | **17.699** | 35.192 | 0.665 | 0.785 | 3.333 | 2.474 | 0.593 |
| 290 | 0.0001 | 10 | 0.60 | **17.699** | 35.192 | 0.665 | 0.785 | 3.333 | 2.474 | 0.593 |
| 291 | 0.0001 | 2 | 0.70 | **17.84** | 34.677 | 0.663 | 0.804 | 3.208 | 2.372 | 0.623 |
| 292 | 0.0001 | 3 | 0.70 | **17.84** | 34.677 | 0.663 | 0.804 | 3.208 | 2.372 | 0.623 |
| 293 | 0.0001 | 4 | 0.70 | **17.84** | 34.677 | 0.663 | 0.804 | 3.208 | 2.372 | 0.623 |
| 294 | 0.0001 | 5 | 0.70 | **17.84** | 34.677 | 0.663 | 0.804 | 3.208 | 2.372 | 0.623 |
| 295 | 0.0001 | 6 | 0.70 | **17.84** | 34.677 | 0.663 | 0.804 | 3.208 | 2.372 | 0.623 |
| 296 | 0.0001 | 7 | 0.70 | **17.84** | 34.677 | 0.663 | 0.804 | 3.208 | 2.372 | 0.623 |
| 297 | 0.0001 | 8 | 0.70 | **17.84** | 34.677 | 0.663 | 0.804 | 3.208 | 2.372 | 0.623 |
| 298 | 0.0001 | 9 | 0.70 | **17.84** | 34.677 | 0.663 | 0.804 | 3.208 | 2.372 | 0.623 |
| 299 | 0.0001 | 10 | 0.70 | **17.84** | 34.677 | 0.663 | 0.804 | 3.208 | 2.372 | 0.623 |
| 300 | 0.0001 | 1 | 0.50 | **17.934** | 34.333 | 0.655 | 0.744 | 3.582 | 2.735 | 0.530 |
| 301 | 0.0001 | 2 | 0.50 | **17.934** | 34.333 | 0.655 | 0.744 | 3.582 | 2.735 | 0.530 |
| 302 | 0.0001 | 3 | 0.50 | **17.934** | 34.333 | 0.655 | 0.744 | 3.582 | 2.735 | 0.530 |
| 303 | 0.0001 | 4 | 0.50 | **17.934** | 34.333 | 0.655 | 0.744 | 3.582 | 2.735 | 0.530 |
| 304 | 0.0001 | 5 | 0.50 | **17.934** | 34.333 | 0.655 | 0.744 | 3.582 | 2.735 | 0.530 |
| 305 | 0.0001 | 6 | 0.50 | **17.934** | 34.333 | 0.655 | 0.744 | 3.582 | 2.735 | 0.530 |
| 306 | 0.0001 | 7 | 0.50 | **17.934** | 34.333 | 0.655 | 0.744 | 3.582 | 2.735 | 0.530 |
| 307 | 0.0001 | 8 | 0.50 | **17.934** | 34.333 | 0.655 | 0.744 | 3.582 | 2.735 | 0.530 |
| 308 | 0.0001 | 9 | 0.50 | **17.934** | 34.333 | 0.655 | 0.744 | 3.582 | 2.735 | 0.530 |
| 309 | 0.0001 | 10 | 0.50 | **17.934** | 34.333 | 0.655 | 0.744 | 3.582 | 2.735 | 0.530 |
| 310 | 0.0001 | 1 | 0.60 | **17.934** | 34.331 | 0.660 | 0.752 | 3.538 | 2.711 | 0.542 |
| 311 | 0.0001 | 1 | 0.55 | **17.968** | 34.207 | 0.656 | 0.747 | 3.569 | 2.733 | 0.534 |
| 312 | 0.0001 | 2 | 0.55 | **17.968** | 34.207 | 0.656 | 0.759 | 3.505 | 2.656 | 0.550 |
| 313 | 0.0001 | 3 | 0.55 | **17.968** | 34.207 | 0.656 | 0.759 | 3.505 | 2.656 | 0.550 |
| 314 | 0.0001 | 4 | 0.55 | **17.968** | 34.207 | 0.656 | 0.759 | 3.505 | 2.656 | 0.550 |
| 315 | 0.0001 | 5 | 0.55 | **17.968** | 34.207 | 0.656 | 0.759 | 3.505 | 2.656 | 0.550 |
| 316 | 0.0001 | 6 | 0.55 | **17.968** | 34.207 | 0.656 | 0.759 | 3.505 | 2.656 | 0.550 |
| 317 | 0.0001 | 7 | 0.55 | **17.968** | 34.207 | 0.656 | 0.759 | 3.505 | 2.656 | 0.550 |
| 318 | 0.0001 | 8 | 0.55 | **17.968** | 34.207 | 0.656 | 0.759 | 3.505 | 2.656 | 0.550 |
| 319 | 0.0001 | 9 | 0.55 | **17.968** | 34.207 | 0.656 | 0.759 | 3.505 | 2.656 | 0.550 |
| 320 | 0.0001 | 10 | 0.55 | **17.968** | 34.207 | 0.656 | 0.759 | 3.505 | 2.656 | 0.550 |
| 321 | 0.0001 | 1 | 0.65 | **18.178** | 33.438 | 0.656 | 0.754 | 3.534 | 2.717 | 0.543 |
| 322 | 0.0001 | 2 | 0.75 | **18.265** | 33.121 | 0.652 | 0.804 | 3.216 | 2.395 | 0.621 |
| 323 | 0.0001 | 3 | 0.75 | **18.265** | 33.121 | 0.652 | 0.809 | 3.180 | 2.362 | 0.630 |
| 324 | 0.0001 | 4 | 0.75 | **18.265** | 33.121 | 0.652 | 0.809 | 3.180 | 2.362 | 0.630 |
| 325 | 0.0001 | 5 | 0.75 | **18.265** | 33.121 | 0.652 | 0.809 | 3.180 | 2.362 | 0.630 |
| 326 | 0.0001 | 6 | 0.75 | **18.265** | 33.121 | 0.652 | 0.809 | 3.180 | 2.362 | 0.630 |
| 327 | 0.0001 | 7 | 0.75 | **18.265** | 33.121 | 0.652 | 0.809 | 3.180 | 2.362 | 0.630 |
| 328 | 0.0001 | 8 | 0.75 | **18.265** | 33.121 | 0.652 | 0.809 | 3.180 | 2.362 | 0.630 |
| 329 | 0.0001 | 9 | 0.75 | **18.265** | 33.121 | 0.652 | 0.809 | 3.180 | 2.362 | 0.630 |
| 330 | 0.0001 | 10 | 0.75 | **18.265** | 33.121 | 0.652 | 0.809 | 3.180 | 2.362 | 0.630 |
| 331 | 0.0001 | 1 | 0.70 | **18.338** | 32.854 | 0.651 | 0.755 | 3.531 | 2.723 | 0.543 |
| 332 | 0.0001 | 1 | 0.75 | **18.627** | 31.795 | 0.644 | 0.754 | 3.538 | 2.732 | 0.542 |
| 333 | 0.05 | 1 | 0.50 | **NA** | NA | NA | NA | NA | NA | NA |
| 334 | 0.05 | 2 | 0.50 | **NA** | NA | NA | NA | NA | NA | NA |
| 335 | 0.05 | 3 | 0.50 | **NA** | NA | NA | NA | NA | NA | NA |
| 336 | 0.05 | 4 | 0.50 | **NA** | NA | NA | NA | NA | NA | NA |
| 337 | 0.05 | 5 | 0.50 | **NA** | NA | NA | NA | NA | NA | NA |
| 338 | 0.05 | 6 | 0.50 | **NA** | NA | NA | NA | NA | NA | NA |
| 339 | 0.05 | 7 | 0.50 | **NA** | NA | NA | NA | NA | NA | NA |
| 340 | 0.05 | 8 | 0.50 | **NA** | NA | NA | NA | NA | NA | NA |
| 341 | 0.05 | 9 | 0.50 | **NA** | NA | NA | NA | NA | NA | NA |
| 342 | 0.05 | 10 | 0.50 | **NA** | NA | NA | NA | NA | NA | NA |
| 343 | 0.05 | 2 | 0.70 | **NA** | NA | NA | NA | NA | NA | NA |
| 344 | 0.05 | 3 | 0.70 | **NA** | NA | NA | NA | NA | NA | NA |
| 345 | 0.05 | 4 | 0.70 | **NA** | NA | NA | NA | NA | NA | NA |
| 346 | 0.05 | 5 | 0.70 | **NA** | NA | NA | NA | NA | NA | NA |
| 347 | 0.05 | 6 | 0.70 | **NA** | NA | NA | NA | NA | NA | NA |
| 348 | 0.05 | 7 | 0.70 | **NA** | NA | NA | NA | NA | NA | NA |
| 349 | 0.05 | 8 | 0.70 | **NA** | NA | NA | NA | NA | NA | NA |
| 350 | 0.05 | 9 | 0.70 | **NA** | NA | NA | NA | NA | NA | NA |
| 351 | 0.05 | 10 | 0.70 | **NA** | NA | NA | NA | NA | NA | NA |
| 352 | 0.05 | 2 | 0.75 | **NA** | NA | NA | NA | NA | NA | NA |
| 353 | 0.05 | 3 | 0.75 | **NA** | NA | NA | NA | NA | NA | NA |
| 354 | 0.05 | 4 | 0.75 | **NA** | NA | NA | NA | NA | NA | NA |
| 355 | 0.05 | 5 | 0.75 | **NA** | NA | NA | NA | NA | NA | NA |
| 356 | 0.05 | 6 | 0.75 | **NA** | NA | NA | NA | NA | NA | NA |
| 357 | 0.05 | 7 | 0.75 | **NA** | NA | NA | NA | NA | NA | NA |
| 358 | 0.05 | 8 | 0.75 | **NA** | NA | NA | NA | NA | NA | NA |
| 359 | 0.05 | 9 | 0.75 | **NA** | NA | NA | NA | NA | NA | NA |
| 360 | 0.05 | 10 | 0.75 | **NA** | NA | NA | NA | NA | NA | NA |

**Table S11.** Results of linear regression models showing the correlations between each of the items and spatial scales.

| Item | Estimate | Standard error | t value | *p* |
| --- | --- | --- | --- | --- |
| *R*^2^ | 0.121 | 0.042 | 2.885 | **0.044*** |
| MDE | -0.043 | 0.014 | -3.021 | **0.039*** |
| Bio14 | -0.017 | 0.006 | -2.775 | **0.050*** |
| HD | 0.006 | 0.010 | 0.648 | 0.552 |
| NPP | 0.035 | 0.011 | 3.055 | **0.037*** |
| Bio1 | 0.006 | 0.008 | 0.769 | 0.484 |
| NDVI | 0.013 | 0.004 | 2.942 | **0.042*** |
| Bio12 | -0.005 | 0.007 | -0.732 | 0.505 |
| Bio7 | 0.003 | 0.001 | 2.815 | **0.048*** |
| Bio6 | 0.000 | 0.000 | -1.846 | 0.139 |
| WS | 0.000 | 0.000 | NA | NA |
| * *p* < 0.05. | | | | |

**Table S12.** Spatial autocorrelation test in residuals of elevation-only GAMs using Moran’s *I* test with Monte Carlo simulation (999 permutations). For groups with significant spatial autocorrelation (*p* < 0.05), explained variance (*R*^2^) were compared between elevation only GAM (Model 1), spatial coordinates only GAM (Model 2), and elevation plus spatial coordinates GAM (Model 3) to clarify whether spatial autocorrelation was an independent effect or a byproduct of elevation.

| Group | Spatial autocorrelation | | |  | Explained variance (*R*^2^) | | | | |
| --- | --- | --- | --- | --- | --- | --- | --- | --- | --- |
|  | Moran’s *I* |  | *p* |  | Model 1 |  | Model 2 |  | Model 3 |
| *c*-value | -0.007 |  | 0.421 |  | - |  | - |  | - |
| *z*-value | -0.138 |  | 0.975 |  | - |  | - |  | - |
| Species richness at 0.0001 m^2^ spatial scale | -0.067 |  | 0.728 |  | - |  | - |  | - |
| Species richness at 0.01 m^2^ spatial scale | -0.125 |  | 0.953 |  | - |  | - |  | - |
| Species richness at 0.25 m^2^ spatial scale | -0.025 |  | 0.509 |  | - |  | - |  | - |
| Species richness at 1 m^2^ spatial scale | 0.099 |  | 0.065 |  | - |  | - |  | - |
| Species richness at 5 m^2^ spatial scale | 0.128 |  | **0.027*** |  | 0.292 |  | 0.418 |  | 0.396 |
| Species richness at 10 m^2^ spatial scale | 0.080 |  | 0.089 |  | - |  | - |  | - |
| * *p* < 0.05. | | | | | | | | | |

**
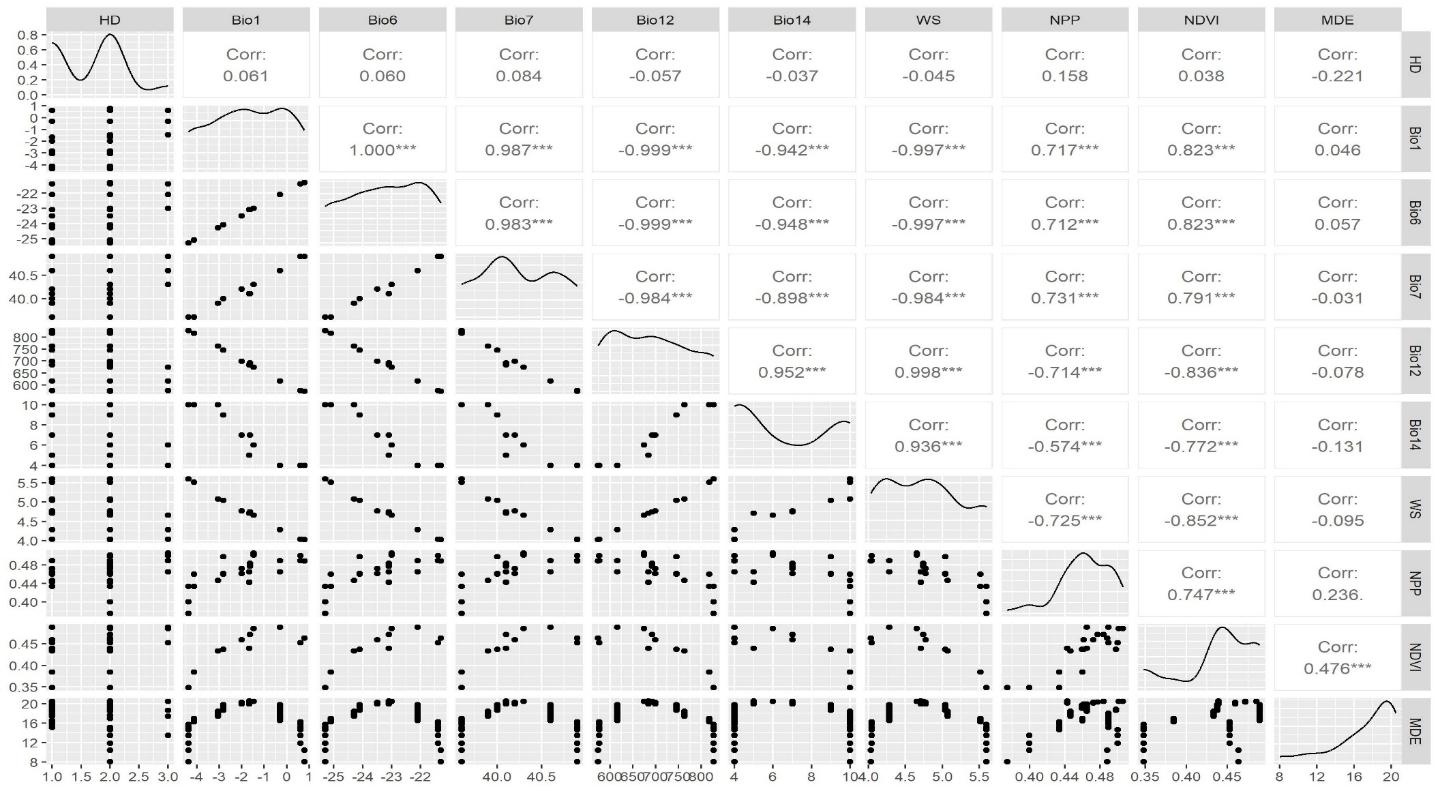
Figure S1.** Scatterplots of each variable for species richness at 100 m^2^ spatial scale.

**Figure S2.** Comparison of *c*- and *z*-values across four main environments. The crossbar within the boxplot shows the median, the length of the box reflects interquartile range, and the thin black lines represent the whiskers. Jittered *c*-value (a) and *z*-value (b) are shown within violin plot. Asterisks indicate statistically significant differences (****p* < 0.001) using pairwise t-test. F, G, M and R represent the four environments: forest, grassland, alpine meadow, and alpine rocky meadow, respectively.

| (a) | 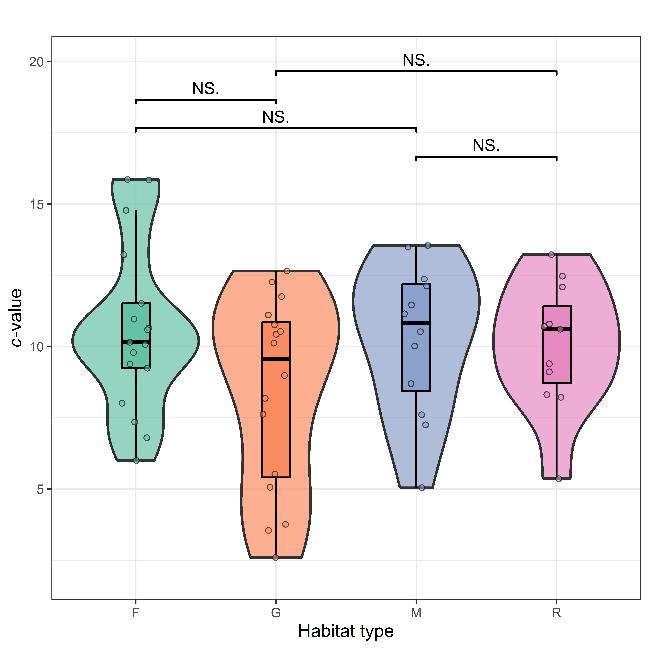 | (b) | 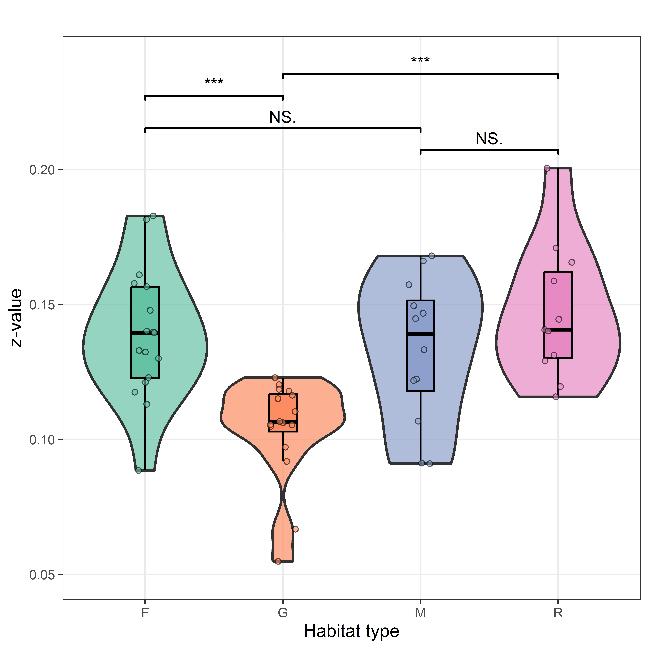 |
| --- | --- | --- | --- |

**Figure S3.** Relationship between the MDE and species richness under each spatial scale.

| **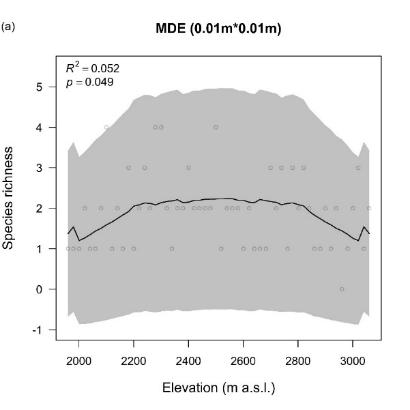** | **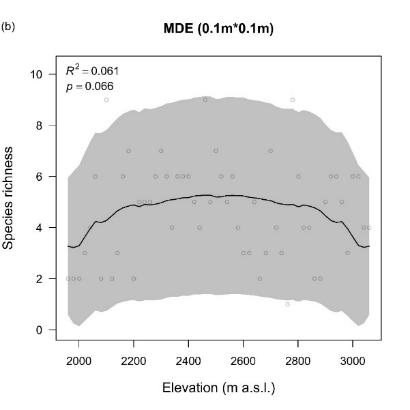** | **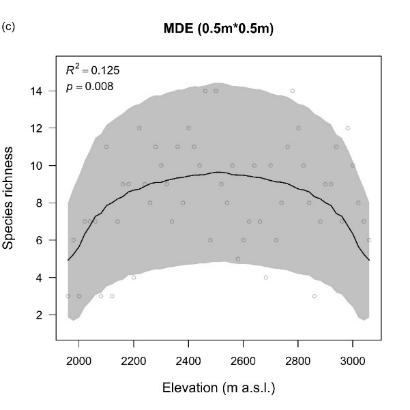** |
| --- | --- | --- |
| **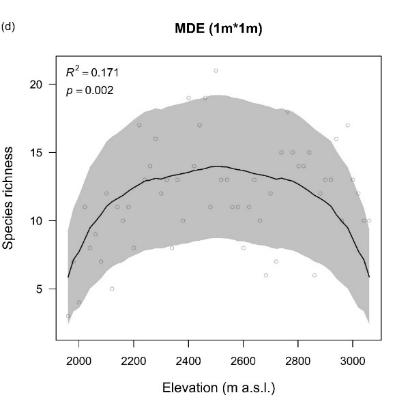** | **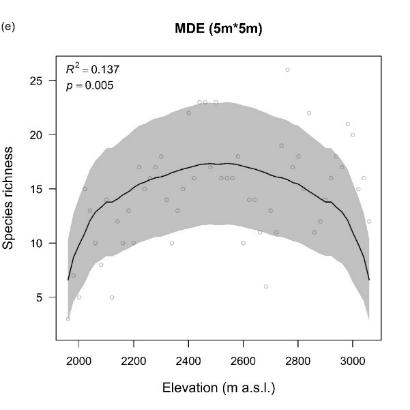** | **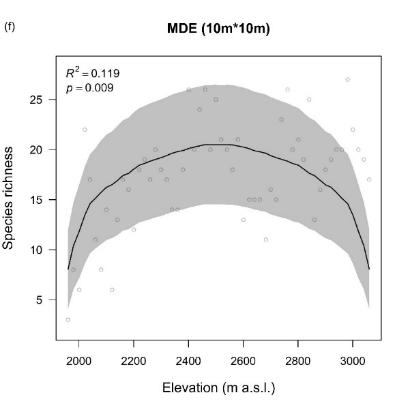** |

**Figure S4.** The important interactive effects between variables in driving *c*-value.

| **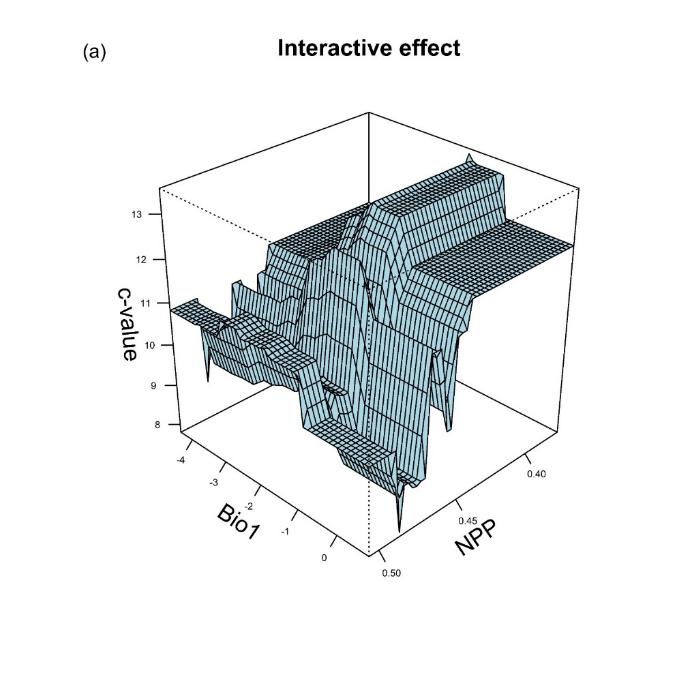** | **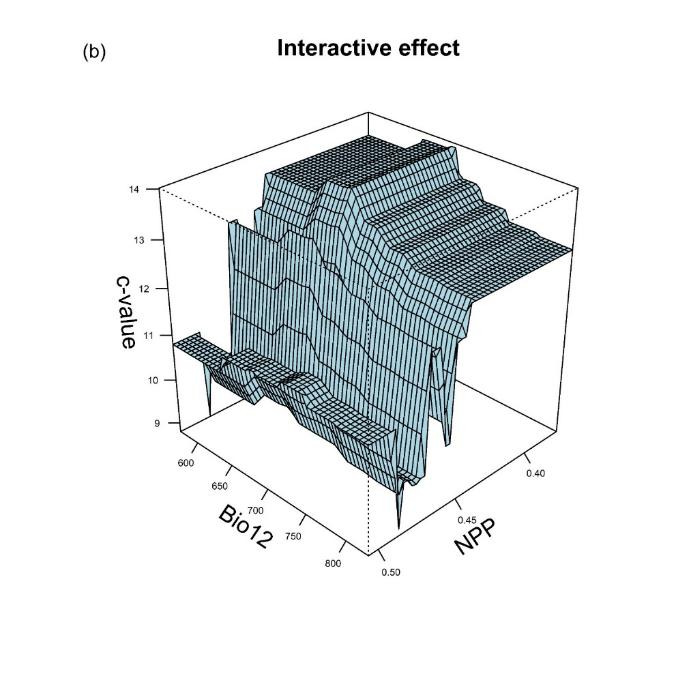** |
| --- | --- |
| **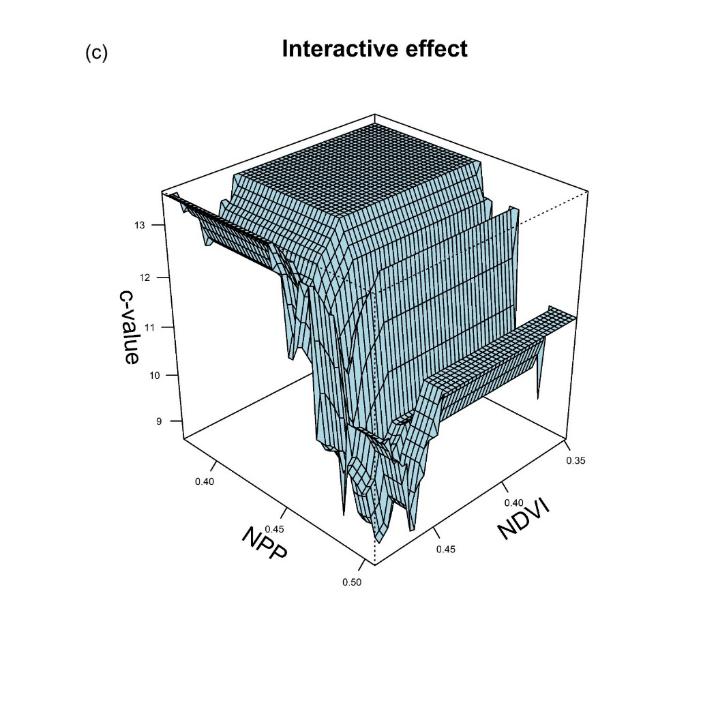** | **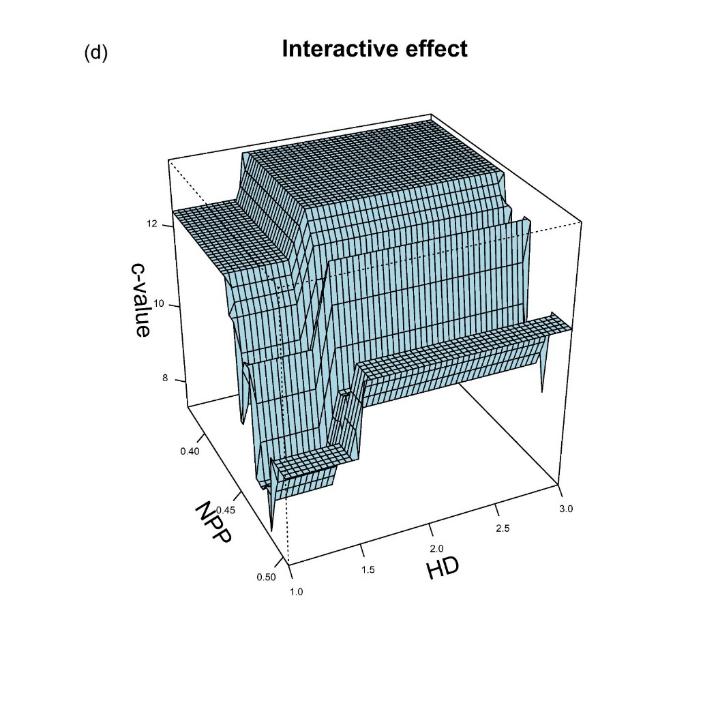** |

**Figure S5.** The important interactive effects between variables in driving species richness at 0.0001 m^2^ spatial scale.

| **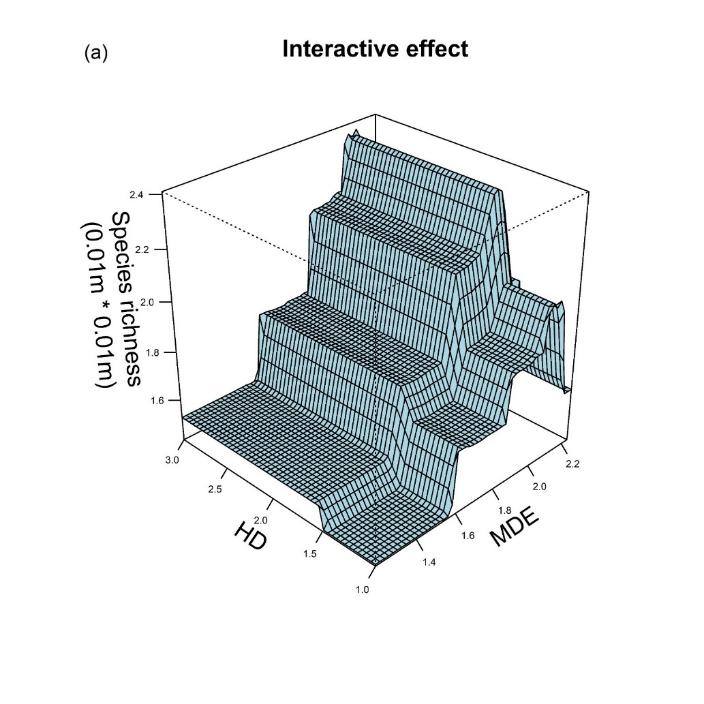** | **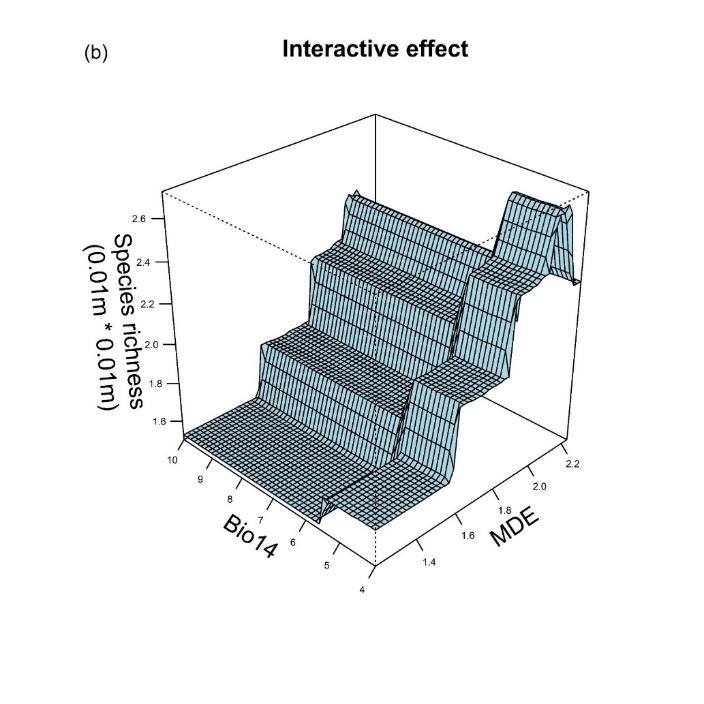** |
| --- | --- |
| **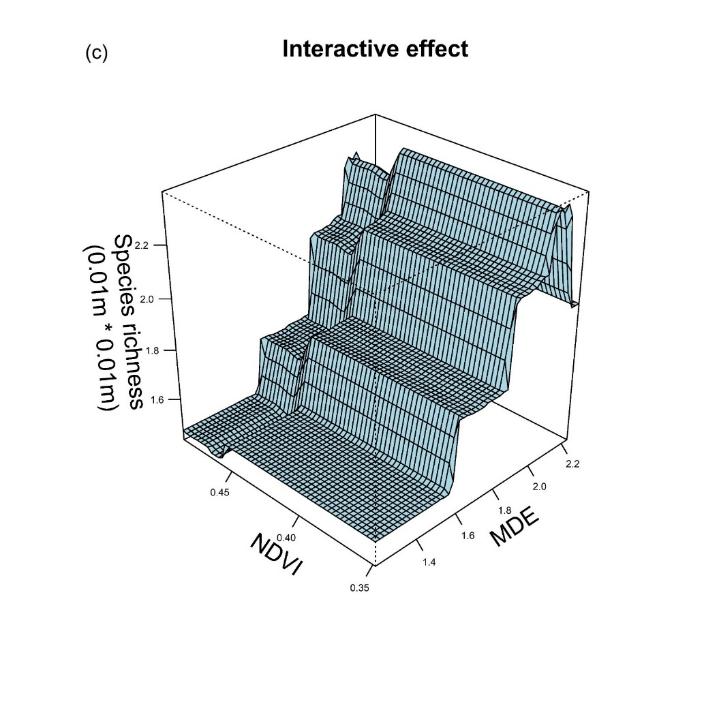** | **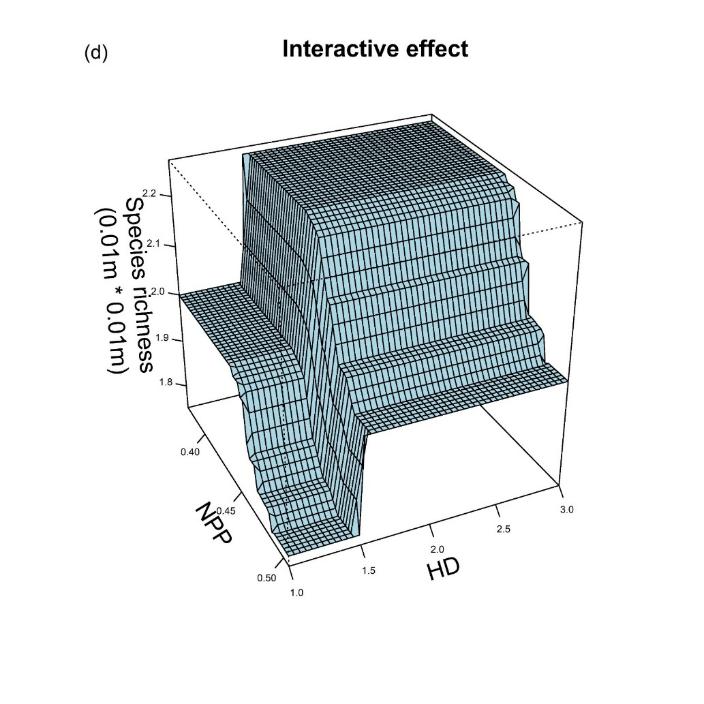** |

**Figure S6.** The important interactive effects between variables in driving species richness at 0.01 m^2^ spatial scale.

| **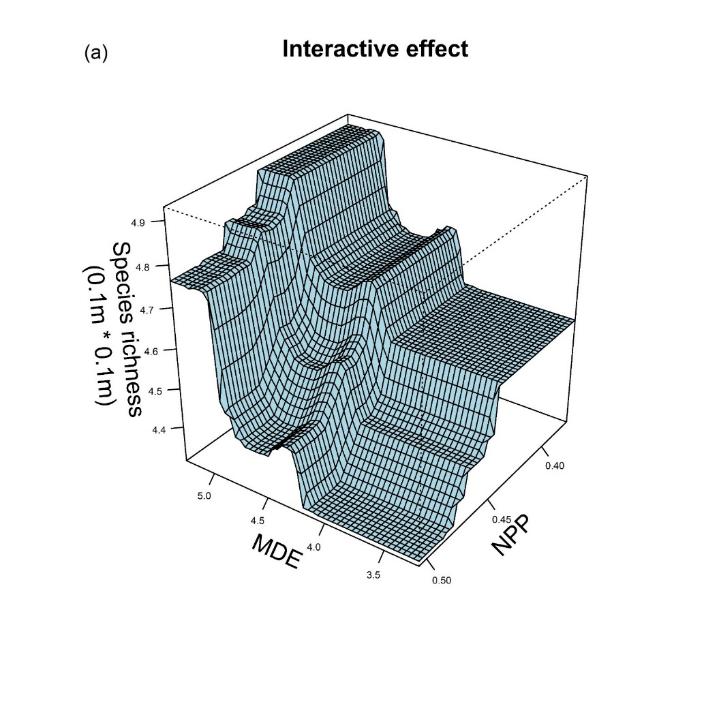** | **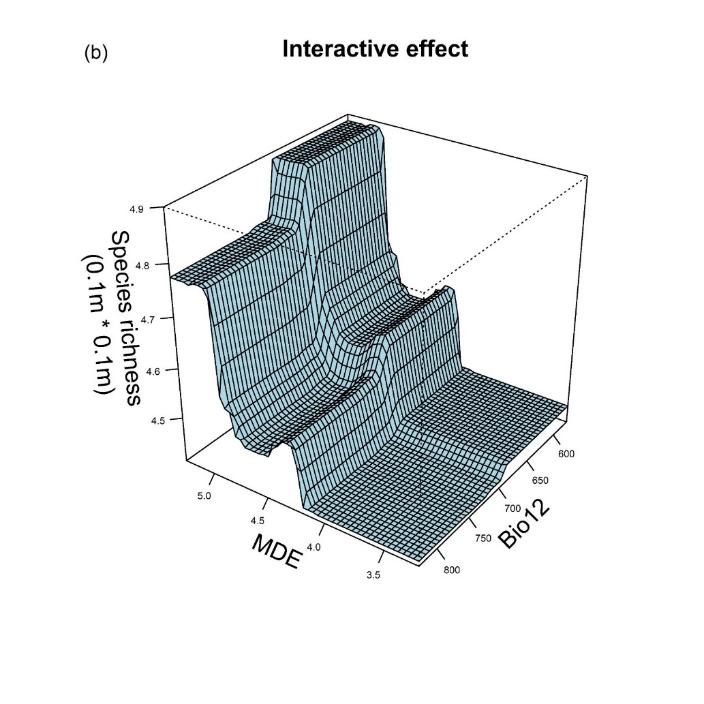** |
| --- | --- |
| **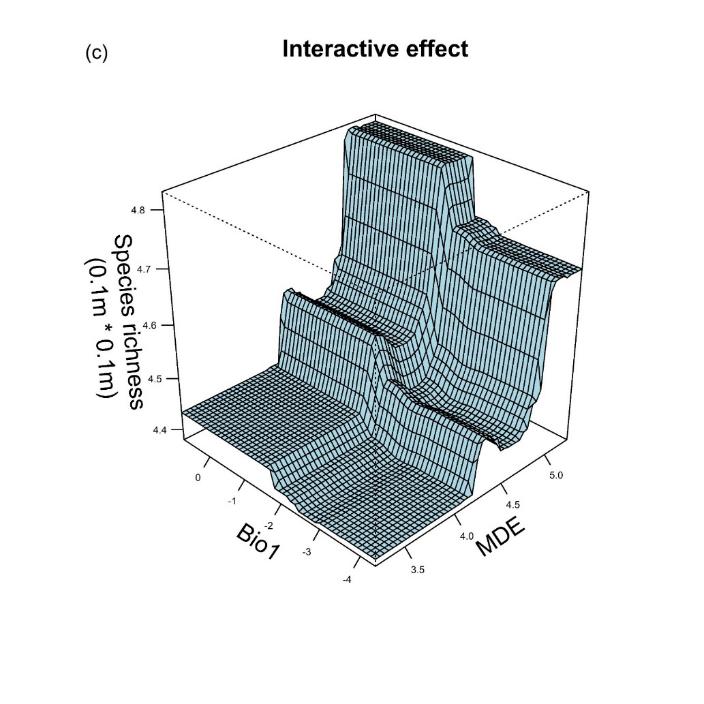** | **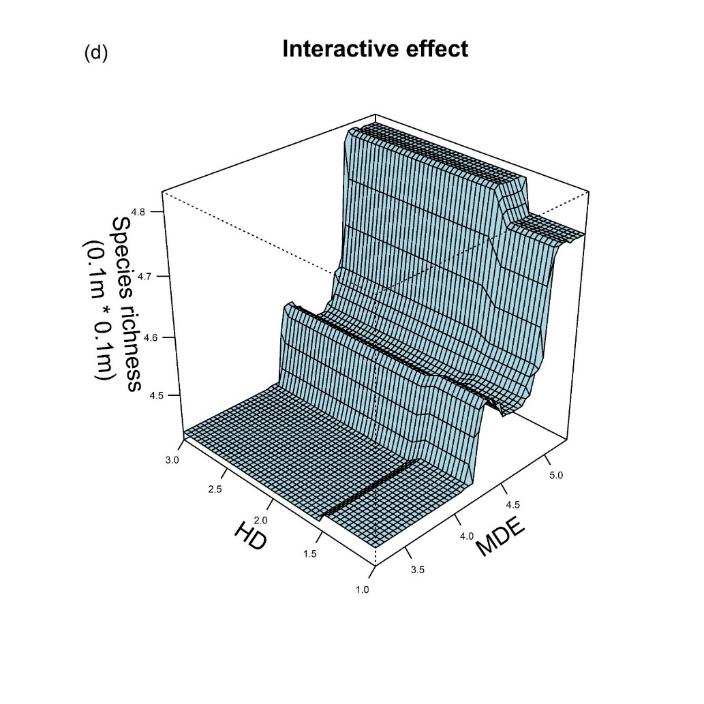** |

**Figure S7.** The important interactive effects between variables in driving species richness at 25 m^2^ spatial scale.

| **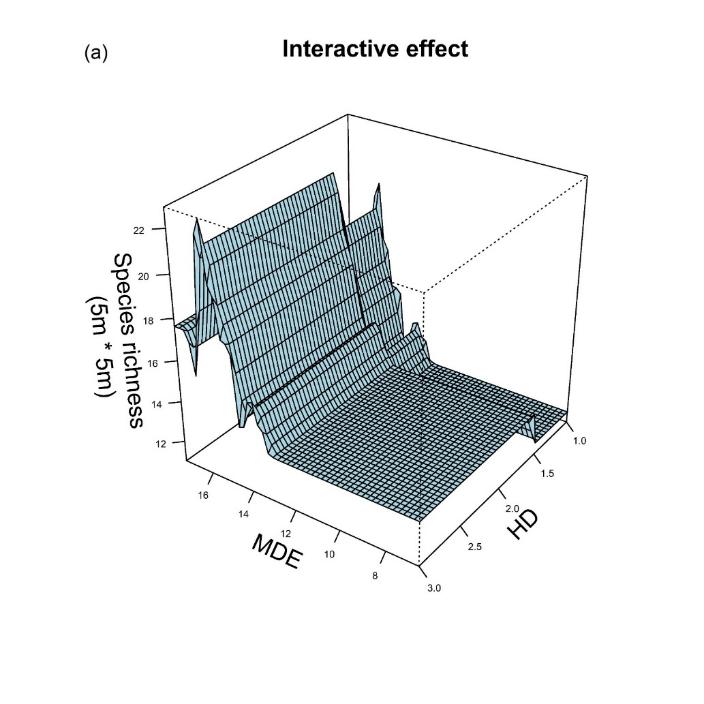** | **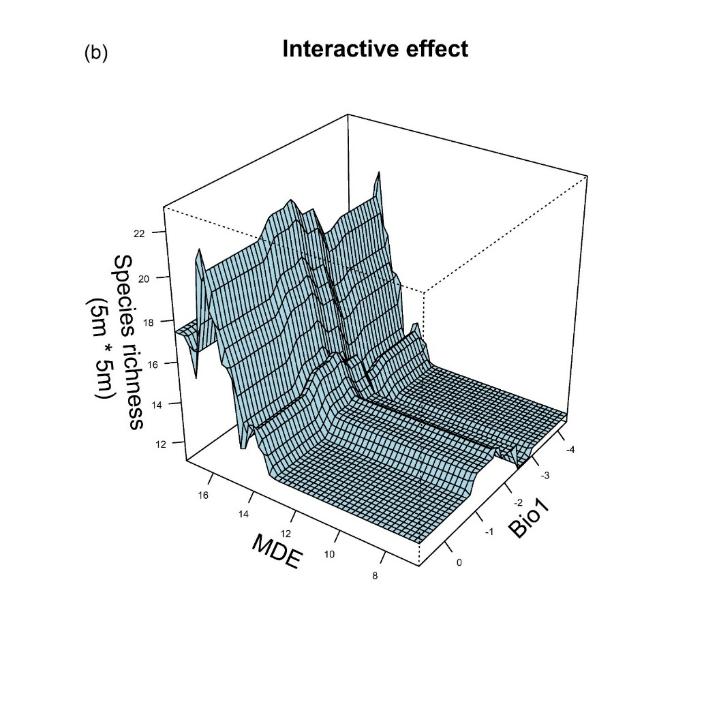** |
| --- | --- |
| **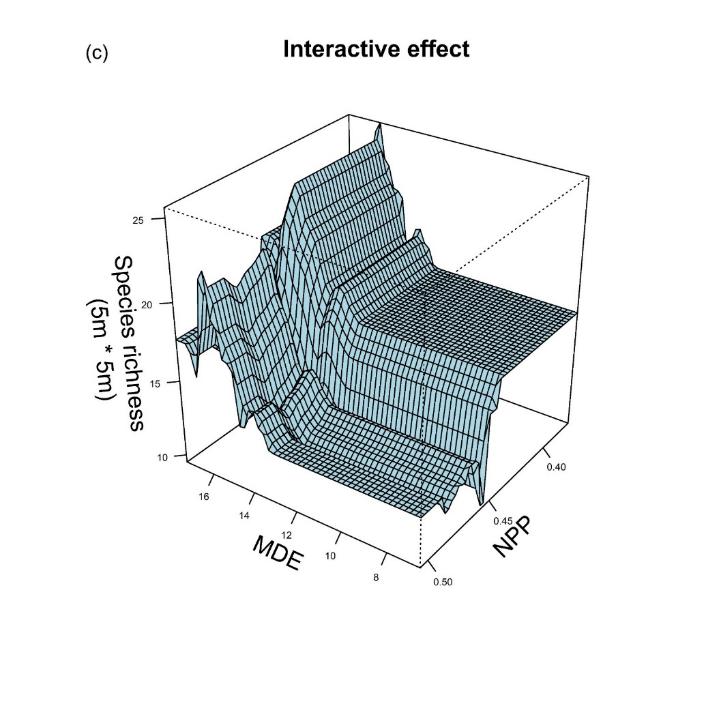** | **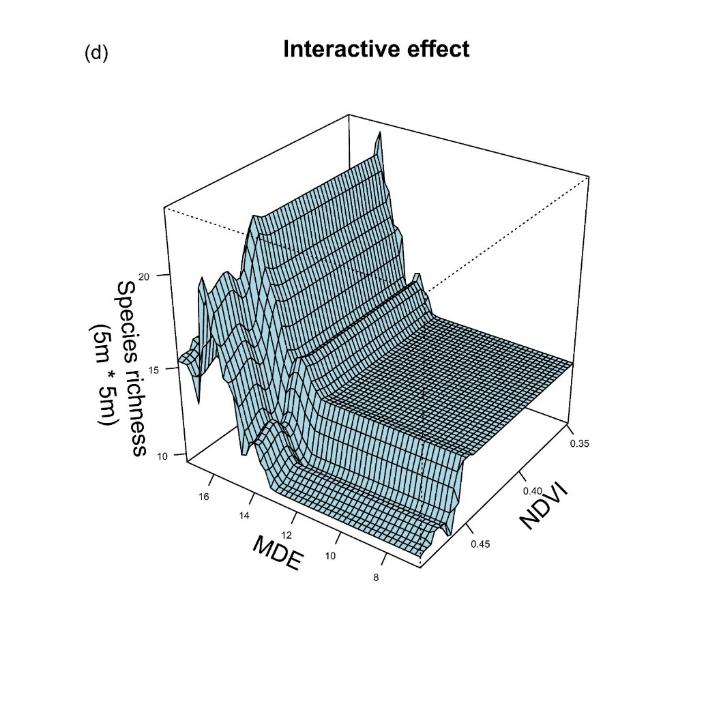** |

**Figure S8.** Relative influence and partial dependence plots of each individual variable on moss species richness variation under each spatial scale: (a–b) 0.0001 m^2^; (c–d) 0.01 m^2^; (e–f) 0.25 m^2^; (g–h) 1 m^2^; (i–j) 25 m^2^; and (k–l) 100 m^2^.

| (a) | 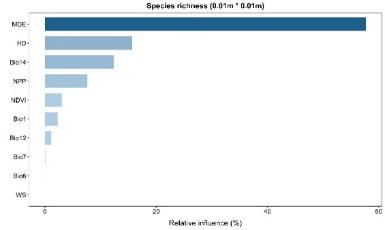 | | | | |
| --- | --- | --- | --- | --- | --- |
| (b) | 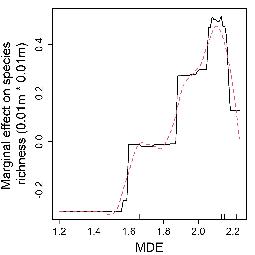 | 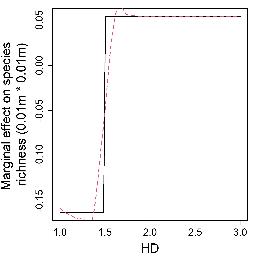 | 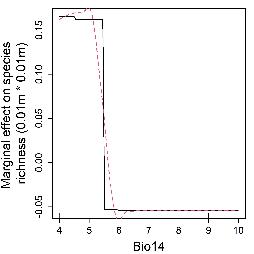 | 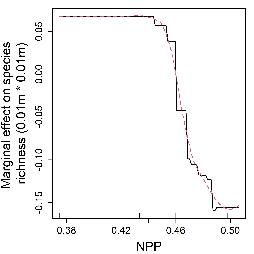 | 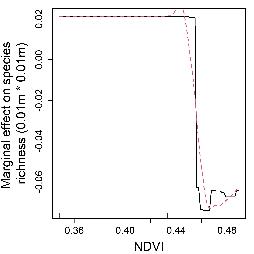 |
|  | 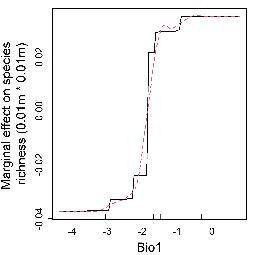 | 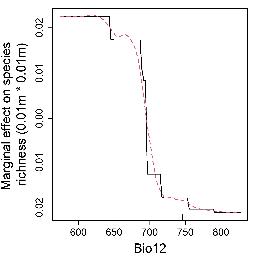 | 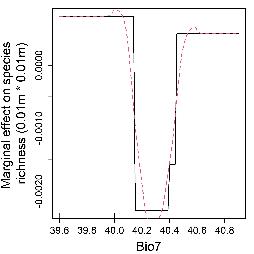 | 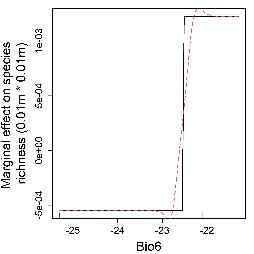 | 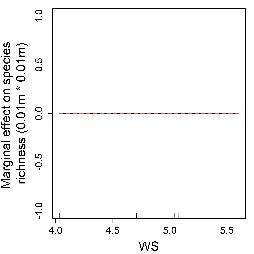 |
| (c) |  | | | | |
| (d) | 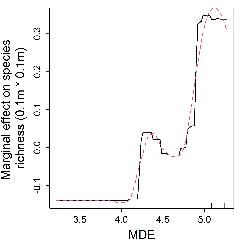 | 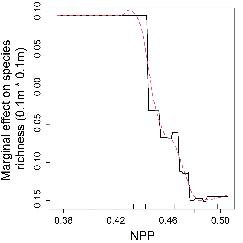 | 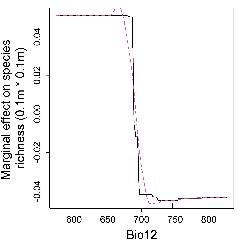 | 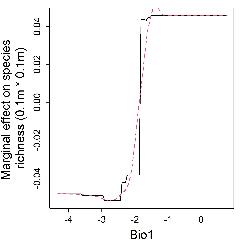 | 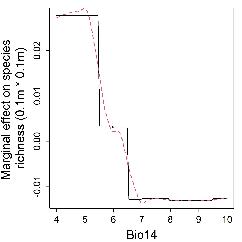 |
|  | 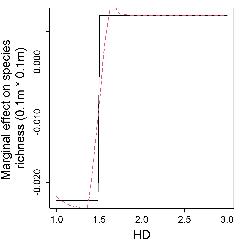 | 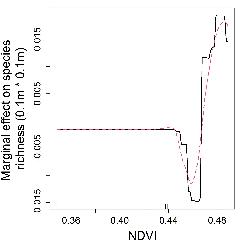 | 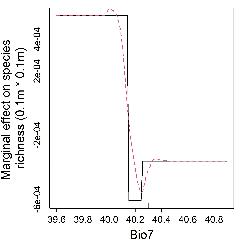 | 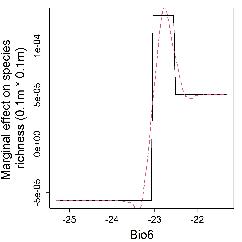 | 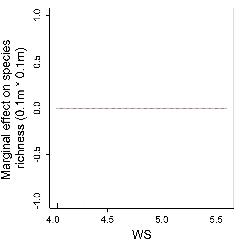 |
| (e) |  | | | | |
| (f) | 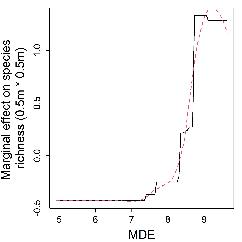 | 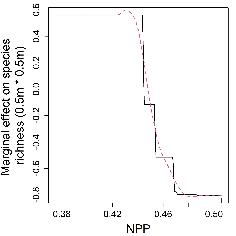 | 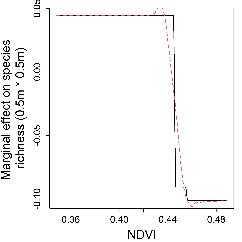 | 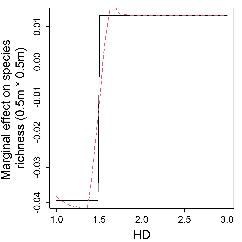 | 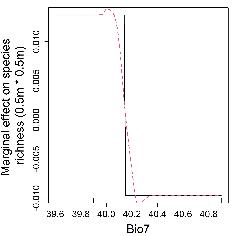 |
|  | 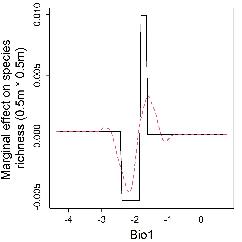 | 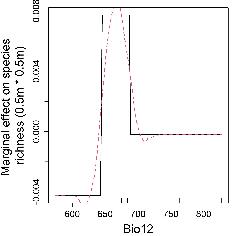 | 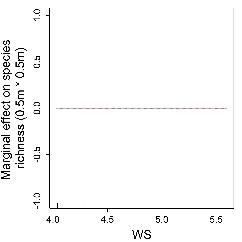 | 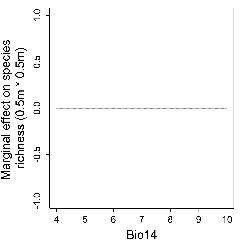 | 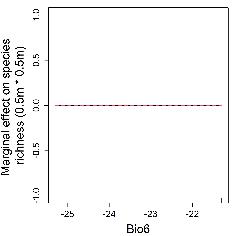 |
| (g) |  | | | | |
| (h) | 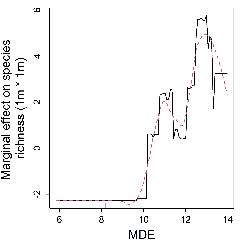 | 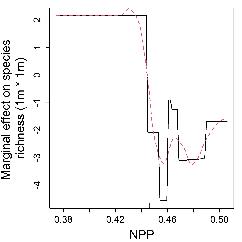 | 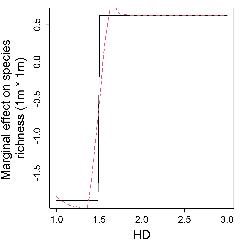 | 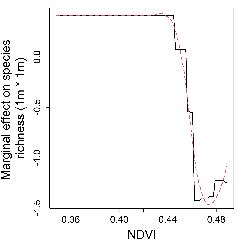 | 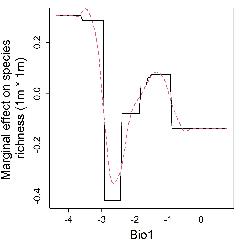 |
|  | 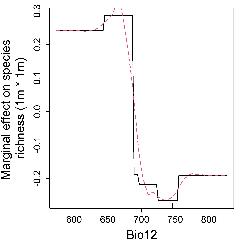 | 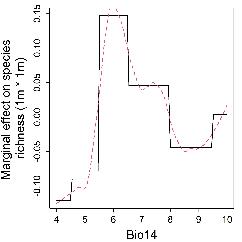 | 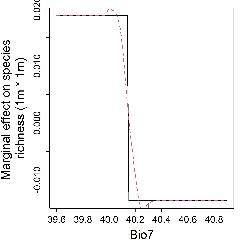 | 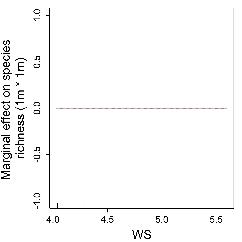 | 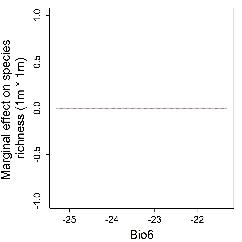 |
| (i) |  | | | | |
| (j) | 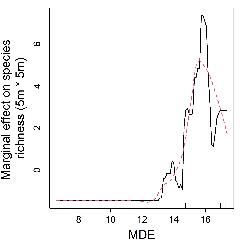 | 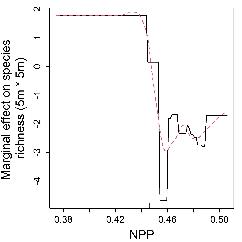 | 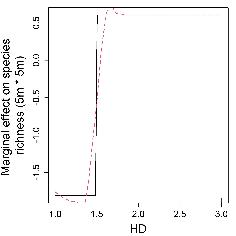 | 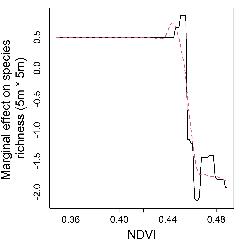 | 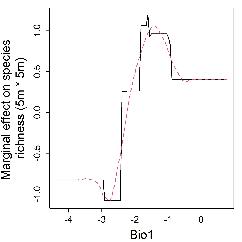 |
|  | 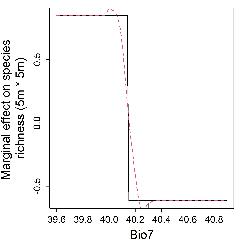 | 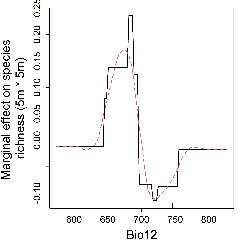 | 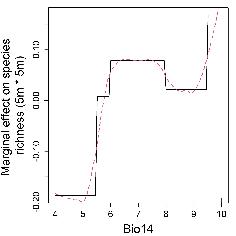 | 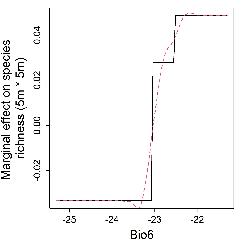 | 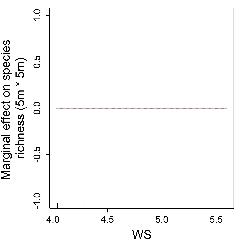 |
| (k) |  | | | | |
| (l) | 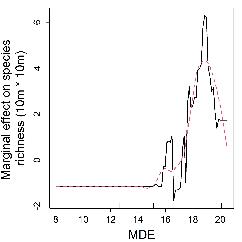 | 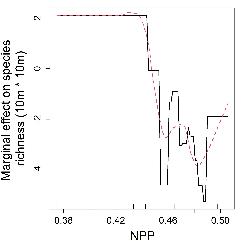 | 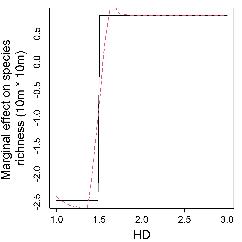 | 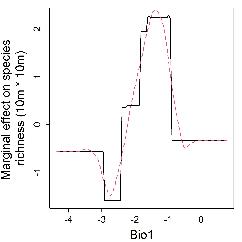 | 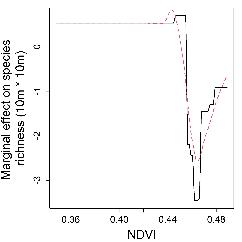 |
|  | 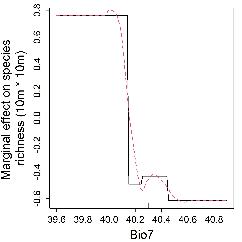 | 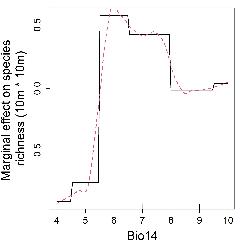 | 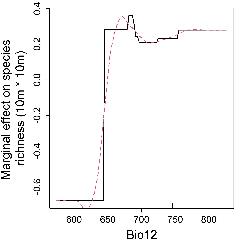 | 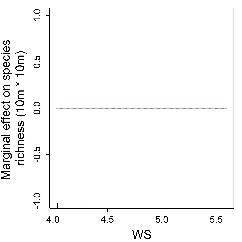 | 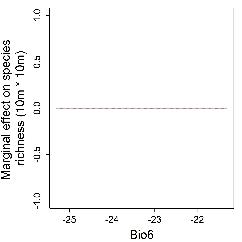 |

**Figure S9.** Sensitivity analysis due to the spatial scale mismatch between biological observations and environmental predictors. Both drop-one-variable sensitivity analysis (left column) and perturbation sensitivity analysis (right column) were performed for *c*-value, *z*-value, and species richness at each spatial scale. The red dashed line represents the *R*^2^ of the baseline model.

| (a) | 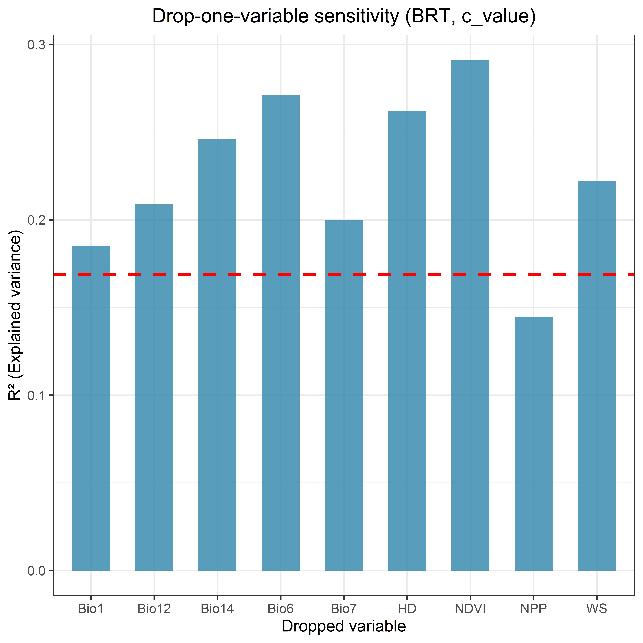 | (b) | 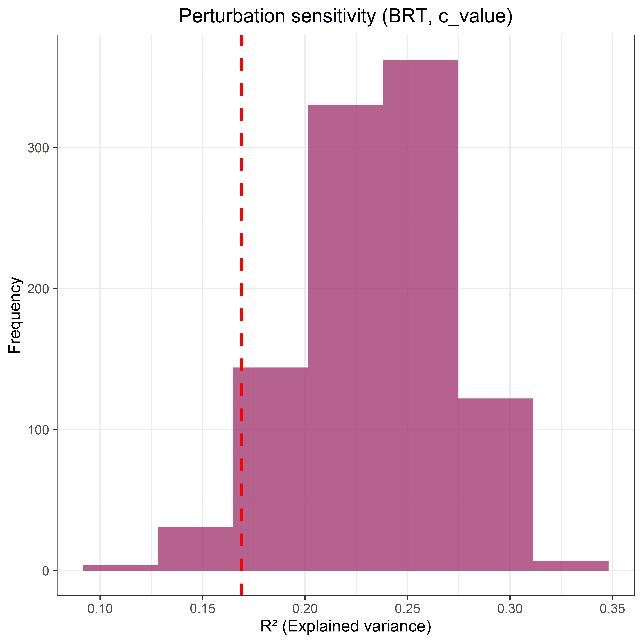 |
| --- | --- | --- | --- |
| (c) | 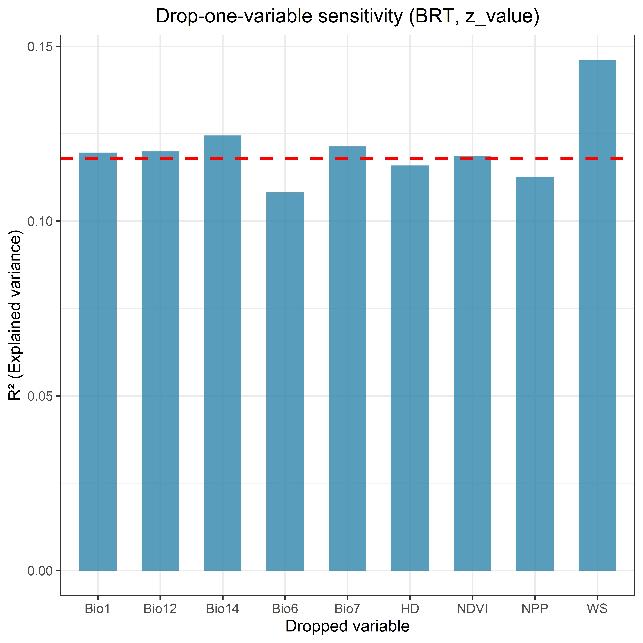 | (d) | 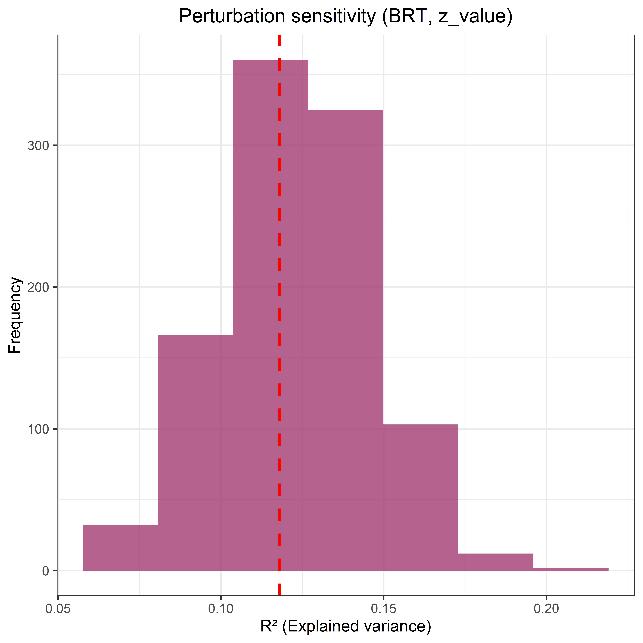 |
| (e) | 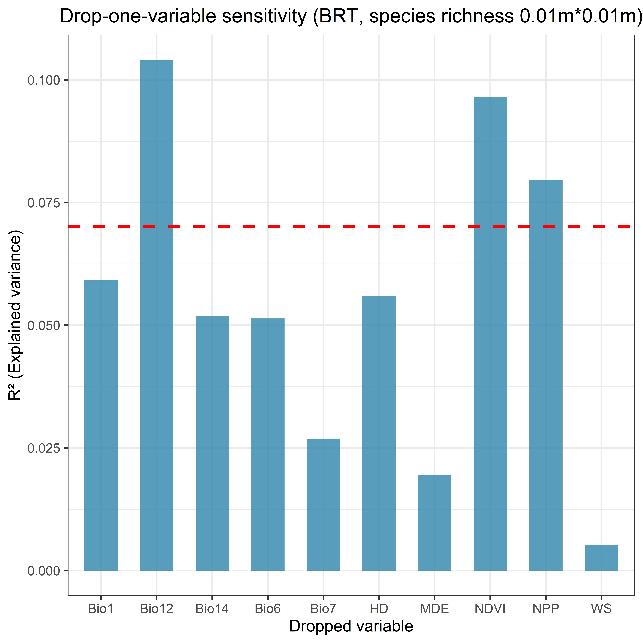 | (f) | 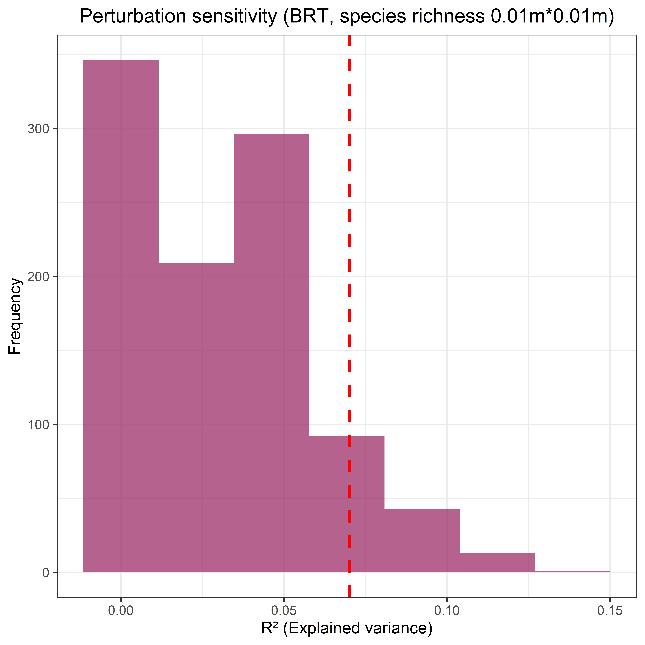 |
| (g) | 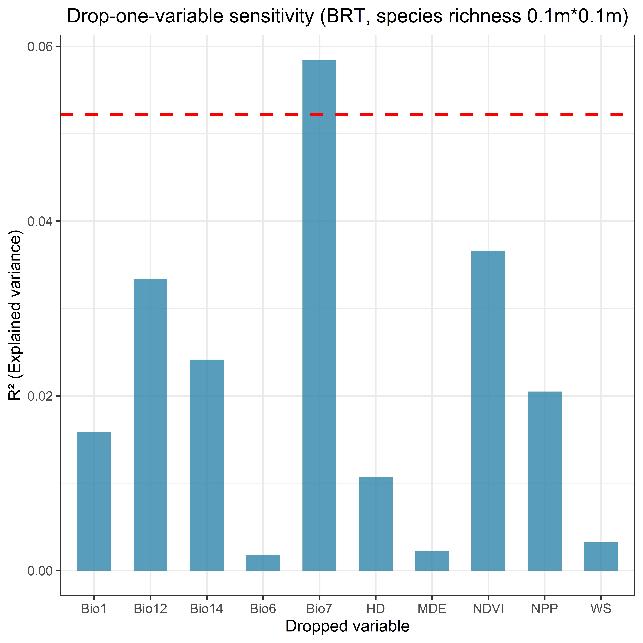 | (h) | 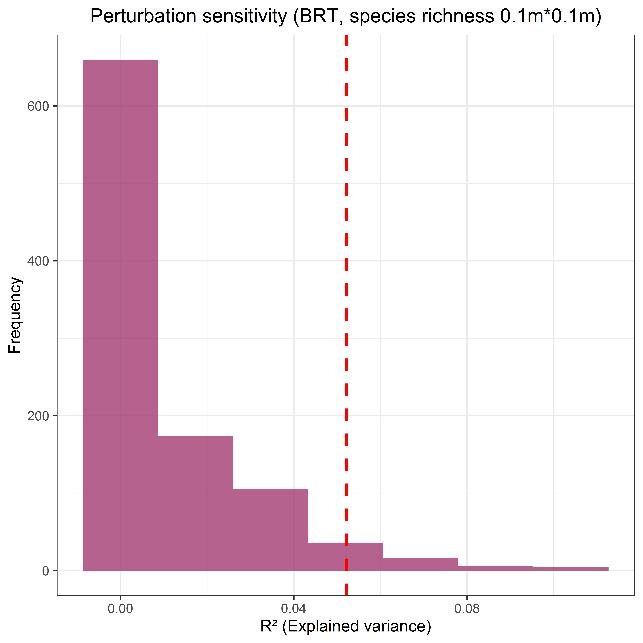 |
| (i) | 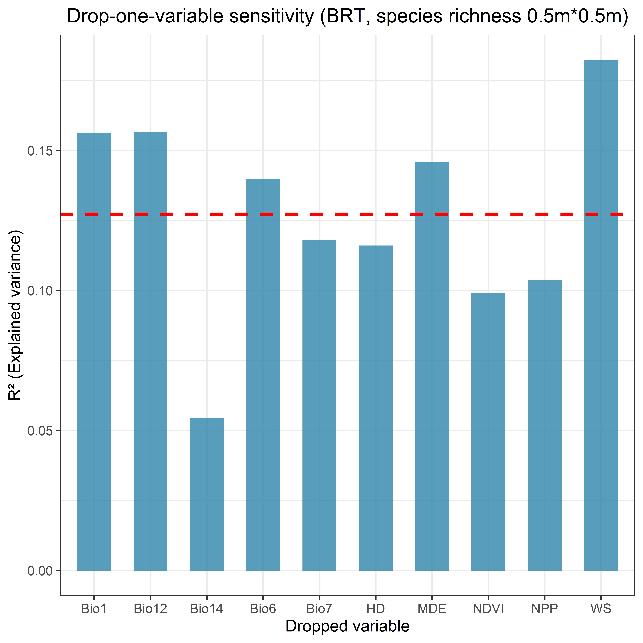 | (j) |  |
| (k) |  | (l) |  |
| (m) |  | (n) |  |
| (o) |  | (p) |  |

**Figure S10.** Spatial distribution of standardized BRT residuals with Moran’s *I* statistic and *p*-value displayed for *c*-values, *z*-values, and species richness at each spatial scale.

| (a) |  |
| --- | --- |
| (b) |  |
| (c) |  |
| (d) |  |
| (e) |  |
| (f) |  |
| (g) |  |
| (h) |  |
