# Supplementary material for: Unraveling the Influence of Elevation on Moss Species–Area Relationships and the Effect of Spatial Scale on Elevational Richness Patterns in Mt Wutai With a Nested‐Plot Sampling Design
Source: Ecol Evol. 2026 Apr 10;16(4):e73473. doi: 10.1002/ece3.73473 (PMC13068500; doi:10.1002/ece3.73473)
Supplement: Supplementary file 2 — Appendix S2: The detailed pseudo R code utilized for the statistical analyses in this study. [file ECE3-16-e73473-s003.docx]

**Unravelling the influence of elevation on moss species–area relationships and the effect of spatial scale on elevational richness patterns in Mt Wutai with a nested-plot sampling design**

Haozhe Wang, Fenghua Wang, Yu Zhao, Chenglong Li, Xiaowei Ma, Xiaopan Wang, Lina Zhang, De Gao*

**SUPPORTING INFORMATION**

**Appendix S2** The detailed pseudo R code utilized for the statistical analyses in this study, including (1) species richness rarefaction; (2) SAR fitting; (3) GAMs; (4) BRTs; (5) linear regressions of *R*^2^ and relative influence (RI) against scale; (6) comparison of *c*- and *z*-values across main environments; and (7) sensitivity analyses due to the spatial scale mismatch between biological observations and environmental predictors.

**## Analysis 1. Species richness rarefaction**

## Six species-plot occurrence/absence datasets were prepared for each spatial scale.

## Below is the r code used to generate Figure 1b of our manuscript.

library(vegan)

Spp0.01 <- read.csv(file.choose())

Spp0.1 <- read.csv(file.choose())

Spp0.5 <- read.csv(file.choose())

Spp1 <- read.csv(file.choose())

Spp5 <- read.csv(file.choose())

Spp10 <- read.csv(file.choose())

sp1 <- specaccum(Spp0.01, method = "random", permutations = 1000)

sp2 <- specaccum(Spp0.1, method = "random", permutations = 1000)

sp3 <- specaccum(Spp0.5, method = "random", permutations = 1000)

sp4 <- specaccum(Spp1, method = "random", permutations = 1000)

sp5 <- specaccum(Spp5, method = "random", permutations = 1000)

sp6 <- specaccum(Spp10, method = "random", permutations = 1000)

ppi<-1200

jpeg("Species richness rarefaction.jpg",width=6*ppi,height=6*ppi,res=ppi)

par(mfrow = c(1, 1), mar = c(4.2, 4.2, 1, 1))

plot(sp6, ci.type="poly", col="#1E88E5", lwd=1.5, ci.lty=0,

ci.col=rgb(30, 136, 229, 51, maxColorValue = 255),

xlab="Sampling plots", ylab="Species richness", cex.lab = 1.4, main="")

lines(sp5, ci.type="poly", col="#42A5F5", lwd=1.5, ci.lty=0,

ci.col=rgb(66, 165, 245, 51, maxColorValue = 255))

lines(sp4, ci.type="poly", col="#64B5F6", lwd=1.5, ci.lty=0,

ci.col=rgb(100, 181, 246, 51, maxColorValue = 255))

lines(sp3, ci.type="poly", col="#90CAF9", lwd=1.5, ci.lty=0,

ci.col=rgb(144, 202, 249, 51, maxColorValue = 255))

lines(sp2, ci.type="poly", col="#BBDEFB", lwd=1.5, ci.lty=0,

ci.col=rgb(187, 222, 251, 51, maxColorValue = 255))

lines(sp1, ci.type="poly", col="#E3F2FD", lwd=1.5, ci.lty=0,

ci.col=rgb(227, 242, 253, 51, maxColorValue = 255))

legend(37, 26, legend=c("10 m * 10 m", "5 m * 5 m","1 m * 1 m",

"0.5 m * 0.5 m","0.1 m * 0.1 m", "0.01 m * 0.01 m"),

col=c("#1E88E5", "#42A5F5", "#64B5F6", "#90CAF9", "#BBDEFB", "#E3F2FD"),

text.font=1, bty="n", lty=1:1, cex=0.9,title = "Spatial scale")

dev.off()

**## Analysis 2. SAR fitting**

## moss1 is a data table consisting of 7 columns, with the first column displaying plot numbers

## and the second to seventh columns displaying species richness at scales of 100 m^2^, 25 m^2^, 1 m^2^,

## 0.25 m^2^, 0.01 m^2^, and 0.0001 m^2^, respectively.

## Given the uncertainty associated with the *c*- and *z*-values derived from the SAR models, the

## standard errors (SEs) for *c* and *z* were calculated and subsequently incorporated into the

## downstream models.

library(sars)

moss1 <- read.csv(file.choose())

for(i in 1:56){

a <- c(100,25,1,0.25,0.01,0.0001)

s <- c(moss1[i,2],moss1[i,3],moss1[i,4],moss1[i,5],moss1[i,6],moss1[i,7])

sadata <- data.frame(a,s)

sar_model <- sar_power(data = sadata)

c_value[i] <- summary(sar_model)$Parameters[1]

z_value[i] <- summary(sar_model)$Parameters[2]

## The sars package fitting model cannot directly obtain the variance-covariance matrix,

## so manual fitting is required.

nls_model <- nls(s ~ c * a^z,

data = sadata,

start = list(c = summary(sar_model)$Parameters[1],

z = summary(sar_model)$Parameters[2]),

control = nls.control(maxiter = 1000))

## Calculate variance-covariance matrix

vcov_nls <- vcov(nls_model)

## Calculate standard error (SE) for *c* and *z*

c_se[i] <- sqrt(diag(vcov_nls))[1]

z_se[i] <- sqrt(diag(vcov_nls))[2]

}

## CZResult summarized *c*, *z*, standard error of *c*, and standard error of *z* for 56 plots.

CZResult<-data.frame(c_value, z_value, c_se, z_se)

**## Analysis 3. Generalized additive models (GAMs)**

## moss2 is a dataset consisting of 13 columns, including: *c*, *z*, standard error of *c* (*c*_se),

## standard error of *z* (*z*_se), species richness at each of the six spatial scales, elevation,

## latitude and longitude, respectively.

## The weighting term “weights = 1/c_se” and “weights = 1/z_se” were implemented in the

## GAMs for *c*~elevation and *z*~elevation to mitigate the uncertainty associated with *c* and *z*. The ## larger the c_se or z_se value, the smaller the corresponding weight; the model thus dampens

## the influence of such *c* or *z* values, characterized by high estimation error and low reliability, ## on the fitting results. Whereas the weighting term were not implemented in the GAMs for

## species richness~elevation.

**## 3.1. Spatial autocorrelation test for model residuals**

## We first assessed spatial autocorrelation in the residuals of the original GAM models (only

## elevation as predictor) using Moran’s *I* test with Monte Carlo simulation (999 permutations)

## based on k-nearest neighbor (k=5) spatial weight matrices (constructed from plot latitude and ## longitude). Taking *c*-value and species richness at 25 m^2^ as examples.

library(mgcv)

library(Matrix)

library(lme4)

library(MASS)

library(blmeco)

library(arm)

library(car)

library(Hmisc)

library(MuMIn)

library(logistf)

library(writexl)

library(plotrix)

moss2<-read.csv(file.choose())

## *c*-value

mod_gam_original <- gam(c~s(Elevation, bs="cr", k=5),

data= moss2,

weights = 1/c_se,

family = gaussian(),

method = "GCV.Cp")

resid_original <- residuals(mod_gam_original)

## Construct the spatial weight matrix using the k-nearest neighbor method (k = 5)

coords_mat <- as.matrix(moss2[, c("Longitude", "Latitude")])

nb <- knn2nb(knearneigh(coords_mat, k = 5))

nb <- make.sym.nb(nb)

lw <- nb2listw(nb, style = "W")

## Moran’s *I* test with Monte Carlo simulation

moran_mc <- moran.mc(resid_original, listw = lw, nsim = 999)

print(moran_mc)

## Species richness 25 m^2^

mod_gam_original <- gam(SpeciesRichness5~s(Elevation, bs="cr", k=5),

data= moss2,

family = gaussian(),

method = "GCV.Cp")

resid_original <- residuals(mod_gam_original)

## Construct the spatial weight matrix using the k-nearest neighbor method (k = 5)

coords_mat <- as.matrix(moss2[, c("Longitude", "Latitude")])

nb <- knn2nb(knearneigh(coords_mat, k = 5))

nb <- make.sym.nb(nb)

lw <- nb2listw(nb, style = "W")

## Moran’s *I* test with Monte Carlo simulation

moran_mc <- moran.mc(resid_original, listw = lw, nsim = 999)

print(moran_mc)

**## 3.2. Distinguishing independent spatial effects from elevational effects**

## To clarify whether spatial autocorrelation for 25 m^2^ species richness was an independent effect ## or a byproduct of elevation, we fitted three alternative GAMs and compared their explained

## variance (*R*^2^): Model 1 (elevation only); Model 2 (spatial coordinates only); and Model 3

## (elevation + spatial coordinates).

## Model 1 (elevation only)

mod_gam_original <- gam(SpeciesRichness5~s(Elevation, bs="cr", k=5),

data= moss2,

family = gaussian(),

method = "GCV.Cp")

## Model 2 (spatial coordinates only)

mod_spatial_only <- gam(SpeciesRichness5~s(Latitude, Longitude, bs="ds", k=10),

data= moss2,

family = gaussian(),

method = "GCV.Cp")

## Model 3 (elevation + spatial coordinates)

mod_both <- gam(SpeciesRichness5~s(Elevation, bs="cr", k=5) +

s(Latitude, Longitude, bs="ds", k=10),

data= moss2,

family = gaussian(),

method = "GCV.Cp")

## Compare their explained variance (R^2^)

summary(mod_gam_original)

summary(mod_spatial_only)

summary(mod_both)

**## 3.3. Characterize their elevational trends**

## We adopted a scale-specific modeling approach to balance rigor and biological interpretability. ## For c-values, *z*-values, and species richness at 0.0001, 0.01, 0.25, 1, and 100 m^2^ scales (no

## significant spatial autocorrelation), we retained the original GAM model (elevation only) to

## characterize their elevational trends, as spatial structure did not confound these relationships.

## For species richness at the 25 m^2^ scale (independent spatial effect), we incorporated latitude

## and longitude as a 2D smooth term into the GAM to control for spatial autocorrelation,

## ensuring the elevational trend was not confounded by independent spatial structure. Taking *c*-## value and species richness at 25 m^2^ as examples.

## *c*

mod_gam <- gam(c~s(Elevation, bs="cr", k = 5),

data= moss2,

weights = 1/c_se,

family = gaussian(),

method = "GCV.Cp")

summary(mod_gam)

## basic diagnostics (residual normality, homoscedasticity, etc.)

## gam.check(mod_gam)

## calculate the percentage of deviance explained for overfitting judgment

## deviance_explained<- 1 - (mod_gam$deviance / mod_gam$null.deviance)

## GAM prediction

## fine grid of points

pd <- data.frame(Elevation = seq(1960.2, 3060, by = 0.1))

## get predicted response values from GAM

pr <- predict(mod_gam, newdata = pd, type = "response", se = TRUE)

## GAM plotting

with(moss2,

plot(Elevation, c, las=1, cex.lab=1.4, cex.main=1.4, cex.axis=1.1, cex=1.1,

xlab="Elevation (m a.s.l.)", ylab=expression(italic(c)-value),

col=rgb(red=0.2, green=0.2, blue=0.2, alpha=0.3)))

## Polygon

## plot predicted fit

lines(pd$Elevation, pr$fit,lwd=2)

polygon(c(pd$Elevation,rev(pd$Elevation)),

c(pr$fit - qnorm(0.975) * pr$se.fit,rev(pr$fit + qnorm(0.975) * pr$se.fit)),

col=rgb(red=0, green=0, blue=0.1, alpha=0.5),border=NA)

text(2100,15.5,expression(italic(R^2)== 0.478),cex=1)

text(2095,14.6,expression(italic(p)< 0.001),cex=1)

mtext("(a)", side=2,las=1,line=3,padj=-15.8,cex=1.2)

## Species richness at 25 m^2^

mod_both <- gam(SpeciesRichness5~s(Elevation, bs="cr", k=5) +

s(Latitude, Longitude, bs="ds", k=10),

data= moss2,

family = gaussian(),

method = "GCV.Cp")

summary(mod_both)

## GAM prediction

pd <- data.frame(

Elevation = seq(1960.2, 3060, by = 0.1),

Latitude = mean(moss2$Latitude),

Longitude = mean(moss2$Longitude)

)

pr <- predict(mod_both, newdata = pd, type = "response", se = TRUE)

## GAM plotting

ppi<-900

jpeg("SppRich5~Elevation.jpg",width=6*ppi,height=6*ppi,res=ppi)

par(mfcol=c(1,1), mar=c(4.5,4.5,1,1))

with(moss2,

plot(Elevation, SpeciesRichness5, las=1, cex.lab=1.4, cex.main=1.4,

cex.axis=1.1,cex=1.1,xlab="Elevation (m a.s.l.)",

ylab="Species richness",

col=rgb(red=0.2, green=0.2, blue=0.2, alpha=0.3)))

## Polygon

lines(pd$Elevation, pr$fit,lwd=2)

polygon(c(pd$Elevation,rev(pd$Elevation)),

c(pr$fit - qnorm(0.975) * pr$se.fit,rev(pr$fit + qnorm(0.975) * pr$se.fit)),

col=rgb(red=0, green=0, blue=0.1, alpha=0.5),border=NA)

text(2067,26,expression(italic(R^2)== 0.396),cex=1)

text(2057,24.2,expression(italic(p)< 0.001),cex=1)

mtext("(g)", side=2,las=1,line=3,padj=-16.3,cex=1.2)

dev.off()

**## Analysis 4. Boosted regression trees (BRT) models**

## Take BRT model for *c*-value as an example. moss3 is a dataset consisting of 13 columns, with ## the first column displaying *c*-value, the second to tenth columns displaying 9 predictors, the

## 11^th^ column displaying c_se, the 12^th^ column displaying longitude (x), and the 13^th^ column

## displaying latitude (y).

## The weighting term “weights = 1/c_se” and “weights = 1/z_se” were implemented in the BRTs ## for *c*- and *z*-values to mitigate the uncertainty associated with *c* and *z*. The larger the c_se or

## z_se value, the smaller the corresponding weight; the model thus dampens the influence of

## such *c* or *z* values, characterized by high estimation error and low reliability, on the fitting

## results. Whereas the weighting term were not implemented in the BRTs for species richness.

**## 4.1. Train model**

library(ade4)

library(MASS)

library(vegan)

library(ecodist)

library(rpart)

library(splines)

library(gam)

library(pgirmess)

library(utils)

library(combinat)

library(cluster)

library(fpc)

library(clusterSim)

library(lmtest)

library(Hmisc)

library(gplots)

library(NbClust)

library(rpart)

library(rpart.plot)

library(dismo)

library(multcomp)

library(gbm)

library(raster)

library(ggplot2)

library(rsample)

library(Metrics)

library(tidyverse)

library(spdep)

library(sf)

library(sp)

moss3 <- read.csv(file.choose())

## create hyperparameter grid

hyper_grid <- expand.grid(

learning.rate = c(0.0001,0.0005,0.001, 0.005, 0.01, 0.05),

tree.complexity = seq(1, 10, by = 1),

bag.fraction = seq(0.5, 0.75, 0.05)

)

## total number of combinations

nrow(hyper_grid)

ncol(hyper_grid)

## possible combinations of the three meta-parameters (learning rate, tree complexity, bag fraction) ## for fitted 360 BRT models based on 10-fold cross validation (CV)

for(i in 1:nrow(hyper_grid)) {

## reproducibility

set.seed(818)

## train model

moss_brt <- gbm.step(data = moss3,

gbm.x = c(2:10),

gbm.y = 1,

weights = 1/c_se,

family = "gaussian",

tree.complexity = hyper_grid$tree.complexity[i],

learning.rate = hyper_grid$learning.rate[i],

bag.fraction = hyper_grid$bag.fraction[i],

n.trees = 100,

max.trees = 60000,

verbose = TRUE)

## number of trees

hyper_grid$n_trees[i] <- list(moss_brt$n.trees)

## cross-validated (CV) deviance

hyper_grid$cv_deviance[i] <- list(moss_brt$cv.statistics["deviance.mean"])

## calculating mean CV correlation

hyper_grid$cv_correlation[i] <- list(moss_brt$cv.statistics["correlation.mean"])

## calculating training data correlation

hyper_grid$train_correlation[i] <- list(moss_brt$self.statistics["correlation"])

## calculating RMSE

hyper_grid$rmse[i] <- rmse(moss3$c, moss_brt$fitted)

## calculating mean square error (MSE)

hyper_grid$MSE[i] <- mse(moss3$c, moss_brt$fitted)

## calculating mean absolute error (MAE)

hyper_grid$MAE[i] <- mae(moss3$c, moss_brt$fitted)

## calculating R square

P <- function(a, b) {

if (is.null(a) || is.null(b)) {

return(NaN)

}

if (b == 0) {

return(NaN)

}

1 - a / b

}

hyper_grid$R2[i] <- P(moss_brt$self.statistics$mean.resid,moss_brt$self.statistics$mean.null)

## mean deviance for a null model

hyper_grid$Mean_deviance_null_model[i] <- list(moss_brt$cv.statistics["deviance.mean"])

}

## show results

hyper_grid

learning_rate <- hyper_grid$learning.rate

tree_complexity <- hyper_grid$tree.complexity

bag_fraction <- hyper_grid$bag.fraction

cv_deviance <- as.numeric(unlist(hyper_grid$cv_deviance))

cv_correlation <- as.numeric(unlist(hyper_grid$cv_correlation))

train_correlation <- as.numeric(unlist(hyper_grid$train_correlation))

RMSE <- hyper_grid$rmse

MSE <- hyper_grid$MSE

MAE <- hyper_grid$MAE

R2 <- hyper_grid$R2

non_nan_pos <- which(!is.nan(R2))

cv_deviance_new <- rep(NaN, length(R2))

cv_deviance_new[non_nan_pos] <- cv_deviance

cv_deviance_new

cv_correlation_new <- rep(NaN, length(R2))

cv_correlation_new[non_nan_pos] <- cv_correlation

cv_correlation_new

train_correlation_new <- rep(NaN, length(R2))

train_correlation_new[non_nan_pos] <- train_correlation

train_correlation_new

## The mean deviance for a null model for c-value is 8.536.

mean_deviance_null_model <- 8.536

percentage_explained_deviance <- (1-cv_deviance_new/mean_deviance_null_model)*100

cv_deviance_new <- round(cv_deviance_new, digits = 3)

percentage_explained_deviance <- round(percentage_explained_deviance, digits = 3)

cv_correlation_new <- round(cv_correlation_new, digits = 3)

train_correlation_new <- round(train_correlation_new, digits = 3)

RMSE <- round(RMSE, digits = 3)

MAE <- round(MAE, digits = 3)

R2 <- round(R2, digits = 3)

## BRTResult recorded cross-validated performance of train models from gbm.step

BRTResult<-data.frame(learning_rate, tree_complexity, bag_fraction, cv_deviance_new,

percentage_explained_deviance, cv_correlation_new,

train_correlation_new, RMSE, MAE, R2)

**## 4.2. Final BRT**

## set seed to guarantee the consistency of output

set.seed(818)

BRT.final <- gbm.step(data = moss3,

gbm.x = c(2:10),

gbm.y = 1,

weights = 1/c_se,

family = "gaussian",

tree.complexity = 3,

learning.rate = 0.01,

bag.fraction = 0.75,

n.trees = 100,

max.trees = 60000,

verbose = TRUE)

**## 4.3. Conditional Variable Importance (CVI), a metric that controls for other ## variables and mitigates multicollinearity, was calculated using the vip package**

library(vip)

library(ggplot2)

set.seed(123)

cvi_result <- vip(

BRT.final,

method = "permute",

nsim = 20,

train = moss3,

target = moss3$c,

metric = "rmse",

pred_wrapper = function(object, newdata) {

predict(object, newdata = newdata, n.trees = object$n.trees)

}

)

## examine and visualize CVI results

cvi_result

cvi_numeric <- cvi_result$data

cvi_simplified <- cvi_numeric[, c("Variable", "Importance")]

print(cvi_simplified)

## manually normalize CVI scores and convert to percentages (total sum = 100)

cvi_total <- sum(cvi_numeric$Importance)

cvi_numeric$Importance_Percent <- (cvi_numeric$Importance / cvi_total) * 100

cvi_numeric_percent <- cvi_numeric[order(cvi_numeric$Importance_Percent,

decreasing = TRUE), ]

## we regard the normalized CVI percentage results as the relative influence (RI) of each variable

var <- cvi_numeric_percent$Variable

rel.inf <- as.numeric(cvi_numeric_percent$Importance_Percent)

## Below is the r code used to generate Figure 5a of our manuscript.

df <- data.frame(

var = factor(var, levels = var),

rel.inf = rel.inf

)

df_sorted <- df[order(df$rel.inf), ]

ppi<-1200

jpeg("Factor importance.jpg",width=10*ppi,height=6*ppi,res=ppi)

ggplot(df_sorted, aes(x = rel.inf, y = reorder(var, rel.inf))) +

geom_col(aes(fill = rel.inf), width = 0.7) +

scale_fill_gradient(low = "#B5D3E7", high = "#1E5F8C", guide = "none") +

labs(title = expression(paste(italic(c),"-value")),

x = "Relative influence (%)",

y = "") +

theme_bw() +

theme(

panel.background = element_rect(fill = "white", colour = "black"),

panel.grid.major.y = element_blank(),

panel.grid.minor.y = element_blank(),

panel.grid.major.x = element_blank(),

panel.grid.minor.x = element_blank(),

axis.title.x = element_text(size = 14, margin = margin(t = 10)),

axis.text = element_text(size = 12, color = "black"),

plot.title = element_text(size = 14, face = "bold", hjust = 0.5),

panel.border = element_blank(),

axis.line.x = element_line(color = "black")

) +

xlim(0, max(df_sorted$rel.inf) * 1)

dev.off()

**## 4.4. Partial dependence plot for each predictor**

## Below is the r code used to generate Figure 3 of our manuscript. Take NPP as an example.

ppi<-500

jpeg("PartialDependence_C_NPP.jpg",width=6*ppi,height=6*ppi,res=ppi)

par(mfrow = c(1, 1), mar = c(4.2, 4.2, 1, 1))

gbm.plot(gbm.object = BRT.final,

variable.no = 8, n.plots=1, plot.layout=c(1, 1), rug = TRUE,

smooth = TRUE, write.title = FALSE,common.scale=T,

x.label="NPP", y.label="Marginal effect on c-value",

show.contrib=F, cex.axis=1.2, cex.lab=1.5)

dev.off()

**## 4.5. Important interactive effects**

gbm.interactions(BRT.final)$rank.list

## interactive effect between NPP and Bio1

ppi<-1200

jpeg("c-value_Inter1.jpg",width=6*ppi,height=6*ppi,res=ppi)

gbm.perspec(BRT.final,

8,

2,

x.label = "NPP",

y.label = "Bio1",

z.label = "c-value",

main = "Interactive effect",

z.range = c(7.8, 13.55),

col="lightblue",phi = 30, theta = 135,

cex.axis = 0.5,

cex.lab=1.2,

smooth = "average")

mtext("(a)", side=2,las=1,line=-0.5,padj=-20)

dev.off()

## interactive effect between NPP and Bio12

ppi<-1200

jpeg("c-value_Inter2.jpg",width=6*ppi,height=6*ppi,res=ppi)

gbm.perspec(BRT.final,

8,

5,

x.label = "NPP",

y.label = "Bio12",

z.label = "c-value",

main = "Interactive effect",

z.range = c(8.8, 14.02),

col="lightblue",phi = 30, theta = 135,

cex.axis = 0.5,

cex.lab=1.2,

smooth = "average")

mtext("(b)", side=2,las=1,line=-0.5,padj=-20)

dev.off()

## interactive effect between NDVI and NPP

ppi<-1200

jpeg("c-value_Inter3.jpg",width=6*ppi,height=6*ppi,res=ppi)

gbm.perspec(BRT.final,

9,

8,

x.label = "NDVI",

y.label = "NPP",

z.label = "c-value",

main = "Interactive effect",

z.range = c(8.6, 13.62),

col="lightblue",phi = 30, theta = 135,

cex.axis = 0.5,

cex.lab=1.2,

smooth = "average")

mtext("(c)", side=2,las=1,line=-0.5,padj=-20)

dev.off()

## interactive effect between NPP and HD

ppi<-1200

jpeg("c-value_Inter4.jpg",width=6*ppi,height=6*ppi,res=ppi)

gbm.perspec(BRT.final,

8,

1,

x.label = "NPP",

y.label = "HD",

z.label = "c-value",

main = "Interactive effect",

z.range = c(7.3, 13.58),

col="lightblue",phi = 30, theta = 65,

cex.axis = 0.5,

cex.lab=1.2,

smooth = "average")

mtext("(d)", side=2,las=1,line=-0.5,padj=-20)

dev.off()

**## 4.6. Test for spatial autocorrelation**

glimpse(moss[, c("x", "y")])

moss_sf <- st_as_sf(

x = moss3,

coords = c("x", "y"),

crs = 4326, # Set CRS to WGS_1984 (EPSG:4326)

remove = FALSE

)

## clean missing values (remove rows with any NA to avoid BRT training errors)

moss_clean <- na.omit(moss_sf)

cat("Number of valid spatial sampling points:", nrow(moss_clean), "\n")

## extract coordinate matrix for k-nearest neighbors calculation

moss_coords <- st_coordinates(moss_clean)

## identify 5 nearest neighbors for each point (standard for point data)

moss_knn <- knearneigh(

x = moss_coords,

k = 5,

longlat = TRUE

)

## convert k-nearest neighbors to a neighbor (nb) object

moss_nb <- knn2nb(moss_knn)

## convert neighbor list to row-standardized spatial weights matrix

## style "W" = row-standardization (weights sum to 1 for each point – ideal for Moran’s *I*)

moss_weights <- nb2listw(

moss_nb,

style = "W"

)

## inspect the spatial weights matrix (verify construction success)

summary(moss_weights)

cat("Number of neighbors for the first 5 points:\n")

print(head(card(moss_nb), 5))

## extract optimal number of trees (automatically selected by gbm.step())

best_trees <- BRT.final$gbm.call$best.trees

cat("Optimal number of BRT trees:", best_trees, "\n")

## calculate BRT predictions and residuals

moss_df <- st_drop_geometry(moss_clean)

moss_df$brt_pred <- predict(

object = BRT.final,

newdata = moss_df,

n.trees = best_trees

)

moss_df$brt_resid_raw <- moss_df$c - moss_df$brt_pred

moss_df$brt_resid_std <- as.vector(scale(moss_df$brt_resid_raw))

moss_clean$brt_resid_std <- moss_df$brt_resid_std

## Moran’s *I* test for standardized BRT residuals (preferred)

moran_brt <- moran.test(

x = moss_clean$brt_resid_std,

listw = moss_weights,

zero.policy = TRUE,

alternative = "two.sided" # Two-tailed test (detect positive/negative autocorrelation)

)

## print the Moran's I test results

cat("\n=== Moran's I Test for BRT Model Residuals (Moss Dataset) ===\n")

print(moran_brt)

## Below is the r code used to generate Figure S10 of our manuscript.

ppi<-1200

jpeg("BRT residuals_c_final.jpg",width=8*ppi,height=4*ppi,res=ppi)

par(mfrow = c(1, 1), mar = c(1, 4.2, 1, 1))

lon_lat <- st_coordinates(moss_clean)

max_lon <- max(lon_lat[, 1])

max_lat <- max(lon_lat[, 2])

ggplot(moss_clean) +

geom_sf(aes(color = brt_resid_std), size = 3, alpha = 0.7) +

scale_color_gradient2(

low = "blue",

mid = "white",

high = "red",

midpoint = 0,

name = "Standardized\nBRT residuals"

) +

labs(

title = "Spatial distribution of BRT model residuals",

subtitle = "c-value: observed - predicted"

) +

annotate(

geom = "text",

x = max_lon*0.99983,

y = max_lat,

label = expression(paste("Moran's ", italic(I), " = -0.084")),

hjust = 0,

vjust = 1,

size = 4,

color = "black",

fontface = "plain"

) +

annotate(

geom = "text",

x = max_lon*0.99983,

y = max_lat - (max_lat - min(lon_lat[, 2])) * 0.09,

label = expression(paste(italic(p), " = 0.369")),

hjust = 0,

vjust = 1,

size = 4,

color = "black"

) +

theme_minimal()+

theme(

axis.title.x = element_blank(),

axis.title.y = element_blank()

)

dev.off()

**## Analysis 5. Relative influence (RI)–scale regressions**

## moss4 is a dataset consisting of 12 columns, with the first column displaying logSpatial, the

## second displaying *R*^2^ of BRTs, the 3^rd^ to 12^th^ columns displaying RI of each predictor at each ## spatial scale.

moss4 <- read.csv(file.choose())

## test for significance

model <- lm(R2 ~ logSpatial, data = moss4)

summary(model)

model <- lm(MDE ~ logSpatial, data = moss4)

summary(model)

model <- lm(Bio14 ~ logSpatial, data = moss4)

summary(model)

model <- lm(HD ~ logSpatial, data = moss4)

summary(model)

model <- lm(NPP ~ logSpatial, data = moss4)

summary(model)

model <- lm(Bio1 ~ logSpatial, data = moss4)

summary(model)

model <- lm(NDVI ~ logSpatial, data = moss4)

summary(model)

model <- lm(Bio12 ~ logSpatial, data = moss4)

summary(model)

model <- lm(Bio7 ~ logSpatial, data = moss4)

summary(model)

model <- lm(Bio6 ~ logSpatial, data = moss4)

summary(model)

model <- lm(WS ~ logSpatial, data = moss4)

summary(model)

moss4$MDE <- data$MDE/100

moss4$Bio14 <- data$Bio14/100

moss4$NPP <- data$NPP/100

moss4$NDVI <- data$NDVI/100

moss4$Bio7 <- data$Bio7/100

## model six linear regressions

model1 <- lm(R2 ~ logSpatial, data =moss4)

model2 <- lm(MDE ~ logSpatial, data = moss4)

model3 <- lm(Bio14 ~ logSpatial, data = moss4)

model4 <- lm(NPP ~ logSpatial, data = moss4)

model5 <- lm(NDVI ~ logSpatial, data = moss4)

model6 <- lm(Bio7 ~ logSpatial, data = moss4)

## Below is the r code used to generate Figure 5 of our manuscript.

ppi<-1200

jpeg("significant_changes.jpg",width=6*ppi,height=6*ppi,res=ppi)

par(mfcol=c(1,1), mar=c(4,4,1,1))

plot(moss4$logSpatial, moss4$R2, col = "blue", pch = 16, ylim =c(0,0.8), cex.lab=1.2,

xlab = "", ylab = "Value")

title(xlab = expression(log*' '*(Spatial*' '*scale)*' '*(m^2)),cex.lab=1.2)

points(moss4$logSpatial, data$MDE, col = "red", pch = 16)

points(moss4$logSpatial, data$Bio14, col = "green", pch = 16)

points(moss4$logSpatial, data$NPP, col = "black", pch = 16)

points(moss4$logSpatial, data$NDVI, col = "orange", pch = 16)

points(moss4$logSpatial, data$Bio7, col = "purple", pch = 16)

abline(model1, col = "blue", lwd = 2)

abline(model2, col = "red", lwd = 2)

abline(model3, col = "green", lwd = 2)

abline(model4, col = "black", lwd = 2)

abline(model5, col = "orange", lwd = 2)

abline(model6, col = "purple", lwd = 2)

legend("topleft", legend = c("MDE","Bio14","NPP","NDVI","Bio7",bquote(italic(R)^2)),

col = c("red","green","black","orange","purple","blue"), pch = c(16, 16,16,16,16,16),

lwd = 2, ncol=3)

dev.off()

**## Analysis 6. Comparison of *c*- and *z*-values across four main environments**

## prepare a dataset consisting of 3 columns, with the first column displaying habitat, the

## second displaying *c_*value, the 3^rd^ displaying *z*_value.

## Below is the r code used to generate Figure S2 of our manuscript.

library(ggplot2)

library(dplyr)

library(ggsignif)

library(ggpubr)

czdata <- read.csv(file.choose())

czdata

## *c*-value

violin_plot <- ggplot(czdata, aes(x = Habitat, y = c_value, fill = Habitat)) +

geom_violin(scale = "width", alpha = 0.7, size = 0.8) +

geom_boxplot(width = 0.2, color = "black", size = 0.6, outlier.shape = NA) +

geom_jitter(shape = 21, size = 1.5, alpha = 0.6, position = position_jitter(0.1)) +

scale_fill_manual(values = c("#66c2a5", "#fc8d62", "#8da0cb", "#e78ac3")) +

geom_signif(

comparisons = list(c("R", "M"),

c("M", "F"),

c("F", "G"),

c("R", "G")),

map_signif_level = TRUE,

y_position = c(16.0, 17.0, 18.0, 19.0),

tip_length = 0.01,

size = 0.6,

test = "t.test",

test.args = list(var.equal = TRUE,

alternative = "two.sided",

conf.level = 0.95)

) +

labs(

x = "Habitat type",

y = expression(paste(italic(c),"-value")),

title = " ",

fill = "Habitat"

) +

theme_bw() +

theme(

plot.title = element_text(hjust = 0.5, size = 14, face = "bold"),

axis.title.x = element_text(size = 12),

axis.title.y = element_text(size = 12),

legend.position = "none"

) +

coord_cartesian(ylim = c(2, 20))

ppi<-1200

jpeg("Comparison_c.jpg",width=6*ppi,height=6*ppi,res=ppi)

par(mfrow = c(1, 1), mar = c(4.2, 4.2, 1, 1))

print(violin_plot)

dev.off()

## *z*-value

violin_plot <- ggplot(czdata, aes(x = Habitat, y = z_value, fill = Habitat)) +

geom_violin(scale = "width", alpha = 0.7, size = 0.8) +

geom_boxplot(width = 0.2, color = "black", size = 0.6, outlier.shape = NA) +

geom_jitter(shape = 21, size = 1.5, alpha = 0.6, position = position_jitter(0.1)) +

scale_fill_manual(values = c("#66c2a5", "#fc8d62", "#8da0cb", "#e78ac3")) +

geom_signif(

comparisons = list(c("R", "M"),

c("M", "F"),

c("F", "G"),

c("R", "G")),

map_signif_level = TRUE,

y_position = c(0.20, 0.208, 0.22, 0.228),

tip_length = 0.01,

size = 0.6,

test = "t.test",

test.args = list(var.equal = TRUE,

alternative = "two.sided",

conf.level = 0.95)

) +

labs(

x = "Habitat type",

y = expression(paste(italic(z),"-value")),

title = " ",

fill = "Habitat"

) +

theme_bw() +

theme(

plot.title = element_text(hjust = 0.5, size = 14, face = "bold"),

axis.title.x = element_text(size = 12),

axis.title.y = element_text(size = 12),

legend.position = "none"

) +

coord_cartesian(ylim = c(0.05,0.24))

ppi<-1200

jpeg("Comparison_z.jpg",width=6*ppi,height=6*ppi,res=ppi)

par(mfrow = c(1, 1), mar = c(4.2, 4.2, 1, 1))

print(violin_plot)

dev.off()

**## Analysis 7. Sensitivity analyses including drop-one-variable sensitivity**

**## analysis and perturbation sensitivity analysis**

## Below is the r code used to generate Figure S9 of our manuscript. Take *c*-value as an example.

library(dplyr)

library(gbm)

library(ggplot2)

library(purrr)

moss_data <- read.csv(file.choose())

head(moss_data)

## c_value

set.seed(123)

brt_base <- gbm(

formula = c ~ HD + Bio1 + Bio6 + Bio7 + Bio12 + Bio14 + WS + NPP + NDVI,

data = moss_data,

distribution = "gaussian",

n.trees = 1000,

interaction.depth = 3,

shrinkage = 0.01,

bag.fraction = 0.75,

verbose = FALSE

)

## Determine the optimal number of trees (to avoid overfitting)

best_trees <- gbm.perf(brt_base, method = "OOB")

cat("Optimal Number of Trees for the BRT Baseline Model =", best_trees, "\n")

## Calculate the R-squared (explanatory power) of the baseline model

pred_base <- predict(brt_base, newdata = moss_data, n.trees = best_trees)

r2_base <- 1 - sum((pred_base - moss_data$c)^2) /

sum((moss_data$c - mean(moss_data$c))^2)

cat("BRT Baseline Model R2 =", round(r2_base, 3), "\n")

**## 7.1 Sensitivity Analysis 1: Sequentially dropping one variable at a time**

**## (drop-one-variable)**

## Validate the model stability against single-variable elimination

vars <- c("HD","Bio1","Bio6","Bio7","Bio12","Bio14","WS","NPP","NDVI")

drop_results <- data.frame()

for (v in vars) {

formula_i <- formula(paste0("c ~ ", paste(setdiff(vars, v), collapse = " + ")))

brt_i <- gbm(

formula = formula_i,

data = moss_data,

distribution = "gaussian",

n.trees = 1000,

interaction.depth = 3,

shrinkage = 0.01,

bag.fraction = 0.75,

verbose = FALSE

)

best_trees_i <- gbm.perf(brt_i, method = "OOB")

pred_i <- predict(brt_i, newdata = moss_data, n.trees = best_trees_i)

r2_i <- 1 - sum((pred_i - moss_data$c)^2) /

sum((moss_data$c - mean(moss_data$c))^2)

drop_results <- rbind(drop_results, data.frame(

dropped_var = v,

best_trees = best_trees_i,

R2 = r2_i

))

}

## Supplement the baseline *R*^2^ and the change in *R*^2^

drop_results <- drop_results %>%

mutate(

R2_base = r2_base,

R2_change = R2 - r2_base

)

cat("\n==== Results of the sequential variable-drop sensitivity analysis ====\n")

print(drop_results)

**## 7.2 Sensitivity Analysis 2: Adding small perturbations (5%) to the**

**## environmental variables downloaded from satellite-derived data**

**## (perturbation)**

n_iter <- 1000

perturb_results <- data.frame()

for (i in 1:n_iter) {

dat_i <- moss_data %>%

mutate(

Bio1 = Bio1 + rnorm(n, 0, sd(Bio1)*0.05),

Bio6 = Bio6 + rnorm(n, 0, sd(Bio6)*0.05),

Bio7 = Bio7 + rnorm(n, 0, sd(Bio7)*0.05),

Bio12 = Bio12 + rnorm(n, 0, sd(Bio12)*0.05),

Bio14 = Bio14 + rnorm(n, 0, sd(Bio14)*0.05),

WS = WS + rnorm(n, 0, sd(WS) *0.05),

NPP = NPP + rnorm(n, 0, sd(NPP) *0.05),

NDVI = NDVI + rnorm(n, 0, sd(NDVI) *0.05)

)

brt_i <- gbm(

formula = c ~ HD + Bio1 + Bio6 + Bio7 + Bio12 + Bio14 + WS + NPP + NDVI,

data = dat_i,

distribution = "gaussian",

n.trees = 1000,

interaction.depth = 3,

shrinkage = 0.01,

bag.fraction = 0.75,

verbose = FALSE

)

best_trees_i <- gbm.perf(brt_i, method = "OOB")

pred_i <- predict(brt_i, newdata = moss_data, n.trees = best_trees_i)

r2_i <- 1 - sum((pred_i - moss_data$c)^2) /

sum((moss_data$c - mean(moss_data$c))^2)

perturb_results <- rbind(perturb_results, data.frame(

iter = i,

best_trees = best_trees_i,

R2 = r2_i

))

}

cat("\n==== Results of Noise Perturbation Sensitivity Analysis (1000 iterations) ====\n")

print(summary(perturb_results$R2))

## visualization

## Figure 1: Changes in *R*^2^ after variable elimination (red dashed line = baseline *R*^2^)

p1 <- ggplot(drop_results, aes(x = dropped_var, y = R2)) +

geom_col(fill = "#2E86AB", alpha = 0.8, width = 0.6) +

geom_hline(yintercept = r2_base, linetype = "dashed", color = "red", linewidth = 1) +

labs(

title = "Drop-one-variable sensitivity (BRT, c_value)",

x = "Dropped variable",

y = "R² (Explained variance)"

) +

theme_bw() +

theme(plot.title = element_text(hjust = 0.5))

ppi<-1200

jpeg("Dropping_one_variable_c.jpg",width=6*ppi,height=6*ppi,res=ppi)

par(mfrow = c(1, 1), mar = c(4.2, 4.2, 4.2, 1))

print(p1)

dev.off()

## Figure 2: Distribution of *R*^2^ after noise perturbation (red dashed line = baseline *R*^2^)

p2 <- ggplot(perturb_results, aes(x = R2)) +

geom_histogram(fill = "#A23B72", alpha = 0.8, bins = 7) +

geom_vline(xintercept = r2_base, linetype = "dashed", color = "red", linewidth = 1) +

labs(

title = "Perturbation sensitivity (BRT, c_value)",

x = "R² (Explained variance)",

y = "Frequency"

) +

theme_bw() +

theme(plot.title = element_text(hjust = 0.5))

ppi<-1200

jpeg("Perturbation_c.jpg",width=6*ppi,height=6*ppi,res=ppi)

par(mfrow = c(1, 1), mar = c(4.2, 4.2, 4.2, 1))

print(p2)

dev.off()
